# Gene Function Hypotheses for the *Campylobacter jejuni* Glycome Generated by a Logic-Based Approach

**DOI:** 10.1016/j.jmb.2012.10.014

**Published:** 2013-01-09

**Authors:** Michael J.E. Sternberg, Alireza Tamaddoni-Nezhad, Victor I. Lesk, Emily Kay, Paul G. Hitchen, Adrian Cootes, Lieke B. van Alphen, Marc P. Lamoureux, Harold C. Jarrell, Christopher J. Rawlings, Evelyn C. Soo, Christine M. Szymanski, Anne Dell, Brendan W. Wren, Stephen H. Muggleton

**Affiliations:** 1 Centre for Integrative Systems Biology, Imperial College London, London SW7 2AZ, UK; 2 Division of Molecular Biosciences, Department of Life Science, Imperial College London, London SW7 2AZ, UK; 3 Department of Computing, Imperial College London, London SW7 2AZ, UK; 4 Department of Pathogen Molecular Biology, London School of Hygiene and Tropical Medicine, Keppel Street, London WC1E 7HT, UK; 5 Alberta Glycomics Centre, Department of Biological Sciences, University of Alberta, Edmonton, Alberta, Canada T6G 2E9; 6 Institute for Biological Sciences, National Research Council, Ottawa, Ontario, Canada K1A 0R6; 7 Department of Computational and Systems Biology, Rothamsted Research, Harpenden AL5 2JQ, UK; 8 Institute for Marine Biosciences, National Research Council, Halifax, Nova Scotia, Canada B3H 3Z1

**Keywords:** ILP, inductive logic programming, CPS, capsular polysaccharide, HR-MAS, high-resolution magic angle spinning, CE-ESMS, capillary electrophoresis coupled to electrospray mass spectrometry, BBSRC, Biotechnology and Biological Sciences Research Council, systems biology, *Campylobacter jejuni*, machine learning, capsular polysaccharide, pathway modelling

## Abstract

Increasingly, experimental data on biological systems are obtained from several sources and computational approaches are required to integrate this information and derive models for the function of the system. Here, we demonstrate the power of a logic-based machine learning approach to propose hypotheses for gene function integrating information from two diverse experimental approaches. Specifically, we use inductive logic programming that automatically proposes hypotheses explaining the empirical data with respect to logically encoded background knowledge. We study the capsular polysaccharide biosynthetic pathway of the major human gastrointestinal pathogen *Campylobacter jejuni*. We consider several key steps in the formation of capsular polysaccharide consisting of 15 genes of which 8 have assigned function, and we explore the extent to which functions can be hypothesised for the remaining 7. Two sources of experimental data provide the information for learning—the results of knockout experiments on the genes involved in capsule formation and the absence/presence of capsule genes in a multitude of strains of different serotypes. The machine learning uses the pathway structure as background knowledge. We propose assignments of specific genes to five previously unassigned reaction steps. For four of these steps, there was an unambiguous optimal assignment of gene to reaction, and to the fifth, there were three candidate genes. Several of these assignments were consistent with additional experimental results. We therefore show that the logic-based methodology provides a robust strategy to integrate results from different experimental approaches and propose hypotheses for the behaviour of a biological system.

## Graphical Abstract

**Figure:**
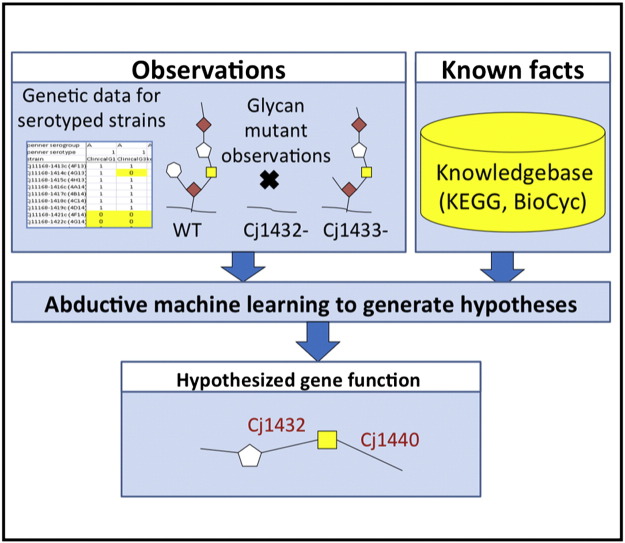

**Highlights:**

► A challenge in systems biology modelling is to integrate different data sources. ► A logic-based approach was used to hypothesise gene function for the *C. jejuni* glycome. ► Gene knockout and serotype strain data were integrated with pathway information. ► Functions were hypothesised for capsule polysaccharide biosynthetic pathway genes. ► Logic-based learning proposed hypotheses for the behaviour of a biological system.

## Introduction

**Fig. 1:**
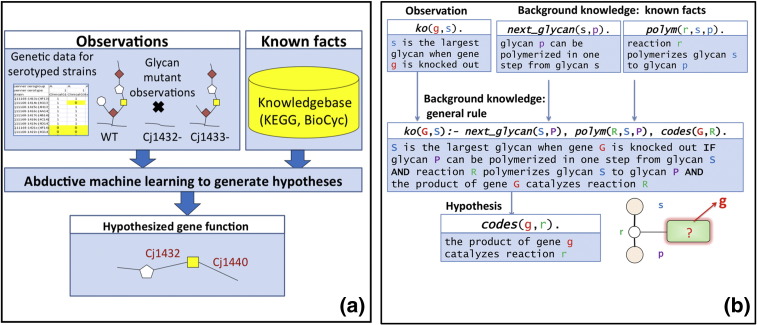
The logic-based machine learning approach to hypothesise functions for genes in the CPS synthetic pathway of *C. jejuni.* (a) Overview of machine learning. The background knowledge, consisting of known facts and rules, is used as a basis for abductive inference of novel hypothetical gene functions that might explain the experimental observations of single-knockout glycan phenotypes. (b) Simple system for abductive inference of gene functions in a metabolic network. Gene functions, represented by instances of the *codes* predicate, are hypothesised by the ILP program, based on the background knowledge of reactions, with the aim of explaining observed knockout phenotypes represented by the *ko* predicate. Capital letters G, R and S represent classes of object (genes, reactions, etc.) while small letters represent specific instances.

Biological systems are made up of very large numbers of different components interacting at various scales. Systems biology provides a framework for integrating biological information from a variety of experimental sources to support analysis and prediction.^1,2^ A major challenge in systems biology modelling is to develop techniques that can readily and robustly integrate information from diverse sources, often including the results of high-throughput experiments. The scale and incompleteness of existing biological data sources has made machine learning a common approach to the construction of systems biology models (e.g., Refs. 3–9). A logic-based representation provides a rich and flexible approach for integrating data, models and hypotheses.^5,6,10^ In this paper, we investigate whether an incomplete biological network description can be automatically extended using both gene knockout and multi-strain microarray data within the context of a substantially incomplete network structure (see Fig. 1a). The approach employs a logic-based machine learning method to integrate this diverse information. This enables us to generate hypotheses assigning function to genes involved in the biosynthesis of the capsular polysaccharide (CPS) of the common food-borne pathogen, *Campylobacter jejuni*.

A wide variety of approaches have been employed to assign protein function from system-wide experimental data (see, e.g., review^11^). Many approaches aim to perform genome-wide predictions and integrate different sources of data using, for example, neural networks,^4^ Bayesian statistical analysis,^3^ flux balance analysis with Boolean logic^12^ and machine learnt data driven scoring schemes.^8^ Logic-based methods^5,6,9,10^ offer a number of major advantages. Logic-based statements can express a concept such as that a protein P1 converts compound A into B and that P2 converts B into C, and from this, it can be inferred that A can be converted into C, that is, the transitivity of conversion. Furthermore, this and other properties and constraints can be expressed using general definitions, which can then be applied to all instances in the network. This supports compact, hierarchically defined descriptions of features that are central to biological networks, such as inhibition,^6,9^ activation, compartmentalisation and temporal and spatial distribution. Additionally, there are well-developed methodologies for learning logic-based descriptions, in particular, inductive logic programming (ILP) (see below).^13^

*C*. *jejuni* is the leading bacterial cause of human gastroenteritis, and its cell surface displays at least four types of glycostructures, which play roles in pathogenesis,^14^ avoiding bacteriophage predation^15^ and interaction with the host immune system. One of these glycostructures is the CPS that is found on the surface of numerous bacterial species involved in diverse roles ranging from prevention of desiccation to immune evasion and pathogenesis. The original full genome sequence^16,17^ of *C*. *jejuni* strain NCTC 11168 identified a locus of 35 genes implicated in the synthesis and export of CPS, whose glycan structure has been fully determined.^18,19^ Here, we focus on several key biosynthetic steps in the formation of CPS and aim to predict function for the several unassigned gene products using abductive machine learning.

**Fig. 2:**
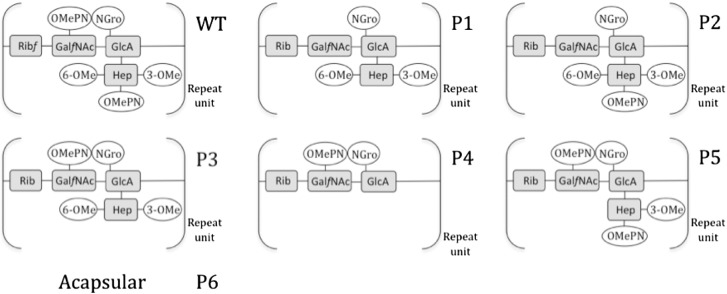
Schematic diagram of the glycan phenotypes described in Table 1. The wild type and knockout mutant repeat unit phenotypes (P1 to P6) are shown to highlight the loss of specific residues from the wild-type structure as a result of gene mutations. In the glycan structure, shading represents CPS sugar residues that form the repeat unit and clear ovals are non-sugar modifications. OMePN, phosphoramidate; NGro, *N*-glycerol; Rib, ribose; Gal*f*NAc, *N*-acetylgalactosamine in the furanose configuration; GlcA, glucuronic acid; 6-OMe, 6-*O*-Methyl; Hep, heptose, 3-OMe, 3-*O*-methyl.

**Fig. 3:**
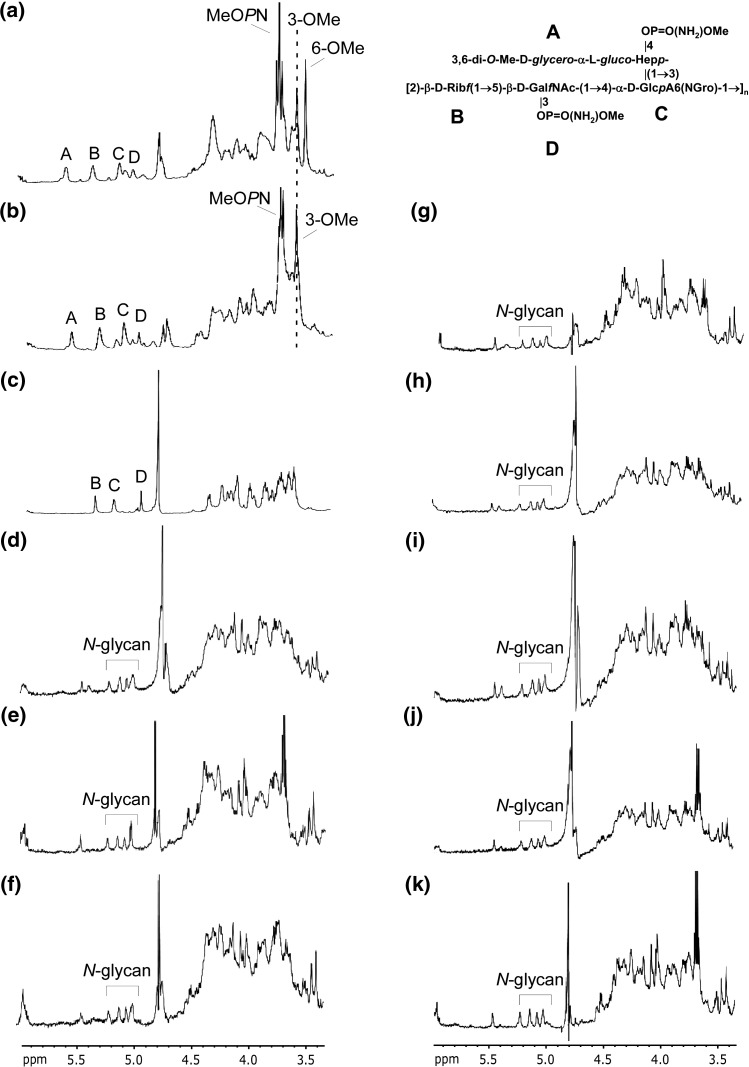
HR-MAS [^1^H]NMR analyses of the CPS of *C. jejuni* 11168 H and several mutants. (a) *C. jejuni* 11168 H; (b) Cj1426; (c) Cj1427; (d) Cj1430; (e) Cj1432; (f) Cj1434; (g) Cj1438; (h) Cj1440; (i) Cj1441; (j) Cj1442. The anomeric protons of the CPS correspond to (A) 3,6-di-*O*-methyl-d-glycero-α-l-glucoheptose, (B) β-d-ribose, (C) α-d-glucuronic acid amidated predominantly with 2-amino-2-deoxyglycerol and (D) β-d-Gal*f*NAc. Other labelled proton resonances include the phase variable *O*-methyl groups linked to heptose and the phosphoramidate (MeOPN) modifications linked to heptose and Gal*f*NAc. In the acapsular mutants, the anomeric resonances from the free oligosaccharides derived from the *N*-glycan pathway are visible and labelled.^22^ The top right corner depicts the structure of the repeating subunit of the capsule of 11168 H.

Two sources of experimental information were used in gene assignment. Of the 35 CPS genes, 28 representing the capsule biosynthesis region and excluding well-characterised export genes (*kpsS*, *C*, *F*, *D*, *E*, *T*, *M*) were systematically knocked out and the capsule structure was examined by high-resolution magic angle spinning (HR-MAS) NMR (Table 1 and Figs. 1–3). The second source of information is the absence or presence of the CPS genes obtained by DNA microarray analysis of 270 diverse *C*. *jejuni* strains (Supplementary Material).

## Results

### Machine learning approach

Our machine learning approach uses logic statements (encoded in the computer language Prolog^23^) as the basis for abductive reasoning. Each abductive step takes as input experimental observations and background knowledge and produces a hypothesis as output. We illustrate the process of abduction using a simplification of our full model consisting of glycans, genes and reactions (Fig. 1b). The figure shows the categories of logic statements with their corresponding Prolog clauses. The experimental observations take the form: s is the largest glycan when gene g is knocked out. There are two components to the background knowledge—known facts and general rules. The facts are of the form: (i) glycan p can be polymerised in one step from glycan s and (ii) reaction r polymerises glycan s to glycan p. The general rule in the background knowledge is: S is the largest glycan when gene G is knocked out if glycan P can be polymerised in one step from glycan S and reaction R polymerises glycan S to glycan P and the product of gene G catalyses reaction R. (In Prolog, lower case letters s, g, p and r are used for specific objects and capitals S, G, P and R are used for variables.)

The abductive hypothesis is automatically generated using the Progol ILP program (see Materials and Methods). This is achieved by matching the observations and known facts to the general rule in order to extract a hypothesis that explains the observations. In this example, abduction will generate the hypothesis that the product of gene g catalyses reaction r.

In our full model, many choices for matching can be made, leading to a variety of alternative hypotheses, and a preference is imposed by Progol using an information-theoretic criterion known as compression. Here, compression = *o* − *n* − *h*, where *o* is the number of observations correctly explained by the hypothesis, *n* is the number incorrectly explained and *h* is the length of the hypothesis (in this study, always 1 because the hypothesis is a single fact).

### Learning from gene knockout information

**Fig. 4:**
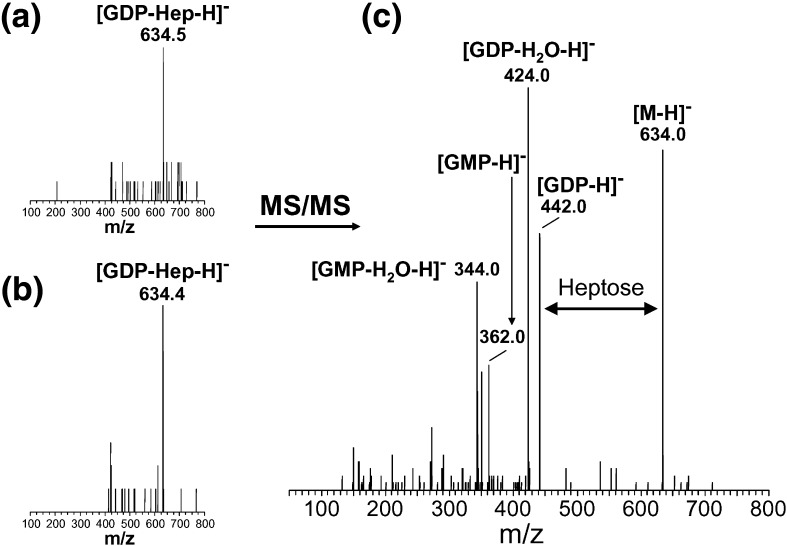
CE-ESMS and precursor ion scanning of *m*/*z* 424 for (a) Cj1430 and (b) Cj1427. The presence of GDP-heptose (*m*/*z* 634) in these two lysates was confirmed by CE-ES mass spectrometry shown in (c).

The experimental observations are the capsule phenotypes of gene knockout mutants using HR-MAS NMR.^19^ Known facts describe the reaction steps based on the known pathways for CPS syntheses. In addition, gene products whose functions have been determined are associated to the appropriate reaction step. Other known facts that were used are reported in Supplementary Material. Manually, we provide general rules describing principles of the mechanism of the synthetic pathway. In this study, to explore the power of this logic-based learning, we employ the assumption that if a gene is knocked out then the precursor of that particular reaction can be identified experimentally (as seen in Fig. 4). This is only a working assumption as feedback regulation in biosynthetic pathways might result in the precursor not being accumulated. However, the results in Fig. 4 confirm that CPS heptose biosynthesis does indeed utilise a GDP-Hep intermediate, as we proposed,^24^ and further supports the modelling and HR-MAS NMR results that Cj1427 and Cj1430 are involved in Hep biosynthesis. HR-MAS NMR analysis together with sequencing also confirmed that Cj1426 is responsible for adding the phase variable 6-*O*-Me residue onto Hep (Fig. 3b).

**Fig. 5:**
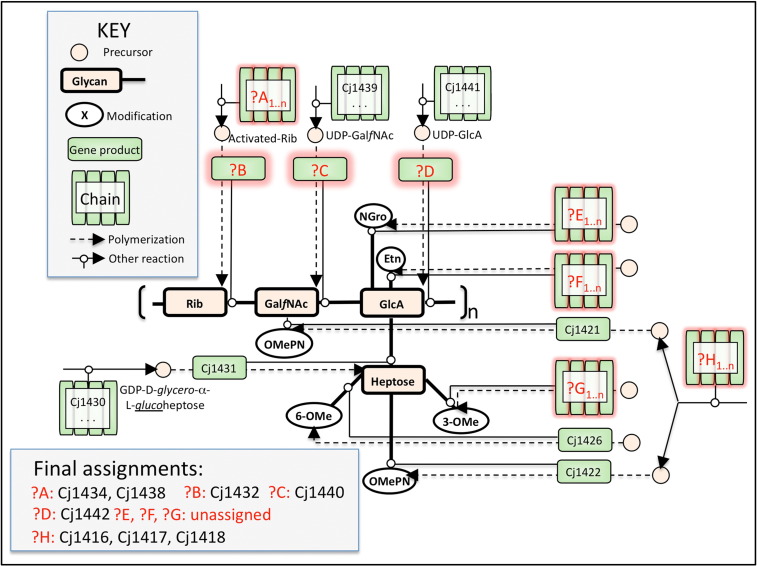
The CPS synthesis pathway of *C. jejuni* showing assigned gene functions. The pathway is shown in a representation based on Systems Biology Graphical Notation. The CPS structure is represented in bold, its non-sugar modifications being shown as white ellipses. Transformations are shown as small white circles. Precursors are represented as medium circles with peach fill. Chemical reactions are shown as broken lines, and enzymatic steps are shown as continuous lines. Genes are shown as green boxes, with sets of genes involved in a path shown as a green accordion. Where genes in a path are known, the final gene number is used to represent the chain, as in the precursor paths for Hep,^24^ Gal*f*NAc^21,25^ and GlcA^18^ and this study (involvement of 1427 and 1430 in Hep biosynthesis and Cj1426 in 6-OMe transfer). Genes of unknown identity are surrounded in red and labelled with a question mark. ?A, ribofuranose biosynthesis pathway; ?B, ribosyltransferase; ?C, *N*-acetyl galactofuranosamine transferase; ?D, glucuronic acid transferase; ?E, *N*-glycerol biosynthesis pathway; ?F, ethanolamine biosynthesis pathway; ?G, *O*-methyl donor biosynthesis pathway; ?H, phosphoramidate biosynthesis pathway.

We focus on the central part of the synthesis pathway leading to the formation of the CPS and consider 15 reactions or sequences of reaction steps (Fig. 5). In our modelling, specific enzymes were assigned to 7 of the 15 reactions and the remaining 8 reactions are considered as unknown (indicated by ?A to ?H) (see Table 1 and Fig. 5). The modelling challenge is to hypothesise which of the un-annotated enzymes perform these steps. Reactions at the perimeter of the pathway (?A,?E,?F,?G,?H) could be a series of enzyme reactions, and thus, several genes can be assigned. The presence of these chains of reactions is encoded in the background knowledge. Of these eight, Cj1416, Cj1417 and Cj1418 were known to be involved in reaction ?H from the HR-MAS NMR results^19^ showing that mutation of any of the genes Cj1416–Cj1418c leads to a capsule that lacks OMePN. Thus, Cj1416, Cj1417 and Cj1418 acted as a test of the modelling.

Table 2 (column 2) gives the results of learning from the knockout data. Certain reactions were not assigned to any gene (Cj1419, Cj1420, Cj1429, Cj1433 and Cj1436) as there were no relevant knockout observations. The reaction step denoted ?H is assigned to genes Cj1416, Cj1417 and Cj1418. However, each of the remaining reactions was ambiguously assigned to several genes (Cj1432, Cj1434, Cj1438, Cj1440 and Cj1442). The compression values (listed after the gene name in Table 2) are the same and equal to 1, that is, the number of observable knockout information for each gene. Compression provides a metric of confidence in an assignment (higher compression indicating higher confidence). All the assignments had the same compression value; thus, no discrimination was possible between the corresponding gene product assignments. Despite these unresolved ambiguities, the machine learning still was able to make assignment for ?H and to exclude several functions for genes.

### Learning from gene knockout and strain data

Recently, whole genome comparison studies using DNA microarrays revealed the gene content compared to the NCTC 11168 reference of the capsule locus across a wide variety of *C*. *jejuni* strains^24^ (data available as Supplementary Material). Many of the genes in the CPS loci appear to be conserved between the same Penner serotype, with the exception of the export genes that are highly conserved between all strains. The Penner serotyping procedure is based on the cross-reaction of antiserum to similar CPS structures and is used as the gold standard to distinguish between groups of strains.^20^ We therefore explored the possibility of using strain data to refine our predictions. To use these data, we developed a working assumption:

*if the product of Gene1 synthesises a compound which is subsequently the substrate of Gene2*, *then Gene1 tends to be present in strains with the same serotype as those expressing Gene2*.

The effect of this working assumption is illustrated in Table 3.

Table 2 (column 3) shows the result of learning using both the gene knockout data and the absence or presence of genes from natural strain variants. The final assignment was made by accepting the assignment with the maximum compression; no enzyme is assigned to more than one reaction. The highest compression values (i.e., the highest confidence) are for ?H. Since ?H denotes a series of reaction steps, all three genes were assigned. Assignments then proceeded as shown in Table 2. However, Cj1442 had a compression of 119 when assigned to ?D and 118 when assigned to ?C. Thus, there is an assignment with sub-maximal compression that is only marginally less confident than the optimal one. In this sub-maximal assignment, ?D would be assigned to Cj1440 and ?C to Cj1442. The compression values for the learning with both knockout and strain data are far larger than those from just the knockout learning because the strain data provide a large number of additional observations.

### Estimation of accuracy of gene function identification

**Fig. 6:**
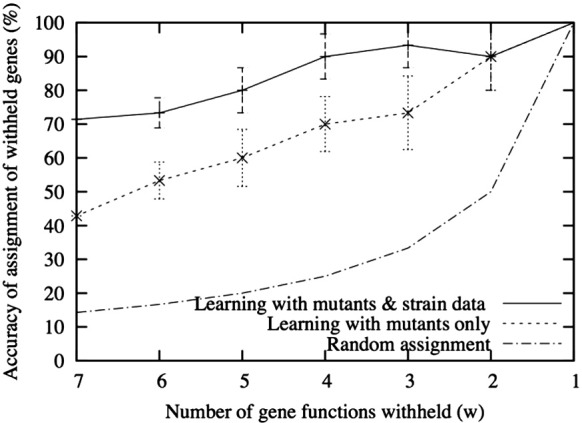
Evaluation of accuracy of machine learning. Performance of knockout-based (broken line) and knockout + strain-based (continuous line) logical models with varying degrees of completeness of the input information (shown on the *x*-axis) relative to the baseline accuracy expected by chance (dot/dash). The spreads are standard error over 10 independent samples.

We studied how well both our knockout-based initial model and our model extended with strain genetic information performed over varying degrees of completeness of the input information (Fig. 6). We considered the seven reactions where the assignment of gene products to reactions was known. For each model, we withheld from the learning framework the assignment of function to a gene product. We varied the number of withheld functions from all seven down to one. The withheld assignments were randomly selected without replacement. Learning was performed and then we evaluated the percentage of all the withheld gene assignments that were correctly assigned. This percentage is referred to as the “identification accuracy”. The spreads for the identification accuracy are the standard error over an ensemble of 10 independent samples. The accuracy expected by chance, if *w* assignments are withheld, is 1/*w*.

Figure 6 shows that the knockout-based model substantially boosts the identification accuracy relative to random. The strain-based extension further boosts the identification accuracy. The results of this study depend on the structure of the network, and different networks with different strain data will yield different improvements from the two models over random. Nevertheless, this study provides evidence that the learning approach is generating hypotheses that are of value to guide subsequent biological experiments.

## Discussion and Conclusion

We examined the hypothesised enzymatic functions of the gene products derived from the *C*. *jejuni* NCTC 11168 CPS locus in terms of supporting biological data. The assignments that Cj1416, Cj1417 and Cj1418 are involved in the series of reactions (denoted ?H) involved in the biosynthesis of the OMePN modification were known prior to our modelling study. These NMR results were incorporated in the observations used for learning, but the assignments of these three genes to ?H were not forced. Thus, the correct assignment of these genes confirms that our modelling approach is valid. The NMR study in Ref. 19 revealed that 70% of *C*. *jejuni* strains tested were shown to express the OMePN modification. A previous study showed that the Cj1416–Cj1418 genes were detected in some of these strains (e.g., strain NCTC 11168 that was used to generate our model, as well as strain 81–176) and that Cj1416–Cj1418 were missing in certain strains without the OMePN side groups.^24^

In the optimal assignment, the transferase of the backbone GlcA (?D) is assigned to Cj1442c while in the best suboptimal to Cj1440. Both alternatives are consistent with the observations that knockout of either Cj1442 or Cj1440 leads to an acapsular phenotype. The optimal assignment of the backbone Gal*f*NAc transferase (?C) is to Cj1440c, while in the suboptimal assignment, it is to Cj1442. Again, either assignment is consistent with the knockout data of an acapsular phenotype.

The transfer of ribose into the CPS backbone (?B) is assigned to Cj1432. This is consistent with the Cj1432 knockout being acapsular, since ribose is also required for capsule biosynthesis. We assign Cj1434 and Cj1438 as steps in ribose synthesis and transfer to CPS (?A), while other genes encoding enzymes for ribose synthesis may be located elsewhere on the chromosome.

The above discussion raises the question of whether the assignments could have been made by manual inspection. A review of the knockout data suggests that the final assignments in Table 2 could not have been made by an expert alone, since mutation of all transferases resulted in an acapsular phenotype. It is also less likely that a prioritised list of gene assignments could have been made without a computational approach when examining the strain data that had experimental error.

There are several novel aspects of the way in which the machine learning was adapted to be used in the study. In the standard setting for ILP, the input consists of two parts: (1) background knowledge and (2) observational examples. While the observational examples are assumed to contain classification errors, the background knowledge has been assumed, to date, to be correct. Here, we introduce a new variant of background knowledge that we refer to as “working assumptions”. The working assumptions (see Learning from gene knock-out and strain data) represent speculative premises that are assumed true for the purposes of generating hypotheses from the examples. The use of these extra working assumptions within the experiments allowed the inclusion of the strain data as a secondary source. The compression achieved on this secondary source was used as a heuristic guide for the hypotheses chosen. Again, this use of secondary observations is novel from a machine learning perspective.

It is our contention that the use of working assumptions and secondary observations follows common practice among human scientists in settings of the kind demonstrated in this paper. Our results also clearly indicate that the approach can lead to increased predictive accuracy by allowing other relevant information involving related biochemistry to be included. Indeed, in a related study,^9^ we have used abduction to infer control points in metabolic networks from transcriptomic and metabolomic data. In conclusion, this paper has shown that abductive ILP can encompass and integrate diverse sources of biological data in a natural and robust approach. This would be more difficult to achieve in many other methodologies currently used for systems biology modelling.

## Materials and Methods

### Construction of the *C*. *jejuni* 11168 mutants

For construction of the Cj1427 and Cj1430 heptose mutants, PCR fragments containing these genes were amplified from a *C. jejuni* 11168 strain lacking the OMePN groups with the following primer pairs. For the Cj1427 mutant, Cj1427cF918 (5′-AACTTTCATCATTTTAAACGCTCTT-3′) and fclR51 (5′-TACAGCATTGGTAGAAAACTTACAA-3′) were used. For the creation of the Cj1430 mutant, primer pairs fclF1023 (5′-CCATTCATACATCATTTTAATACCA-3′) and Cj1431Cr7 (5′-AATTCAAAACCTCTCATAATTGCAG-3′) were used. The PCR products were ligated into pPCR-Script-Amp according to manufacturer's instructions. A blunt-ended kanamycin resistance cassette (KmR) from pILL600 was inserted into the BsaBI restriction site of Cj1427 or the NruI restriction site for Cj1430. The orientation of the KmR cassette was determined by sequencing with the ckanB primer (5′-CCTGGGTTTCAAGCATTAG-3′) using terminator chemistry and AmpliTaq FS cycle polymerase sequencing kits (Applied Biosystems, Carlsbad, CA) and analysed on an Applied Biosystems 373 sequencer. The plasmids were electroporated into *C. jejuni* 11168 to give the strains Cj1427 and Cj1430. The KmR transformants were characterised by PCR to confirm a double-crossover event. The construction of other *C. jejuni* 11168 mutants was undertaken as described previously.^19^ The BamHI fragment containing KmR from pJMK30 was inserted into unique restriction sites within target gene-containing fragments from a 2-kb sequencing library from the *C. jejuni* NCTC 11168 genome project.^16^ KmR was inserted in a nonpolar orientation, and the derivatives were used for transformation of *C. jejuni* cells. The mutants were verified by PCR using KmR and gene-specific primers.

### CE-ESMS of the *C. jejuni* 11168 Cj1427 and Cj1430 cell lysates

Cell lysates from wild type and the isogenic mutants of *C. jejuni* 11168 were prepared for metabolomic analysis as previously described (McNally *et al.*, 2006). These lysates were subsequently probed for intracellular sugar nucleotide intermediates using capillary electrophoresis coupled to electrospray mass spectrometry (CE-ESMS) and precursor ion scanning as previously described.^19,26^

### Glycan structure and gene knockout results

Table 1 and Fig. 2 show the results of the gene knockout experiments based on HR-MAS [^1^H]NMR analyses of the capsule of *C. jejuni* 11168 H and its mutants summarised in Fig. 3. HR-MAS NMR experiments were performed as previously described.^19^ In addition to genes reported in Table 1, McNally *et al.* showed that Cj1415 is also involved in OMePN biosynthesis,^19^ but this mutant was not included in this study because it also shows a second phenotype of reduced CPS production.^19^

For the Cj1426 mutant, HR-MAS NMR demonstrated loss of the 6-*O*-Me. In an earlier study, we isolated a *C. jejuni* 11168 variant that lacked the 6-*O*-Me group and concluded that this modification was phase variable.^22^ In this study, we compared the homopolymeric tract of the Cj1426 gene in this variant and the wild type by PCR amplification using the primers 1426-PCR-F (5′-TTGAGAATTATGATAAGATGAAGG-3′) and 1426-PCR-R (5′-TTTCCTAAGAATTCTTTACTTTCG-3′) and then sequenced using the primers 1426-seq-F (5′-AAGATCCAGATAAAAGAGATTATTTGG-3′) and 1426-seq-R (5′-ATCAGGAGAATCAAAAATGATTTTTCC-3′). The results confirmed that this gene is inactive in the variant (9 Gs) described in our original study^22^ and is active in the wild type (10 Gs), providing further support that Cj1426 is indeed the 6-*O*-Me transferase. We subsequently demonstrated that Cj1426 is phase variable *in* *vivo*, and modification provides *C. jejuni* resistance to phage F336 during co-infection studies in chickens.^27^

To further confirm that Cj1427 and Cj1430 were involved in heptose biosynthesis, we looked for GDP-heptose intermediates using CE-ESMS and precursor ion scanning. Figure 4 demonstrates that GDP-Hep does indeed accumulate in the mutants, but not in the wild type, identifying two additional enzymes involved in heptose biosynthesis in *C. jejuni* 11168 and confirming that this pathway proceeds through GDP-linked intermediates.

### HR-MAS NMR

HR-MAS NMR analysis of intact bacterial cells was performed as previously described.^19^

### Strain data

DNA was extracted from 270 strains and hybridised to the *C. jejuni* 11168 DNA microarray containing reporters for all 1575 genes in the genome as described by Champion *et al.*^28^ Data were processed as either absence (0) of presence (1) of genes. The experiments were performed in triplicate, and from average values, the presence or absence of a gene was determined based on a threshold using the GACK (*G*enomotyping *A*lgorithm by *C*harles *K*im) program^29^ as described in Champion *et al*.^28^ In summary, DNA from each test strain and control strain (11168) was labelled with Cy5 and Cy3, respectively, and then hybridised against the 11168 microarray. Microarrays were scanned using an Affymetrix 418 scanner (MWG Biotech, High Point, NC), and signal data were extracted by using BlueFuse (BlueGnome Ltd., Cambridge, UK). Triplicate spot averaging and data quality control were performed in GeneSpring v7.3.1 (Agilent Technologies, Santa Clara, CA).

The experiments and raw data on 270 strains are in BUGSBASE† and are available from ArrayExpress via the accession number obtainable from BUGSBASE. A subset of these data was used in the strain modelling and is provided in the Excel file “Strain Data”. This contains the Penner serogroup and serotype for a series of strains and the presence (1) and absence (0) of a gene; undetermined results are denoted by ?. Machine learning only used entries with 1 to show the presence of a gene within that strain.

### Machine learning

Machine learning used Progol 5.0.^30^ Prolog clauses express the background knowledge, both known facts and general rules, together with the observations.

The known facts:

1. define the compounds (which are both the metabolites and the glycan structures), for example, *compound('gdp-dd-hep')* and *compound('glca6ngro + hep + omepn + 6ome')* where *gdp-dd-hep* is a metabolite and *glca6ngro + hep + omepn + 6ome* is a glycan structure;
2. define the gene names, for example, *gene('cj1430c')*;
3. define the strains and their serotypes, where known, for example, *strain(strain176_83)*, *penner_serotype(hs41)* and *strain_has_penner_serotype(strain176_83, hs41)*;
4. state that there is a single reaction between two compounds, for example, *reaction(capsule_hep5,'gdp-dd-hep','gdp-d-al-gluco-hep')*, which states that the reaction named *capsule_hep5* catalyses the conversion of *'gdp-dd-hep'* to *'gdp-d-al-gluco-hep'*;
5. state that a gene performs a reaction, for example, *codes(cj1430c, capsule_hep5)*, which states that gene *cj1430c* performs the reaction named *capsule_hep5*; and
6. state that one glycan structure is related to another by the addition of a single chemical group, for example, *struct_next('glca6ngro-galfnac-ribf', 'glca6ngro-galfnac + omepn-ribf')*.

General rules encode:

1. the pathway in terms of steps of sequential reactions, that is,
  *path(R, A, B) if*
  *reaction(R, A, B)*
  *path(R, A, B) if*
  *reaction(R0, A, X) and*
  *path(R, X, B)*
2. the effect of knocking out a gene in terms of the absence of a glycan structure and the implications for assigning a gene to a specific reaction, that is,
  *knockout_observable(Gene, Observable) if*
  *struct_next(Observable, Abs_Struct) and*
  *path(R, Prs_Struct, Abs_Struct) and*
  *codes(Gene, R)*
3. the working assumption that, if a pair of genes is present in two strains that share the same serotype, they both perform neighbour reactions in a path that synthesises a particular glycan, that is,
  *occurs(Strain1,Gene1) if*
  *codes(Gene1, R1) and*
  *neighbour_reaction(R1, R2) and*
  *codes(Gene2, R2) and*
  *occurs(Strain2, Gene2) and*
  *have_same_serotype(Strain1, Strain2)*

The observations:

1. state that when a specific gene is knocked out, this result causes a particular glycan structure to be the largest synthesised, for example, *knockout_observable(cj1431c, 'glca6ngro-galfnac + omepn-ribf')* and
2. state that a particular gene is absent or present from a particular strain, for example, *occurs(strainClinical44811, cj1431c)*.

The Prolog files used are available as supplementary data files (background_pl, codes_pl, common_pl, CPS_pathwayd_pl, glycan_structure_pl, learn_h_pl, learn_H-out, mutant_pl and strains_pl, together with a documentation file README).

### Data sources

The background knowledge was largely compiled from KEGG^31^ and BioCyc^32^ augmented by some specific information about the *C. jejuni* glycan structures that was extracted from publications.^18^ The gene knockout experiments were from previously published data^19^ and included the data presented here. The cross-strain genomic data for the CPS loci originate from an in-progress study in which 270 *C. jejuni* isolates were analysed by comparative phylogenomics (whole genome comparisons of bacteria using DNA microarrays, combined with Bayesian-based algorithms, to model the phylogeny), using a previously published method.^28^

## Figures and Tables

**Table 1:** CPS glycan phenotypes for single-knockout *C. jejuni* mutants assigned by HR-MAS NMR

Gene knockout	Phenotype of CPS	Functional annotation	Reference
*Specific gene function modelled as unknown*			
Cj1416	P1-loss of OMePN	OMePN nucleotidyltransferase	McNally *et al.*^19^
Cj1417	P1-loss of OMePN	OMePN biosynthesis	McNally *et al.*^19^
Cj1418	P1-loss of OMePN	OMePN biosynthesis	McNally *et al.*^19^
Cj1432	P6-loss of CPS	Sugar transferase	This study
Cj1434	P6-loss of CPS	Sugar transferase	This study
Cj1438	P6-loss of CPS	Sugar transferase	This study
Cj1440	P6-loss of CPS	Sugar transferase	This study
Cj1442	P6-loss of CPS	Sugar transferase	This study

*Specific gene function modelled as known*			
Cj1421	P2-loss of OMePN on Gal*f*NAc	NDP-OMePN transferase	McNally *et al.*^19^
Cj1422	P3-loss of OMePN on Hep	NDP-OMePN transferase	McNally *et al.*^19^
Cj1423	P4-loss of Hep	Heptose guanosyltransferase	Karlyshev *et al.*^20^
Cj1424	P4-loss of Hep	Sedoheptulose isomerase	Karlyshev *et al.*^20^
Cj1425	P4-loss of Hep	Heptose kinase	Karlyshev *et al.*^20^
Cj1426	P5-loss of 6-OMe on Hep	Methyltransferase	This study
Cj1427	P4-loss of Hep	GDP-heptose epimerase	This study
Cj1428	P4-loss of Hep	GDP-heptose epimerase	St Michael *et al.*^18^
Cj1430	P4-loss of Hep	GDP-heptose epimerase	This study
Cj1431	P4-loss of Hep	GDP-heptosyltransferase	Karlyshev *et al.*^20^
Cj1439	P6-loss of CPS	UDP-Gal/GalNac pyranose mutase	St Michael *et al.*^18^
			Poulin *et al.*^21^
Cj1441	P6-loss of CPS	UDP-GlcA dehydrogenase	St Michael *et al.*^18^ and this study

The wild type and knockout mutant glycan phenotypes (P1 to P6) for the CPS repeat unit are described as well as being shown schematically in Fig. 2. Functional assignments were made for the genes involved in capsule biosynthesis on the basis of the publications listed.

**Table 2:** The five transformations for which gene assignments were made (see Fig. 3)

Enzyme reaction (names as in Fig. 3)	Learnt from knockout data	Learnt from knockout and strain data	Final assignment based on maximally compressive selection
?H (phosphoramidate synthesis chain)	Cj1416 (1)	Cj1416 (1 + 130)	Cj1416 (131)
	Cj1417 (1)	Cj1417 (1 + 127)	Cj1417 (128)
	Cj1418 (1)	Cj1418 (1 + 127)	Cj1418 (128)
?D (glucuronic acid transferase)	Cj1442 (1)	Cj1442 (1 + 118)	Cj1442 (119)
	Cj1440 (1)	Cj1440 (1 + 104)	
	Cj1432 (1)	Cj1432 (1 + 103)	
	Cj1438 (1)	Cj1438 (1 + 100)	
	Cj1434 (1)	Cj1434 (1 + 99)	
?C (*N*-acetyl galactofuranosamine transferase)	Cj1442 (1)	Cj1442 (1 + 117)	Cj1440 (118)
	Cj1440 (1)	Cj1440 (1 + 103)	
	Cj1432 (1)	Cj1432 (1 + 102)	
	Cj1438 (1)	Cj1438 (1 + 101)	
	Cj1434 (1)	Cj1434 (1 + 100)	
?B (ribosyltransferase)	Cj1442 (1)	Cj1442 (1 + 116)	Cj1432 (117)
	Cj1440 (1)	Cj1440 (1 + 103)	
	Cj1432 (1)	Cj1432 (1 + 102)	
	Cj1438 (1)	Cj1438 (1 + 100)	
	Cj1434 (1)	Cj1434 (1 + 99)	
?A (ribose precursor synthesis chain)	Cj1432 (1)	Cj1432 (1 + 87)	Cj1434 (88)
	Cj1434 (1)	Cj1434 (1 + 87)	Cj1438 (88)
	Cj1438 (1)	Cj1438 (1 + 87)	
	Cj1440 (1)	Cj1440 (1 + 87)	
	Cj1442 (1)	Cj1442 (1 + 87)	

Column 2 gives the genes assigned using just knockout data with the number in parentheses being the compression. Column 3 gives the assignment after learning from both knockout and strain data. The compression is given as the compression from the knockout data followed by the compression from the strain data. The higher the compression, the greater confidence there is in the assignment. Column 4 gives the final assignment based on the progressive selection of genes with maximal compression from column 3. The number in parentheses is the compression. The table is ordered based on decreasing compression values in the final assignment. The transformations ?H and ?A each constitute a series of individual reactions, and consequently, several genes are assigned.

**Table 3:** An illustration of the working assumption that allows us to make inferences about gene function from the relationship of gene occurrences in different strains with the same serotype

Gene	Present in strain 1	Present in strain 2	Supports rule
Cj1432	Yes	Yes	Yes
Cj1433	Yes	No	No
Cj1434	No	Yes	No
Cj1435	No	No	No

## Appendix A Supplementary Data

Sup v1.pdf

Strain Data.cvs

## Footnotes

† http://bugs.sgul.ac.uk/bugsbase/
