# Supplementary material for: Gene Function Hypotheses for the Campylobacter jejuni Glycome Generated by a Logic-Based Approach
Source: J Mol Biol. 2013 Jan 9;425(1):186–97. doi: 10.1016/j.jmb.2012.10.014 (PMC3546167; doi:10.1016/j.jmb.2012.10.014)
Supplement: Supplementary file 1 — Sup v1.pdf [file mmc1.pdf]

This README defines the Prolog data files used in the following paper:

Michael Sternberg, et. al. Gene function hypotheses for the Campylobacter jejuni glycome generated by a logic-based approach.

DATA

The data used for learning are arranged in the following files.
(Note genes have the suffic "c" after their number which was, for brevity, ommitted in the paper).

background_pl
    The background knowledge for the machine learning which includes the general rules, working assumptions and the abductive learning settings for Progol 5.0.

codes_pl
    Known gene functions represented by instances of the 'codes' predicate

common_pl
    Common predicate declarations for genes and strains

CPS_pathway_pl
    The C. jejuni CPS pathway information compiled from KEGG and BioCyc augmented by some specific information about the C. jejuni capsul glycan structure that were extracted from publications.

glycan_structure_pl
    The C. jejuni CPS glycan structures. This includes the definition of struct_next which states that one glycan structure is related to another by the addition of a single chemical group

mutants_pl
    The gene knock-out experiments for C. jejuni CPS. The data is mainly compiled from previously published data. The data is represented by instances of the 'knockout_observable' predicate which states when a specific gene is knocked out this result causes a particular glycan structure to be the largest synthesised

strains_pl
    The cross-strain genomic data for the CPS loci originate from an in-progress study in which 270 C. jejuni isolates were analysed by comparative phylogenomics (whole genome comparisons of bacteria using DNA microarrays, combined with Bayesian-based algorithms, to model the phylogeny), using a previously published method. The data includes predicates 'absent' and 'occurs' which state that a particular gene is absent or present from a particular strain.

MACHINE LEARNING USING PROGOL

These files were prepared to be used with the Inductive Logic Programming (ILP) system Progol 5.0. The version of Progol used in this study can be downloaded from

http://www.doc.ic.ac.uk/Campy

The latest version of Progol is available from:
http://www.doc.ic.ac.uk/~shm/Software/progol5.0/

It is necessary to abduce the function of each gene in turn to obtain the independent compression values reported in the paper. As an example below we give the input to adbuce the function of cj1416c and the ouput obtained.

learn_cj1416c_pl
    Example learning file for the reaction cj1416c in the paper. This is an input file for Progol 5.0 which loads other files in this directory into Progol and does the learning.

learn_cj1416c_out
    The output of running Progol 5.0 on the example file learn_cj1416c.pl

After compiling the source file, Progol 5.0 can be used to run the example learning file described above. Under UNIX, to run Progol, rename each file as .pl so for example, codes_pl.txt should be renamed as codes.pl

To intitiate the learning, type the following at the command line:

```
$ progol learn_cj1416c.pl
```

Then you should obtain the same output as the one in learn_cj1416c_out.

VISUALISATION

We developed a portal-based workbench, WIBL (Workbench for Integrative Biological Learning) to browse the glycan structure, metabolic and protein-protein interaction networks as well as the underlying Prolog. WIBL is hosted at
http://www.sbg.bio.ic.ac.uk/wibl (username guest, project campy).
Lesk V, Taubert J, Rawlings C, Dunbar S, Muggleton S (2011)
WIBL: Workbench for Integrative Biological Learning.
Journal of integrative bioinformatics 8: 156

```
%  FILE BACKGROUND_PL
%%%%%%%%%%%%%%%%%%%%%%%%%%%%%%%%%%%%%%%%%%%%%%%%%%%%%%%
% A logic program for modelling genotype-phenotype
%   relation in Campylobacter jejuni. This model
%   integrates data from different sources, i.e. mutants
%   data from gene knock-out experiments, cross-strain
%   genomic data, glycan structures and pathways
%   information compiled from KEGG and BioCyc
%
% N.B. This model was prepared to be used with Progol 5.0


% Abductive learning settings for  Progol 5.0

:- modeh(1,codes(#any,#any))?

:- observable(knockout_observable/2)?

% The following rule models the effect of knocking-out a gene in terms
% of the absence of a glycan structure and the implications for assigning
% a gene to a specific reaction

knockout_observable(Gene,Observable):-
        unassigned_gene(Gene),
        struct_observable(Prs_Struct,Observable),
        struct_next(Prs_Struct,Abs_Struct),
        path3(R,Prs_Struct,Abs_Struct),
        unassigned_reaction(R),
        codes(Gene,R).

% The working assumption that if a pair of genes are present in two strains
% which share the same serotype, they both perform neighbour reactions in a
% path which synthesises a particular glycan

occurs(Strain1,Gene):-
        codes(Gene,R1),
        neighbour_reaction(R1,R2),
        codes(G2,R2),
        occurs0(Strain2,G2),
        have_same_serotype(Strain1,Strain2),!.

have_same_serotype(Strain1,Strain2):-
        Strain1 \= Strain2,
        strain_has_penner_serotype(Strain1,Serotype),
        strain_has_penner_serotype(Strain2,Serotype),!.



%%%%%%%%%%%%%%%%%%%%%%%%%%%%%%%%%%%%%%%%%%%%%%%%%%%%%%%%%
%%%%%
% The definition of pathways in terms of steps of sequential reactions

path3(R,X,Y):-
```

```
    reaction3(R,X,Y).

path3(R,X,Y):-
    reaction3(R0,X,Y),
    reaction3(R0,Z,Y),
    not(X=Z),
    leading_to(R,Z).

leading_to(R,X):-
    reaction3(R,Z,X).
leading_to(R,X):-
    reaction3(_,Z,X),
    leading_to(R,Z).

reaction3(R,X,Y):-
    reaction2(R,X,Y).

reaction2(R,X,Y):-
    reaction(D,O,R,X,Y,Dir).


%%%%%%%%%%%%%%%%%%%%%%%%%%%%%%%%%%%%%%%%%%%%%%%%%%%%%%%%%%%%
%%%%%%%%%%%
% Integrity constraints

:- codes(Gene, Reaction1),
   codes(Gene, Reaction2),
   not(Reaction1 = Reaction2).

:- codes(Gene1, Reaction),
   codes(Gene2, Reaction),
   not(chain(Reaction)),
   not(Gene1 = Gene2).
```

```
%   FILE CODES_PL
% Known gene functions represented by instances of the 'codes' predicate

codes(cj1421c,capsule_ngro36a).
codes(cj1422c,capsule_ngro21a).
codes(cj1426c,capsule_ngro21c).
codes(cj1430c,capsule_hep5).
codes(cj1431c,capsule_ngro1c).
codes(cj1439c,capsule_pre_pre_galfnac).
codes(cj1441c,capsule_pre_pre_glca).
```

```
%   FILE COMMON_PL
% Common predicate declarations for genes and strains

gene(cj1413c).
gene(cj1414c).
gene(cj1415c).
gene(cj1416c).
gene(cj1417c).
gene(cj1418c).
gene(cj1419c).
gene(cj1420c).
gene(cj1421c).
gene(cj1422c).
gene(cj1423c).
gene(cj1424c).
gene(cj1425c).
gene(cj1426c).
gene(cj1427c).
gene(cj1428c).
gene(cj1429c).
gene(cj1430c).
gene(cj1431c).
gene(cj1432c).
gene(cj1433c).
gene(cj1434c).
gene(cj1435c).
gene(cj1436c).
gene(cj1437c).
gene(cj1438c).
gene(cj1439c).
gene(cj1440c).
gene(cj1441c).
gene(cj1442c).
gene(cj1443c).
gene(cj1444c).
gene(cj1445c).
gene(cj1447c).
gene(cj1448c).

strain(strain176_83).
strain(strain81116).
strain(strain81_176).
strain(strainBeach1771).
strain(strainBeach1772).
strain(strainBeach1773).
strain(strainBeach1791).
strain(strainBeach1792).
strain(strainBeach1793).
strain(strainBeach28766X).
strain(strainBeach28770).
strain(strainBovine27).
strain(strainBovine37).
strain(strainBovineC115).
```

strain(strainBovineC119).
strain(strainBovineC123).
strain(strainBovineC156).
strain(strainBovineC180).
strain(strainBovineC30).
strain(strainBovinevineine13305).
strain(strainCalf2).
strain(strainCalf3).
strain(strainCalf5).
strain(strainCamel0014).
strain(strainCamel0029).
strain(strainCamel0032).
strain(strainCamel0037).
strain(strainCamel0049).
strain(strainCamel0062).
strain(strainCamel0064).
strain(strainCamel0079).
strain(strainCamel0086).
strain(strainCattel11).
strain(strainCattle10).
strain(strainCattle8_O).
strain(strainCG8486).
strain(strainChicken11848).
strain(strainChicken11919).
strain(strainChicken11973).
strain(strainChicken12196).
strain(strainChicken12567).
strain(strainChicken13713).
strain(strainChicken40209).
strain(strainChicken40267).
strain(strainChicken47693).
strain(strainChicken59214).
strain(strainChicken91B1).
strain(strainChicken9B39).
strain(strainChickenicken11818).
strain(strainChickenicken11848).
strain(strainChickenicken11856).
strain(strainChickenicken11919).
strain(strainChickenicken11973).
strain(strainChickenicken11974).
strain(strainChickenicken12450).
strain(strainChickenicken12487).
strain(strainChickenicken12567).
strain(strainChickenicken12912).
strain(strainChickenicken13040).
strain(strainChickenicken13082).
strain(strainChickenicken13249).
strain(strainChickenicken13411).
strain(strainChickenicken13713).
strain(strainCjejuni11168).
strain(strainCjejuniRM1221).
strain(strainClinical18836).
strain(strainClinical30280).
strain(strainClinical30328).

strain(strainClinical31481).
strain(strainClinical31485).
strain(strainClinical32787).
strain(strainClinical32799).
strain(strainClinical33084).
strain(strainClinical33106).
strain(strainClinical34007).
strain(strainClinical35424).
strain(strainClinical35535).
strain(strainClinical35799).
strain(strainClinical36069).
strain(strainClinical36439).
strain(strainClinical36860).
strain(strainClinical36952).
strain(strainClinical37537).
strain(strainClinical38553).
strain(strainClinical38762).
strain(strainClinical38857).
strain(strainClinical39828).
strain(strainClinical40671).
strain(strainClinical40917).
strain(strainClinical41651).
strain(strainClinical42724).
strain(strainClinical43205).
strain(strainClinical43983).
strain(strainClinical44119).
strain(strainClinical44811).
strain(strainClinical44933).
strain(strainClinical44958).
strain(strainClinical45557).
strain(strainClinical45631).
strain(strainClinical47886).
strain(strainClinical47939).
strain(strainClinical48612).
strain(strainClinical52331).
strain(strainClinical52368).
strain(strainClinical52471).
strain(strainClinical53250).
strain(strainClinical55320).
strain(strainClinical55703).
strain(strainClinical56281).
strain(strainClinical56282).
strain(strainClinical56519).
strain(strainClinical56832).
strain(strainClinical58473).
strain(strainClinical59364).
strain(strainClinical59424).
strain(strainClinical62914).
strain(strainClinical63326).
strain(strainClinical64555).
strain(strainClinical81116).
strain(strainClinicalF1).
strain(strainClinicalF2).
strain(strainClinicalF3).

strain(strainClinicalG1).
strain(strainClinicalG2).
strain(strainClinicalG3).
strain(strainClinicalG4).
strain(strainClinicalinical15168).
strain(strainClinicall31467).
strain(strainClinicall38556).
strain(strainClinicall39182).
strain(strainClinicalM1).
strain(strainG1).
strain(strainGoose126).
strain(strainGoose222).
strain(strainGoose66).
strain(strainGoose86).
strain(strainHi40500471).
strain(strainHi40520410).
strain(strainHi40540310).
strain(strainHi40540311).
strain(strainHi40620293).
strain(strainHi40620296).
strain(strainHi40620300).
strain(strainHi40680224).
strain(strainHi40800231).
strain(strainHi40840318).
strain(strainHi40840324).
strain(strainHi40900388).
strain(strainHi40980306).
strain(strainHi41000246).
strain(strainHi41020285).
strain(strainHi41040340).
strain(strainHi41080433).
strain(strainHi41080434).
strain(strainHi41100305).
strain(strainHi41120166).
strain(strainHi41120167).
strain(strainHi41160244).
strain(strainHi41220381).
strain(strainHi41220382).
strain(strainHi41300251).
strain(strainHi41300252).
strain(strainHi41300259).
strain(strainHi41360174).
strain(strainHi41380304).
strain(strainHi41500294).
strain(strainHi41720262).
strain(strainHi41760156).
strain(strainHi41780227).
strain(strainHi41780229).
strain(strainHi41800342).
strain(strainHi41840334).
strain(strainHi41840336).
strain(strainHi41880318).
strain(strainHi42080176).
strain(strainHi43240534).

strain(strainHi43240536).
strain(strainHi43320472rept).
strain(strainHi43320475).
strain(strainHi43340233).
strain(strainHi43380463).
strain(strainHi43400514).
strain(strainHi43480582).
strain(strainHi43500234).
strain(strainHi43580563).
strain(strainHi43700324).
strain(strainHi50520408).
strain(strainHi80512).
strain(strainHi80530).
strain(strainHi80531).
strain(strainHi80536).
strain(strainHi80547).
strain(strainHi80552).
strain(strainHi80554).
strain(strainHi80563).
strain(strainHi80583).
strain(strainHi80584).
strain(strainHi80586).
strain(strainHi80614).
strain(strainHi80646).
strain(strainHi80650).
strain(strainHi80659).
strain(strainHi80675).
strain(strainHi80809).
strain(strainHi80838).
strain(strainHi80841).
strain(strainHi80864).
strain(strainHi80866).
strain(strainHi80876).
strain(strainHi80884).
strain(strainHi80885).
strain(strainHi80889).
strain(strainHi80896).
strain(strainHi80901).
strain(strainHi80908).
strain(strainHi80935).
strain(strainHi80938).
strain(strainHi80939).
strain(strainHi80983).
strain(strainHi80986).
strain(strainHi81003).
strain(strainHi81005).
strain(strainHi81006).
strain(strainHi81006rept).
strain(strainHi81018).
strain(strainHi81035).
strain(strainHi81036).
strain(strainHi81040).
strain(strainHi81049).
strain(strainHi81061).

strain(strainHi81062rep).
strain(strainHi81109).
strain(strainHi81121).
strain(strainHi81132).
strain(strainHi81135).
strain(strainHi81139).
strain(strainHi81143).
strain(strainHi81180).
strain(strainHi81181).
strain(strainHi81205).
strain(strainHi81206).
strain(strainHi81214).
strain(strainHi81239).
strain(strainHi81266).
strain(strainHi81268).
strain(strainHi81270).
strain(strainHi81276).
strain(strainHi81289).
strain(strainHi81290).
strain(strainHi81292).
strain(strainHi81311).
strain(strainHi81335).
strain(strainHi81338).
strain(strainHi81342).
strain(strainHi81357).
strain(strainHi81363).
strain(strainHi81371).
strain(strainLamb11).
strain(strainMilk1398).
strain(strainMilk1403).
strain(strainMilk1899_BA).
strain(strainMilk1899_BC).
strain(strainNCTC12517).
strain(strainOvine12241).
strain(strainOvine12481).
strain(strainOvineC55).
strain(strainOvineSM4).
strain(strainOvineSM6).
strain(strainOvineSM8).
strain(strainRM1221).
strain(strainSheep11).
strain(strainSheep18).
strain(strainSheep2).
strain(strainSheep8).
strain(strainSheepSM7).
strain(strainSheepSM9).
strain(strainStarling86857).
strain(strainTurkey4).
strain(strainTurkey8).
strain(strainWaterS5).
strain(strainWaterSA5).

penner_serogroup(serogroupA).
penner_serogroup(serogroupB).

```
penner_serogroup(serogroupD).
penner_serogroup(serogroupE).
penner_serogroup(serogroupF).
penner_serogroup(serogroupK).
penner_serogroup(serogroupN).
penner_serogroup(serogroupO).
penner_serogroup(serogroupP).
penner_serogroup(serogroupR).
penner_serogroup(serogroupS).
penner_serogroup(serogroupU).
penner_serogroup(serogroupY).
penner_serogroup(serogroupZ2).
penner_serogroup(serogroupZ6).

penner_serotype(hs1).
penner_serotype(hs12).
penner_serotype(hs13).
penner_serotype(hs16).
penner_serotype(hs18).
penner_serotype(hs19).
penner_serotype(hs2).
penner_serotype(hs21).
penner_serotype(hs23).
penner_serotype(hs2336).
penner_serotype(hs27).
penner_serotype(hs31).
penner_serotype(hs35).
penner_serotype(hs37).
penner_serotype(hs4).
penner_serotype(hs41).
penner_serotype(hs42).
penner_serotype(hs44).
penner_serotype(hs5).
penner_serotype(hs50).
penner_serotype(hs53).
penner_serotype(hs55).
penner_serotype(hs6).
penner_serotype(hs60).
penner_serotype(hs67).

clade(cladeA1).
clade(cladeA3).
clade(cladeB1).
clade(cladeB2).
clade(cladeB3).
clade(cladeB4).

superclade(supercladeA).
superclade(supercladeB).

strain_has_penner_serotype(strain176_83,hs41).
strain_has_penner_serotype(strain81116,hs6).
strain_has_penner_serotype(strain81_176,hs2336).
strain_has_penner_serotype(strainBeach1771,hs55).
```

strain_has_penner_serotype(strainBeach1791,hs2).
strain_has_penner_serotype(strainBeach1793,hs5).
strain_has_penner_serotype(strainBovinevineine13305,hs50).
strain_has_penner_serotype(strainCG8486,hs4).
strain_has_penner_serotype(strainChicken11848,hs2).
strain_has_penner_serotype(strainChicken11919,hs2).
strain_has_penner_serotype(strainChicken11973,hs2).
strain_has_penner_serotype(strainChicken12196,hs50).
strain_has_penner_serotype(strainChicken12567,hs2).
strain_has_penner_serotype(strainChicken13713,hs2).
strain_has_penner_serotype(strainChicken40209,hs5).
strain_has_penner_serotype(strainChicken47693,hs27).
strain_has_penner_serotype(strainChickenicken11818,hs50).
strain_has_penner_serotype(strainChickenicken11856,hs50).
strain_has_penner_serotype(strainChickenicken11974,hs44).
strain_has_penner_serotype(strainChickenicken12450,hs50).
strain_has_penner_serotype(strainChickenicken12487,hs50).
strain_has_penner_serotype(strainChickenicken12912,hs50).
strain_has_penner_serotype(strainChickenicken13040,hs50).
strain_has_penner_serotype(strainChickenicken13082,hs50).
strain_has_penner_serotype(strainChickenicken13249,hs44).
strain_has_penner_serotype(strainChickenicken13411,hs44).
strain_has_penner_serotype(strainCjejuniRM1221,hs53).
strain_has_penner_serotype(strainClinical18836,hs19).
strain_has_penner_serotype(strainClinical30280,hs16).
strain_has_penner_serotype(strainClinical30328,hs16).
strain_has_penner_serotype(strainClinical31481,hs37).
strain_has_penner_serotype(strainClinical32787,hs18).
strain_has_penner_serotype(strainClinical32799,hs50).
strain_has_penner_serotype(strainClinical33084,hs35).
strain_has_penner_serotype(strainClinical33106,hs4).
strain_has_penner_serotype(strainClinical34007,hs18).
strain_has_penner_serotype(strainClinical36069,hs5).
strain_has_penner_serotype(strainClinical36439,hs12).
strain_has_penner_serotype(strainClinical36860,hs21).
strain_has_penner_serotype(strainClinical38553,hs5).
strain_has_penner_serotype(strainClinical38762,hs18).
strain_has_penner_serotype(strainClinical38857,hs23).
strain_has_penner_serotype(strainClinical39828,hs42).
strain_has_penner_serotype(strainClinical40671,hs50).
strain_has_penner_serotype(strainClinical40917,hs21).
strain_has_penner_serotype(strainClinical41651,hs16).
strain_has_penner_serotype(strainClinical43205,hs2).
strain_has_penner_serotype(strainClinical43983,hs50).
strain_has_penner_serotype(strainClinical44119,hs18).
strain_has_penner_serotype(strainClinical44811,hs2).
strain_has_penner_serotype(strainClinical44933,hs13).
strain_has_penner_serotype(strainClinical44958,hs50).
strain_has_penner_serotype(strainClinical45557,hs60).
strain_has_penner_serotype(strainClinical45631,hs13).
strain_has_penner_serotype(strainClinical47939,hs67).
strain_has_penner_serotype(strainClinical48612,hs2).
strain_has_penner_serotype(strainClinical52331,hs50).
strain_has_penner_serotype(strainClinical53250,hs60).

strain_has_penner_serotype(strainClinical55320,hs13).
strain_has_penner_serotype(strainClinical55703,hs13).
strain_has_penner_serotype(strainClinical56281,hs50).
strain_has_penner_serotype(strainClinical56282,hs50).
strain_has_penner_serotype(strainClinical56519,hs12).
strain_has_penner_serotype(strainClinical56832,hs50).
strain_has_penner_serotype(strainClinical58473,hs2).
strain_has_penner_serotype(strainClinical59364,hs31).
strain_has_penner_serotype(strainClinical59424,hs31).
strain_has_penner_serotype(strainClinical63326,hs31).
strain_has_penner_serotype(strainClinical64555,hs31).
strain_has_penner_serotype(strainClinical81116,hs6).
strain_has_penner_serotype(strainClinicalG1,hs1).
strain_has_penner_serotype(strainClinicalG3,hs1).
strain_has_penner_serotype(strainClinicalinical15168,hs19).
strain_has_penner_serotype(strainClinicall31467,hs18).
strain_has_penner_serotype(strainClinicall38556,hs13).
strain_has_penner_serotype(strainClinicall39182,hs13).
strain_has_penner_serotype(strainClinicalM1,hs21).
strain_has_penner_serotype(strainG1,hs1).
strain_has_penner_serotype(strainNCTC12517,hs19).
strain_has_penner_serotype(strainOvine12241,hs50).
strain_has_penner_serotype(strainOvine12481,hs50).
strain_has_penner_serotype(strainRM1221,hs53).

strain_has_penner_serogroup(strain176_83,serogroupZ2).
strain_has_penner_serogroup(strain81116,serogroupF).
strain_has_penner_serogroup(strain81_176,serogroupR).
strain_has_penner_serogroup(strainBeach1771,serogroupZ6).
strain_has_penner_serogroup(strainBeach1791,serogroupB).
strain_has_penner_serogroup(strainBeach1793,serogroupE).
strain_has_penner_serogroup(strainBovinevineine13305,serogroupD).
strain_has_penner_serogroup(strainCG8486,serogroupD).
strain_has_penner_serogroup(strainChicken11848,serogroupB).
strain_has_penner_serogroup(strainChicken11919,serogroupB).
strain_has_penner_serogroup(strainChicken11973,serogroupB).
strain_has_penner_serogroup(strainChicken12196,serogroupD).
strain_has_penner_serogroup(strainChicken12567,serogroupB).
strain_has_penner_serogroup(strainChicken13713,serogroupB).
strain_has_penner_serogroup(strainChicken40209,serogroupE).
strain_has_penner_serogroup(strainChicken47693,serogroupS).
strain_has_penner_serogroup(strainChickenicken11818,serogroupD).
strain_has_penner_serogroup(strainChickenicken11856,serogroupD).
strain_has_penner_serogroup(strainChickenicken11974,serogroupA).
strain_has_penner_serogroup(strainChickenicken12450,serogroupD).
strain_has_penner_serogroup(strainChickenicken12487,serogroupD).
strain_has_penner_serogroup(strainChickenicken12912,serogroupD).
strain_has_penner_serogroup(strainChickenicken13040,serogroupD).
strain_has_penner_serogroup(strainChickenicken13082,serogroupD).
strain_has_penner_serogroup(strainChickenicken13249,serogroupA).
strain_has_penner_serogroup(strainChickenicken13411,serogroupA).
strain_has_penner_serogroup(strainCjejuniRM1221,serogroupR).
strain_has_penner_serogroup(strainClinical18836,serogroupO).
strain_has_penner_serogroup(strainClinical30280,serogroupD).

strain_has_penner_serogroup(strainClinical30328,serogroupD).
strain_has_penner_serogroup(strainClinical31481,serogroupY).
strain_has_penner_serogroup(strainClinical32787,serogroupN).
strain_has_penner_serogroup(strainClinical32799,serogroupD).
strain_has_penner_serogroup(strainClinical33106,serogroupD).
strain_has_penner_serogroup(strainClinical34007,serogroupN).
strain_has_penner_serogroup(strainClinical36069,serogroupE).
strain_has_penner_serogroup(strainClinical36439,serogroupK).
strain_has_penner_serogroup(strainClinical36860,serogroupP).
strain_has_penner_serogroup(strainClinical38553,serogroupE).
strain_has_penner_serogroup(strainClinical38762,serogroupN).
strain_has_penner_serogroup(strainClinical38857,serogroupR).
strain_has_penner_serogroup(strainClinical40671,serogroupD).
strain_has_penner_serogroup(strainClinical40917,serogroupP).
strain_has_penner_serogroup(strainClinical41651,serogroupD).
strain_has_penner_serogroup(strainClinical43205,serogroupB).
strain_has_penner_serogroup(strainClinical43983,serogroupD).
strain_has_penner_serogroup(strainClinical44119,serogroupN).
strain_has_penner_serogroup(strainClinical44811,serogroupB).
strain_has_penner_serogroup(strainClinical44933,serogroupD).
strain_has_penner_serogroup(strainClinical44958,serogroupD).
strain_has_penner_serogroup(strainClinical45631,serogroupD).
strain_has_penner_serogroup(strainClinical48612,serogroupB).
strain_has_penner_serogroup(strainClinical52331,serogroupD).
strain_has_penner_serogroup(strainClinical55320,serogroupD).
strain_has_penner_serogroup(strainClinical55703,serogroupD).
strain_has_penner_serogroup(strainClinical56281,serogroupD).
strain_has_penner_serogroup(strainClinical56282,serogroupD).
strain_has_penner_serogroup(strainClinical56519,serogroupK).
strain_has_penner_serogroup(strainClinical56832,serogroupD).
strain_has_penner_serogroup(strainClinical58473,serogroupB).
strain_has_penner_serogroup(strainClinical59364,serogroupU).
strain_has_penner_serogroup(strainClinical59424,serogroupU).
strain_has_penner_serogroup(strainClinical63326,serogroupU).
strain_has_penner_serogroup(strainClinical64555,serogroupU).
strain_has_penner_serogroup(strainClinical81116,serogroupF).
strain_has_penner_serogroup(strainClinicalG1,serogroupA).
strain_has_penner_serogroup(strainClinicalG3,serogroupA).
strain_has_penner_serogroup(strainClinicalinical15168,serogroupO).
strain_has_penner_serogroup(strainClinicall31467,serogroupN).
strain_has_penner_serogroup(strainClinicall38556,serogroupD).
strain_has_penner_serogroup(strainClinicall39182,serogroupD).
strain_has_penner_serogroup(strainClinicalM1,serogroupP).
strain_has_penner_serogroup(strainG1,serogroupA).
strain_has_penner_serogroup(strainNCTC12517,serogroupO).
strain_has_penner_serogroup(strainOvine12241,serogroupD).
strain_has_penner_serogroup(strainOvine12481,serogroupD).
strain_has_penner_serogroup(strainRM1221,serogroupR).

strain_has_clade(strainBeach1771,cladeB4).
strain_has_clade(strainBeach1772,cladeB4).
strain_has_clade(strainBeach1773,cladeB4).
strain_has_clade(strainBovinevineine13305,cladeB4).
strain_has_clade(strainCattle10,cladeB4).

```
strain_has_clade(strainChicken47693,cladeB4).
strain_has_clade(strainClinical35799,cladeB4).
strain_has_clade(strainClinical36439,cladeB4).
strain_has_clade(strainClinical38553,cladeB1).
strain_has_clade(strainClinical40671,cladeB4).
strain_has_clade(strainClinical43205,cladeB3).
strain_has_clade(strainClinical43983,cladeB2).
strain_has_clade(strainClinical53250,cladeB4).
strain_has_clade(strainClinical55320,cladeB2).
strain_has_clade(strainClinical56281,cladeA1).
strain_has_clade(strainClinical56519,cladeB4).
strain_has_clade(strainGoose126,cladeB4).
strain_has_clade(strainGoose222,cladeB4).
strain_has_clade(strainHi40540310,cladeA3).
strain_has_clade(strainHi40680224,cladeB1).
strain_has_clade(strainHi40900388,cladeB1).
strain_has_clade(strainHi41040340,cladeB3).
strain_has_clade(strainHi41100305,cladeB3).
strain_has_clade(strainHi41120166,cladeA3).
strain_has_clade(strainHi41220381,cladeA3).
strain_has_clade(strainHi41220382,cladeA3).
strain_has_clade(strainHi41500294,cladeB4).
strain_has_clade(strainHi43400514,cladeB4).
strain_has_clade(strainHi43580563,cladeB3).
strain_has_clade(strainHi43700324,cladeB4).
strain_has_clade(strainHi50520408,cladeB4).
strain_has_clade(strainHi80512,cladeB1).
strain_has_clade(strainHi80659,cladeB1).
strain_has_clade(strainHi80675,cladeB1).
strain_has_clade(strainHi80841,cladeB3).
strain_has_clade(strainHi80866,cladeB1).
strain_has_clade(strainHi80884,cladeB3).
strain_has_clade(strainHi80908,cladeB4).
strain_has_clade(strainHi81005,cladeB4).
strain_has_clade(strainHi81036,cladeB3).
strain_has_clade(strainHi81049,cladeB4).
strain_has_clade(strainHi81061,cladeB4).
strain_has_clade(strainHi81143,cladeB1).
strain_has_clade(strainHi81181,cladeB4).
strain_has_clade(strainHi81239,cladeB1).
strain_has_clade(strainHi81311,cladeB3).
strain_has_clade(strainHi81338,cladeB1).
strain_has_clade(strainHi81371,cladeB1).
strain_has_clade(strainOvine12241,cladeB4).
strain_has_clade(strainOvine12481,cladeB4).
strain_has_clade(strainSheepSM7,cladeB4).
strain_has_clade(strainStarling86857,cladeB4).
strain_has_clade(strainWaterSA5,cladeB4).

strain_has_superclade(strainBeach1771,supercladeB).
strain_has_superclade(strainBeach1772,supercladeB).
strain_has_superclade(strainBeach1773,supercladeB).
strain_has_superclade(strainBeach1791,supercladeA).
strain_has_superclade(strainBeach1792,supercladeB).
```

```
strain_has_superclade(strainBeach1793,supercladeB).
strain_has_superclade(strainBeach28766X,supercladeA).
strain_has_superclade(strainBeach28770,supercladeA).
strain_has_superclade(strainBovine27,supercladeA).
strain_has_superclade(strainBovine37,supercladeA).
strain_has_superclade(strainBovineC115,supercladeA).
strain_has_superclade(strainBovineC119,supercladeA).
strain_has_superclade(strainBovineC123,supercladeA).
strain_has_superclade(strainBovineC156,supercladeA).
strain_has_superclade(strainBovineC180,supercladeA).
strain_has_superclade(strainBovineC30,supercladeA).
strain_has_superclade(strainBovinevineine13305,supercladeB).
strain_has_superclade(strainCalf2,supercladeB).
strain_has_superclade(strainCalf3,supercladeB).
strain_has_superclade(strainCalf5,supercladeA).
strain_has_superclade(strainCamel0014,supercladeA).
strain_has_superclade(strainCamel0029,supercladeA).
strain_has_superclade(strainCamel0032,supercladeA).
strain_has_superclade(strainCamel0037,supercladeA).
strain_has_superclade(strainCamel0049,supercladeA).
strain_has_superclade(strainCamel0062,supercladeA).
strain_has_superclade(strainCamel0064,supercladeA).
strain_has_superclade(strainCamel0079,supercladeA).
strain_has_superclade(strainCamel0086,supercladeA).
strain_has_superclade(strainCattel11,supercladeA).
strain_has_superclade(strainCattle10,supercladeB).
strain_has_superclade(strainCattle8_O,supercladeA).
strain_has_superclade(strainChicken12196,supercladeA).
strain_has_superclade(strainChicken40209,supercladeA).
strain_has_superclade(strainChicken40267,supercladeA).
strain_has_superclade(strainChicken47693,supercladeB).
strain_has_superclade(strainChicken59214,supercladeA).
strain_has_superclade(strainChicken91B1,supercladeA).
strain_has_superclade(strainChicken9B39,supercladeA).
strain_has_superclade(strainChickenicken11818,supercladeA).
strain_has_superclade(strainChickenicken11848,supercladeA).
strain_has_superclade(strainChickenicken11856,supercladeA).
strain_has_superclade(strainChickenicken11919,supercladeA).
strain_has_superclade(strainChickenicken11973,supercladeA).
strain_has_superclade(strainChickenicken11974,supercladeA).
strain_has_superclade(strainChickenicken12450,supercladeA).
strain_has_superclade(strainChickenicken12487,supercladeA).
strain_has_superclade(strainChickenicken12567,supercladeA).
strain_has_superclade(strainChickenicken12912,supercladeA).
strain_has_superclade(strainChickenicken13040,supercladeA).
strain_has_superclade(strainChickenicken13082,supercladeA).
strain_has_superclade(strainChickenicken13249,supercladeA).
strain_has_superclade(strainChickenicken13411,supercladeA).
strain_has_superclade(strainChickenicken13713,supercladeA).
strain_has_superclade(strainCjejuni11168,supercladeA).
strain_has_superclade(strainCjejuniRM1221,supercladeB).
strain_has_superclade(strainClinical18836,supercladeB).
strain_has_superclade(strainClinical30280,supercladeA).
strain_has_superclade(strainClinical30328,supercladeA).
```

strain_has_superclade(strainClinical31481,supercladeB).
strain_has_superclade(strainClinical31485,supercladeA).
strain_has_superclade(strainClinical32787,supercladeB).
strain_has_superclade(strainClinical32799,supercladeA).
strain_has_superclade(strainClinical33084,supercladeB).
strain_has_superclade(strainClinical33106,supercladeB).
strain_has_superclade(strainClinical34007,supercladeB).
strain_has_superclade(strainClinical35424,supercladeB).
strain_has_superclade(strainClinical35535,supercladeA).
strain_has_superclade(strainClinical35799,supercladeB).
strain_has_superclade(strainClinical36069,supercladeB).
strain_has_superclade(strainClinical36439,supercladeB).
strain_has_superclade(strainClinical36860,supercladeB).
strain_has_superclade(strainClinical36952,supercladeA).
strain_has_superclade(strainClinical37537,supercladeA).
strain_has_superclade(strainClinical38553,supercladeB).
strain_has_superclade(strainClinical38762,supercladeB).
strain_has_superclade(strainClinical38857,supercladeB).
strain_has_superclade(strainClinical39828,supercladeA).
strain_has_superclade(strainClinical40671,supercladeB).
strain_has_superclade(strainClinical40917,supercladeB).
strain_has_superclade(strainClinical41651,supercladeA).
strain_has_superclade(strainClinical42724,supercladeB).
strain_has_superclade(strainClinical43205,supercladeB).
strain_has_superclade(strainClinical43983,supercladeB).
strain_has_superclade(strainClinical44119,supercladeB).
strain_has_superclade(strainClinical44811,supercladeA).
strain_has_superclade(strainClinical44933,supercladeB).
strain_has_superclade(strainClinical44958,supercladeA).
strain_has_superclade(strainClinical45557,supercladeA).
strain_has_superclade(strainClinical45631,supercladeB).
strain_has_superclade(strainClinical47886,supercladeA).
strain_has_superclade(strainClinical47939,supercladeB).
strain_has_superclade(strainClinical48612,supercladeA).
strain_has_superclade(strainClinical52331,supercladeA).
strain_has_superclade(strainClinical52368,supercladeB).
strain_has_superclade(strainClinical52471,supercladeB).
strain_has_superclade(strainClinical53250,supercladeB).
strain_has_superclade(strainClinical55320,supercladeB).
strain_has_superclade(strainClinical55703,supercladeA).
strain_has_superclade(strainClinical56281,supercladeA).
strain_has_superclade(strainClinical56282,supercladeA).
strain_has_superclade(strainClinical56519,supercladeB).
strain_has_superclade(strainClinical56832,supercladeB).
strain_has_superclade(strainClinical58473,supercladeA).
strain_has_superclade(strainClinical59364,supercladeB).
strain_has_superclade(strainClinical59424,supercladeB).
strain_has_superclade(strainClinical62914,supercladeB).
strain_has_superclade(strainClinical63326,supercladeB).
strain_has_superclade(strainClinical64555,supercladeB).
strain_has_superclade(strainClinical81116,supercladeB).
strain_has_superclade(strainClinicalF1,supercladeA).
strain_has_superclade(strainClinicalF2,supercladeA).
strain_has_superclade(strainClinicalF3,supercladeA).

strain_has_superclade(strainClinicalG1,supercladeA).
strain_has_superclade(strainClinicalG2,supercladeA).
strain_has_superclade(strainClinicalG3,supercladeA).
strain_has_superclade(strainClinicalG4,supercladeA).
strain_has_superclade(strainClinicalinical15168,supercladeB).
strain_has_superclade(strainClinicall31467,supercladeB).
strain_has_superclade(strainClinicall38556,supercladeB).
strain_has_superclade(strainClinicall39182,supercladeB).
strain_has_superclade(strainClinicalM1,supercladeB).
strain_has_superclade(strainGoose126,supercladeB).
strain_has_superclade(strainGoose222,supercladeB).
strain_has_superclade(strainGoose66,supercladeA).
strain_has_superclade(strainGoose86,supercladeB).
strain_has_superclade(strainHi40500471,supercladeB).
strain_has_superclade(strainHi40520410,supercladeB).
strain_has_superclade(strainHi40540310,supercladeA).
strain_has_superclade(strainHi40540311,supercladeB).
strain_has_superclade(strainHi40620293,supercladeA).
strain_has_superclade(strainHi40620296,supercladeB).
strain_has_superclade(strainHi40620300,supercladeB).
strain_has_superclade(strainHi40680224,supercladeB).
strain_has_superclade(strainHi40800231,supercladeB).
strain_has_superclade(strainHi40840318,supercladeA).
strain_has_superclade(strainHi40840324,supercladeB).
strain_has_superclade(strainHi40900388,supercladeB).
strain_has_superclade(strainHi40980306,supercladeB).
strain_has_superclade(strainHi41000246,supercladeA).
strain_has_superclade(strainHi41020285,supercladeA).
strain_has_superclade(strainHi41040340,supercladeB).
strain_has_superclade(strainHi41080433,supercladeA).
strain_has_superclade(strainHi41080434,supercladeA).
strain_has_superclade(strainHi41100305,supercladeB).
strain_has_superclade(strainHi41120166,supercladeA).
strain_has_superclade(strainHi41120167,supercladeA).
strain_has_superclade(strainHi41160244,supercladeA).
strain_has_superclade(strainHi41220381,supercladeA).
strain_has_superclade(strainHi41220382,supercladeA).
strain_has_superclade(strainHi41300251,supercladeB).
strain_has_superclade(strainHi41300252,supercladeB).
strain_has_superclade(strainHi41300259,supercladeB).
strain_has_superclade(strainHi41360174,supercladeA).
strain_has_superclade(strainHi41380304,supercladeA).
strain_has_superclade(strainHi41500294,supercladeB).
strain_has_superclade(strainHi41720262,supercladeB).
strain_has_superclade(strainHi41760156,supercladeB).
strain_has_superclade(strainHi41780227,supercladeB).
strain_has_superclade(strainHi41780229,supercladeB).
strain_has_superclade(strainHi41800342,supercladeB).
strain_has_superclade(strainHi41840334,supercladeB).
strain_has_superclade(strainHi41840336,supercladeB).
strain_has_superclade(strainHi41880318,supercladeA).
strain_has_superclade(strainHi42080176,supercladeB).
strain_has_superclade(strainHi43240534,supercladeB).
strain_has_superclade(strainHi43240536,supercladeB).

strain_has_superclade(strainHi43320472rept,supercladeB).
strain_has_superclade(strainHi43320475,supercladeA).
strain_has_superclade(strainHi43340233,supercladeA).
strain_has_superclade(strainHi43380463,supercladeB).
strain_has_superclade(strainHi43400514,supercladeB).
strain_has_superclade(strainHi43480582,supercladeB).
strain_has_superclade(strainHi43500234,supercladeA).
strain_has_superclade(strainHi43580563,supercladeB).
strain_has_superclade(strainHi43700324,supercladeB).
strain_has_superclade(strainHi50520408,supercladeB).
strain_has_superclade(strainHi80512,supercladeB).
strain_has_superclade(strainHi80530,supercladeB).
strain_has_superclade(strainHi80531,supercladeA).
strain_has_superclade(strainHi80536,supercladeB).
strain_has_superclade(strainHi80547,supercladeA).
strain_has_superclade(strainHi80552,supercladeA).
strain_has_superclade(strainHi80554,supercladeA).
strain_has_superclade(strainHi80563,supercladeB).
strain_has_superclade(strainHi80583,supercladeA).
strain_has_superclade(strainHi80584,supercladeB).
strain_has_superclade(strainHi80586,supercladeA).
strain_has_superclade(strainHi80614,supercladeB).
strain_has_superclade(strainHi80646,supercladeB).
strain_has_superclade(strainHi80650,supercladeA).
strain_has_superclade(strainHi80659,supercladeB).
strain_has_superclade(strainHi80675,supercladeB).
strain_has_superclade(strainHi80809,supercladeB).
strain_has_superclade(strainHi80838,supercladeB).
strain_has_superclade(strainHi80841,supercladeB).
strain_has_superclade(strainHi80864,supercladeA).
strain_has_superclade(strainHi80866,supercladeB).
strain_has_superclade(strainHi80876,supercladeB).
strain_has_superclade(strainHi80884,supercladeB).
strain_has_superclade(strainHi80885,supercladeA).
strain_has_superclade(strainHi80889,supercladeA).
strain_has_superclade(strainHi80896,supercladeA).
strain_has_superclade(strainHi80901,supercladeA).
strain_has_superclade(strainHi80908,supercladeB).
strain_has_superclade(strainHi80935,supercladeB).
strain_has_superclade(strainHi80938,supercladeA).
strain_has_superclade(strainHi80939,supercladeB).
strain_has_superclade(strainHi80983,supercladeA).
strain_has_superclade(strainHi80986,supercladeA).
strain_has_superclade(strainHi81003,supercladeA).
strain_has_superclade(strainHi81005,supercladeB).
strain_has_superclade(strainHi81006rept,supercladeA).
strain_has_superclade(strainHi81006,supercladeA).
strain_has_superclade(strainHi81018,supercladeB).
strain_has_superclade(strainHi81035,supercladeB).
strain_has_superclade(strainHi81036,supercladeB).
strain_has_superclade(strainHi81040,supercladeB).
strain_has_superclade(strainHi81049,supercladeB).
strain_has_superclade(strainHi81061,supercladeB).
strain_has_superclade(strainHi81062rep,supercladeB).

strain_has_superclade(strainHi81109,supercladeA).
strain_has_superclade(strainHi81121,supercladeB).
strain_has_superclade(strainHi81132,supercladeA).
strain_has_superclade(strainHi81135,supercladeA).
strain_has_superclade(strainHi81139,supercladeB).
strain_has_superclade(strainHi81143,supercladeB).
strain_has_superclade(strainHi81180,supercladeA).
strain_has_superclade(strainHi81181,supercladeB).
strain_has_superclade(strainHi81205,supercladeB).
strain_has_superclade(strainHi81206,supercladeA).
strain_has_superclade(strainHi81214,supercladeB).
strain_has_superclade(strainHi81239,supercladeB).
strain_has_superclade(strainHi81266,supercladeA).
strain_has_superclade(strainHi81268,supercladeA).
strain_has_superclade(strainHi81270,supercladeA).
strain_has_superclade(strainHi81276,supercladeA).
strain_has_superclade(strainHi81289,supercladeA).
strain_has_superclade(strainHi81290,supercladeA).
strain_has_superclade(strainHi81292,supercladeB).
strain_has_superclade(strainHi81311,supercladeB).
strain_has_superclade(strainHi81335,supercladeA).
strain_has_superclade(strainHi81338,supercladeB).
strain_has_superclade(strainHi81342,supercladeA).
strain_has_superclade(strainHi81357,supercladeB).
strain_has_superclade(strainHi81363,supercladeB).
strain_has_superclade(strainHi81371,supercladeB).
strain_has_superclade(strainLamb11,supercladeB).
strain_has_superclade(strainMilk1398,supercladeB).
strain_has_superclade(strainMilk1403,supercladeA).
strain_has_superclade(strainMilk1899_BA,supercladeA).
strain_has_superclade(strainMilk1899_BC,supercladeB).
strain_has_superclade(strainOvine12241,supercladeB).
strain_has_superclade(strainOvine12481,supercladeB).
strain_has_superclade(strainOvineC55,supercladeA).
strain_has_superclade(strainOvineSM4,supercladeA).
strain_has_superclade(strainOvineSM6,supercladeA).
strain_has_superclade(strainOvineSM8,supercladeA).
strain_has_superclade(strainSheep11,supercladeA).
strain_has_superclade(strainSheep18,supercladeA).
strain_has_superclade(strainSheep2,supercladeA).
strain_has_superclade(strainSheep8,supercladeB).
strain_has_superclade(strainSheepSM7,supercladeB).
strain_has_superclade(strainSheepSM9,supercladeA).
strain_has_superclade(strainStarling86857,supercladeB).
strain_has_superclade(strainTurkey4,supercladeA).
strain_has_superclade(strainTurkey8,supercladeB).
strain_has_superclade(strainWaterS5,supercladeB).
strain_has_superclade(strainWaterSA5,supercladeB).

```
%   CPS_PATHWAY_PL
%   Campylobacter jejuni capsule pathway compiled from KEGG and BioCyc
%   augmented by some specific information about the C. jejuni capsul
%   glycan structure that were extracted from publications.

species_t(cje).
db_t(brendan).

pathway_t(capsule).
pathway_name(brendan,cje,capsule,'Capsule synthesis pathway').

subpathway_t(cap_hep).
subpathway(brendan,cje,cap_hep,capsule).
subpathway_name(brendan,cje,cap_hep,'Heptose biosynthesis').

%% Hep biosynthesis component (alternative to that given for LOS pathway above)

compound_t('gdp-dd-hep').
compound(brendan,cje,'gdp-dd-hep').
compound_name(brendan,'gdp-dd-hep','GDP-D,D-heptose').

compound_t('gdp-d-al-gluco-hep').
compound(brendan,cje,'gdp-d-al-gluco-hep').
compound_name(brendan,'gdp-d-al-gluco-hep','GDP-D-glycero-alpha-L-glucoheptose').


reaction_t(capsule_hep1).
reaction(brendan,cje,capsule_hep1,'d-sedohep-7-p','dd-hep-7-p',1).
reaction_gene(brendan,cje,capsule_hep1,cj1424c).
reaction_subpathway(brendan,cje,capsule_hep1,cap_hep).
reaction_pathway(brendan,cje,capsule_hep1,capsule).

gene_t(cj1424c).
gene(brendan,cje,cj1424c).
gene_name(brendan,cj1424c,'GmhA2').

reaction_t(capsule_hep2).
reaction_gene(brendan,cje,capsule_hep2,cj1425c).
reaction(brendan,cje,capsule_hep2,'dd-hep-7-p','dd-hep-17-pp',1).
reaction_subpathway(brendan,cje,capsule_hep2,cap_hep).
reaction_pathway(brendan,cje,capsule_hep2,capsule).

gene_t(cj1425c).
gene(brendan,cje,cj1425c).
gene_name(brendan,cj1425c,'HddA').

reaction_t(capsule_hep3).
reaction_gene(brendan,cje,capsule_hep3,cj1152c).
reaction(brendan,cje,capsule_hep3,'dd-hep-17-pp','dd-hep-1-p',1).
reaction_subpathway(brendan,cje,capsule_hep3,cap_hep).
reaction_pathway(brendan,cje,capsule_hep3,capsule).

reaction_t(capsule_hep4).
```

```
reaction_gene(brendan,cje,capsule_hep4,cj1423c).
reaction(brendan,cje,capsule_hep4,'dd-hep-1-p','gdp-dd-hep',1).
reaction_subpathway(brendan,cje,capsule_hep4,cap_hep).
reaction_pathway(brendan,cje,capsule_hep4,capsule).

gene_t(cj1423c).
gene(brendan,cje,cj1423c).
gene_name(brendan,cj1423c,'HddC').

reaction_t(capsule_hep5).
reaction_gene(brendan,cje,capsule_hep5,cj1427c).
reaction_gene(brendan,cje,capsule_hep5,cj1428c).
reaction_gene(brendan,cje,capsule_hep5,cj1430c).
reaction(brendan,cje,capsule_hep5,'gdp-dd-hep','gdp-d-al-gluco-hep',1).
reaction_subpathway(brendan,cje,capsule_hep5,cap_hep).
reaction_pathway(brendan,cje,capsule_hep5,capsule).

gene_t(cj1427c).
gene(brendan,cje,cj1427c).
gene_name(brendan,cj1427c,'Putative UDP-glucose 4-epimerase').
gene_t(cj1428c).
gene(brendan,cje,cj1428c).
gene_name(brendan,cj1428c,'Fcl').
gene_t(cj1430c).
gene(brendan,cje,cj1430c).
gene_name(brendan,cj1430c,'Putative dTDP-4-dehydrorhamnose 3,5-epimerase').


%% adding next unit to chain

compound_t('capsule_chain').
compound(brendan,cje,'capsule_chain').
compound_name(brendan,'capsule_chain','Capsule chain (X)').

compound_t('glca').
compound(brendan,cje,'glca').
compound_name(brendan,'glca','GlcA-X').

compound_t('glca6ngro').
compound(brendan,cje,'glca6ngro').
compound_name(brendan,'glca6ngro','GlcA6\(NGro\)-X').

compound_t('glca6etn').
compound(brendan,cje,'glca6etn').
compound_name(brendan,'glca6etn','GlcA6\(EtN\)-X').


%% for glca6ngro

%% side Hep group + phase variable modifications

compound_t('glca6ngro+hep').
compound(brendan,cje,'glca6ngro+hep').
compound_name(brendan,'glca6ngro+hep','GlcA6\(NGro\)+Hep-X').
```

```
compound_t('glca6ngro+hep+omepn').
compound(brendan,cje,'glca6ngro+hep+omepn').
compound_name(brendan,'glca6ngro+hep+omepn','GlcA6\(NGro\)+\(Hep+OMePN\)-X').

compound_t('glca6ngro+hep+3ome').
compound(brendan,cje,'glca6ngro+hep+3ome').
compound_name(brendan,'glca6ngro+hep+3ome','GlcA6\(NGro\)+\(Hep+3OMe\)-X').

compound_t('glca6ngro+hep+6ome').
compound(brendan,cje,'glca6ngro+hep+6ome').
compound_name(brendan,'glca6ngro+hep+6ome','GlcA6\(NGro\)+\(Hep+6OMe\)-X').

compound_t('glca6ngro+hep+omepn+3ome').
compound(brendan,cje,'glca6ngro+hep+omepn+3ome').
compound_name(brendan,'glca6ngro+hep+omepn+3ome','GlcA6\(NGro\)+\(Hep+OMePN+3OMe\)-X').

compound_t('glca6ngro+hep+omepn+6ome').
compound(brendan,cje,'glca6ngro+hep+omepn+6ome').
compound_name(brendan,'glca6ngro+hep+omepn+6ome','GlcA6\(NGro\)+\(Hep+OMePN+6OMe\)-X').

compound_t('glca6ngro+hep+3ome+6ome').
compound(brendan,cje,'glca6ngro+hep+3ome+6ome').
compound_name(brendan,'glca6ngro+hep+3ome+6ome','GlcA6\(NGro\)+\(Hep+3OMe+6OMe\)-X').

compound_t('glca6ngro+hep+omepn+3ome+6ome').
compound(brendan,cje,'glca6ngro+hep+omepn+3ome+6ome').
compound_name(brendan,'glca6ngro+hep+omepn+3ome+6ome','GlcA6\(NGro\)+\(Hep+OMePN+3OMe+6OMe\)-X').

%% GalfNAc group + phase variable group

compound_t('glca6ngro-galfnac').
compound(brendan,cje,'glca6ngro-galfnac').
compound_name(brendan,'glca6ngro-galfnac','GalfNAc-GlcA6\(NGro\)-X').

compound_t('glca6ngro+hep-galfnac').
compound(brendan,cje,'glca6ngro+hep-galfnac').
compound_name(brendan,'glca6ngro+hep-galfnac','GalfNAc-GlcA6\(NGro\)+Hep-X').

compound_t('glca6ngro+hep+omepn-galfnac').
compound(brendan,cje,'glca6ngro+hep+omepn-galfnac').
compound_name(brendan,'glca6ngro+hep+omepn-galfnac','GalfNAc-GlcA6\(NGro\)+\(Hep+OMePN\)-X').

compound_t('glca6ngro+hep+3ome-galfnac').
compound(brendan,cje,'glca6ngro+hep+3ome-galfnac').
compound_name(brendan,'glca6ngro+hep+3ome-galfnac','GalfNAc-GlcA6\(NGro\)+\(Hep+3OMe\)-X').

compound_t('glca6ngro+hep+6ome-galfnac').
compound(brendan,cje,'glca6ngro+hep+6ome-galfnac').
compound_name(brendan,'glca6ngro+hep+6ome-galfnac','GalfNAc-GlcA6\(NGro\)+\(Hep+6OMe\)-X').

compound_t('glca6ngro+hep+omepn+3ome-galfnac').
compound(brendan,cje,'glca6ngro+hep+omepn+3ome-galfnac').
compound_name(brendan,'glca6ngro+hep+omepn+3ome-galfnac','GalfNAc-GlcA6\(NGro\)+\(Hep+OMePN+3OMe\)-
```

X').

compound_t('glca6ngro+hep+omepn+6ome-galfnac').
compound(brendan,cje,'glca6ngro+hep+omepn+6ome-galfnac').
compound_name(brendan,'glca6ngro+hep+omepn+6ome-galfnac','GalfNAc-GlcA6\(NGro\)+\(Hep+OMePN+6OMe\)-X').

compound_t('glca6ngro+hep+3ome+6ome-galfnac').
compound(brendan,cje,'glca6ngro+hep+3ome+6ome-galfnac').
compound_name(brendan,'glca6ngro+hep+3ome+6ome-galfnac','GalfNAc-GlcA6\(NGro\)+\(Hep+3OMe+6OMe\)-X').

compound_t('glca6ngro+hep+omepn+3ome+6ome-galfnac').
compound(brendan,cje,'glca6ngro+hep+omepn+3ome+6ome-galfnac').
compound_name(brendan,'glca6ngro+hep+omepn+3ome+6ome-galfnac','GalfNAc-GlcA6\(NGro\)+\(Hep+OMePN+3OMe+6OMe\)-X').

compound_t('glca6ngro-galfnac+omepn').
compound(brendan,cje,'glca6ngro-galfnac+omepn').
compound_name(brendan,'glca6ngro-galfnac+omepn','GalfNAc+OMePN-GlcA6\(NGro\)-X').

compound_t('glca6ngro+hep-galfnac+omepn').
compound(brendan,cje,'glca6ngro+hep-galfnac+omepn').
compound_name(brendan,'glca6ngro+hep-galfnac+omepn','GalfNAc+OMePN-GlcA6\(NGro\)+Hep-X').

compound_t('glca6ngro+hep+omepn-galfnac+omepn').
compound(brendan,cje,'glca6ngro+hep+omepn-galfnac+omepn').
compound_name(brendan,'glca6ngro+hep+omepn-galfnac+omepn','GalfNAc+OMePN-GlcA6\(NGro\)+\(Hep+OMePN\)-X').

compound_t('glca6ngro+hep+3ome-galfnac+omepn').
compound(brendan,cje,'glca6ngro+hep+3ome-galfnac+omepn').
compound_name(brendan,'glca6ngro+hep+3ome-galfnac+omepn','GalfNAc+OMePN-GlcA6\(NGro\)+\(Hep+3OMe\)-X').

compound_t('glca6ngro+hep+6ome-galfnac+omepn').
compound(brendan,cje,'glca6ngro+hep+6ome-galfnac+omepn').
compound_name(brendan,'glca6ngro+hep+6ome-galfnac+omepn','GalfNAc+OMePN-GlcA6\(NGro\)+\(Hep+6OMe\)-X').

compound_t('glca6ngro+hep+omepn+3ome-galfnac+omepn').
compound(brendan,cje,'glca6ngro+hep+omepn+3ome-galfnac+omepn').
compound_name(brendan,'glca6ngro+hep+omepn+3ome-galfnac+omepn','GalfNAc+OMePN-GlcA6\(NGro\)+\(Hep+OMePN+3OMe\)-X').

compound_t('glca6ngro+hep+omepn+6ome-galfnac+omepn').
compound(brendan,cje,'glca6ngro+hep+omepn+6ome-galfnac+omepn').
compound_name(brendan,'glca6ngro+hep+omepn+6ome-galfnac+omepn','GalfNAc+OMePN-GlcA6\(NGro\)+\(Hep+OMePN+6OMe\)-X').

compound_t('glca6ngro+hep+3ome+6ome-galfnac+omepn').
compound(brendan,cje,'glca6ngro+hep+3ome+6ome-galfnac+omepn').
compound_name(brendan,'glca6ngro+hep+3ome+6ome-galfnac+omepn','GalfNAc+OMePN-GlcA6\(NGro\)+\(Hep+3OMe+6OMe\)-X').

```
compound_t('glca6ngro+hep+omepn+3ome+6ome-galfnac+omepn').
compound(brendan,cje,'glca6ngro+hep+omepn+3ome+6ome-galfnac+omepn').
compound_name(brendan,'glca6ngro+hep+omepn+3ome+6ome-galfnac+omepn','GalfNAc+OMePN-
GlcA6\(NGro\)+\(Hep+OMePN+3OMe+6OMe\)-X').

%% Ribf group

compound_t('glca6ngro-galfnac-ribf').
compound(brendan,cje,'glca6ngro-galfnac-ribf').
compound_name(brendan,'glca6ngro-galfnac-ribf','Ribf-GalfNAc-GlcA6\(NGro\)-X').

compound_t('glca6ngro+hep-galfnac-ribf').
compound(brendan,cje,'glca6ngro+hep-galfnac-ribf').
compound_name(brendan,'glca6ngro+hep-galfnac-ribf','Ribf-GalfNAc-GlcA6\(NGro\)+Hep-X').

compound_t('glca6ngro+hep+omepn-galfnac-ribf').
compound(brendan,cje,'glca6ngro+hep+omepn-galfnac-ribf').
compound_name(brendan,'glca6ngro+hep+omepn-galfnac-ribf','Ribf-GalfNAc-GlcA6\(NGro\)+\(Hep+OMePN\)-X').

compound_t('glca6ngro+hep+3ome-galfnac-ribf').
compound(brendan,cje,'glca6ngro+hep+3ome-galfnac-ribf').
compound_name(brendan,'glca6ngro+hep+3ome-galfnac-ribf','Ribf-GalfNAc-GlcA6\(NGro\)+\(Hep+3OMe\)-X').

compound_t('glca6ngro+hep+6ome-galfnac-ribf').
compound(brendan,cje,'glca6ngro+hep+6ome-galfnac-ribf').
compound_name(brendan,'glca6ngro+hep+6ome-galfnac-ribf','Ribf-GalfNAc-GlcA6\(NGro\)+\(Hep+6OMe\)-X').

compound_t('glca6ngro+hep+omepn+3ome-galfnac-ribf').
compound(brendan,cje,'glca6ngro+hep+omepn+3ome-galfnac-ribf').
compound_name(brendan,'glca6ngro+hep+omepn+3ome-galfnac-ribf','Ribf-GalfNAc-
GlcA6\(NGro\)+\(Hep+OMePN+3OMe\)-X').

compound_t('glca6ngro+hep+omepn+6ome-galfnac-ribf').
compound(brendan,cje,'glca6ngro+hep+omepn+6ome-galfnac-ribf').
compound_name(brendan,'glca6ngro+hep+omepn+6ome-galfnac-ribf','Ribf-GalfNAc-
GlcA6\(NGro\)+\(Hep+OMePN+6OMe\)-X').

compound_t('glca6ngro+hep+3ome+6ome-galfnac-ribf').
compound(brendan,cje,'glca6ngro+hep+3ome+6ome-galfnac-ribf').
compound_name(brendan,'glca6ngro+hep+3ome+6ome-galfnac-ribf','Ribf-GalfNAc-
GlcA6\(NGro\)+\(Hep+3OMe+6OMe\)-X').

compound_t('glca6ngro+hep+omepn+3ome+6ome-galfnac-ribf').
compound(brendan,cje,'glca6ngro+hep+omepn+3ome+6ome-galfnac-ribf').
compound_name(brendan,'glca6ngro+hep+omepn+3ome+6ome-galfnac-ribf','Ribf-GalfNAc-
GlcA6\(NGro\)+\(Hep+OMePN+3OMe+6OMe\)-X').

compound_t('glca6ngro-galfnac+omepn-ribf').
compound(brendan,cje,'glca6ngro-galfnac+omepn-ribf').
compound_name(brendan,'glca6ngro-galfnac+omepn-ribf','Ribf-GalfNAc+OMePN-GlcA6\(NGro\)-X').

compound_t('glca6ngro+hep-galfnac+omepn-ribf').
compound(brendan,cje,'glca6ngro+hep-galfnac+omepn-ribf').
compound_name(brendan,'glca6ngro+hep-galfnac+omepn-ribf','Ribf-GalfNAc+OMePN-GlcA6\(NGro\)+Hep-X').
```

```
compound_t('glca6ngro+hep+omepn-galfnac+omepn-ribf').
compound(brendan,cje,'glca6ngro+hep+omepn-galfnac+omepn-ribf').
compound_name(brendan,'glca6ngro+hep+omepn-galfnac+omepn-ribf','Ribf-GalfNAc+OMePN-GlcA6\(NGro\)+\(Hep+OMePN\)-X').

compound_t('glca6ngro+hep+3ome-galfnac+omepn-ribf').
compound(brendan,cje,'glca6ngro+hep+3ome-galfnac+omepn-ribf').
compound_name(brendan,'glca6ngro+hep+3ome-galfnac+omepn-ribf','Ribf-GalfNAc+OMePN-GlcA6\(NGro\)+\(Hep+3OMe\)-X').

compound_t('glca6ngro+hep+6ome-galfnac+omepn-ribf').
compound(brendan,cje,'glca6ngro+hep+6ome-galfnac+omepn-ribf').
compound_name(brendan,'glca6ngro+hep+6ome-galfnac+omepn-ribf','Ribf-GalfNAc+OMePN-GlcA6\(NGro\)+\(Hep+6OMe\)-X').

compound_t('glca6ngro+hep+omepn+3ome-galfnac+omepn-ribf').
compound(brendan,cje,'glca6ngro+hep+omepn+3ome-galfnac+omepn-ribf').
compound_name(brendan,'glca6ngro+hep+omepn+3ome-galfnac+omepn-ribf','Ribf-GalfNAc+OMePN-GlcA6\(NGro\)+\(Hep+OMePN+3OMe\)-X').

compound_t('glca6ngro+hep+omepn+6ome-galfnac+omepn-ribf').
compound(brendan,cje,'glca6ngro+hep+omepn+6ome-galfnac+omepn-ribf').
compound_name(brendan,'glca6ngro+hep+omepn+6ome-galfnac+omepn-ribf','Ribf-GalfNAc+OMePN-GlcA6\(NGro\)+\(Hep+OMePN+6OMe\)-X').

compound_t('glca6ngro+hep+3ome+6ome-galfnac+omepn-ribf').
compound(brendan,cje,'glca6ngro+hep+3ome+6ome-galfnac+omepn-ribf').
compound_name(brendan,'glca6ngro+hep+3ome+6ome-galfnac+omepn-ribf','Ribf-GalfNAc+OMePN-GlcA6\(NGro\)+\(Hep+3OMe+6OMe\)-X').

compound_t('glca6ngro+hep+omepn+3ome+6ome-galfnac+omepn-ribf').
compound(brendan,cje,'glca6ngro+hep+omepn+3ome+6ome-galfnac+omepn-ribf').
compound_name(brendan,'glca6ngro+hep+omepn+3ome+6ome-galfnac+omepn-ribf','Ribf-GalfNAc+OMePN-GlcA6\(NGro\)+\(Hep+OMePN+3OMe+6OMe\)-X').


%% now for EtN phase variable modification

%% side Hep group + phase variable modifications

compound_t('glca6etn+hep').
compound(brendan,cje,'glca6etn+hep').
compound_name(brendan,'glca6etn+hep','GlcA6\(EtN\)+Hep-X').

compound_t('glca6etn+hep+omepn').
compound(brendan,cje,'glca6etn+hep+omepn').
compound_name(brendan,'glca6etn+hep+omepn','GlcA6\(EtN\)+\(Hep+OMePN\)-X').

compound_t('glca6etn+hep+3ome').
compound(brendan,cje,'glca6etn+hep+3ome').
compound_name(brendan,'glca6etn+hep+3ome','GlcA6\(EtN\)+\(Hep+3OMe\)-X').

compound_t('glca6etn+hep+6ome').
```

```
compound(brendan,cje,'glca6etn+hep+6ome').
compound_name(brendan,'glca6etn+hep+6ome','GlcA6\(EtN\)+\(Hep+6OMe\)-X').

compound_t('glca6etn+hep+omepn+3ome').
compound(brendan,cje,'glca6etn+hep+omepn+3ome').
compound_name(brendan,'glca6etn+hep+omepn+3ome','GlcA6\(EtN\)+\(Hep+OMePN+3OMe\)-X').

compound_t('glca6etn+hep+omepn+6ome').
compound(brendan,cje,'glca6etn+hep+omepn+6ome').
compound_name(brendan,'glca6etn+hep+omepn+6ome','GlcA6\(EtN\)+\(Hep+OMePN+6OMe\)-X').

compound_t('glca6etn+hep+3ome+6ome').
compound(brendan,cje,'glca6etn+hep+3ome+6ome').
compound_name(brendan,'glca6etn+hep+3ome+6ome','GlcA6\(EtN\)+\(Hep+3OMe+6OMe\)-X').

compound_t('glca6etn+hep+omepn+3ome+6ome').
compound(brendan,cje,'glca6etn+hep+omepn+3ome+6ome').
compound_name(brendan,'glca6etn+hep+omepn+3ome+6ome','GlcA6\(EtN\)+\(Hep+OMePN+3OMe+6OMe\)-X').

%% GalfNAc group + phase variable group

compound_t('glca6etn-galfnac').
compound(brendan,cje,'glca6etn-galfnac').
compound_name(brendan,'glca6etn-galfnac','GalfNAc-GlcA6\(EtN\)-X').

compound_t('glca6etn+hep-galfnac').
compound(brendan,cje,'glca6etn+hep-galfnac').
compound_name(brendan,'glca6etn+hep-galfnac','GalfNAc-GlcA6\(EtN\)+Hep-X').

compound_t('glca6etn+hep+omepn-galfnac').
compound(brendan,cje,'glca6etn+hep+omepn-galfnac').
compound_name(brendan,'glca6etn+hep+omepn-galfnac','GalfNAc-GlcA6\(EtN\)+\(Hep+OMePN\)-X').

compound_t('glca6etn+hep+3ome-galfnac').
compound(brendan,cje,'glca6etn+hep+3ome-galfnac').
compound_name(brendan,'glca6etn+hep+3ome-galfnac','GalfNAc-GlcA6\(EtN\)+\(Hep+3OMe\)-X').

compound_t('glca6etn+hep+6ome-galfnac').
compound(brendan,cje,'glca6etn+hep+6ome-galfnac').
compound_name(brendan,'glca6etn+hep+6ome-galfnac','GalfNAc-GlcA6\(EtN\)+\(Hep+6OMe\)-X').

compound_t('glca6etn+hep+omepn+3ome-galfnac').
compound(brendan,cje,'glca6etn+hep+omepn+3ome-galfnac').
compound_name(brendan,'glca6etn+hep+omepn+3ome-galfnac','GalfNAc-GlcA6\(EtN\)+\(Hep+OMePN+3OMe\)-X').

compound_t('glca6etn+hep+omepn+6ome-galfnac').
compound(brendan,cje,'glca6etn+hep+omepn+6ome-galfnac').
compound_name(brendan,'glca6etn+hep+omepn+6ome-galfnac','GalfNAc-GlcA6\(EtN\)+\(Hep+OMePN+6OMe\)-X').

compound_t('glca6etn+hep+3ome+6ome-galfnac').
compound(brendan,cje,'glca6etn+hep+3ome+6ome-galfnac').
compound_name(brendan,'glca6etn+hep+3ome+6ome-galfnac','GalfNAc-GlcA6\(EtN\)+\(Hep+3OMe+6OMe\)-X').

compound_t('glca6etn+hep+omepn+3ome+6ome-galfnac').
```

```
compound(brendan,cje,'glca6etn+hep+omepn+3ome+6ome-galfnac').
compound_name(brendan,'glca6etn+hep+omepn+3ome+6ome-galfnac','GalfNAc-
GlcA6\(EtN\)+\(Hep+OMePN+3OMe+6OMe\)-X').

compound_t('glca6etn-galfnac+omepn').
compound(brendan,cje,'glca6etn-galfnac+omepn').
compound_name(brendan,'glca6etn-galfnac+omepn','GalfNAc+OMePN-GlcA6\(EtN\)-X').

compound_t('glca6etn+hep-galfnac+omepn').
compound(brendan,cje,'glca6etn+hep-galfnac+omepn').
compound_name(brendan,'glca6etn+hep-galfnac+omepn','GalfNAc+OMePN-GlcA6\(EtN\)+Hep-X').

compound_t('glca6etn+hep+omepn-galfnac+omepn').
compound(brendan,cje,'glca6etn+hep+omepn-galfnac+omepn').
compound_name(brendan,'glca6etn+hep+omepn-galfnac+omepn','GalfNAc+OMePN-GlcA6\(EtN\)+\(Hep+OMePN\)-
X').

compound_t('glca6etn+hep+3ome-galfnac+omepn').
compound(brendan,cje,'glca6etn+hep+3ome-galfnac+omepn').
compound_name(brendan,'glca6etn+hep+3ome-galfnac+omepn','GalfNAc+OMePN-GlcA6\(EtN\)+\(Hep+3OMe\)-X').

compound_t('glca6etn+hep+6ome-galfnac+omepn').
compound(brendan,cje,'glca6etn+hep+6ome-galfnac+omepn').
compound_name(brendan,'glca6etn+hep+6ome-galfnac+omepn','GalfNAc+OMePN-GlcA6\(EtN\)+\(Hep+6OMe\)-X').

compound_t('glca6etn+hep+omepn+3ome-galfnac+omepn').
compound(brendan,cje,'glca6etn+hep+omepn+3ome-galfnac+omepn').
compound_name(brendan,'glca6etn+hep+omepn+3ome-galfnac+omepn','GalfNAc+OMePN-
GlcA6\(EtN\)+\(Hep+OMePN+3OMe\)-X').

compound_t('glca6etn+hep+omepn+6ome-galfnac+omepn').
compound(brendan,cje,'glca6etn+hep+omepn+6ome-galfnac+omepn').
compound_name(brendan,'glca6etn+hep+omepn+6ome-galfnac+omepn','GalfNAc+OMePN-
GlcA6\(EtN\)+\(Hep+OMePN+6OMe\)-X').

compound_t('glca6etn+hep+3ome+6ome-galfnac+omepn').
compound(brendan,cje,'glca6etn+hep+3ome+6ome-galfnac+omepn').
compound_name(brendan,'glca6etn+hep+3ome+6ome-galfnac+omepn','GalfNAc+OMePN-
GlcA6\(EtN\)+\(Hep+3OMe+6OMe\)-X').

compound_t('glca6etn+hep+omepn+3ome+6ome-galfnac+omepn').
compound(brendan,cje,'glca6etn+hep+omepn+3ome+6ome-galfnac+omepn').
compound_name(brendan,'glca6etn+hep+omepn+3ome+6ome-galfnac+omepn','GalfNAc+OMePN-
GlcA6\(EtN\)+\(Hep+OMePN+3OMe+6OMe\)-X').

%% Ribf group

compound_t('glca6etn-galfnac-ribf').
compound(brendan,cje,'glca6etn-galfnac-ribf').
compound_name(brendan,'glca6etn-galfnac-ribf','Ribf-GalfNAc-GlcA6\(EtN\)-X').

compound_t('glca6etn+hep-galfnac-ribf').
compound(brendan,cje,'glca6etn+hep-galfnac-ribf').
compound_name(brendan,'glca6etn+hep-galfnac-ribf','Ribf-GalfNAc-GlcA6\(EtN\)+Hep-X').
```

compound_t('glca6etn+hep+omepn-galfnac-ribf').
compound(brendan,cje,'glca6etn+hep+omepn-galfnac-ribf').
compound_name(brendan,'glca6etn+hep+omepn-galfnac-ribf','Ribf-GalfNAc-GlcA6\(EtN\)+\(Hep+OMePN\)-X').

compound_t('glca6etn+hep+3ome-galfnac-ribf').
compound(brendan,cje,'glca6etn+hep+3ome-galfnac-ribf').
compound_name(brendan,'glca6etn+hep+3ome-galfnac-ribf','Ribf-GalfNAc-GlcA6\(EtN\)+\(Hep+3OMe\)-X').

compound_t('glca6etn+hep+6ome-galfnac-ribf').
compound(brendan,cje,'glca6etn+hep+6ome-galfnac-ribf').
compound_name(brendan,'glca6etn+hep+6ome-galfnac-ribf','Ribf-GalfNAc-GlcA6\(EtN\)+\(Hep+6OMe\)-X').

compound_t('glca6etn+hep+omepn+3ome-galfnac-ribf').
compound(brendan,cje,'glca6etn+hep+omepn+3ome-galfnac-ribf').
compound_name(brendan,'glca6etn+hep+omepn+3ome-galfnac-ribf','Ribf-GalfNAc-
GlcA6\(EtN\)+\(Hep+OMePN+3OMe\)-X').

compound_t('glca6etn+hep+omepn+6ome-galfnac-ribf').
compound(brendan,cje,'glca6etn+hep+omepn+6ome-galfnac-ribf').
compound_name(brendan,'glca6etn+hep+omepn+6ome-galfnac-ribf','Ribf-GalfNAc-
GlcA6\(EtN\)+\(Hep+OMePN+6OMe\)-X').

compound_t('glca6etn+hep+3ome+6ome-galfnac-ribf').
compound(brendan,cje,'glca6etn+hep+3ome+6ome-galfnac-ribf').
compound_name(brendan,'glca6etn+hep+3ome+6ome-galfnac-ribf','Ribf-GalfNAc-
GlcA6\(EtN\)+\(Hep+3OMe+6OMe\)-X').

compound_t('glca6etn+hep+omepn+3ome+6ome-galfnac-ribf').
compound(brendan,cje,'glca6etn+hep+omepn+3ome+6ome-galfnac-ribf').
compound_name(brendan,'glca6etn+hep+omepn+3ome+6ome-galfnac-ribf','Ribf-GalfNAc-
GlcA6\(EtN\)+\(Hep+OMePN+3OMe+6OMe\)-X').

compound_t('glca6etn-galfnac+omepn-ribf').
compound(brendan,cje,'glca6etn-galfnac+omepn-ribf').
compound_name(brendan,'glca6etn-galfnac+omepn-ribf','Ribf-GalfNAc+OMePN-GlcA6\(EtN\)-X').

compound_t('glca6etn+hep-galfnac+omepn-ribf').
compound(brendan,cje,'glca6etn+hep-galfnac+omepn-ribf').
compound_name(brendan,'glca6etn+hep-galfnac+omepn-ribf','Ribf-GalfNAc+OMePN-GlcA6\(EtN\)+Hep-X').

compound_t('glca6etn+hep+omepn-galfnac+omepn-ribf').
compound(brendan,cje,'glca6etn+hep+omepn-galfnac+omepn-ribf').
compound_name(brendan,'glca6etn+hep+omepn-galfnac+omepn-ribf','Ribf-GalfNAc+OMePN-
GlcA6\(EtN\)+\(Hep+OMePN\)-X').

compound_t('glca6etn+hep+3ome-galfnac+omepn-ribf').
compound(brendan,cje,'glca6etn+hep+3ome-galfnac+omepn-ribf').
compound_name(brendan,'glca6etn+hep+3ome-galfnac+omepn-ribf','Ribf-GalfNAc+OMePN-
GlcA6\(EtN\)+\(Hep+3OMe\)-X').

compound_t('glca6etn+hep+6ome-galfnac+omepn-ribf').
compound(brendan,cje,'glca6etn+hep+6ome-galfnac+omepn-ribf').
compound_name(brendan,'glca6etn+hep+6ome-galfnac+omepn-ribf','Ribf-GalfNAc+OMePN-

GlcA6\(EtN\)+\(Hep+6OMe\)-X').

compound_t('glca6etn+hep+omepn+3ome-galfnac+omepn-ribf').
compound(brendan,cje,'glca6etn+hep+omepn+3ome-galfnac+omepn-ribf').
compound_name(brendan,'glca6etn+hep+omepn+3ome-galfnac+omepn-ribf','Ribf-GalfNAc+OMePN-GlcA6\(EtN\)+\(Hep+OMePN+3OMe\)-X').

compound_t('glca6etn+hep+omepn+6ome-galfnac+omepn-ribf').
compound(brendan,cje,'glca6etn+hep+omepn+6ome-galfnac+omepn-ribf').
compound_name(brendan,'glca6etn+hep+omepn+6ome-galfnac+omepn-ribf','Ribf-GalfNAc+OMePN-GlcA6\(EtN\)+\(Hep+OMePN+6OMe\)-X').

compound_t('glca6etn+hep+3ome+6ome-galfnac+omepn-ribf').
compound(brendan,cje,'glca6etn+hep+3ome+6ome-galfnac+omepn-ribf').
compound_name(brendan,'glca6etn+hep+3ome+6ome-galfnac+omepn-ribf','Ribf-GalfNAc+OMePN-GlcA6\(EtN\)+\(Hep+3OMe+6OMe\)-X').

compound_t('glca6etn+hep+omepn+3ome+6ome-galfnac+omepn-ribf').
compound(brendan,cje,'glca6etn+hep+omepn+3ome+6ome-galfnac+omepn-ribf').
compound_name(brendan,'glca6etn+hep+omepn+3ome+6ome-galfnac+omepn-ribf','Ribf-GalfNAc+OMePN-GlcA6\(EtN\)+\(Hep+OMePN+3OMe+6OMe\)-X').

%% for glca without ngro or etn phase variable modification

%% side Hep group + phase variable modifications

compound_t('glca+hep').
compound(brendan,cje,'glca+hep').
compound_name(brendan,'glca+hep','GlcA+Hep-X').

compound_t('glca+hep+omepn').
compound(brendan,cje,'glca+hep+omepn').
compound_name(brendan,'glca+hep+omepn','GlcA+\(Hep+OMePN\)-X').

compound_t('glca+hep+3ome').
compound(brendan,cje,'glca+hep+3ome').
compound_name(brendan,'glca+hep+3ome','GlcA+\(Hep+3OMe\)-X').

compound_t('glca+hep+6ome').
compound(brendan,cje,'glca+hep+6ome').
compound_name(brendan,'glca+hep+6ome','GlcA+\(Hep+6OMe\)-X').

compound_t('glca+hep+omepn+3ome').
compound(brendan,cje,'glca+hep+omepn+3ome').
compound_name(brendan,'glca+hep+omepn+3ome','GlcA+\(Hep+OMePN+3OMe\)-X').

compound_t('glca+hep+omepn+6ome').
compound(brendan,cje,'glca+hep+omepn+6ome').
compound_name(brendan,'glca+hep+omepn+6ome','GlcA+\(Hep+OMePN+6OMe\)-X').

compound_t('glca+hep+3ome+6ome').
compound(brendan,cje,'glca+hep+3ome+6ome').
compound_name(brendan,'glca+hep+3ome+6ome','GlcA+\(Hep+3OMe+6OMe\)-X').

```
compound_t('glca+hep+omepn+3ome+6ome').
compound(brendan,cje,'glca+hep+omepn+3ome+6ome').
compound_name(brendan,'glca+hep+omepn+3ome+6ome','GlcA+\(Hep+OMePN+3OMe+6OMe\)-X').

%% GalfNAc group + phase variable group

compound_t('glca-galfnac').
compound(brendan,cje,'glca-galfnac').
compound_name(brendan,'glca-galfnac','GalfNAc-GlcA-X').

compound_t('glca+hep-galfnac').
compound(brendan,cje,'glca+hep-galfnac').
compound_name(brendan,'glca+hep-galfnac','GalfNAc-GlcA+Hep-X').

compound_t('glca+hep+omepn-galfnac').
compound(brendan,cje,'glca+hep+omepn-galfnac').
compound_name(brendan,'glca+hep+omepn-galfnac','GalfNAc-GlcA+\(Hep+OMePN\)-X').

compound_t('glca+hep+3ome-galfnac').
compound(brendan,cje,'glca+hep+3ome-galfnac').
compound_name(brendan,'glca+hep+3ome-galfnac','GalfNAc-GlcA+\(Hep+3OMe\)-X').

compound_t('glca+hep+6ome-galfnac').
compound(brendan,cje,'glca+hep+6ome-galfnac').
compound_name(brendan,'glca+hep+6ome-galfnac','GalfNAc-GlcA+\(Hep+6OMe\)-X').

compound_t('glca+hep+omepn+3ome-galfnac').
compound(brendan,cje,'glca+hep+omepn+3ome-galfnac').
compound_name(brendan,'glca+hep+omepn+3ome-galfnac','GalfNAc-GlcA+\(Hep+OMePN+3OMe\)-X').

compound_t('glca+hep+omepn+6ome-galfnac').
compound(brendan,cje,'glca+hep+omepn+6ome-galfnac').
compound_name(brendan,'glca+hep+omepn+6ome-galfnac','GalfNAc-GlcA+\(Hep+OMePN+6OMe\)-X').

compound_t('glca+hep+3ome+6ome-galfnac').
compound(brendan,cje,'glca+hep+3ome+6ome-galfnac').
compound_name(brendan,'glca+hep+3ome+6ome-galfnac','GalfNAc-GlcA+\(Hep+3OMe+6OMe\)-X').

compound_t('glca+hep+omepn+3ome+6ome-galfnac').
compound(brendan,cje,'glca+hep+omepn+3ome+6ome-galfnac').
compound_name(brendan,'glca+hep+omepn+3ome+6ome-galfnac','GalfNAc-GlcA+\(Hep+OMePN+3OMe+6OMe\)-
X').

compound_t('glca-galfnac+omepn').
compound(brendan,cje,'glca-galfnac+omepn').
compound_name(brendan,'glca-galfnac+omepn','GalfNAc+OMePN-GlcA-X').

compound_t('glca+hep-galfnac+omepn').
compound(brendan,cje,'glca+hep-galfnac+omepn').
compound_name(brendan,'glca+hep-galfnac+omepn','GalfNAc+OMePN-GlcA+Hep-X').

compound_t('glca+hep+omepn-galfnac+omepn').
compound(brendan,cje,'glca+hep+omepn-galfnac+omepn').
```

compound_name(brendan,'glca+hep+omepn-galfnac+omepn','GalfNAc+OMePN-GlcA+\(Hep+OMePN\)-X').

compound_t('glca+hep+3ome-galfnac+omepn').
compound(brendan,cje,'glca+hep+3ome-galfnac+omepn').
compound_name(brendan,'glca+hep+3ome-galfnac+omepn','GalfNAc+OMePN-GlcA+\(Hep+3OMe\)-X').

compound_t('glca+hep+6ome-galfnac+omepn').
compound(brendan,cje,'glca+hep+6ome-galfnac+omepn').
compound_name(brendan,'glca+hep+6ome-galfnac+omepn','GalfNAc+OMePN-GlcA+\(Hep+6OMe\)-X').

compound_t('glca+hep+omepn+3ome-galfnac+omepn').
compound(brendan,cje,'glca+hep+omepn+3ome-galfnac+omepn').
compound_name(brendan,'glca+hep+omepn+3ome-galfnac+omepn','GalfNAc+OMePN-GlcA+\(Hep+OMePN+3OMe\)-X').

compound_t('glca+hep+omepn+6ome-galfnac+omepn').
compound(brendan,cje,'glca+hep+omepn+6ome-galfnac+omepn').
compound_name(brendan,'glca+hep+omepn+6ome-galfnac+omepn','GalfNAc+OMePN-GlcA+\(Hep+OMePN+6OMe\)-X').

compound_t('glca+hep+3ome+6ome-galfnac+omepn').
compound(brendan,cje,'glca+hep+3ome+6ome-galfnac+omepn').
compound_name(brendan,'glca+hep+3ome+6ome-galfnac+omepn','GalfNAc+OMePN-GlcA+\(Hep+3OMe+6OMe\)-X').

compound_t('glca+hep+omepn+3ome+6ome-galfnac+omepn').
compound(brendan,cje,'glca+hep+omepn+3ome+6ome-galfnac+omepn').
compound_name(brendan,'glca+hep+omepn+3ome+6ome-galfnac+omepn','GalfNAc+OMePN-GlcA+\(Hep+OMePN+3OMe+6OMe\)-X').

%% Ribf group

compound_t('glca-galfnac-ribf').
compound(brendan,cje,'glca-galfnac-ribf').
compound_name(brendan,'glca-galfnac-ribf','Ribf-GalfNAc-GlcA-X').

compound_t('glca+hep-galfnac-ribf').
compound(brendan,cje,'glca+hep-galfnac-ribf').
compound_name(brendan,'glca+hep-galfnac-ribf','Ribf-GalfNAc-GlcA+Hep-X').

compound_t('glca+hep+omepn-galfnac-ribf').
compound(brendan,cje,'glca+hep+omepn-galfnac-ribf').
compound_name(brendan,'glca+hep+omepn-galfnac-ribf','Ribf-GalfNAc-GlcA+\(Hep+OMePN\)-X').

compound_t('glca+hep+3ome-galfnac-ribf').
compound(brendan,cje,'glca+hep+3ome-galfnac-ribf').
compound_name(brendan,'glca+hep+3ome-galfnac-ribf','Ribf-GalfNAc-GlcA+\(Hep+3OMe\)-X').

compound_t('glca+hep+6ome-galfnac-ribf').
compound(brendan,cje,'glca+hep+6ome-galfnac-ribf').
compound_name(brendan,'glca+hep+6ome-galfnac-ribf','Ribf-GalfNAc-GlcA+\(Hep+6OMe\)-X').

compound_t('glca+hep+omepn+3ome-galfnac-ribf').
compound(brendan,cje,'glca+hep+omepn+3ome-galfnac-ribf').

compound_name(brendan,'glca+hep+omepn+3ome-galfnac-ribf','Ribf-GalfNAc-GlcA+\(Hep+OMePN+3OMe\)-X').

compound_t('glca+hep+omepn+6ome-galfnac-ribf').
compound(brendan,cje,'glca+hep+omepn+6ome-galfnac-ribf').
compound_name(brendan,'glca+hep+omepn+6ome-galfnac-ribf','Ribf-GalfNAc-GlcA+\(Hep+OMePN+6OMe\)-X').

compound_t('glca+hep+3ome+6ome-galfnac-ribf').
compound(brendan,cje,'glca+hep+3ome+6ome-galfnac-ribf').
compound_name(brendan,'glca+hep+3ome+6ome-galfnac-ribf','Ribf-GalfNAc-GlcA+\(Hep+3OMe+6OMe\)-X').

compound_t('glca+hep+omepn+3ome+6ome-galfnac-ribf').
compound(brendan,cje,'glca+hep+omepn+3ome+6ome-galfnac-ribf').
compound_name(brendan,'glca+hep+omepn+3ome+6ome-galfnac-ribf','Ribf-GalfNAc-GlcA+\(Hep+OMePN+3OMe+6OMe\)-X').

compound_t('glca-galfnac+omepn-ribf').
compound(brendan,cje,'glca-galfnac+omepn-ribf').
compound_name(brendan,'glca-galfnac+omepn-ribf','Ribf-GalfNAc+OMePN-GlcA-X').

compound_t('glca+hep-galfnac+omepn-ribf').
compound(brendan,cje,'glca+hep-galfnac+omepn-ribf').
compound_name(brendan,'glca+hep-galfnac+omepn-ribf','Ribf-GalfNAc+OMePN-GlcA+Hep-X').

compound_t('glca+hep+omepn-galfnac+omepn-ribf').
compound(brendan,cje,'glca+hep+omepn-galfnac+omepn-ribf').
compound_name(brendan,'glca+hep+omepn-galfnac+omepn-ribf','Ribf-GalfNAc+OMePN-GlcA+\(Hep+OMePN\)-X').

compound_t('glca+hep+3ome-galfnac+omepn-ribf').
compound(brendan,cje,'glca+hep+3ome-galfnac+omepn-ribf').
compound_name(brendan,'glca+hep+3ome-galfnac+omepn-ribf','Ribf-GalfNAc+OMePN-GlcA+\(Hep+3OMe\)-X').

compound_t('glca+hep+6ome-galfnac+omepn-ribf').
compound(brendan,cje,'glca+hep+6ome-galfnac+omepn-ribf').
compound_name(brendan,'glca+hep+6ome-galfnac+omepn-ribf','Ribf-GalfNAc+OMePN-GlcA+\(Hep+6OMe\)-X').

compound_t('glca+hep+omepn+3ome-galfnac+omepn-ribf').
compound(brendan,cje,'glca+hep+omepn+3ome-galfnac+omepn-ribf').
compound_name(brendan,'glca+hep+omepn+3ome-galfnac+omepn-ribf','Ribf-GalfNAc+OMePN-GlcA+\(Hep+OMePN+3OMe\)-X').

compound_t('glca+hep+omepn+6ome-galfnac+omepn-ribf').
compound(brendan,cje,'glca+hep+omepn+6ome-galfnac+omepn-ribf').
compound_name(brendan,'glca+hep+omepn+6ome-galfnac+omepn-ribf','Ribf-GalfNAc+OMePN-GlcA+\(Hep+OMePN+6OMe\)-X').

compound_t('glca+hep+3ome+6ome-galfnac+omepn-ribf').
compound(brendan,cje,'glca+hep+3ome+6ome-galfnac+omepn-ribf').
compound_name(brendan,'glca+hep+3ome+6ome-galfnac+omepn-ribf','Ribf-GalfNAc+OMePN-GlcA+\(Hep+3OMe+6OMe\)-X').

compound_t('glca+hep+omepn+3ome+6ome-galfnac+omepn-ribf').
compound(brendan,cje,'glca+hep+omepn+3ome+6ome-galfnac+omepn-ribf').
compound_name(brendan,'glca+hep+omepn+3ome+6ome-galfnac+omepn-ribf','Ribf-GalfNAc+OMePN-

```
GlcA+\(Hep+OMePN+3OMe+6OMe\)-X').


%% what form of sugars and side groups are involved in reactions??

compound_t('pre-omepn').
compound(brendan,cje,'pre-omepn').
compound_name(brendan,'pre-omepn','Pre-OMePN').

compound_t('pre-ome').
compound(brendan,cje,'pre-ome').
compound_name(brendan,'pre-ome','Pre-OMe').

compound_t('pre-galfnac').
compound(brendan,cje,'pre-galfnac').
compound_name(brendan,'pre-galfnac','Pre-GalfNAc').

compound_t('pre-ribf').
compound(brendan,cje,'pre-ribf').
compound_name(brendan,'pre-ribf','Pre-Ribf').

compound_t('pre-glca').
compound(brendan,cje,'pre-glca').
compound_name(brendan,'pre-glca','Pre-GlcA').

compound_t('pre-ngro').
compound(brendan,cje,'pre-ngro').
compound_name(brendan,'pre-ngro','Pre-NGro').

compound_t('pre-etn').
compound(brendan,cje,'pre-etn').
compound_name(brendan,'pre-etn','Pre-Etn').

%% Adding glca or glca6ngro or glca6etn to capsule chain

subpathway_t(cap_addngro).
subpathway(brendan,cje,cap_addngro,capsule).
subpathway_name(brendan,cje,cap_addngro,'Add NGro').

subpathway_t(cap_addetn).
subpathway(brendan,cje,cap_addetn,capsule).
subpathway_name(brendan,cje,cap_addetn,'Add EtN').

reaction_t(capsule_0).
reaction_gene(brendan,cje,capsule_0,cj1441c).
guess_rtn_gene(brendan,cje,capsule_0,cj1441c).
reaction(brendan,cje,capsule_0,'capsule_chain','glca',1).
reaction(brendan,cje,capsule_0,'pre-glca','glca',1).
reaction_pathway(brendan,cje,capsule_0,capsule).

reaction_t(capsule_ngro0).
reaction_gene(brendan,cje,capsule_ngro0,unknown).
reaction(brendan,cje,capsule_ngro0,'glca','glca6ngro',1).
reaction(brendan,cje,capsule_ngro0,'pre-ngro','glca6ngro',1).
```

```
reaction_subpathway(brendan,cje,capsule_ngro0,cap_addngro).
reaction_pathway(brendan,cje,capsule_ngro0,capsule).

gene_t(cj1441c).
gene(brendan,cje,cj1441c).
gene_name(brenday,cj1441c,'KfiD').



%% for glca6ngro

%% Adding Hep and associated phase-variable modifications

subpathway_t(cap_addhep).
subpathway(brendan,cje,cap_addhep,capsule).
subpathway_name(brendan,cje,cap_addhep,'Add Hep').

reaction_t(capsule_ngro1).
reaction_gene(brendan,cje,capsule_ngro1,cj1431c).
reaction(brendan,cje,capsule_ngro1,'glca6ngro','glca6ngro+hep',1).
reaction(brendan,cje,capsule_ngro1,'gdp-d-al-gluco-hep','glca6ngro+hep',1).
reaction_subpathway(brendan,cje,capsule_ngro1,cap_addhep).
reaction_pathway(brendan,cje,capsule_ngro1,capsule).

reaction_t(capsule_ngro1a).
reaction_gene(brendan,cje,capsule_ngro1a,cj1431c).
reaction(brendan,cje,capsule_ngro1a,'glca6ngro-galfnac','glca6ngro+hep-galfnac',1).
reaction(brendan,cje,capsule_ngro1a,'gdp-d-al-gluco-hep','glca6ngro+hep-galfnac',1).
reaction_subpathway(brendan,cje,capsule_ngro1a,cap_addhep).
reaction_pathway(brendan,cje,capsule_ngro1a,capsule).

reaction_t(capsule_ngro1b).
reaction_gene(brendan,cje,capsule_ngro1b,cj1431c).
reaction(brendan,cje,capsule_ngro1b,'glca6ngro-galfnac-ribf','glca6ngro+hep-galfnac-ribf',1).
reaction(brendan,cje,capsule_ngro1b,'gdp-d-al-gluco-hep','glca6ngro+hep-galfnac-ribf',1).
reaction_subpathway(brendan,cje,capsule_ngro1b,cap_addhep).
reaction_pathway(brendan,cje,capsule_ngro1b,capsule).

reaction_t(capsule_ngro1c).
reaction_gene(brendan,cje,capsule_ngro1c,cj1431c).
reaction(brendan,cje,capsule_ngro1c,'glca6ngro-galfnac+omepn-ribf','glca6ngro+hep-galfnac+omepn-ribf',1).
reaction(brendan,cje,capsule_ngro1c,'gdp-d-al-gluco-hep','glca6ngro+hep-galfnac+omepn-ribf',1).
reaction_subpathway(brendan,cje,capsule_ngro1c,cap_addhep).
reaction_pathway(brendan,cje,capsule_ngro1c,capsule).

gene_t(cj1431c).
gene(brendan,cje,cj1431c).
gene_name(brendan,cj1431c,'HddC').

subpathway_t(cap_addhep3ome).
subpathway(brendan,cje,cap_addhep3ome,capsule).
subpathway_name(brendan,cje,cap_addhep3ome,'Add Hep 3-OMe').

subpathway_t(cap_addhep6ome).
```

```
subpathway(brendan,cje,cap_addhep6ome,capsule).
subpathway_name(brendan,cje,cap_addhep6ome,'Add Hep 6-OMe').

subpathway_t(cap_addhepomepn).
subpathway(brendan,cje,cap_addhepomepn,capsule).
subpathway_name(brendan,cje,cap_addhepomepn,'Add Hep OMePN').

%% 3-OMe
% ....


%% OMePN

reaction_t(capsule_ngro4).
reaction_gene(brendan,cje,capsule_ngro4,cj1422c).
reaction(brendan,cje,capsule_ngro4,'glca6ngro+hep','glca6ngro+hep+omepn',1).
reaction(brendan,cje,capsule_ngro4,'pre-omepn','glca6ngro+hep+omepn',1).
reaction_subpathway(brendan,cje,capsule_ngro4,cap_addhepomepn).
reaction_pathway(brendan,cje,capsule_ngro4,capsule).



reaction_t(capsule_ngro5a).
reaction_gene(brendan,cje,capsule_ngro5a,cj1422c).
reaction(brendan,cje,capsule_ngro5a,'glca6ngro+hep+6ome','glca6ngro+hep+omepn+6ome',1).
reaction(brendan,cje,capsule_ngro5a,'pre-omepn','glca6ngro+hep+omepn+6ome',1).
reaction_subpathway(brendan,cje,capsule_ngro5a,cap_addhepomepn).
reaction_pathway(brendan,cje,capsule_ngro5a,capsule).

%% 6-OMe

reaction_t(capsule_ngro5c).
reaction_gene(brendan,cje,capsule_ngro5c,cj1426c).
reaction(brendan,cje,capsule_ngro5c,'glca6ngro+hep','glca6ngro+hep+6ome',1).
reaction(brendan,cje,capsule_ngro5c,'pre-ome','glca6ngro+hep+6ome',1).
reaction_subpathway(brendan,cje,capsule_ngro5c,cap_addhep6ome).
reaction_pathway(brendan,cje,capsule_ngro5c,capsule).



reaction_t(capsule_ngro5e).
reaction_gene(brendan,cje,capsule_ngro5e,cj1426c).
reaction(brendan,cje,capsule_ngro5e,'glca6ngro+hep+omepn','glca6ngro+hep+omepn+6ome',1).
reaction(brendan,cje,capsule_ngro5e,'pre-ome','glca6ngro+hep+omepn+6ome',1).
reaction_subpathway(brendan,cje,capsule_ngro5e,cap_addhep6ome).
reaction_pathway(brendan,cje,capsule_ngro5e,capsule).



%% 3-OMe


% ...
```

%% OMePN

reaction_t(capsule_ngro8).
reaction_gene(brendan,cje,capsule_ngro8,cj1422c).
reaction(brendan,cje,capsule_ngro8,'glca6ngro+hep-galfnac','glca6ngro+hep+omepn-galfnac',1).
reaction(brendan,cje,capsule_ngro8,'pre-omepn','glca6ngro+hep+omepn-galfnac',1).
reaction_subpathway(brendan,cje,capsule_ngro8,cap_addhepomepn).
reaction_pathway(brendan,cje,capsule_ngro8,capsule).

reaction_t(capsule_ngro9a).
reaction_gene(brendan,cje,capsule_ngro9a,cj1422c).
reaction(brendan,cje,capsule_ngro9a,'glca6ngro+hep+6ome-galfnac','glca6ngro+hep+omepn+6ome-galfnac',1).
reaction(brendan,cje,capsule_ngro9a,'pre-omepn','glca6ngro+hep+omepn+6ome-galfnac',1).
reaction_subpathway(brendan,cje,capsule_ngro9a,cap_addhepomepn).
reaction_pathway(brendan,cje,capsule_ngro9a,capsule).

%% 6-OMe

reaction_t(capsule_ngro9c).
reaction_gene(brendan,cje,capsule_ngro9c,cj1426c).
reaction(brendan,cje,capsule_ngro9c,'glca6ngro+hep-galfnac','glca6ngro+hep+6ome-galfnac',1).
reaction(brendan,cje,capsule_ngro9c,'pre-ome','glca6ngro+hep+6ome-galfnac',1).
reaction_subpathway(brendan,cje,capsule_ngro9c,cap_addhep6ome).
reaction_pathway(brendan,cje,capsule_ngro9c,capsule).

reaction_t(capsule_ngro9e).
reaction_gene(brendan,cje,capsule_ngro9e,cj1426c).
reaction(brendan,cje,capsule_ngro9e,'glca6ngro+hep+omepn-galfnac','glca6ngro+hep+omepn+6ome-galfnac',1).
reaction(brendan,cje,capsule_ngro9e,'pre-ome','glca6ngro+hep+omepn+6ome-galfnac',1).
reaction_subpathway(brendan,cje,capsule_ngro9e,cap_addhep6ome).
reaction_pathway(brendan,cje,capsule_ngro9e,capsule).

%% 3-OMe

%% OMePN

reaction_t(capsule_ngro12).
reaction_gene(brendan,cje,capsule_ngro12,cj1422c).
reaction(brendan,cje,capsule_ngro12,'glca6ngro+hep-galfnac+omepn','glca6ngro+hep+omepn-galfnac+omepn',1).
reaction(brendan,cje,capsule_ngro12,'pre-omepn','glca6ngro+hep+omepn-galfnac+omepn',1).
reaction_subpathway(brendan,cje,capsule_ngro12,cap_addhepomepn).
reaction_pathway(brendan,cje,capsule_ngro12,capsule).

reaction_t(capsule_ngro13a).
reaction_gene(brendan,cje,capsule_ngro13a,cj1422c).
reaction(brendan,cje,capsule_ngro13a,'glca6ngro+hep+6ome-galfnac+omepn','glca6ngro+hep+omepn+6ome-galfnac+omepn',1).
reaction(brendan,cje,capsule_ngro13a,'pre-omepn','glca6ngro+hep+omepn+6ome-galfnac+omepn',1).
reaction_subpathway(brendan,cje,capsule_ngro13a,cap_addhepomepn).
reaction_pathway(brendan,cje,capsule_ngro13a,capsule).

%% 6-OMe

reaction_t(capsule_ngro13c).
reaction_gene(brendan,cje,capsule_ngro13c,cj1426c).
reaction(brendan,cje,capsule_ngro13c,'glca6ngro+hep-galfnac+omepn','glca6ngro+hep+6ome-galfnac+omepn',1).
reaction(brendan,cje,capsule_ngro13c,'pre-ome','glca6ngro+hep+6ome-galfnac+omepn',1).
reaction_subpathway(brendan,cje,capsule_ngro13c,cap_addhep6ome).
reaction_pathway(brendan,cje,capsule_ngro13c,capsule).

reaction_t(capsule_ngro13e).
reaction_gene(brendan,cje,capsule_ngro13e,cj1426c).
reaction(brendan,cje,capsule_ngro13e,'glca6ngro+hep+omepn-galfnac+omepn','glca6ngro+hep+omepn+6ome-galfnac+omepn',1).
reaction(brendan,cje,capsule_ngro13e,'pre-ome','glca6ngro+hep+omepn+6ome-galfnac+omepn',1).
reaction_subpathway(brendan,cje,capsule_ngro13e,cap_addhep6ome).
reaction_pathway(brendan,cje,capsule_ngro13e,capsule).

%% 3-OMe

%% OMePN

reaction_t(capsule_ngro16).
reaction_gene(brendan,cje,capsule_ngro16,cj1422c).
reaction(brendan,cje,capsule_ngro16,'glca6ngro+hep-galfnac-ribf','glca6ngro+hep+omepn-galfnac-ribf',1).
reaction(brendan,cje,capsule_ngro16,'pre-omepn','glca6ngro+hep+omepn-galfnac-ribf',1).
reaction_subpathway(brendan,cje,capsule_ngro16,cap_addhepomepn).
reaction_pathway(brendan,cje,capsule_ngro16,capsule).

reaction_t(capsule_ngro17a).
reaction_gene(brendan,cje,capsule_ngro17a,cj1422c).
reaction(brendan,cje,capsule_ngro17a,'glca6ngro+hep+6ome-galfnac-ribf','glca6ngro+hep+omepn+6ome-galfnac-ribf',1).
reaction(brendan,cje,capsule_ngro17a,'pre-omepn','glca6ngro+hep+omepn+6ome-galfnac-ribf',1).
reaction_subpathway(brendan,cje,capsule_ngro17a,cap_addhepomepn).
reaction_pathway(brendan,cje,capsule_ngro17a,capsule).

%% 6-OMe

reaction_t(capsule_ngro17c).
reaction_gene(brendan,cje,capsule_ngro17c,cj1426c).
reaction(brendan,cje,capsule_ngro17c,'glca6ngro+hep-galfnac-ribf','glca6ngro+hep+6ome-galfnac-ribf',1).
reaction(brendan,cje,capsule_ngro17c,'pre-ome','glca6ngro+hep+6ome-galfnac-ribf',1).
reaction_subpathway(brendan,cje,capsule_ngro17c,cap_addhep6ome).
reaction_pathway(brendan,cje,capsule_ngro17c,capsule).

reaction_t(capsule_ngro17e).
reaction_gene(brendan,cje,capsule_ngro17e,cj1426c).
reaction(brendan,cje,capsule_ngro17e,'glca6ngro+hep+omepn-galfnac-ribf','glca6ngro+hep+omepn+6ome-galfnac-ribf',1).
reaction(brendan,cje,capsule_ngro17e,'pre-ome','glca6ngro+hep+omepn+6ome-galfnac-ribf',1).
reaction_subpathway(brendan,cje,capsule_ngro17e,cap_addhep6ome).

reaction_pathway(brendan,cje,capsule_ngro17e,capsule).

%% 3-OMe

%% OMePN

reaction_t(capsule_ngro20).
reaction_gene(brendan,cje,capsule_ngro20,cj1422c).
reaction(brendan,cje,capsule_ngro20,'glca6ngro+hep-galfnac+omepn-ribf','glca6ngro+hep+omepn-galfnac+omepn-ribf',1).
reaction(brendan,cje,capsule_ngro20,'pre-omepn','glca6ngro+hep+omepn-galfnac+omepn-ribf',1).
reaction_subpathway(brendan,cje,capsule_ngro20,cap_addhepomepn).
reaction_pathway(brendan,cje,capsule_ngro20,capsule).

reaction_t(capsule_ngro21a).
reaction_gene(brendan,cje,capsule_ngro21a,cj1422c).
reaction(brendan,cje,capsule_ngro21a,'glca6ngro+hep+6ome-galfnac+omepn-ribf','glca6ngro+hep+omepn+6ome-galfnac+omepn-ribf',1).
reaction(brendan,cje,capsule_ngro21a,'pre-omepn','glca6ngro+hep+omepn+6ome-galfnac+omepn-ribf',1).
reaction_subpathway(brendan,cje,capsule_ngro21a,cap_addhepomepn).
reaction_pathway(brendan,cje,capsule_ngro21a,capsule).

%% 6-OMe

reaction_t(capsule_ngro21c).
reaction_gene(brendan,cje,capsule_ngro21c,cj1426c).
reaction(brendan,cje,capsule_ngro21c,'glca6ngro+hep-galfnac+omepn-ribf','glca6ngro+hep+6ome-galfnac+omepn-ribf',1).
reaction(brendan,cje,capsule_ngro21c,'pre-ome','glca6ngro+hep+6ome-galfnac+omepn-ribf',1).
reaction_subpathway(brendan,cje,capsule_ngro21c,cap_addhep6ome).
reaction_pathway(brendan,cje,capsule_ngro21c,capsule).

reaction_t(capsule_ngro21e).
reaction_gene(brendan,cje,capsule_ngro21e,cj1426c).
reaction(brendan,cje,capsule_ngro21e,'glca6ngro+hep+omepn-galfnac+omepn-ribf','glca6ngro+hep+omepn+6ome-galfnac+omepn-ribf',1).
reaction(brendan,cje,capsule_ngro21e,'pre-ome','glca6ngro+hep+omepn+6ome-galfnac+omepn-ribf',1).
reaction_subpathway(brendan,cje,capsule_ngro21e,cap_addhep6ome).
reaction_pathway(brendan,cje,capsule_ngro21e,capsule).

gene_t(cj1422c).
gene(brendan,cje,cj1422c).
gene_name(brendan,cj1422c,'putative sugar transferase').

gene_t(cj1426c).
gene(brendan,cje,cj1426c).
gene_name(brendan,cj1426c,'putative methyltransferase family protein').

gene_t(unknown).

```
gene(brendan,cje,unknown).
gene_name(brendan,unknown,'Unknown gene or spontaneous reaction').


%% Adding galfnac and associated phase-variable modification

subpathway_t(cap_addgalf).
subpathway(brendan,cje,cap_addgalf,capsule).
subpathway_name(brendan,cje,cap_addgalf,'Add Galfnac').

reaction_t(capsule_ngro22).
reaction_gene(brendan,cje,capsule_ngro22,cj1439c).
guess_rtn_gene(brendan,cje,capsule_ngro22,cj1439c).
reaction(brendan,cje,capsule_ngro22,'glca6ngro','glca6ngro-galfnac',1).
reaction(brendan,cje,capsule_ngro22,'pre-galfnac','glca6ngro-galfnac',1).
reaction_subpathway(brendan,cje,capsule_ngro22,cap_addgalf).
reaction_pathway(brendan,cje,capsule_ngro22,capsule).

reaction_t(capsule_ngro23).
reaction_gene(brendan,cje,capsule_ngro23,cj1439c).
guess_rtn_gene(brendan,cje,capsule_ngro23,cj1439c).
reaction(brendan,cje,capsule_ngro23,'glca6ngro+hep','glca6ngro+hep-galfnac',1).
reaction(brendan,cje,capsule_ngro23,'pre-galfnac','glca6ngro+hep-galfnac',1).
reaction_subpathway(brendan,cje,capsule_ngro23,cap_addgalf).
reaction_pathway(brendan,cje,capsule_ngro23,capsule).

reaction_t(capsule_ngro24a).
reaction_gene(brendan,cje,capsule_ngro24a,cj1439c).
guess_rtn_gene(brendan,cje,capsule_ngro24a,cj1439c).
reaction(brendan,cje,capsule_ngro24a,'glca6ngro+hep+6ome','glca6ngro+hep+6ome-galfnac',1).
reaction(brendan,cje,capsule_ngro24a,'pre-galfnac','glca6ngro+hep+6ome-galfnac',1).
reaction_subpathway(brendan,cje,capsule_ngro24a,cap_addgalf).
reaction_pathway(brendan,cje,capsule_ngro24a,capsule).

reaction_t(capsule_ngro25).
reaction_gene(brendan,cje,capsule_ngro25,cj1439c).
guess_rtn_gene(brendan,cje,capsule_ngro25,cj1439c).
reaction(brendan,cje,capsule_ngro25,'glca6ngro+hep+omepn','glca6ngro+hep+omepn-galfnac',1).
reaction(brendan,cje,capsule_ngro25,'pre-galfnac','glca6ngro+hep+omepn-galfnac',1).
reaction_subpathway(brendan,cje,capsule_ngro25,cap_addgalf).
reaction_pathway(brendan,cje,capsule_ngro25,capsule).

reaction_t(capsule_ngro26a).
reaction_gene(brendan,cje,capsule_ngro26a,cj1439c).
guess_rtn_gene(brendan,cje,capsule_ngro26a,cj1439c).
reaction(brendan,cje,capsule_ngro26a,'glca6ngro+hep+omepn+6ome','glca6ngro+hep+omepn+6ome-galfnac',1).
reaction(brendan,cje,capsule_ngro26a,'pre-galfnac','glca6ngro+hep+omepn+6ome-galfnac',1).
reaction_subpathway(brendan,cje,capsule_ngro26a,cap_addgalf).
reaction_pathway(brendan,cje,capsule_ngro26a,capsule).


gene_t(cj1439c).
gene(brendan,cje,cj1439c).
gene_name(brendan,cj1439c,'Glf').
```

subpathway_t(cap_addgalfomepn).
subpathway(brendan,cje,cap_addgalfomepn,capsule).
subpathway_name(brendan,cje,cap_addgalfomepn,'Add Galfnac OMePN').

reaction_t(capsule_ngro27).
reaction_gene(brendan,cje,capsule_ngro27,cj1421c).
reaction(brendan,cje,capsule_ngro27,'glca6ngro-galfnac','glca6ngro-galfnac+omepn',1).
reaction(brendan,cje,capsule_ngro27,'pre-omepn','glca6ngro-galfnac+omepn',1).
reaction_subpathway(brendan,cje,capsule_ngro27,cap_addgalfomepn).
reaction_pathway(brendan,cje,capsule_ngro27,capsule).

reaction_t(capsule_ngro28).
reaction_gene(brendan,cje,capsule_ngro28,cj1421c).
reaction(brendan,cje,capsule_ngro28,'glca6ngro+hep-galfnac','glca6ngro+hep-galfnac+omepn',1).
reaction(brendan,cje,capsule_ngro28,'pre-omepn','glca6ngro+hep-galfnac+omepn',1).
reaction_subpathway(brendan,cje,capsule_ngro28,cap_addgalfomepn).
reaction_pathway(brendan,cje,capsule_ngro28,capsule).

reaction_t(capsule_ngro29a).
reaction_gene(brendan,cje,capsule_ngro29a,cj1421c).
reaction(brendan,cje,capsule_ngro29a,'glca6ngro+hep+6ome-galfnac','glca6ngro+hep+6ome-galfnac+omepn',1).
reaction(brendan,cje,capsule_ngro29a,'pre-omepn','glca6ngro+hep+6ome-galfnac+omepn',1).
reaction_subpathway(brendan,cje,capsule_ngro29a,cap_addgalfomepn).
reaction_pathway(brendan,cje,capsule_ngro29a,capsule).

reaction_t(capsule_ngro30).
reaction_gene(brendan,cje,capsule_ngro30,cj1421c).
reaction(brendan,cje,capsule_ngro30,'glca6ngro+hep+omepn-galfnac','glca6ngro+hep+omepn-galfnac+omepn',1).
reaction(brendan,cje,capsule_ngro30,'pre-omepn','glca6ngro+hep+omepn-galfnac+omepn',1).
reaction_subpathway(brendan,cje,capsule_ngro30,cap_addgalfomepn).
reaction_pathway(brendan,cje,capsule_ngro30,capsule).

reaction_t(capsule_ngro31a).
reaction_gene(brendan,cje,capsule_ngro31a,cj1421c).
reaction(brendan,cje,capsule_ngro31a,'glca6ngro+hep+omepn+6ome-galfnac','glca6ngro+hep+omepn+6ome-galfnac+omepn',1).
reaction(brendan,cje,capsule_ngro31a,'pre-omepn','glca6ngro+hep+omepn+6ome-galfnac+omepn',1).
reaction_subpathway(brendan,cje,capsule_ngro31a,cap_addgalfomepn).
reaction_pathway(brendan,cje,capsule_ngro31a,capsule).

reaction_t(capsule_ngro32).
reaction_gene(brendan,cje,capsule_ngro32,cj1421c).
reaction(brendan,cje,capsule_ngro32,'glca6ngro-galfnac-ribf','glca6ngro-galfnac+omepn-ribf',1).
reaction(brendan,cje,capsule_ngro32,'pre-omepn','glca6ngro-galfnac+omepn-ribf',1).
reaction_subpathway(brendan,cje,capsule_ngro32,cap_addgalfomepn).
reaction_pathway(brendan,cje,capsule_ngro32,capsule).

reaction_t(capsule_ngro33).
reaction_gene(brendan,cje,capsule_ngro33,cj1421c).
reaction(brendan,cje,capsule_ngro33,'glca6ngro+hep-galfnac-ribf','glca6ngro+hep-galfnac+omepn-ribf',1).
reaction(brendan,cje,capsule_ngro33,'pre-omepn','glca6ngro+hep-galfnac+omepn-ribf',1).
reaction_subpathway(brendan,cje,capsule_ngro33,cap_addgalfomepn).

```
reaction_pathway(brendan,cje,capsule_ngro33,capsule).

reaction_t(capsule_ngro34a).
reaction_gene(brendan,cje,capsule_ngro34a,cj1421c).
reaction(brendan,cje,capsule_ngro34a,'glca6ngro+hep+6ome-galfnac-ribf','glca6ngro+hep+6ome-galfnac+omepn-
ribf',1).
reaction(brendan,cje,capsule_ngro34a,'pre-omepn','glca6ngro+hep+6ome-galfnac+omepn-ribf',1).
reaction_subpathway(brendan,cje,capsule_ngro34a,cap_addgalfomepn).
reaction_pathway(brendan,cje,capsule_ngro34a,capsule).

reaction_t(capsule_ngro35).
reaction_gene(brendan,cje,capsule_ngro35,cj1421c).
reaction(brendan,cje,capsule_ngro35,'glca6ngro+hep+omepn-galfnac-ribf','glca6ngro+hep+omepn-galfnac+omepn-
ribf',1).
reaction(brendan,cje,capsule_ngro35,'pre-omepn','glca6ngro+hep+omepn-galfnac+omepn-ribf',1).
reaction_subpathway(brendan,cje,capsule_ngro35,cap_addgalfomepn).
reaction_pathway(brendan,cje,capsule_ngro35,capsule).

reaction_t(capsule_ngro36a).
reaction_gene(brendan,cje,capsule_ngro36a,cj1421c).
reaction(brendan,cje,capsule_ngro36a,'glca6ngro+hep+omepn+6ome-galfnac-ribf','glca6ngro+hep+omepn+6ome-
galfnac+omepn-ribf',1).
reaction(brendan,cje,capsule_ngro36a,'pre-omepn','glca6ngro+hep+omepn+6ome-galfnac+omepn-ribf',1).
reaction_subpathway(brendan,cje,capsule_ngro36a,cap_addgalfomepn).
reaction_pathway(brendan,cje,capsule_ngro36a,capsule).

gene_t(cj1421c).
gene(brendan,cje,cj1421c).
gene_name(brendan,cj1421c,'putative sugar transferase').

%% Adding ribf

subpathway_t(cap_addribf).
subpathway(brendan,cje,cap_addribf,capsule).
subpathway_name(brendan,cje,cap_addribf,'Add Ribf').

reaction_t(capsule_ngro37).
reaction_gene(brendan,cje,capsule_ngro37,unknown).
reaction(brendan,cje,capsule_ngro37,'glca6ngro-galfnac','glca6ngro-galfnac-ribf',1).
reaction(brendan,cje,capsule_ngro37,'pre-ribf','glca6ngro-galfnac-ribf',1).
reaction_subpathway(brendan,cje,capsule_ngro37,cap_addribf).
reaction_pathway(brendan,cje,capsule_ngro37,capsule).

reaction_t(capsule_ngro38).
reaction_gene(brendan,cje,capsule_ngro38,unknown).
reaction(brendan,cje,capsule_ngro38,'glca6ngro+hep-galfnac','glca6ngro+hep-galfnac-ribf',1).
reaction(brendan,cje,capsule_ngro38,'pre-ribf','glca6ngro+hep-galfnac-ribf',1).
reaction_subpathway(brendan,cje,capsule_ngro38,cap_addribf).
reaction_pathway(brendan,cje,capsule_ngro38,capsule).

reaction_t(capsule_ngro39a).
reaction_gene(brendan,cje,capsule_ngro39a,unknown).
reaction(brendan,cje,capsule_ngro39a,'glca6ngro+hep+6ome-galfnac','glca6ngro+hep+6ome-galfnac-ribf',1).
reaction(brendan,cje,capsule_ngro39a,'pre-ribf','glca6ngro+hep+6ome-galfnac-ribf',1).
```

reaction_subpathway(brendan,cje,capsule_ngro39a,cap_addribf).
reaction_pathway(brendan,cje,capsule_ngro39a,capsule).

reaction_t(capsule_ngro40).
reaction_gene(brendan,cje,capsule_ngro40,unknown).
reaction(brendan,cje,capsule_ngro40,'glca6ngro+hep+omepn-galfnac','glca6ngro+hep+omepn-galfnac-ribf',1).
reaction(brendan,cje,capsule_ngro40,'pre-ribf','glca6ngro+hep+omepn-galfnac-ribf',1).
reaction_subpathway(brendan,cje,capsule_ngro40,cap_addribf).
reaction_pathway(brendan,cje,capsule_ngro40,capsule).

reaction_t(capsule_ngro41a).
reaction_gene(brendan,cje,capsule_ngro41a,unknown).
reaction(brendan,cje,capsule_ngro41a,'glca6ngro+hep+omepn+6ome-galfnac','glca6ngro+hep+omepn+6ome-galfnac-ribf',1).
reaction(brendan,cje,capsule_ngro41a,'pre-ribf','glca6ngro+hep+omepn+6ome-galfnac-ribf',1).
reaction_subpathway(brendan,cje,capsule_ngro41a,cap_addribf).
reaction_pathway(brendan,cje,capsule_ngro41a,capsule).

reaction_t(capsule_ngro42).
reaction_gene(brendan,cje,capsule_ngro42,unknown).
reaction(brendan,cje,capsule_ngro42,'glca6ngro-galfnac+omepn','glca6ngro-galfnac+omepn-ribf',1).
reaction(brendan,cje,capsule_ngro42,'pre-ribf','glca6ngro-galfnac+omepn-ribf',1).
reaction_subpathway(brendan,cje,capsule_ngro42,cap_addribf).
reaction_pathway(brendan,cje,capsule_ngro42,capsule).

reaction_t(capsule_ngro43).
reaction_gene(brendan,cje,capsule_ngro43,unknown).
reaction(brendan,cje,capsule_ngro43,'glca6ngro+hep-galfnac+omepn','glca6ngro+hep-galfnac+omepn-ribf',1).
reaction(brendan,cje,capsule_ngro43,'pre-ribf','glca6ngro+hep-galfnac+omepn-ribf',1).
reaction_subpathway(brendan,cje,capsule_ngro43,cap_addribf).
reaction_pathway(brendan,cje,capsule_ngro43,capsule).

reaction_t(capsule_ngro44a).
reaction_gene(brendan,cje,capsule_ngro44a,unknown).
reaction(brendan,cje,capsule_ngro44a,'glca6ngro+hep+6ome-galfnac+omepn','glca6ngro+hep+6ome-galfnac+omepn-ribf',1).
reaction(brendan,cje,capsule_ngro44a,'pre-ribf','glca6ngro+hep+6ome-galfnac+omepn-ribf',1).
reaction_subpathway(brendan,cje,capsule_ngro44a,cap_addribf).
reaction_pathway(brendan,cje,capsule_ngro44a,capsule).

reaction_t(capsule_ngro45).
reaction_gene(brendan,cje,capsule_ngro45,unknown).
reaction(brendan,cje,capsule_ngro45,'glca6ngro+hep+omepn-galfnac+omepn','glca6ngro+hep+omepn-galfnac+omepn-ribf',1).
reaction(brendan,cje,capsule_ngro45,'pre-ribf','glca6ngro+hep+omepn-galfnac+omepn-ribf',1).
reaction_subpathway(brendan,cje,capsule_ngro45,cap_addribf).
reaction_pathway(brendan,cje,capsule_ngro45,capsule).

reaction_t(capsule_ngro46a).
reaction_gene(brendan,cje,capsule_ngro46a,unknown).
reaction(brendan,cje,capsule_ngro46a,'glca6ngro+hep+omepn+6ome-galfnac+omepn','glca6ngro+hep+omepn+6ome-galfnac+omepn-ribf',1).
reaction(brendan,cje,capsule_ngro46a,'pre-ribf','glca6ngro+hep+omepn+6ome-galfnac+omepn-ribf',1).
reaction_subpathway(brendan,cje,capsule_ngro46a,cap_addribf).

```
reaction_pathway(brendan,cje,capsule_ngro46a,capsule).

%% now for glca6etn

% ...........

%% now for glca

%% Adding Hep and associated phase-variable modifications

reaction_t(capsule_1).
reaction_gene(brendan,cje,capsule_1,cj1431c).
reaction(brendan,cje,capsule_1,'glca','glca+hep',1).
reaction(brendan,cje,capsule_1,'gdp-d-al-gluco-hep','glca+hep',1).
reaction_subpathway(brendan,cje,capsule_1,cap_addhep).
reaction_pathway(brendan,cje,capsule_1,capsule).

reaction_t(capsule_1a).
reaction_gene(brendan,cje,capsule_1a,cj1431c).
reaction(brendan,cje,capsule_1a,'glca-galfnac','glca+hep-galfnac',1).
reaction(brendan,cje,capsule_1a,'gdp-d-al-gluco-hep','glca+hep-galfnac',1).
reaction_subpathway(brendan,cje,capsule_1a,cap_addhep).
reaction_pathway(brendan,cje,capsule_1a,capsule).

reaction_t(capsule_1b).
reaction_gene(brendan,cje,capsule_1b,cj1431c).
reaction(brendan,cje,capsule_1b,'glca-galfnac-ribf','glca+hep-galfnac-ribf',1).
reaction(brendan,cje,capsule_1b,'gdp-d-al-gluco-hep','glca+hep-galfnac-ribf',1).
reaction_subpathway(brendan,cje,capsule_1b,cap_addhep).
reaction_pathway(brendan,cje,capsule_1b,capsule).

reaction_t(capsule_1c).
reaction_gene(brendan,cje,capsule_1c,cj1431c).
reaction(brendan,cje,capsule_1c,'glca-galfnac+omepn-ribf','glca+hep-galfnac+omepn-ribf',1).
reaction(brendan,cje,capsule_1c,'gdp-d-al-gluco-hep','glca+hep-galfnac+omepn-ribf',1).
reaction_subpathway(brendan,cje,capsule_1c,cap_addhep).
reaction_pathway(brendan,cje,capsule_1c,capsule).

%% 3-OMe


%% OMePN

reaction_t(capsule_4).
reaction_gene(brendan,cje,capsule_4,cj1422c).
reaction(brendan,cje,capsule_4,'glca+hep','glca+hep+omepn',1).
reaction(brendan,cje,capsule_4,'pre-omepn','glca+hep+omepn',1).
reaction_subpathway(brendan,cje,capsule_4,cap_addhepomepn).
reaction_pathway(brendan,cje,capsule_4,capsule).

reaction_t(capsule_5a).
reaction_gene(brendan,cje,capsule_5a,cj1422c).
reaction(brendan,cje,capsule_5a,'glca+hep+6ome','glca+hep+omepn+6ome',1).
reaction(brendan,cje,capsule_5a,'pre-omepn','glca+hep+omepn+6ome',1).
```

```
reaction_subpathway(brendan,cje,capsule_5a,cap_addhepomepn).
reaction_pathway(brendan,cje,capsule_5a,capsule).

%% 6-OMe

reaction_t(capsule_5c).
reaction_gene(brendan,cje,capsule_5c,cj1426c).
reaction(brendan,cje,capsule_5c,'glca+hep','glca+hep+6ome',1).
reaction(brendan,cje,capsule_5c,'pre-ome','glca+hep+6ome',1).
reaction_subpathway(brendan,cje,capsule_5c,cap_addhep6ome).
reaction_pathway(brendan,cje,capsule_5c,capsule).

reaction_t(capsule_5e).
reaction_gene(brendan,cje,capsule_5e,cj1426c).
reaction(brendan,cje,capsule_5e,'glca+hep+omepn','glca+hep+omepn+6ome',1).
reaction(brendan,cje,capsule_5e,'pre-ome','glca+hep+omepn+6ome',1).
reaction_subpathway(brendan,cje,capsule_5e,cap_addhep6ome).
reaction_pathway(brendan,cje,capsule_5e,capsule).

%% 3-OMe

%% OMePN

reaction_t(capsule_8).
reaction_gene(brendan,cje,capsule_8,cj1422c).
reaction(brendan,cje,capsule_8,'glca+hep-galfnac','glca+hep+omepn-galfnac',1).
reaction(brendan,cje,capsule_8,'pre-omepn','glca+hep+omepn-galfnac',1).
reaction_subpathway(brendan,cje,capsule_8,cap_addhepomepn).
reaction_pathway(brendan,cje,capsule_8,capsule).

reaction_t(capsule_9a).
reaction_gene(brendan,cje,capsule_9a,cj1422c).
reaction(brendan,cje,capsule_9a,'glca+hep+6ome-galfnac','glca+hep+omepn+6ome-galfnac',1).
reaction(brendan,cje,capsule_9a,'pre-omepn','glca+hep+omepn+6ome-galfnac',1).
reaction_subpathway(brendan,cje,capsule_9a,cap_addhepomepn).
reaction_pathway(brendan,cje,capsule_9a,capsule).

%% 6-OMe

reaction_t(capsule_9c).
reaction_gene(brendan,cje,capsule_9c,cj1426c).
reaction(brendan,cje,capsule_9c,'glca+hep-galfnac','glca+hep+6ome-galfnac',1).
reaction(brendan,cje,capsule_9c,'pre-ome','glca+hep+6ome-galfnac',1).
reaction_subpathway(brendan,cje,capsule_9c,cap_addhep6ome).
reaction_pathway(brendan,cje,capsule_9c,capsule).

reaction_t(capsule_9e).
reaction_gene(brendan,cje,capsule_9e,cj1426c).
reaction(brendan,cje,capsule_9e,'glca+hep+omepn-galfnac','glca+hep+omepn+6ome-galfnac',1).
reaction(brendan,cje,capsule_9e,'pre-ome','glca+hep+omepn+6ome-galfnac',1).
reaction_subpathway(brendan,cje,capsule_9e,cap_addhep6ome).
reaction_pathway(brendan,cje,capsule_9e,capsule).

%% 3-OMe
```

```
%% OMePN

reaction_t(capsule_12).
reaction_gene(brendan,cje,capsule_12,cj1422c).
reaction(brendan,cje,capsule_12,'glca+hep-galfnac+omepn','glca+hep+omepn-galfnac+omepn',1).
reaction(brendan,cje,capsule_12,'pre-omepn','glca+hep+omepn-galfnac+omepn',1).
reaction_subpathway(brendan,cje,capsule_12,cap_addhepomepn).
reaction_pathway(brendan,cje,capsule_12,capsule).

reaction_t(capsule_13a).
reaction_gene(brendan,cje,capsule_13a,cj1422c).
reaction(brendan,cje,capsule_13a,'glca+hep+6ome-galfnac+omepn','glca+hep+omepn+6ome-galfnac+omepn',1).
reaction(brendan,cje,capsule_13a,'pre-omepn','glca+hep+omepn+6ome-galfnac+omepn',1).
reaction_subpathway(brendan,cje,capsule_13a,cap_addhepomepn).
reaction_pathway(brendan,cje,capsule_13a,capsule).

%% 6-OMe

reaction_t(capsule_13c).
reaction_gene(brendan,cje,capsule_13c,cj1426c).
reaction(brendan,cje,capsule_13c,'glca+hep-galfnac+omepn','glca+hep+6ome-galfnac+omepn',1).
reaction(brendan,cje,capsule_13c,'pre-ome','glca+hep+6ome-galfnac+omepn',1).
reaction_subpathway(brendan,cje,capsule_13c,cap_addhep6ome).
reaction_pathway(brendan,cje,capsule_13c,capsule).

reaction_t(capsule_13e).
reaction_gene(brendan,cje,capsule_13e,cj1426c).
reaction(brendan,cje,capsule_13e,'glca+hep+omepn-galfnac+omepn','glca+hep+omepn+6ome-galfnac+omepn',1).
reaction(brendan,cje,capsule_13e,'pre-ome','glca+hep+omepn+6ome-galfnac+omepn',1).
reaction_subpathway(brendan,cje,capsule_13e,cap_addhep6ome).
reaction_pathway(brendan,cje,capsule_13e,capsule).

%% 3-OMe

%% OMePN

reaction_t(capsule_16).
reaction_gene(brendan,cje,capsule_16,cj1422c).
reaction(brendan,cje,capsule_16,'glca+hep-galfnac-ribf','glca+hep+omepn-galfnac-ribf',1).
reaction(brendan,cje,capsule_16,'pre-omepn','glca+hep+omepn-galfnac-ribf',1).
reaction_subpathway(brendan,cje,capsule_16,cap_addhepomepn).
reaction_pathway(brendan,cje,capsule_16,capsule).

reaction_t(capsule_17a).
reaction_gene(brendan,cje,capsule_17a,cj1422c).
reaction(brendan,cje,capsule_17a,'glca+hep+6ome-galfnac-ribf','glca+hep+omepn+6ome-galfnac-ribf',1).
reaction(brendan,cje,capsule_17a,'pre-omepn','glca+hep+omepn+6ome-galfnac-ribf',1).
reaction_subpathway(brendan,cje,capsule_17a,cap_addhepomepn).
reaction_pathway(brendan,cje,capsule_17a,capsule).

%% 6-OMe

reaction_t(capsule_17c).
```

```
reaction_gene(brendan,cje,capsule_17c,cj1426c).
reaction(brendan,cje,capsule_17c,'glca+hep-galfnac-ribf','glca+hep+6ome-galfnac-ribf',1).
reaction(brendan,cje,capsule_17c,'pre-ome','glca+hep+6ome-galfnac-ribf',1).
reaction_subpathway(brendan,cje,capsule_17c,cap_addhep6ome).
reaction_pathway(brendan,cje,capsule_17c,capsule).

reaction_t(capsule_17e).
reaction_gene(brendan,cje,capsule_17e,cj1426c).
reaction(brendan,cje,capsule_17e,'glca+hep+omepn-galfnac-ribf','glca+hep+omepn+6ome-galfnac-ribf',1).
reaction(brendan,cje,capsule_17e,'pre-ome','glca+hep+omepn+6ome-galfnac-ribf',1).
reaction_subpathway(brendan,cje,capsule_17e,cap_addhep6ome).
reaction_pathway(brendan,cje,capsule_17e,capsule).

%% 3-OMe

%% OMePN

reaction_t(capsule_20).
reaction_gene(brendan,cje,capsule_20,cj1422c).
reaction(brendan,cje,capsule_20,'glca+hep-galfnac+omepn-ribf','glca+hep+omepn-galfnac+omepn-ribf',1).
reaction(brendan,cje,capsule_20,'pre-omepn','glca+hep+omepn-galfnac+omepn-ribf',1).
reaction_subpathway(brendan,cje,capsule_20,cap_addhepomepn).
reaction_pathway(brendan,cje,capsule_20,capsule).

reaction_t(capsule_21a).
reaction_gene(brendan,cje,capsule_21a,cj1422c).
reaction(brendan,cje,capsule_21a,'glca+hep+6ome-galfnac+omepn-ribf','glca+hep+omepn+6ome-galfnac+omepn-
ribf',1).
reaction(brendan,cje,capsule_21a,'pre-omepn','glca+hep+omepn+6ome-galfnac+omepn-ribf',1).
reaction_subpathway(brendan,cje,capsule_21a,cap_addhepomepn).
reaction_pathway(brendan,cje,capsule_21a,capsule).

%% 6-OMe

reaction_t(capsule_21c).
reaction_gene(brendan,cje,capsule_21c,cj1426c).
reaction(brendan,cje,capsule_21c,'glca+hep-galfnac+omepn-ribf','glca+hep+6ome-galfnac+omepn-ribf',1).
reaction(brendan,cje,capsule_21c,'pre-ome','glca+hep+6ome-galfnac+omepn-ribf',1).
reaction_subpathway(brendan,cje,capsule_21c,cap_addhep6ome).
reaction_pathway(brendan,cje,capsule_21c,capsule).

reaction_t(capsule_21e).
reaction_gene(brendan,cje,capsule_21e,cj1426c).
reaction(brendan,cje,capsule_21e,'glca+hep+omepn-galfnac+omepn-ribf','glca+hep+omepn+6ome-galfnac+omepn-
ribf',1).
reaction(brendan,cje,capsule_21e,'pre-ome','glca+hep+omepn+6ome-galfnac+omepn-ribf',1).
reaction_subpathway(brendan,cje,capsule_21e,cap_addhep6ome).
reaction_pathway(brendan,cje,capsule_21e,capsule).
```

```
%% Adding galfnac and associated phase-variable modification

reaction_t(capsule_22).
reaction_gene(brendan,cje,capsule_22,cj1439c).
guess_rtn_gene(brendan,cje,capsule_22,cj1439c).
reaction(brendan,cje,capsule_22,'glca','glca-galfnac',1).
reaction(brendan,cje,capsule_22,'pre-galfnac','glca-galfnac',1).
reaction_subpathway(brendan,cje,capsule_22,cap_addgalf).
reaction_pathway(brendan,cje,capsule_22,capsule).

reaction_t(capsule_23).
reaction_gene(brendan,cje,capsule_23,cj1439c).
guess_rtn_gene(brendan,cje,capsule_23,cj1439c).
reaction(brendan,cje,capsule_23,'glca+hep','glca+hep-galfnac',1).
reaction(brendan,cje,capsule_23,'pre-galfnac','glca+hep-galfnac',1).
reaction_subpathway(brendan,cje,capsule_23,cap_addgalf).
reaction_pathway(brendan,cje,capsule_23,capsule).

reaction_t(capsule_24a).
reaction_gene(brendan,cje,capsule_24a,cj1439c).
guess_rtn_gene(brendan,cje,capsule_24a,cj1439c).
reaction(brendan,cje,capsule_24a,'glca+hep+6ome','glca+hep+6ome-galfnac',1).
reaction(brendan,cje,capsule_24a,'pre-galfnac','glca+hep+6ome-galfnac',1).
reaction_subpathway(brendan,cje,capsule_24a,cap_addgalf).
reaction_pathway(brendan,cje,capsule_24a,capsule).

reaction_t(capsule_25).
reaction_gene(brendan,cje,capsule_25,cj1439c).
guess_rtn_gene(brendan,cje,capsule_25,cj1439c).
reaction(brendan,cje,capsule_25,'glca+hep+omepn','glca+hep+omepn-galfnac',1).
reaction(brendan,cje,capsule_25,'pre-galfnac','glca+hep+omepn-galfnac',1).
reaction_subpathway(brendan,cje,capsule_25,cap_addgalf).
reaction_pathway(brendan,cje,capsule_25,capsule).

reaction_t(capsule_26a).
reaction_gene(brendan,cje,capsule_26a,cj1439c).
guess_rtn_gene(brendan,cje,capsule_26a,cj1439c).
reaction(brendan,cje,capsule_26a,'glca+hep+omepn+6ome','glca+hep+omepn+6ome-galfnac',1).
reaction(brendan,cje,capsule_26a,'pre-galfnac','glca+hep+omepn+6ome-galfnac',1).
reaction_subpathway(brendan,cje,capsule_26a,cap_addgalf).
reaction_pathway(brendan,cje,capsule_26a,capsule).


reaction_t(capsule_27).
reaction_gene(brendan,cje,capsule_27,cj1421c).
reaction(brendan,cje,capsule_27,'glca-galfnac','glca-galfnac+omepn',1).
reaction(brendan,cje,capsule_27,'pre-omepn','glca-galfnac+omepn',1).
reaction_subpathway(brendan,cje,capsule_27,cap_addgalfomepn).
reaction_pathway(brendan,cje,capsule_27,capsule).

reaction_t(capsule_28).
reaction_gene(brendan,cje,capsule_28,cj1421c).
reaction(brendan,cje,capsule_28,'glca+hep-galfnac','glca+hep-galfnac+omepn',1).
```

```
reaction(brendan,cje,capsule_28,'pre-omepn','glca+hep-galfnac+omepn',1).
reaction_subpathway(brendan,cje,capsule_28,cap_addgalfomepn).
reaction_pathway(brendan,cje,capsule_28,capsule).

reaction_t(capsule_29a).
reaction_gene(brendan,cje,capsule_29a,cj1421c).
reaction(brendan,cje,capsule_29a,'glca+hep+6ome-galfnac','glca+hep+6ome-galfnac+omepn',1).
reaction(brendan,cje,capsule_29a,'pre-omepn','glca+hep+6ome-galfnac+omepn',1).
reaction_subpathway(brendan,cje,capsule_29a,cap_addgalfomepn).
reaction_pathway(brendan,cje,capsule_29a,capsule).

reaction_t(capsule_30).
reaction_gene(brendan,cje,capsule_30,cj1421c).
reaction(brendan,cje,capsule_30,'glca+hep+omepn-galfnac','glca+hep+omepn-galfnac+omepn',1).
reaction(brendan,cje,capsule_30,'pre-omepn','glca+hep+omepn-galfnac+omepn',1).
reaction_subpathway(brendan,cje,capsule_30,cap_addgalfomepn).
reaction_pathway(brendan,cje,capsule_30,capsule).

reaction_t(capsule_31a).
reaction_gene(brendan,cje,capsule_31a,cj1421c).
reaction(brendan,cje,capsule_31a,'glca+hep+omepn+6ome-galfnac','glca+hep+omepn+6ome-galfnac+omepn',1).
reaction(brendan,cje,capsule_31a,'pre-omepn','glca+hep+omepn+6ome-galfnac+omepn',1).
reaction_subpathway(brendan,cje,capsule_31a,cap_addgalfomepn).
reaction_pathway(brendan,cje,capsule_31a,capsule).

reaction_t(capsule_32).
reaction_gene(brendan,cje,capsule_32,cj1421c).
reaction(brendan,cje,capsule_32,'glca-galfnac-ribf','glca-galfnac+omepn-ribf',1).
reaction(brendan,cje,capsule_32,'pre-omepn','glca-galfnac+omepn-ribf',1).
reaction_subpathway(brendan,cje,capsule_32,cap_addgalfomepn).
reaction_pathway(brendan,cje,capsule_32,capsule).

reaction_t(capsule_33).
reaction_gene(brendan,cje,capsule_33,cj1421c).
reaction(brendan,cje,capsule_33,'glca+hep-galfnac-ribf','glca+hep-galfnac+omepn-ribf',1).
reaction(brendan,cje,capsule_33,'pre-omepn','glca+hep-galfnac+omepn-ribf',1).
reaction_subpathway(brendan,cje,capsule_33,cap_addgalfomepn).
reaction_pathway(brendan,cje,capsule_33,capsule).

reaction_t(capsule_34a).
reaction_gene(brendan,cje,capsule_34a,cj1421c).
reaction(brendan,cje,capsule_34a,'glca+hep+6ome-galfnac-ribf','glca+hep+6ome-galfnac+omepn-ribf',1).
reaction(brendan,cje,capsule_34a,'pre-omepn','glca+hep+6ome-galfnac+omepn-ribf',1).
reaction_subpathway(brendan,cje,capsule_34a,cap_addgalfomepn).
reaction_pathway(brendan,cje,capsule_34a,capsule).

reaction_t(capsule_35).
reaction_gene(brendan,cje,capsule_35,cj1421c).
reaction(brendan,cje,capsule_35,'glca+hep+omepn-galfnac-ribf','glca+hep+omepn-galfnac+omepn-ribf',1).
reaction(brendan,cje,capsule_35,'pre-omepn','glca+hep+omepn-galfnac+omepn-ribf',1).
reaction_subpathway(brendan,cje,capsule_35,cap_addgalfomepn).
reaction_pathway(brendan,cje,capsule_35,capsule).

reaction_t(capsule_36a).
```

```
reaction_gene(brendan,cje,capsule_36a,cj1421c).
reaction(brendan,cje,capsule_36a,'glca+hep+omepn+6ome-galfnac-ribf','glca+hep+omepn+6ome-galfnac+omepn-ribf',1).
reaction(brendan,cje,capsule_36a,'pre-omepn','glca+hep+omepn+6ome-galfnac+omepn-ribf',1).
reaction_subpathway(brendan,cje,capsule_36a,cap_addgalfomepn).
reaction_pathway(brendan,cje,capsule_36a,capsule).

%% Adding ribf

reaction_t(capsule_37).
reaction_gene(brendan,cje,capsule_37,unknown).
reaction(brendan,cje,capsule_37,'glca-galfnac','glca-galfnac-ribf',1).
reaction(brendan,cje,capsule_37,'pre-ribf','glca-galfnac-ribf',1).
reaction_subpathway(brendan,cje,capsule_37,cap_addribf).
reaction_pathway(brendan,cje,capsule_37,capsule).

reaction_t(capsule_38).
reaction_gene(brendan,cje,capsule_38,unknown).
reaction(brendan,cje,capsule_38,'glca+hep-galfnac','glca+hep-galfnac-ribf',1).
reaction(brendan,cje,capsule_38,'pre-ribf','glca+hep-galfnac-ribf',1).
reaction_subpathway(brendan,cje,capsule_38,cap_addribf).
reaction_pathway(brendan,cje,capsule_38,capsule).

reaction_t(capsule_39a).
reaction_gene(brendan,cje,capsule_39a,unknown).
reaction(brendan,cje,capsule_39a,'glca+hep+6ome-galfnac','glca+hep+6ome-galfnac-ribf',1).
reaction(brendan,cje,capsule_39a,'pre-ribf','glca+hep+6ome-galfnac-ribf',1).
reaction_subpathway(brendan,cje,capsule_39a,cap_addribf).
reaction_pathway(brendan,cje,capsule_39a,capsule).

reaction_t(capsule_40).
reaction_gene(brendan,cje,capsule_40,unknown).
reaction(brendan,cje,capsule_40,'glca+hep+omepn-galfnac','glca+hep+omepn-galfnac-ribf',1).
reaction(brendan,cje,capsule_40,'pre-ribf','glca+hep+omepn-galfnac-ribf',1).
reaction_subpathway(brendan,cje,capsule_40,cap_addribf).
reaction_pathway(brendan,cje,capsule_40,capsule).

reaction_t(capsule_41a).
reaction_gene(brendan,cje,capsule_41a,unknown).
reaction(brendan,cje,capsule_41a,'glca+hep+omepn+6ome-galfnac','glca+hep+omepn+6ome-galfnac-ribf',1).
reaction(brendan,cje,capsule_41a,'pre-ribf','glca+hep+omepn+6ome-galfnac-ribf',1).
reaction_subpathway(brendan,cje,capsule_41a,cap_addribf).
reaction_pathway(brendan,cje,capsule_41a,capsule).

reaction_t(capsule_42).
reaction_gene(brendan,cje,capsule_42,unknown).
reaction(brendan,cje,capsule_42,'glca-galfnac+omepn','glca-galfnac+omepn-ribf',1).
reaction(brendan,cje,capsule_42,'pre-ribf','glca-galfnac+omepn-ribf',1).
reaction_subpathway(brendan,cje,capsule_42,cap_addribf).
reaction_pathway(brendan,cje,capsule_42,capsule).

reaction_t(capsule_43).
reaction_gene(brendan,cje,capsule_43,unknown).
reaction(brendan,cje,capsule_43,'glca+hep-galfnac+omepn','glca+hep-galfnac+omepn-ribf',1).
```

```
reaction(brendan,cje,capsule_43,'pre-ribf','glca+hep-galfnac+omepn-ribf',1).
reaction_subpathway(brendan,cje,capsule_43,cap_addribf).
reaction_pathway(brendan,cje,capsule_43,capsule).

reaction_t(capsule_44a).
reaction_gene(brendan,cje,capsule_44a,unknown).
reaction(brendan,cje,capsule_44a,'glca+hep+6ome-galfnac+omepn','glca+hep+6ome-galfnac+omepn-ribf',1).
reaction(brendan,cje,capsule_44a,'pre-ribf','glca+hep+6ome-galfnac+omepn-ribf',1).
reaction_subpathway(brendan,cje,capsule_44a,cap_addribf).
reaction_pathway(brendan,cje,capsule_44a,capsule).

reaction_t(capsule_45).
reaction_gene(brendan,cje,capsule_45,unknown).
reaction(brendan,cje,capsule_45,'glca+hep+omepn-galfnac+omepn','glca+hep+omepn-galfnac+omepn-ribf',1).
reaction(brendan,cje,capsule_45,'pre-ribf','glca+hep+omepn-galfnac+omepn-ribf',1).
reaction_subpathway(brendan,cje,capsule_45,cap_addribf).
reaction_pathway(brendan,cje,capsule_45,capsule).

reaction_t(capsule_46a).
reaction_gene(brendan,cje,capsule_46a,unknown).
reaction(brendan,cje,capsule_46a,'glca+hep+omepn+6ome-galfnac+omepn','glca+hep+omepn+6ome-galfnac+omepn-
ribf',1).
reaction(brendan,cje,capsule_46a,'pre-ribf','glca+hep+omepn+6ome-galfnac+omepn-ribf',1).
reaction_subpathway(brendan,cje,capsule_46a,cap_addribf).
reaction_pathway(brendan,cje,capsule_46a,capsule).


%% adding ngro

reaction_t(capsule_addngro1).
reaction_gene(brendan,cje,capsule_addngro1,unknown).
reaction_subpathway(brendan,cje,capsule_addngro1,cap_addngro).
reaction_pathway(brendan,cje,capsule_addngro1,capsule).
reaction(brendan,cje,capsule_addngro1,'glca+hep','glca6ngro+hep',1).
reaction(brendan,cje,capsule_addngro1,'pre-ngro','glca6ngro+hep',1).

reaction_t(capsule_addngro2).
reaction_gene(brendan,cje,capsule_addngro2,unknown).
reaction_subpathway(brendan,cje,capsule_addngro2,cap_addngro).
reaction_pathway(brendan,cje,capsule_addngro2,capsule).
reaction(brendan,cje,capsule_addngro2,'glca+hep+omepn','glca6ngro+hep+omepn',1).
reaction(brendan,cje,capsule_addngro2,'pre-ngro','glca6ngro+hep+omepn',1).

reaction_t(capsule_addngro4).
reaction_gene(brendan,cje,capsule_addngro4,unknown).
reaction_subpathway(brendan,cje,capsule_addngro4,cap_addngro).
reaction_pathway(brendan,cje,capsule_addngro4,capsule).
reaction(brendan,cje,capsule_addngro4,'glca+hep+6ome','glca6ngro+hep+6ome',1).
reaction(brendan,cje,capsule_addngro4,'pre-ngro','glca6ngro+hep+6ome',1).

reaction_t(capsule_addngro6).
reaction_gene(brendan,cje,capsule_addngro6,unknown).
reaction_subpathway(brendan,cje,capsule_addngro6,cap_addngro).
reaction_pathway(brendan,cje,capsule_addngro6,capsule).
```

```
reaction(brendan,cje,capsule_addngro6,'glca+hep+omepn+6ome','glca6ngro+hep+omepn+6ome',1).
reaction(brendan,cje,capsule_addngro6,'pre-ngro','glca6ngro+hep+omepn+6ome',1).


%% GalfNAc group + phase variable group

reaction_t(capsule_addngro9).
reaction_gene(brendan,cje,capsule_addngro9,unknown).
reaction_subpathway(brendan,cje,capsule_addngro9,cap_addngro).
reaction_pathway(brendan,cje,capsule_addngro9,capsule).
reaction(brendan,cje,capsule_addngro9,'glca-galfnac','glca6ngro-galfnac',1).
reaction(brendan,cje,capsule_addngro9,'pre-ngro','glca6ngro-galfnac',1).

reaction_t(capsule_addngro10).
reaction_gene(brendan,cje,capsule_addngro10,unknown).
reaction_subpathway(brendan,cje,capsule_addngro10,cap_addngro).
reaction_pathway(brendan,cje,capsule_addngro10,capsule).
reaction(brendan,cje,capsule_addngro10,'glca+hep-galfnac','glca6ngro+hep-galfnac',1).
reaction(brendan,cje,capsule_addngro10,'pre-ngro','glca6ngro+hep-galfnac',1).

reaction_t(capsule_addngro11).
reaction_gene(brendan,cje,capsule_addngro11,unknown).
reaction_subpathway(brendan,cje,capsule_addngro11,cap_addngro).
reaction_pathway(brendan,cje,capsule_addngro11,capsule).
reaction(brendan,cje,capsule_addngro11,'glca+hep+omepn-galfnac','glca6ngro+hep+omepn-galfnac',1).
reaction(brendan,cje,capsule_addngro11,'pre-ngro','glca6ngro+hep+omepn-galfnac',1).

reaction_t(capsule_addngro13).
reaction_gene(brendan,cje,capsule_addngro13,unknown).
reaction_subpathway(brendan,cje,capsule_addngro13,cap_addngro).
reaction_pathway(brendan,cje,capsule_addngro13,capsule).
reaction(brendan,cje,capsule_addngro13,'glca+hep+6ome-galfnac','glca6ngro+hep+6ome-galfnac',1).
reaction(brendan,cje,capsule_addngro13,'pre-ngro','glca6ngro+hep+6ome-galfnac',1).

reaction_t(capsule_addngro15).
reaction_gene(brendan,cje,capsule_addngro15,unknown).
reaction_subpathway(brendan,cje,capsule_addngro15,cap_addngro).
reaction_pathway(brendan,cje,capsule_addngro15,capsule).
reaction(brendan,cje,capsule_addngro15,'glca+hep+omepn+6ome-galfnac','glca6ngro+hep+omepn+6ome-galfnac',1).
reaction(brendan,cje,capsule_addngro15,'pre-ngro','glca6ngro+hep+omepn+6ome-galfnac',1).

reaction_t(capsule_addngro18).
reaction_gene(brendan,cje,capsule_addngro18,unknown).
reaction_subpathway(brendan,cje,capsule_addngro18,cap_addngro).
reaction_pathway(brendan,cje,capsule_addngro18,capsule).
reaction(brendan,cje,capsule_addngro18,'glca-galfnac+omepn','glca6ngro-galfnac+omepn',1).
reaction(brendan,cje,capsule_addngro18,'pre-ngro','glca6ngro-galfnac+omepn',1).

reaction_t(capsule_addngro19).
reaction_gene(brendan,cje,capsule_addngro19,unknown).
reaction_subpathway(brendan,cje,capsule_addngro19,cap_addngro).
reaction_pathway(brendan,cje,capsule_addngro19,capsule).
reaction(brendan,cje,capsule_addngro19,'glca+hep-galfnac+omepn','glca6ngro+hep-galfnac+omepn',1).
reaction(brendan,cje,capsule_addngro19,'pre-ngro','glca6ngro+hep-galfnac+omepn',1).
```

```
reaction_t(capsule_addngro20).
reaction_gene(brendan,cje,capsule_addngro20,unknown).
reaction_subpathway(brendan,cje,capsule_addngro20,cap_addngro).
reaction_pathway(brendan,cje,capsule_addngro20,capsule).
reaction(brendan,cje,capsule_addngro20,'glca+hep+omepn-galfnac+omepn','glca6ngro+hep+omepn-galfnac+omepn',1).
reaction(brendan,cje,capsule_addngro20,'pre-ngro','glca6ngro+hep+omepn-galfnac+omepn',1).

reaction_t(capsule_addngro22).
reaction_gene(brendan,cje,capsule_addngro22,unknown).
reaction_subpathway(brendan,cje,capsule_addngro22,cap_addngro).
reaction_pathway(brendan,cje,capsule_addngro22,capsule).
reaction(brendan,cje,capsule_addngro22,'glca+hep+6ome-galfnac+omepn','glca6ngro+hep+6ome-galfnac+omepn',1).
reaction(brendan,cje,capsule_addngro22,'pre-ngro','glca6ngro+hep+6ome-galfnac+omepn',1).

reaction_t(capsule_addngro24).
reaction_gene(brendan,cje,capsule_addngro24,unknown).
reaction_subpathway(brendan,cje,capsule_addngro24,cap_addngro).
reaction_pathway(brendan,cje,capsule_addngro24,capsule).
reaction(brendan,cje,capsule_addngro24,'glca+hep+omepn+6ome-galfnac+omepn','glca6ngro+hep+omepn+6ome-
galfnac+omepn',1).
reaction(brendan,cje,capsule_addngro24,'pre-ngro','glca6ngro+hep+omepn+6ome-galfnac+omepn',1).


%% Ribf group

reaction_t(capsule_addngro27).
reaction_gene(brendan,cje,capsule_addngro27,unknown).
reaction_subpathway(brendan,cje,capsule_addngro27,cap_addngro).
reaction_pathway(brendan,cje,capsule_addngro27,capsule).
reaction(brendan,cje,capsule_addngro27,'glca-galfnac-ribf','glca6ngro-galfnac-ribf',1).
reaction(brendan,cje,capsule_addngro27,'pre-ngro','glca6ngro-galfnac-ribf',1).

reaction_t(capsule_addngro28).
reaction_gene(brendan,cje,capsule_addngro28,unknown).
reaction_subpathway(brendan,cje,capsule_addngro28,cap_addngro).
reaction_pathway(brendan,cje,capsule_addngro28,capsule).
reaction(brendan,cje,capsule_addngro28,'glca+hep-galfnac-ribf','glca6ngro+hep-galfnac-ribf',1).
reaction(brendan,cje,capsule_addngro28,'pre-ngro','glca6ngro+hep-galfnac-ribf',1).

reaction_t(capsule_addngro29).
reaction_gene(brendan,cje,capsule_addngro29,unknown).
reaction_subpathway(brendan,cje,capsule_addngro29,cap_addngro).
reaction_pathway(brendan,cje,capsule_addngro29,capsule).
reaction(brendan,cje,capsule_addngro29,'glca+hep+omepn-galfnac-ribf','glca6ngro+hep+omepn-galfnac-ribf',1).
reaction(brendan,cje,capsule_addngro29,'pre-ngro','glca6ngro+hep+omepn-galfnac-ribf',1).

reaction_t(capsule_addngro31).
reaction_gene(brendan,cje,capsule_addngro31,unknown).
reaction_subpathway(brendan,cje,capsule_addngro31,cap_addngro).
reaction_pathway(brendan,cje,capsule_addngro31,capsule).
reaction(brendan,cje,capsule_addngro31,'glca+hep+6ome-galfnac-ribf','glca6ngro+hep+6ome-galfnac-ribf',1).
reaction(brendan,cje,capsule_addngro31,'pre-ngro','glca6ngro+hep+6ome-galfnac-ribf',1).
```

```
reaction_t(capsule_addngro33).
reaction_gene(brendan,cje,capsule_addngro33,unknown).
reaction_subpathway(brendan,cje,capsule_addngro33,cap_addngro).
reaction_pathway(brendan,cje,capsule_addngro33,capsule).
reaction(brendan,cje,capsule_addngro33,'glca+hep+omepn+6ome-galfnac-ribf','glca6ngro+hep+omepn+6ome-galfnac-ribf',1).
reaction(brendan,cje,capsule_addngro33,'pre-ngro','glca6ngro+hep+omepn+6ome-galfnac-ribf',1).

reaction_t(capsule_addngro36).
reaction_gene(brendan,cje,capsule_addngro36,unknown).
reaction_subpathway(brendan,cje,capsule_addngro36,cap_addngro).
reaction_pathway(brendan,cje,capsule_addngro36,capsule).
reaction(brendan,cje,capsule_addngro36,'glca-galfnac+omepn-ribf','glca6ngro-galfnac+omepn-ribf',1).
reaction(brendan,cje,capsule_addngro36,'pre-ngro','glca6ngro-galfnac+omepn-ribf',1).

reaction_t(capsule_addngro37).
reaction_gene(brendan,cje,capsule_addngro37,unknown).
reaction_subpathway(brendan,cje,capsule_addngro37,cap_addngro).
reaction_pathway(brendan,cje,capsule_addngro37,capsule).
reaction(brendan,cje,capsule_addngro37,'glca+hep-galfnac+omepn-ribf','glca6ngro+hep-galfnac+omepn-ribf',1).
reaction(brendan,cje,capsule_addngro37,'pre-ngro','glca6ngro+hep-galfnac+omepn-ribf',1).

reaction_t(capsule_addngro38).
reaction_gene(brendan,cje,capsule_addngro38,unknown).
reaction_subpathway(brendan,cje,capsule_addngro38,cap_addngro).
reaction_pathway(brendan,cje,capsule_addngro38,capsule).
reaction(brendan,cje,capsule_addngro38,'glca+hep+omepn-galfnac+omepn-ribf','glca6ngro+hep+omepn-galfnac+omepn-ribf',1).
reaction(brendan,cje,capsule_addngro38,'pre-ngro','glca6ngro+hep+omepn-galfnac+omepn-ribf',1).

reaction_t(capsule_addngro40).
reaction_gene(brendan,cje,capsule_addngro40,unknown).
reaction_subpathway(brendan,cje,capsule_addngro40,cap_addngro).
reaction_pathway(brendan,cje,capsule_addngro40,capsule).
reaction(brendan,cje,capsule_addngro40,'glca+hep+6ome-galfnac+omepn-ribf','glca6ngro+hep+6ome-galfnac+omepn-ribf',1).
reaction(brendan,cje,capsule_addngro40,'pre-ngro','glca6ngro+hep+6ome-galfnac+omepn-ribf',1).

reaction_t(capsule_addngro42).
reaction_gene(brendan,cje,capsule_addngro42,unknown).
reaction_subpathway(brendan,cje,capsule_addngro42,cap_addngro).
reaction_pathway(brendan,cje,capsule_addngro42,capsule).
reaction(brendan,cje,capsule_addngro42,'glca+hep+omepn+6ome-galfnac+omepn-ribf','glca6ngro+hep+omepn+6ome-galfnac+omepn-ribf',1).
reaction(brendan,cje,capsule_addngro42,'pre-ngro','glca6ngro+hep+omepn+6ome-galfnac+omepn-ribf',1).




%% adding etn
% .....................
```

```
%% Lipooligosaccharide synthesis pathway
%%...

%% Capsule synthesis pathway

glycan_pathway(brendan,cje,capsule).
glycan_root(brendan,cje,capsule,'Chain').
glycan_part(brendan,cje,capsule,'Chain','Chain').
glycan_part(brendan,cje,capsule,'GlcA','GlcA').
glycan_part(brendan,cje,capsule,'NGro','NGro').
glycan_part(brendan,cje,capsule,'EtN','EtN').
glycan_part(brendan,cje,capsule,'Hep','Hep').
glycan_part(brendan,cje,capsule,'Hep OMePN','OMePN').
glycan_part(brendan,cje,capsule,'Hep 3-OMe','3-OMe').
glycan_part(brendan,cje,capsule,'Hep 6-OMe','6-OMe').
glycan_part(brendan,cje,capsule,'GalfNAc','GalfNAc').
glycan_part(brendan,cje,capsule,'GalfNAc OMePN','OMePN').
glycan_part(brendan,cje,capsule,'Ribf','Ribf').

part_precursor(brendan,cje,capsule,'GlcA','pre-glca').
part_precursor(brendan,cje,capsule,'NGro','pre-ngro').
part_precursor(brendan,cje,capsule,'EtN','pre-etn').
part_precursor(brendan,cje,capsule,'Hep','gdp-d-al-gluco-hep').
part_precursor(brendan,cje,capsule,'Hep OMePN','pre-omepn').
part_precursor(brendan,cje,capsule,'Hep 3-OMe','pre-ome').
part_precursor(brendan,cje,capsule,'Hep 6-OMe','pre-ome').
part_precursor(brendan,cje,capsule,'GalfNAc','pre-galfnac').
part_precursor(brendan,cje,capsule,'GalfNAc OMePN','pre-omepn').
part_precursor(brendan,cje,capsule,'Ribf','pre-ribf').

glycan_join(brendan,cje,capsule,'Chain','GlcA',cj1441c).
guess_glycan_join(brendan,cje,capsule,'Chain','GlcA',cj1441c).
glycan_join(brendan,cje,capsule,'GlcA','NGro',unknown).
glycan_join(brendan,cje,capsule,'GlcA','EtN',unknown).
glycan_join(brendan,cje,capsule,'GlcA','Hep',cj1431c).
glycan_join(brendan,cje,capsule,'Hep','Hep OMePN',cj1422c).
glycan_join(brendan,cje,capsule,'Hep','Hep 3-OMe',unknown).
glycan_join(brendan,cje,capsule,'Hep','Hep 6-OMe',cj1426c).
glycan_join(brendan,cje,capsule,'GlcA','GalfNAc',cj1439c).
guess_glycan_join(brendan,cje,capsule,'GlcA','GalfNAc',cj1439c).
glycan_join(brendan,cje,capsule,'GalfNAc','GalfNAc OMePN',cj1421c).
glycan_join(brendan,cje,capsule,'GalfNAc','Ribf',unknown).


%% Contains part

%% Chain

contains_part(brendan,cje,capsule,'capsule_chain','Chain').
contains_part(brendan,cje,capsule,'glca','Chain').
contains_part(brendan,cje,capsule,'glca6ngro','Chain').
contains_part(brendan,cje,capsule,'glca6etn','Chain').
contains_part(brendan,cje,capsule,'glca6ngro+hep','Chain').
contains_part(brendan,cje,capsule,'glca6ngro+hep+omepn','Chain').
```

```
contains_part(brendan,cje,capsule,'glca6ngro+hep+3ome','Chain').
contains_part(brendan,cje,capsule,'glca6ngro+hep+6ome','Chain').
contains_part(brendan,cje,capsule,'glca6ngro+hep+omepn+3ome','Chain').
contains_part(brendan,cje,capsule,'glca6ngro+hep+omepn+6ome','Chain').
contains_part(brendan,cje,capsule,'glca6ngro+hep+3ome+6ome','Chain').
contains_part(brendan,cje,capsule,'glca6ngro+hep+omepn+3ome+6ome','Chain').
contains_part(brendan,cje,capsule,'glca6ngro-galfnac','Chain').
contains_part(brendan,cje,capsule,'glca6ngro+hep-galfnac','Chain').
contains_part(brendan,cje,capsule,'glca6ngro+hep+omepn-galfnac','Chain').
contains_part(brendan,cje,capsule,'glca6ngro+hep+3ome-galfnac','Chain').
contains_part(brendan,cje,capsule,'glca6ngro+hep+6ome-galfnac','Chain').
contains_part(brendan,cje,capsule,'glca6ngro+hep+omepn+3ome-galfnac','Chain').
contains_part(brendan,cje,capsule,'glca6ngro+hep+omepn+6ome-galfnac','Chain').
contains_part(brendan,cje,capsule,'glca6ngro+hep+3ome+6ome-galfnac','Chain').
contains_part(brendan,cje,capsule,'glca6ngro+hep+omepn+3ome+6ome-galfnac','Chain').
contains_part(brendan,cje,capsule,'glca6ngro-galfnac+omepn','Chain').
contains_part(brendan,cje,capsule,'glca6ngro+hep-galfnac+omepn','Chain').
contains_part(brendan,cje,capsule,'glca6ngro+hep+omepn-galfnac+omepn','Chain').
contains_part(brendan,cje,capsule,'glca6ngro+hep+3ome-galfnac+omepn','Chain').
contains_part(brendan,cje,capsule,'glca6ngro+hep+6ome-galfnac+omepn','Chain').
contains_part(brendan,cje,capsule,'glca6ngro+hep+omepn+3ome-galfnac+omepn','Chain').
contains_part(brendan,cje,capsule,'glca6ngro+hep+omepn+6ome-galfnac+omepn','Chain').
contains_part(brendan,cje,capsule,'glca6ngro+hep+3ome+6ome-galfnac+omepn','Chain').
contains_part(brendan,cje,capsule,'glca6ngro+hep+omepn+3ome+6ome-galfnac+omepn','Chain').
contains_part(brendan,cje,capsule,'glca6ngro-galfnac-ribf','Chain').
contains_part(brendan,cje,capsule,'glca6ngro+hep-galfnac-ribf','Chain').
contains_part(brendan,cje,capsule,'glca6ngro+hep+omepn-galfnac-ribf','Chain').
contains_part(brendan,cje,capsule,'glca6ngro+hep+3ome-galfnac-ribf','Chain').
contains_part(brendan,cje,capsule,'glca6ngro+hep+6ome-galfnac-ribf','Chain').
contains_part(brendan,cje,capsule,'glca6ngro+hep+omepn+3ome-galfnac-ribf','Chain').
contains_part(brendan,cje,capsule,'glca6ngro+hep+omepn+6ome-galfnac-ribf','Chain').
contains_part(brendan,cje,capsule,'glca6ngro+hep+3ome+6ome-galfnac-ribf','Chain').
contains_part(brendan,cje,capsule,'glca6ngro+hep+omepn+3ome+6ome-galfnac-ribf','Chain').
contains_part(brendan,cje,capsule,'glca6ngro-galfnac+omepn-ribf','Chain').
contains_part(brendan,cje,capsule,'glca6ngro+hep-galfnac+omepn-ribf','Chain').
contains_part(brendan,cje,capsule,'glca6ngro+hep+omepn-galfnac+omepn-ribf','Chain').
contains_part(brendan,cje,capsule,'glca6ngro+hep+3ome-galfnac+omepn-ribf','Chain').
contains_part(brendan,cje,capsule,'glca6ngro+hep+6ome-galfnac+omepn-ribf','Chain').
contains_part(brendan,cje,capsule,'glca6ngro+hep+omepn+3ome-galfnac+omepn-ribf','Chain').
contains_part(brendan,cje,capsule,'glca6ngro+hep+omepn+6ome-galfnac+omepn-ribf','Chain').
contains_part(brendan,cje,capsule,'glca6ngro+hep+3ome+6ome-galfnac+omepn-ribf','Chain').
contains_part(brendan,cje,capsule,'glca6ngro+hep+omepn+3ome+6ome-galfnac+omepn-ribf','Chain').
contains_part(brendan,cje,capsule,'glca6etn+hep','Chain').
contains_part(brendan,cje,capsule,'glca6etn+hep+omepn','Chain').
contains_part(brendan,cje,capsule,'glca6etn+hep+3ome','Chain').
contains_part(brendan,cje,capsule,'glca6etn+hep+6ome','Chain').
contains_part(brendan,cje,capsule,'glca6etn+hep+omepn+3ome','Chain').
contains_part(brendan,cje,capsule,'glca6etn+hep+omepn+6ome','Chain').
contains_part(brendan,cje,capsule,'glca6etn+hep+3ome+6ome','Chain').
contains_part(brendan,cje,capsule,'glca6etn+hep+omepn+3ome+6ome','Chain').
contains_part(brendan,cje,capsule,'glca6etn-galfnac','Chain').
contains_part(brendan,cje,capsule,'glca6etn+hep-galfnac','Chain').
contains_part(brendan,cje,capsule,'glca6etn+hep+omepn-galfnac','Chain').
contains_part(brendan,cje,capsule,'glca6etn+hep+3ome-galfnac','Chain').
```

```
contains_part(brendan,cje,capsule,'glca6etn+hep+6ome-galfnac','Chain').
contains_part(brendan,cje,capsule,'glca6etn+hep+omepn+3ome-galfnac','Chain').
contains_part(brendan,cje,capsule,'glca6etn+hep+omepn+6ome-galfnac','Chain').
contains_part(brendan,cje,capsule,'glca6etn+hep+3ome+6ome-galfnac','Chain').
contains_part(brendan,cje,capsule,'glca6etn+hep+omepn+3ome+6ome-galfnac','Chain').
contains_part(brendan,cje,capsule,'glca6etn-galfnac+omepn','Chain').
contains_part(brendan,cje,capsule,'glca6etn+hep-galfnac+omepn','Chain').
contains_part(brendan,cje,capsule,'glca6etn+hep+omepn-galfnac+omepn','Chain').
contains_part(brendan,cje,capsule,'glca6etn+hep+3ome-galfnac+omepn','Chain').
contains_part(brendan,cje,capsule,'glca6etn+hep+6ome-galfnac+omepn','Chain').
contains_part(brendan,cje,capsule,'glca6etn+hep+omepn+3ome-galfnac+omepn','Chain').
contains_part(brendan,cje,capsule,'glca6etn+hep+omepn+6ome-galfnac+omepn','Chain').
contains_part(brendan,cje,capsule,'glca6etn+hep+3ome+6ome-galfnac+omepn','Chain').
contains_part(brendan,cje,capsule,'glca6etn+hep+omepn+3ome+6ome-galfnac+omepn','Chain').
contains_part(brendan,cje,capsule,'glca6etn-galfnac-ribf','Chain').
contains_part(brendan,cje,capsule,'glca6etn+hep-galfnac-ribf','Chain').
contains_part(brendan,cje,capsule,'glca6etn+hep+omepn-galfnac-ribf','Chain').
contains_part(brendan,cje,capsule,'glca6etn+hep+3ome-galfnac-ribf','Chain').
contains_part(brendan,cje,capsule,'glca6etn+hep+6ome-galfnac-ribf','Chain').
contains_part(brendan,cje,capsule,'glca6etn+hep+omepn+3ome-galfnac-ribf','Chain').
contains_part(brendan,cje,capsule,'glca6etn+hep+omepn+6ome-galfnac-ribf','Chain').
contains_part(brendan,cje,capsule,'glca6etn+hep+3ome+6ome-galfnac-ribf','Chain').
contains_part(brendan,cje,capsule,'glca6etn+hep+omepn+3ome+6ome-galfnac-ribf','Chain').
contains_part(brendan,cje,capsule,'glca6etn-galfnac+omepn-ribf','Chain').
contains_part(brendan,cje,capsule,'glca6etn+hep-galfnac+omepn-ribf','Chain').
contains_part(brendan,cje,capsule,'glca6etn+hep+omepn-galfnac+omepn-ribf','Chain').
contains_part(brendan,cje,capsule,'glca6etn+hep+3ome-galfnac+omepn-ribf','Chain').
contains_part(brendan,cje,capsule,'glca6etn+hep+6ome-galfnac+omepn-ribf','Chain').
contains_part(brendan,cje,capsule,'glca6etn+hep+omepn+3ome-galfnac+omepn-ribf','Chain').
contains_part(brendan,cje,capsule,'glca6etn+hep+omepn+6ome-galfnac+omepn-ribf','Chain').
contains_part(brendan,cje,capsule,'glca6etn+hep+3ome+6ome-galfnac+omepn-ribf','Chain').
contains_part(brendan,cje,capsule,'glca6etn+hep+omepn+3ome+6ome-galfnac+omepn-ribf','Chain').
contains_part(brendan,cje,capsule,'glca+hep','Chain').
contains_part(brendan,cje,capsule,'glca+hep+omepn','Chain').
contains_part(brendan,cje,capsule,'glca+hep+3ome','Chain').
contains_part(brendan,cje,capsule,'glca+hep+6ome','Chain').
contains_part(brendan,cje,capsule,'glca+hep+omepn+3ome','Chain').
contains_part(brendan,cje,capsule,'glca+hep+omepn+6ome','Chain').
contains_part(brendan,cje,capsule,'glca+hep+3ome+6ome','Chain').
contains_part(brendan,cje,capsule,'glca+hep+omepn+3ome+6ome','Chain').
contains_part(brendan,cje,capsule,'glca-galfnac','Chain').
contains_part(brendan,cje,capsule,'glca+hep-galfnac','Chain').
contains_part(brendan,cje,capsule,'glca+hep+omepn-galfnac','Chain').
contains_part(brendan,cje,capsule,'glca+hep+3ome-galfnac','Chain').
contains_part(brendan,cje,capsule,'glca+hep+6ome-galfnac','Chain').
contains_part(brendan,cje,capsule,'glca+hep+omepn+3ome-galfnac','Chain').
contains_part(brendan,cje,capsule,'glca+hep+omepn+6ome-galfnac','Chain').
contains_part(brendan,cje,capsule,'glca+hep+3ome+6ome-galfnac','Chain').
contains_part(brendan,cje,capsule,'glca+hep+omepn+3ome+6ome-galfnac','Chain').
contains_part(brendan,cje,capsule,'glca-galfnac+omepn','Chain').
contains_part(brendan,cje,capsule,'glca+hep-galfnac+omepn','Chain').
contains_part(brendan,cje,capsule,'glca+hep+omepn-galfnac+omepn','Chain').
contains_part(brendan,cje,capsule,'glca+hep+3ome-galfnac+omepn','Chain').
contains_part(brendan,cje,capsule,'glca+hep+6ome-galfnac+omepn','Chain').
```

contains_part(brendan,cje,capsule,'glca+hep+omepn+3ome-galfnac+omepn','Chain').
contains_part(brendan,cje,capsule,'glca+hep+omepn+6ome-galfnac+omepn','Chain').
contains_part(brendan,cje,capsule,'glca+hep+3ome+6ome-galfnac+omepn','Chain').
contains_part(brendan,cje,capsule,'glca+hep+omepn+3ome+6ome-galfnac+omepn','Chain').
contains_part(brendan,cje,capsule,'glca-galfnac-ribf','Chain').
contains_part(brendan,cje,capsule,'glca+hep-galfnac-ribf','Chain').
contains_part(brendan,cje,capsule,'glca+hep+omepn-galfnac-ribf','Chain').
contains_part(brendan,cje,capsule,'glca+hep+3ome-galfnac-ribf','Chain').
contains_part(brendan,cje,capsule,'glca+hep+6ome-galfnac-ribf','Chain').
contains_part(brendan,cje,capsule,'glca+hep+omepn+3ome-galfnac-ribf','Chain').
contains_part(brendan,cje,capsule,'glca+hep+omepn+6ome-galfnac-ribf','Chain').
contains_part(brendan,cje,capsule,'glca+hep+3ome+6ome-galfnac-ribf','Chain').
contains_part(brendan,cje,capsule,'glca+hep+omepn+3ome+6ome-galfnac-ribf','Chain').
contains_part(brendan,cje,capsule,'glca-galfnac+omepn-ribf','Chain').
contains_part(brendan,cje,capsule,'glca+hep-galfnac+omepn-ribf','Chain').
contains_part(brendan,cje,capsule,'glca+hep+omepn-galfnac+omepn-ribf','Chain').
contains_part(brendan,cje,capsule,'glca+hep+3ome-galfnac+omepn-ribf','Chain').
contains_part(brendan,cje,capsule,'glca+hep+6ome-galfnac+omepn-ribf','Chain').
contains_part(brendan,cje,capsule,'glca+hep+omepn+3ome-galfnac+omepn-ribf','Chain').
contains_part(brendan,cje,capsule,'glca+hep+omepn+6ome-galfnac+omepn-ribf','Chain').
contains_part(brendan,cje,capsule,'glca+hep+3ome+6ome-galfnac+omepn-ribf','Chain').
contains_part(brendan,cje,capsule,'glca+hep+omepn+3ome+6ome-galfnac+omepn-ribf','Chain').

%% GlcA

contains_part(brendan,cje,capsule,'glca','GlcA').
contains_part(brendan,cje,capsule,'glca6ngro','GlcA').
contains_part(brendan,cje,capsule,'glca6etn','GlcA').
contains_part(brendan,cje,capsule,'glca6ngro+hep','GlcA').
contains_part(brendan,cje,capsule,'glca6ngro+hep+omepn','GlcA').
contains_part(brendan,cje,capsule,'glca6ngro+hep+3ome','GlcA').
contains_part(brendan,cje,capsule,'glca6ngro+hep+6ome','GlcA').
contains_part(brendan,cje,capsule,'glca6ngro+hep+omepn+3ome','GlcA').
contains_part(brendan,cje,capsule,'glca6ngro+hep+omepn+6ome','GlcA').
contains_part(brendan,cje,capsule,'glca6ngro+hep+3ome+6ome','GlcA').
contains_part(brendan,cje,capsule,'glca6ngro+hep+omepn+3ome+6ome','GlcA').
contains_part(brendan,cje,capsule,'glca6ngro-galfnac','GlcA').
contains_part(brendan,cje,capsule,'glca6ngro+hep-galfnac','GlcA').
contains_part(brendan,cje,capsule,'glca6ngro+hep+omepn-galfnac','GlcA').
contains_part(brendan,cje,capsule,'glca6ngro+hep+3ome-galfnac','GlcA').
contains_part(brendan,cje,capsule,'glca6ngro+hep+6ome-galfnac','GlcA').
contains_part(brendan,cje,capsule,'glca6ngro+hep+omepn+3ome-galfnac','GlcA').
contains_part(brendan,cje,capsule,'glca6ngro+hep+omepn+6ome-galfnac','GlcA').
contains_part(brendan,cje,capsule,'glca6ngro+hep+3ome+6ome-galfnac','GlcA').
contains_part(brendan,cje,capsule,'glca6ngro+hep+omepn+3ome+6ome-galfnac','GlcA').
contains_part(brendan,cje,capsule,'glca6ngro-galfnac+omepn','GlcA').
contains_part(brendan,cje,capsule,'glca6ngro+hep-galfnac+omepn','GlcA').
contains_part(brendan,cje,capsule,'glca6ngro+hep+omepn-galfnac+omepn','GlcA').
contains_part(brendan,cje,capsule,'glca6ngro+hep+3ome-galfnac+omepn','GlcA').
contains_part(brendan,cje,capsule,'glca6ngro+hep+6ome-galfnac+omepn','GlcA').
contains_part(brendan,cje,capsule,'glca6ngro+hep+omepn+3ome-galfnac+omepn','GlcA').
contains_part(brendan,cje,capsule,'glca6ngro+hep+omepn+6ome-galfnac+omepn','GlcA').
contains_part(brendan,cje,capsule,'glca6ngro+hep+3ome+6ome-galfnac+omepn','GlcA').
contains_part(brendan,cje,capsule,'glca6ngro+hep+omepn+3ome+6ome-galfnac+omepn','GlcA').

contains_part(brendan,cje,capsule,'glca6ngro-galfnac-ribf','GlcA').
contains_part(brendan,cje,capsule,'glca6ngro+hep-galfnac-ribf','GlcA').
contains_part(brendan,cje,capsule,'glca6ngro+hep+omepn-galfnac-ribf','GlcA').
contains_part(brendan,cje,capsule,'glca6ngro+hep+3ome-galfnac-ribf','GlcA').
contains_part(brendan,cje,capsule,'glca6ngro+hep+6ome-galfnac-ribf','GlcA').
contains_part(brendan,cje,capsule,'glca6ngro+hep+omepn+3ome-galfnac-ribf','GlcA').
contains_part(brendan,cje,capsule,'glca6ngro+hep+omepn+6ome-galfnac-ribf','GlcA').
contains_part(brendan,cje,capsule,'glca6ngro+hep+3ome+6ome-galfnac-ribf','GlcA').
contains_part(brendan,cje,capsule,'glca6ngro+hep+omepn+3ome+6ome-galfnac-ribf','GlcA').
contains_part(brendan,cje,capsule,'glca6ngro-galfnac+omepn-ribf','GlcA').
contains_part(brendan,cje,capsule,'glca6ngro+hep-galfnac+omepn-ribf','GlcA').
contains_part(brendan,cje,capsule,'glca6ngro+hep+omepn-galfnac+omepn-ribf','GlcA').
contains_part(brendan,cje,capsule,'glca6ngro+hep+3ome-galfnac+omepn-ribf','GlcA').
contains_part(brendan,cje,capsule,'glca6ngro+hep+6ome-galfnac+omepn-ribf','GlcA').
contains_part(brendan,cje,capsule,'glca6ngro+hep+omepn+3ome-galfnac+omepn-ribf','GlcA').
contains_part(brendan,cje,capsule,'glca6ngro+hep+omepn+6ome-galfnac+omepn-ribf','GlcA').
contains_part(brendan,cje,capsule,'glca6ngro+hep+3ome+6ome-galfnac+omepn-ribf','GlcA').
contains_part(brendan,cje,capsule,'glca6ngro+hep+omepn+3ome+6ome-galfnac+omepn-ribf','GlcA').
contains_part(brendan,cje,capsule,'glca6etn+hep','GlcA').
contains_part(brendan,cje,capsule,'glca6etn+hep+omepn','GlcA').
contains_part(brendan,cje,capsule,'glca6etn+hep+3ome','GlcA').
contains_part(brendan,cje,capsule,'glca6etn+hep+6ome','GlcA').
contains_part(brendan,cje,capsule,'glca6etn+hep+omepn+3ome','GlcA').
contains_part(brendan,cje,capsule,'glca6etn+hep+omepn+6ome','GlcA').
contains_part(brendan,cje,capsule,'glca6etn+hep+3ome+6ome','GlcA').
contains_part(brendan,cje,capsule,'glca6etn+hep+omepn+3ome+6ome','GlcA').
contains_part(brendan,cje,capsule,'glca6etn-galfnac','GlcA').
contains_part(brendan,cje,capsule,'glca6etn+hep-galfnac','GlcA').
contains_part(brendan,cje,capsule,'glca6etn+hep+omepn-galfnac','GlcA').
contains_part(brendan,cje,capsule,'glca6etn+hep+3ome-galfnac','GlcA').
contains_part(brendan,cje,capsule,'glca6etn+hep+6ome-galfnac','GlcA').
contains_part(brendan,cje,capsule,'glca6etn+hep+omepn+3ome-galfnac','GlcA').
contains_part(brendan,cje,capsule,'glca6etn+hep+omepn+6ome-galfnac','GlcA').
contains_part(brendan,cje,capsule,'glca6etn+hep+3ome+6ome-galfnac','GlcA').
contains_part(brendan,cje,capsule,'glca6etn+hep+omepn+3ome+6ome-galfnac','GlcA').
contains_part(brendan,cje,capsule,'glca6etn-galfnac+omepn','GlcA').
contains_part(brendan,cje,capsule,'glca6etn+hep-galfnac+omepn','GlcA').
contains_part(brendan,cje,capsule,'glca6etn+hep+omepn-galfnac+omepn','GlcA').
contains_part(brendan,cje,capsule,'glca6etn+hep+3ome-galfnac+omepn','GlcA').
contains_part(brendan,cje,capsule,'glca6etn+hep+6ome-galfnac+omepn','GlcA').
contains_part(brendan,cje,capsule,'glca6etn+hep+omepn+3ome-galfnac+omepn','GlcA').
contains_part(brendan,cje,capsule,'glca6etn+hep+omepn+6ome-galfnac+omepn','GlcA').
contains_part(brendan,cje,capsule,'glca6etn+hep+3ome+6ome-galfnac+omepn','GlcA').
contains_part(brendan,cje,capsule,'glca6etn+hep+omepn+3ome+6ome-galfnac+omepn','GlcA').
contains_part(brendan,cje,capsule,'glca6etn-galfnac-ribf','GlcA').
contains_part(brendan,cje,capsule,'glca6etn+hep-galfnac-ribf','GlcA').
contains_part(brendan,cje,capsule,'glca6etn+hep+omepn-galfnac-ribf','GlcA').
contains_part(brendan,cje,capsule,'glca6etn+hep+3ome-galfnac-ribf','GlcA').
contains_part(brendan,cje,capsule,'glca6etn+hep+6ome-galfnac-ribf','GlcA').
contains_part(brendan,cje,capsule,'glca6etn+hep+omepn+3ome-galfnac-ribf','GlcA').
contains_part(brendan,cje,capsule,'glca6etn+hep+omepn+6ome-galfnac-ribf','GlcA').
contains_part(brendan,cje,capsule,'glca6etn+hep+3ome+6ome-galfnac-ribf','GlcA').
contains_part(brendan,cje,capsule,'glca6etn+hep+omepn+3ome+6ome-galfnac-ribf','GlcA').
contains_part(brendan,cje,capsule,'glca6etn-galfnac+omepn-ribf','GlcA').

contains_part(brendan,cje,capsule,'glca6etn+hep-galfnac+omepn-ribf','GlcA').
contains_part(brendan,cje,capsule,'glca6etn+hep+omepn-galfnac+omepn-ribf','GlcA').
contains_part(brendan,cje,capsule,'glca6etn+hep+3ome-galfnac+omepn-ribf','GlcA').
contains_part(brendan,cje,capsule,'glca6etn+hep+6ome-galfnac+omepn-ribf','GlcA').
contains_part(brendan,cje,capsule,'glca6etn+hep+omepn+3ome-galfnac+omepn-ribf','GlcA').
contains_part(brendan,cje,capsule,'glca6etn+hep+omepn+6ome-galfnac+omepn-ribf','GlcA').
contains_part(brendan,cje,capsule,'glca6etn+hep+3ome+6ome-galfnac+omepn-ribf','GlcA').
contains_part(brendan,cje,capsule,'glca6etn+hep+omepn+3ome+6ome-galfnac+omepn-ribf','GlcA').
contains_part(brendan,cje,capsule,'glca+hep','GlcA').
contains_part(brendan,cje,capsule,'glca+hep+omepn','GlcA').
contains_part(brendan,cje,capsule,'glca+hep+3ome','GlcA').
contains_part(brendan,cje,capsule,'glca+hep+6ome','GlcA').
contains_part(brendan,cje,capsule,'glca+hep+omepn+3ome','GlcA').
contains_part(brendan,cje,capsule,'glca+hep+omepn+6ome','GlcA').
contains_part(brendan,cje,capsule,'glca+hep+3ome+6ome','GlcA').
contains_part(brendan,cje,capsule,'glca+hep+omepn+3ome+6ome','GlcA').
contains_part(brendan,cje,capsule,'glca-galfnac','GlcA').
contains_part(brendan,cje,capsule,'glca+hep-galfnac','GlcA').
contains_part(brendan,cje,capsule,'glca+hep+omepn-galfnac','GlcA').
contains_part(brendan,cje,capsule,'glca+hep+3ome-galfnac','GlcA').
contains_part(brendan,cje,capsule,'glca+hep+6ome-galfnac','GlcA').
contains_part(brendan,cje,capsule,'glca+hep+omepn+3ome-galfnac','GlcA').
contains_part(brendan,cje,capsule,'glca+hep+omepn+6ome-galfnac','GlcA').
contains_part(brendan,cje,capsule,'glca+hep+3ome+6ome-galfnac','GlcA').
contains_part(brendan,cje,capsule,'glca+hep+omepn+3ome+6ome-galfnac','GlcA').
contains_part(brendan,cje,capsule,'glca-galfnac+omepn','GlcA').
contains_part(brendan,cje,capsule,'glca+hep-galfnac+omepn','GlcA').
contains_part(brendan,cje,capsule,'glca+hep+omepn-galfnac+omepn','GlcA').
contains_part(brendan,cje,capsule,'glca+hep+3ome-galfnac+omepn','GlcA').
contains_part(brendan,cje,capsule,'glca+hep+6ome-galfnac+omepn','GlcA').
contains_part(brendan,cje,capsule,'glca+hep+omepn+3ome-galfnac+omepn','GlcA').
contains_part(brendan,cje,capsule,'glca+hep+omepn+6ome-galfnac+omepn','GlcA').
contains_part(brendan,cje,capsule,'glca+hep+3ome+6ome-galfnac+omepn','GlcA').
contains_part(brendan,cje,capsule,'glca+hep+omepn+3ome+6ome-galfnac+omepn','GlcA').
contains_part(brendan,cje,capsule,'glca-galfnac-ribf','GlcA').
contains_part(brendan,cje,capsule,'glca+hep-galfnac-ribf','GlcA').
contains_part(brendan,cje,capsule,'glca+hep+omepn-galfnac-ribf','GlcA').
contains_part(brendan,cje,capsule,'glca+hep+3ome-galfnac-ribf','GlcA').
contains_part(brendan,cje,capsule,'glca+hep+6ome-galfnac-ribf','GlcA').
contains_part(brendan,cje,capsule,'glca+hep+omepn+3ome-galfnac-ribf','GlcA').
contains_part(brendan,cje,capsule,'glca+hep+omepn+6ome-galfnac-ribf','GlcA').
contains_part(brendan,cje,capsule,'glca+hep+3ome+6ome-galfnac-ribf','GlcA').
contains_part(brendan,cje,capsule,'glca+hep+omepn+3ome+6ome-galfnac-ribf','GlcA').
contains_part(brendan,cje,capsule,'glca-galfnac+omepn-ribf','GlcA').
contains_part(brendan,cje,capsule,'glca+hep-galfnac+omepn-ribf','GlcA').
contains_part(brendan,cje,capsule,'glca+hep+omepn-galfnac+omepn-ribf','GlcA').
contains_part(brendan,cje,capsule,'glca+hep+3ome-galfnac+omepn-ribf','GlcA').
contains_part(brendan,cje,capsule,'glca+hep+6ome-galfnac+omepn-ribf','GlcA').
contains_part(brendan,cje,capsule,'glca+hep+omepn+3ome-galfnac+omepn-ribf','GlcA').
contains_part(brendan,cje,capsule,'glca+hep+omepn+6ome-galfnac+omepn-ribf','GlcA').
contains_part(brendan,cje,capsule,'glca+hep+3ome+6ome-galfnac+omepn-ribf','GlcA').
contains_part(brendan,cje,capsule,'glca+hep+omepn+3ome+6ome-galfnac+omepn-ribf','GlcA').

%% NGro

```
contains_part(brendan,cje,capsule,'glca6ngro','NGro').
contains_part(brendan,cje,capsule,'glca6ngro+hep','NGro').
contains_part(brendan,cje,capsule,'glca6ngro+hep+omepn','NGro').
contains_part(brendan,cje,capsule,'glca6ngro+hep+3ome','NGro').
contains_part(brendan,cje,capsule,'glca6ngro+hep+6ome','NGro').
contains_part(brendan,cje,capsule,'glca6ngro+hep+omepn+3ome','NGro').
contains_part(brendan,cje,capsule,'glca6ngro+hep+omepn+6ome','NGro').
contains_part(brendan,cje,capsule,'glca6ngro+hep+3ome+6ome','NGro').
contains_part(brendan,cje,capsule,'glca6ngro+hep+omepn+3ome+6ome','NGro').
contains_part(brendan,cje,capsule,'glca6ngro-galfnac','NGro').
contains_part(brendan,cje,capsule,'glca6ngro+hep-galfnac','NGro').
contains_part(brendan,cje,capsule,'glca6ngro+hep+omepn-galfnac','NGro').
contains_part(brendan,cje,capsule,'glca6ngro+hep+3ome-galfnac','NGro').
contains_part(brendan,cje,capsule,'glca6ngro+hep+6ome-galfnac','NGro').
contains_part(brendan,cje,capsule,'glca6ngro+hep+omepn+3ome-galfnac','NGro').
contains_part(brendan,cje,capsule,'glca6ngro+hep+omepn+6ome-galfnac','NGro').
contains_part(brendan,cje,capsule,'glca6ngro+hep+3ome+6ome-galfnac','NGro').
contains_part(brendan,cje,capsule,'glca6ngro+hep+omepn+3ome+6ome-galfnac','NGro').
contains_part(brendan,cje,capsule,'glca6ngro-galfnac+omepn','NGro').
contains_part(brendan,cje,capsule,'glca6ngro+hep-galfnac+omepn','NGro').
contains_part(brendan,cje,capsule,'glca6ngro+hep+omepn-galfnac+omepn','NGro').
contains_part(brendan,cje,capsule,'glca6ngro+hep+3ome-galfnac+omepn','NGro').
contains_part(brendan,cje,capsule,'glca6ngro+hep+6ome-galfnac+omepn','NGro').
contains_part(brendan,cje,capsule,'glca6ngro+hep+omepn+3ome-galfnac+omepn','NGro').
contains_part(brendan,cje,capsule,'glca6ngro+hep+omepn+6ome-galfnac+omepn','NGro').
contains_part(brendan,cje,capsule,'glca6ngro+hep+3ome+6ome-galfnac+omepn','NGro').
contains_part(brendan,cje,capsule,'glca6ngro+hep+omepn+3ome+6ome-galfnac+omepn','NGro').
contains_part(brendan,cje,capsule,'glca6ngro-galfnac-ribf','NGro').
contains_part(brendan,cje,capsule,'glca6ngro+hep-galfnac-ribf','NGro').
contains_part(brendan,cje,capsule,'glca6ngro+hep+omepn-galfnac-ribf','NGro').
contains_part(brendan,cje,capsule,'glca6ngro+hep+3ome-galfnac-ribf','NGro').
contains_part(brendan,cje,capsule,'glca6ngro+hep+6ome-galfnac-ribf','NGro').
contains_part(brendan,cje,capsule,'glca6ngro+hep+omepn+3ome-galfnac-ribf','NGro').
contains_part(brendan,cje,capsule,'glca6ngro+hep+omepn+6ome-galfnac-ribf','NGro').
contains_part(brendan,cje,capsule,'glca6ngro+hep+3ome+6ome-galfnac-ribf','NGro').
contains_part(brendan,cje,capsule,'glca6ngro+hep+omepn+3ome+6ome-galfnac-ribf','NGro').
contains_part(brendan,cje,capsule,'glca6ngro-galfnac+omepn-ribf','NGro').
contains_part(brendan,cje,capsule,'glca6ngro+hep-galfnac+omepn-ribf','NGro').
contains_part(brendan,cje,capsule,'glca6ngro+hep+omepn-galfnac+omepn-ribf','NGro').
contains_part(brendan,cje,capsule,'glca6ngro+hep+3ome-galfnac+omepn-ribf','NGro').
contains_part(brendan,cje,capsule,'glca6ngro+hep+6ome-galfnac+omepn-ribf','NGro').
contains_part(brendan,cje,capsule,'glca6ngro+hep+omepn+3ome-galfnac+omepn-ribf','NGro').
contains_part(brendan,cje,capsule,'glca6ngro+hep+omepn+6ome-galfnac+omepn-ribf','NGro').
contains_part(brendan,cje,capsule,'glca6ngro+hep+3ome+6ome-galfnac+omepn-ribf','NGro').
contains_part(brendan,cje,capsule,'glca6ngro+hep+omepn+3ome+6ome-galfnac+omepn-ribf','NGro').

%% EtN

contains_part(brendan,cje,capsule,'glca6etn','EtN').
contains_part(brendan,cje,capsule,'glca6etn+hep','EtN').
contains_part(brendan,cje,capsule,'glca6etn+hep+omepn','EtN').
contains_part(brendan,cje,capsule,'glca6etn+hep+3ome','EtN').
contains_part(brendan,cje,capsule,'glca6etn+hep+6ome','EtN').
```

```
contains_part(brendan,cje,capsule,'glca6etn+hep+omepn+3ome','EtN').
contains_part(brendan,cje,capsule,'glca6etn+hep+omepn+6ome','EtN').
contains_part(brendan,cje,capsule,'glca6etn+hep+3ome+6ome','EtN').
contains_part(brendan,cje,capsule,'glca6etn+hep+omepn+3ome+6ome','EtN').
contains_part(brendan,cje,capsule,'glca6etn-galfnac','EtN').
contains_part(brendan,cje,capsule,'glca6etn+hep-galfnac','EtN').
contains_part(brendan,cje,capsule,'glca6etn+hep+omepn-galfnac','EtN').
contains_part(brendan,cje,capsule,'glca6etn+hep+3ome-galfnac','EtN').
contains_part(brendan,cje,capsule,'glca6etn+hep+6ome-galfnac','EtN').
contains_part(brendan,cje,capsule,'glca6etn+hep+omepn+3ome-galfnac','EtN').
contains_part(brendan,cje,capsule,'glca6etn+hep+omepn+6ome-galfnac','EtN').
contains_part(brendan,cje,capsule,'glca6etn+hep+3ome+6ome-galfnac','EtN').
contains_part(brendan,cje,capsule,'glca6etn+hep+omepn+3ome+6ome-galfnac','EtN').
contains_part(brendan,cje,capsule,'glca6etn-galfnac+omepn','EtN').
contains_part(brendan,cje,capsule,'glca6etn+hep-galfnac+omepn','EtN').
contains_part(brendan,cje,capsule,'glca6etn+hep+omepn-galfnac+omepn','EtN').
contains_part(brendan,cje,capsule,'glca6etn+hep+3ome-galfnac+omepn','EtN').
contains_part(brendan,cje,capsule,'glca6etn+hep+6ome-galfnac+omepn','EtN').
contains_part(brendan,cje,capsule,'glca6etn+hep+omepn+3ome-galfnac+omepn','EtN').
contains_part(brendan,cje,capsule,'glca6etn+hep+omepn+6ome-galfnac+omepn','EtN').
contains_part(brendan,cje,capsule,'glca6etn+hep+3ome+6ome-galfnac+omepn','EtN').
contains_part(brendan,cje,capsule,'glca6etn+hep+omepn+3ome+6ome-galfnac+omepn','EtN').
contains_part(brendan,cje,capsule,'glca6etn-galfnac-ribf','EtN').
contains_part(brendan,cje,capsule,'glca6etn+hep-galfnac-ribf','EtN').
contains_part(brendan,cje,capsule,'glca6etn+hep+omepn-galfnac-ribf','EtN').
contains_part(brendan,cje,capsule,'glca6etn+hep+3ome-galfnac-ribf','EtN').
contains_part(brendan,cje,capsule,'glca6etn+hep+6ome-galfnac-ribf','EtN').
contains_part(brendan,cje,capsule,'glca6etn+hep+omepn+3ome-galfnac-ribf','EtN').
contains_part(brendan,cje,capsule,'glca6etn+hep+omepn+6ome-galfnac-ribf','EtN').
contains_part(brendan,cje,capsule,'glca6etn+hep+3ome+6ome-galfnac-ribf','EtN').
contains_part(brendan,cje,capsule,'glca6etn+hep+omepn+3ome+6ome-galfnac-ribf','EtN').
contains_part(brendan,cje,capsule,'glca6etn-galfnac+omepn-ribf','EtN').
contains_part(brendan,cje,capsule,'glca6etn+hep-galfnac+omepn-ribf','EtN').
contains_part(brendan,cje,capsule,'glca6etn+hep+omepn-galfnac+omepn-ribf','EtN').
contains_part(brendan,cje,capsule,'glca6etn+hep+3ome-galfnac+omepn-ribf','EtN').
contains_part(brendan,cje,capsule,'glca6etn+hep+6ome-galfnac+omepn-ribf','EtN').
contains_part(brendan,cje,capsule,'glca6etn+hep+omepn+3ome-galfnac+omepn-ribf','EtN').
contains_part(brendan,cje,capsule,'glca6etn+hep+omepn+6ome-galfnac+omepn-ribf','EtN').
contains_part(brendan,cje,capsule,'glca6etn+hep+3ome+6ome-galfnac+omepn-ribf','EtN').
contains_part(brendan,cje,capsule,'glca6etn+hep+omepn+3ome+6ome-galfnac+omepn-ribf','EtN').

%% Hep

contains_part(brendan,cje,capsule,'glca6ngro+hep','Hep').
contains_part(brendan,cje,capsule,'glca6ngro+hep+omepn','Hep').
contains_part(brendan,cje,capsule,'glca6ngro+hep+3ome','Hep').
contains_part(brendan,cje,capsule,'glca6ngro+hep+6ome','Hep').
contains_part(brendan,cje,capsule,'glca6ngro+hep+omepn+3ome','Hep').
contains_part(brendan,cje,capsule,'glca6ngro+hep+omepn+6ome','Hep').
contains_part(brendan,cje,capsule,'glca6ngro+hep+3ome+6ome','Hep').
contains_part(brendan,cje,capsule,'glca6ngro+hep+omepn+3ome+6ome','Hep').
contains_part(brendan,cje,capsule,'glca6ngro+hep-galfnac','Hep').
contains_part(brendan,cje,capsule,'glca6ngro+hep+omepn-galfnac','Hep').
contains_part(brendan,cje,capsule,'glca6ngro+hep+3ome-galfnac','Hep').
```

```
contains_part(brendan,cje,capsule,'glca6ngro+hep+6ome-galfnac','Hep').
contains_part(brendan,cje,capsule,'glca6ngro+hep+omepn+3ome-galfnac','Hep').
contains_part(brendan,cje,capsule,'glca6ngro+hep+omepn+6ome-galfnac','Hep').
contains_part(brendan,cje,capsule,'glca6ngro+hep+3ome+6ome-galfnac','Hep').
contains_part(brendan,cje,capsule,'glca6ngro+hep+omepn+3ome+6ome-galfnac','Hep').
contains_part(brendan,cje,capsule,'glca6ngro+hep-galfnac+omepn','Hep').
contains_part(brendan,cje,capsule,'glca6ngro+hep+omepn-galfnac+omepn','Hep').
contains_part(brendan,cje,capsule,'glca6ngro+hep+3ome-galfnac+omepn','Hep').
contains_part(brendan,cje,capsule,'glca6ngro+hep+6ome-galfnac+omepn','Hep').
contains_part(brendan,cje,capsule,'glca6ngro+hep+omepn+3ome-galfnac+omepn','Hep').
contains_part(brendan,cje,capsule,'glca6ngro+hep+omepn+6ome-galfnac+omepn','Hep').
contains_part(brendan,cje,capsule,'glca6ngro+hep+3ome+6ome-galfnac+omepn','Hep').
contains_part(brendan,cje,capsule,'glca6ngro+hep+omepn+3ome+6ome-galfnac+omepn','Hep').
contains_part(brendan,cje,capsule,'glca6ngro+hep-galfnac-ribf','Hep').
contains_part(brendan,cje,capsule,'glca6ngro+hep+omepn-galfnac-ribf','Hep').
contains_part(brendan,cje,capsule,'glca6ngro+hep+3ome-galfnac-ribf','Hep').
contains_part(brendan,cje,capsule,'glca6ngro+hep+6ome-galfnac-ribf','Hep').
contains_part(brendan,cje,capsule,'glca6ngro+hep+omepn+3ome-galfnac-ribf','Hep').
contains_part(brendan,cje,capsule,'glca6ngro+hep+omepn+6ome-galfnac-ribf','Hep').
contains_part(brendan,cje,capsule,'glca6ngro+hep+3ome+6ome-galfnac-ribf','Hep').
contains_part(brendan,cje,capsule,'glca6ngro+hep+omepn+3ome+6ome-galfnac-ribf','Hep').
contains_part(brendan,cje,capsule,'glca6ngro+hep-galfnac+omepn-ribf','Hep').
contains_part(brendan,cje,capsule,'glca6ngro+hep+omepn-galfnac+omepn-ribf','Hep').
contains_part(brendan,cje,capsule,'glca6ngro+hep+3ome-galfnac+omepn-ribf','Hep').
contains_part(brendan,cje,capsule,'glca6ngro+hep+6ome-galfnac+omepn-ribf','Hep').
contains_part(brendan,cje,capsule,'glca6ngro+hep+omepn+3ome-galfnac+omepn-ribf','Hep').
contains_part(brendan,cje,capsule,'glca6ngro+hep+omepn+6ome-galfnac+omepn-ribf','Hep').
contains_part(brendan,cje,capsule,'glca6ngro+hep+3ome+6ome-galfnac+omepn-ribf','Hep').
contains_part(brendan,cje,capsule,'glca6ngro+hep+omepn+3ome+6ome-galfnac+omepn-ribf','Hep').
contains_part(brendan,cje,capsule,'glca6etn+hep','Hep').
contains_part(brendan,cje,capsule,'glca6etn+hep+omepn','Hep').
contains_part(brendan,cje,capsule,'glca6etn+hep+3ome','Hep').
contains_part(brendan,cje,capsule,'glca6etn+hep+6ome','Hep').
contains_part(brendan,cje,capsule,'glca6etn+hep+omepn+3ome','Hep').
contains_part(brendan,cje,capsule,'glca6etn+hep+omepn+6ome','Hep').
contains_part(brendan,cje,capsule,'glca6etn+hep+3ome+6ome','Hep').
contains_part(brendan,cje,capsule,'glca6etn+hep+omepn+3ome+6ome','Hep').
contains_part(brendan,cje,capsule,'glca6etn+hep-galfnac','Hep').
contains_part(brendan,cje,capsule,'glca6etn+hep+omepn-galfnac','Hep').
contains_part(brendan,cje,capsule,'glca6etn+hep+3ome-galfnac','Hep').
contains_part(brendan,cje,capsule,'glca6etn+hep+6ome-galfnac','Hep').
contains_part(brendan,cje,capsule,'glca6etn+hep+omepn+3ome-galfnac','Hep').
contains_part(brendan,cje,capsule,'glca6etn+hep+omepn+6ome-galfnac','Hep').
contains_part(brendan,cje,capsule,'glca6etn+hep+3ome+6ome-galfnac','Hep').
contains_part(brendan,cje,capsule,'glca6etn+hep+omepn+3ome+6ome-galfnac','Hep').
contains_part(brendan,cje,capsule,'glca6etn+hep-galfnac+omepn','Hep').
contains_part(brendan,cje,capsule,'glca6etn+hep+omepn-galfnac+omepn','Hep').
contains_part(brendan,cje,capsule,'glca6etn+hep+3ome-galfnac+omepn','Hep').
contains_part(brendan,cje,capsule,'glca6etn+hep+6ome-galfnac+omepn','Hep').
contains_part(brendan,cje,capsule,'glca6etn+hep+omepn+3ome-galfnac+omepn','Hep').
contains_part(brendan,cje,capsule,'glca6etn+hep+omepn+6ome-galfnac+omepn','Hep').
contains_part(brendan,cje,capsule,'glca6etn+hep+3ome+6ome-galfnac+omepn','Hep').
contains_part(brendan,cje,capsule,'glca6etn+hep+omepn+3ome+6ome-galfnac+omepn','Hep').
contains_part(brendan,cje,capsule,'glca6etn+hep-galfnac-ribf','Hep').
```

contains_part(brendan,cje,capsule,'glca6etn+hep+omepn-galfnac-ribf','Hep').
contains_part(brendan,cje,capsule,'glca6etn+hep+3ome-galfnac-ribf','Hep').
contains_part(brendan,cje,capsule,'glca6etn+hep+6ome-galfnac-ribf','Hep').
contains_part(brendan,cje,capsule,'glca6etn+hep+omepn+3ome-galfnac-ribf','Hep').
contains_part(brendan,cje,capsule,'glca6etn+hep+omepn+6ome-galfnac-ribf','Hep').
contains_part(brendan,cje,capsule,'glca6etn+hep+3ome+6ome-galfnac-ribf','Hep').
contains_part(brendan,cje,capsule,'glca6etn+hep+omepn+3ome+6ome-galfnac-ribf','Hep').
contains_part(brendan,cje,capsule,'glca6etn+hep-galfnac+omepn-ribf','Hep').
contains_part(brendan,cje,capsule,'glca6etn+hep+omepn-galfnac+omepn-ribf','Hep').
contains_part(brendan,cje,capsule,'glca6etn+hep+3ome-galfnac+omepn-ribf','Hep').
contains_part(brendan,cje,capsule,'glca6etn+hep+6ome-galfnac+omepn-ribf','Hep').
contains_part(brendan,cje,capsule,'glca6etn+hep+omepn+3ome-galfnac+omepn-ribf','Hep').
contains_part(brendan,cje,capsule,'glca6etn+hep+omepn+6ome-galfnac+omepn-ribf','Hep').
contains_part(brendan,cje,capsule,'glca6etn+hep+3ome+6ome-galfnac+omepn-ribf','Hep').
contains_part(brendan,cje,capsule,'glca6etn+hep+omepn+3ome+6ome-galfnac+omepn-ribf','Hep').

%% Hep OMePN

contains_part(brendan,cje,capsule,'glca6ngro+hep+omepn','Hep OMePN').
contains_part(brendan,cje,capsule,'glca6ngro+hep+omepn+3ome','Hep OMePN').
contains_part(brendan,cje,capsule,'glca6ngro+hep+omepn+6ome','Hep OMePN').
contains_part(brendan,cje,capsule,'glca6ngro+hep+omepn+3ome+6ome','Hep OMePN').
contains_part(brendan,cje,capsule,'glca6ngro+hep+omepn-galfnac','Hep OMePN').
contains_part(brendan,cje,capsule,'glca6ngro+hep+omepn+3ome-galfnac','Hep OMePN').
contains_part(brendan,cje,capsule,'glca6ngro+hep+omepn+6ome-galfnac','Hep OMePN').
contains_part(brendan,cje,capsule,'glca6ngro+hep+omepn+3ome+6ome-galfnac','Hep OMePN').
contains_part(brendan,cje,capsule,'glca6ngro+hep+omepn-galfnac+omepn','Hep OMePN').
contains_part(brendan,cje,capsule,'glca6ngro+hep+omepn+3ome-galfnac+omepn','Hep OMePN').
contains_part(brendan,cje,capsule,'glca6ngro+hep+omepn+6ome-galfnac+omepn','Hep OMePN').
contains_part(brendan,cje,capsule,'glca6ngro+hep+omepn+3ome+6ome-galfnac+omepn','Hep OMePN').
contains_part(brendan,cje,capsule,'glca6ngro+hep+omepn-galfnac-ribf','Hep OMePN').
contains_part(brendan,cje,capsule,'glca6ngro+hep+omepn+3ome-galfnac-ribf','Hep OMePN').
contains_part(brendan,cje,capsule,'glca6ngro+hep+omepn+6ome-galfnac-ribf','Hep OMePN').
contains_part(brendan,cje,capsule,'glca6ngro+hep+omepn+3ome+6ome-galfnac-ribf','Hep OMePN').
contains_part(brendan,cje,capsule,'glca6ngro+hep+omepn-galfnac+omepn-ribf','Hep OMePN').
contains_part(brendan,cje,capsule,'glca6ngro+hep+omepn+3ome-galfnac+omepn-ribf','Hep OMePN').
contains_part(brendan,cje,capsule,'glca6ngro+hep+omepn+6ome-galfnac+omepn-ribf','Hep OMePN').
contains_part(brendan,cje,capsule,'glca6ngro+hep+omepn+3ome+6ome-galfnac+omepn-ribf','Hep OMePN').
contains_part(brendan,cje,capsule,'glca6etn+hep+omepn','Hep OMePN').
contains_part(brendan,cje,capsule,'glca6etn+hep+omepn+3ome','Hep OMePN').
contains_part(brendan,cje,capsule,'glca6etn+hep+omepn+6ome','Hep OMePN').
contains_part(brendan,cje,capsule,'glca6etn+hep+omepn+3ome+6ome','Hep OMePN').
contains_part(brendan,cje,capsule,'glca6etn+hep+omepn-galfnac','Hep OMePN').
contains_part(brendan,cje,capsule,'glca6etn+hep+omepn+3ome-galfnac','Hep OMePN').
contains_part(brendan,cje,capsule,'glca6etn+hep+omepn+6ome-galfnac','Hep OMePN').
contains_part(brendan,cje,capsule,'glca6etn+hep+omepn+3ome+6ome-galfnac','Hep OMePN').
contains_part(brendan,cje,capsule,'glca6etn+hep+omepn-galfnac+omepn','Hep OMePN').
contains_part(brendan,cje,capsule,'glca6etn+hep+omepn+3ome-galfnac+omepn','Hep OMePN').
contains_part(brendan,cje,capsule,'glca6etn+hep+omepn+6ome-galfnac+omepn','Hep OMePN').
contains_part(brendan,cje,capsule,'glca6etn+hep+omepn+3ome+6ome-galfnac+omepn','Hep OMePN').
contains_part(brendan,cje,capsule,'glca6etn+hep+omepn-galfnac-ribf','Hep OMePN').
contains_part(brendan,cje,capsule,'glca6etn+hep+omepn+3ome-galfnac-ribf','Hep OMePN').
contains_part(brendan,cje,capsule,'glca6etn+hep+omepn+6ome-galfnac-ribf','Hep OMePN').
contains_part(brendan,cje,capsule,'glca6etn+hep+omepn+3ome+6ome-galfnac-ribf','Hep OMePN').

contains_part(brendan,cje,capsule,'glca6etn+hep+omepn-galfnac+omepn-ribf','Hep OMePN').
contains_part(brendan,cje,capsule,'glca6etn+hep+omepn+3ome-galfnac+omepn-ribf','Hep OMePN').
contains_part(brendan,cje,capsule,'glca6etn+hep+omepn+6ome-galfnac+omepn-ribf','Hep OMePN').
contains_part(brendan,cje,capsule,'glca6etn+hep+omepn+3ome+6ome-galfnac+omepn-ribf','Hep OMePN').

%% Hep 3-OMe

contains_part(brendan,cje,capsule,'glca6ngro+hep+3ome','Hep 3-OMe').
contains_part(brendan,cje,capsule,'glca6ngro+hep+omepn+3ome','Hep 3-OMe').
contains_part(brendan,cje,capsule,'glca6ngro+hep+3ome+6ome','Hep 3-OMe').
contains_part(brendan,cje,capsule,'glca6ngro+hep+omepn+3ome+6ome','Hep 3-OMe').
contains_part(brendan,cje,capsule,'glca6ngro+hep+3ome-galfnac','Hep 3-OMe').
contains_part(brendan,cje,capsule,'glca6ngro+hep+omepn+3ome-galfnac','Hep 3-OMe').
contains_part(brendan,cje,capsule,'glca6ngro+hep+3ome+6ome-galfnac','Hep 3-OMe').
contains_part(brendan,cje,capsule,'glca6ngro+hep+omepn+3ome+6ome-galfnac','Hep 3-OMe').
contains_part(brendan,cje,capsule,'glca6ngro+hep+3ome-galfnac+omepn','Hep 3-OMe').
contains_part(brendan,cje,capsule,'glca6ngro+hep+omepn+3ome-galfnac+omepn','Hep 3-OMe').
contains_part(brendan,cje,capsule,'glca6ngro+hep+3ome+6ome-galfnac+omepn','Hep 3-OMe').
contains_part(brendan,cje,capsule,'glca6ngro+hep+omepn+3ome+6ome-galfnac+omepn','Hep 3-OMe').
contains_part(brendan,cje,capsule,'glca6ngro+hep+3ome-galfnac-ribf','Hep 3-OMe').
contains_part(brendan,cje,capsule,'glca6ngro+hep+omepn+3ome-galfnac-ribf','Hep 3-OMe').
contains_part(brendan,cje,capsule,'glca6ngro+hep+3ome+6ome-galfnac-ribf','Hep 3-OMe').
contains_part(brendan,cje,capsule,'glca6ngro+hep+omepn+3ome+6ome-galfnac-ribf','Hep 3-OMe').
contains_part(brendan,cje,capsule,'glca6ngro+hep+3ome-galfnac+omepn-ribf','Hep 3-OMe').
contains_part(brendan,cje,capsule,'glca6ngro+hep+omepn+3ome-galfnac+omepn-ribf','Hep 3-OMe').
contains_part(brendan,cje,capsule,'glca6ngro+hep+3ome+6ome-galfnac+omepn-ribf','Hep 3-OMe').
contains_part(brendan,cje,capsule,'glca6ngro+hep+omepn+3ome+6ome-galfnac+omepn-ribf','Hep 3-OMe').
contains_part(brendan,cje,capsule,'glca6etn+hep+3ome','Hep 3-OMe').
contains_part(brendan,cje,capsule,'glca6etn+hep+omepn+3ome','Hep 3-OMe').
contains_part(brendan,cje,capsule,'glca6etn+hep+3ome+6ome','Hep 3-OMe').
contains_part(brendan,cje,capsule,'glca6etn+hep+omepn+3ome+6ome','Hep 3-OMe').
contains_part(brendan,cje,capsule,'glca6etn+hep+3ome-galfnac','Hep 3-OMe').
contains_part(brendan,cje,capsule,'glca6etn+hep+omepn+3ome-galfnac','Hep 3-OMe').
contains_part(brendan,cje,capsule,'glca6etn+hep+3ome+6ome-galfnac','Hep 3-OMe').
contains_part(brendan,cje,capsule,'glca6etn+hep+omepn+3ome+6ome-galfnac','Hep 3-OMe').
contains_part(brendan,cje,capsule,'glca6etn+hep+3ome-galfnac+omepn','Hep 3-OMe').
contains_part(brendan,cje,capsule,'glca6etn+hep+omepn+3ome-galfnac+omepn','Hep 3-OMe').
contains_part(brendan,cje,capsule,'glca6etn+hep+3ome+6ome-galfnac+omepn','Hep 3-OMe').
contains_part(brendan,cje,capsule,'glca6etn+hep+omepn+3ome+6ome-galfnac+omepn','Hep 3-OMe').
contains_part(brendan,cje,capsule,'glca6etn+hep+3ome-galfnac-ribf','Hep 3-OMe').
contains_part(brendan,cje,capsule,'glca6etn+hep+omepn+3ome-galfnac-ribf','Hep 3-OMe').
contains_part(brendan,cje,capsule,'glca6etn+hep+3ome+6ome-galfnac-ribf','Hep 3-OMe').
contains_part(brendan,cje,capsule,'glca6etn+hep+omepn+3ome+6ome-galfnac-ribf','Hep 3-OMe').
contains_part(brendan,cje,capsule,'glca6etn+hep+3ome-galfnac+omepn-ribf','Hep 3-OMe').
contains_part(brendan,cje,capsule,'glca6etn+hep+omepn+3ome-galfnac+omepn-ribf','Hep 3-OMe').
contains_part(brendan,cje,capsule,'glca6etn+hep+3ome+6ome-galfnac+omepn-ribf','Hep 3-OMe').
contains_part(brendan,cje,capsule,'glca6etn+hep+omepn+3ome+6ome-galfnac+omepn-ribf','Hep 3-OMe').
contains_part(brendan,cje,capsule,'glca+hep+3ome','Hep 3-OMe').
contains_part(brendan,cje,capsule,'glca+hep+omepn+3ome','Hep 3-OMe').
contains_part(brendan,cje,capsule,'glca+hep+3ome+6ome','Hep 3-OMe').
contains_part(brendan,cje,capsule,'glca+hep+omepn+3ome+6ome','Hep 3-OMe').
contains_part(brendan,cje,capsule,'glca+hep+3ome-galfnac','Hep 3-OMe').
contains_part(brendan,cje,capsule,'glca+hep+omepn+3ome-galfnac','Hep 3-OMe').
contains_part(brendan,cje,capsule,'glca+hep+3ome+6ome-galfnac','Hep 3-OMe').

```
contains_part(brendan,cje,capsule,'glca+hep+omepn+3ome+6ome-galfnac','Hep 3-OMe').
contains_part(brendan,cje,capsule,'glca+hep+3ome-galfnac+omepn','Hep 3-OMe').
contains_part(brendan,cje,capsule,'glca+hep+omepn+3ome-galfnac+omepn','Hep 3-OMe').
contains_part(brendan,cje,capsule,'glca+hep+3ome+6ome-galfnac+omepn','Hep 3-OMe').
contains_part(brendan,cje,capsule,'glca+hep+omepn+3ome+6ome-galfnac+omepn','Hep 3-OMe').
contains_part(brendan,cje,capsule,'glca+hep+3ome-galfnac-ribf','Hep 3-OMe').
contains_part(brendan,cje,capsule,'glca+hep+omepn+3ome-galfnac-ribf','Hep 3-OMe').
contains_part(brendan,cje,capsule,'glca+hep+3ome+6ome-galfnac-ribf','Hep 3-OMe').
contains_part(brendan,cje,capsule,'glca+hep+omepn+3ome+6ome-galfnac-ribf','Hep 3-OMe').
contains_part(brendan,cje,capsule,'glca+hep+3ome-galfnac+omepn-ribf','Hep 3-OMe').
contains_part(brendan,cje,capsule,'glca+hep+omepn+3ome-galfnac+omepn-ribf','Hep 3-OMe').
contains_part(brendan,cje,capsule,'glca+hep+3ome+6ome-galfnac+omepn-ribf','Hep 3-OMe').
contains_part(brendan,cje,capsule,'glca+hep+omepn+3ome+6ome-galfnac+omepn-ribf','Hep 3-OMe').

%% Hep 6-Ome

contains_part(brendan,cje,capsule,'glca6ngro+hep+6ome','Hep 6-OMe').
contains_part(brendan,cje,capsule,'glca6ngro+hep+omepn+6ome','Hep 6-OMe').
contains_part(brendan,cje,capsule,'glca6ngro+hep+3ome+6ome','Hep 6-OMe').
contains_part(brendan,cje,capsule,'glca6ngro+hep+omepn+3ome+6ome','Hep 6-OMe').
contains_part(brendan,cje,capsule,'glca6ngro+hep+6ome-galfnac','Hep 6-OMe').
contains_part(brendan,cje,capsule,'glca6ngro+hep+omepn+6ome-galfnac','Hep 6-OMe').
contains_part(brendan,cje,capsule,'glca6ngro+hep+3ome+6ome-galfnac','Hep 6-OMe').
contains_part(brendan,cje,capsule,'glca6ngro+hep+omepn+3ome+6ome-galfnac','Hep 6-OMe').
contains_part(brendan,cje,capsule,'glca6ngro+hep+6ome-galfnac+omepn','Hep 6-OMe').
contains_part(brendan,cje,capsule,'glca6ngro+hep+omepn+6ome-galfnac+omepn','Hep 6-OMe').
contains_part(brendan,cje,capsule,'glca6ngro+hep+3ome+6ome-galfnac+omepn','Hep 6-OMe').
contains_part(brendan,cje,capsule,'glca6ngro+hep+omepn+3ome+6ome-galfnac+omepn','Hep 6-OMe').
contains_part(brendan,cje,capsule,'glca6ngro+hep+6ome-galfnac-ribf','Hep 6-OMe').
contains_part(brendan,cje,capsule,'glca6ngro+hep+omepn+6ome-galfnac-ribf','Hep 6-OMe').
contains_part(brendan,cje,capsule,'glca6ngro+hep+3ome+6ome-galfnac-ribf','Hep 6-OMe').
contains_part(brendan,cje,capsule,'glca6ngro+hep+omepn+3ome+6ome-galfnac-ribf','Hep 6-OMe').
contains_part(brendan,cje,capsule,'glca6ngro+hep+6ome-galfnac+omepn-ribf','Hep 6-OMe').
contains_part(brendan,cje,capsule,'glca6ngro+hep+omepn+6ome-galfnac+omepn-ribf','Hep 6-OMe').
contains_part(brendan,cje,capsule,'glca6ngro+hep+3ome+6ome-galfnac+omepn-ribf','Hep 6-OMe').
contains_part(brendan,cje,capsule,'glca6ngro+hep+omepn+3ome+6ome-galfnac+omepn-ribf','Hep 6-OMe').
contains_part(brendan,cje,capsule,'glca6etn+hep+6ome','Hep 6-OMe').
contains_part(brendan,cje,capsule,'glca6etn+hep+omepn+6ome','Hep 6-OMe').
contains_part(brendan,cje,capsule,'glca6etn+hep+3ome+6ome','Hep 6-OMe').
contains_part(brendan,cje,capsule,'glca6etn+hep+omepn+3ome+6ome','Hep 6-OMe').
contains_part(brendan,cje,capsule,'glca6etn+hep+6ome-galfnac','Hep 6-OMe').
contains_part(brendan,cje,capsule,'glca6etn+hep+omepn+6ome-galfnac','Hep 6-OMe').
contains_part(brendan,cje,capsule,'glca6etn+hep+3ome+6ome-galfnac','Hep 6-OMe').
contains_part(brendan,cje,capsule,'glca6etn+hep+omepn+3ome+6ome-galfnac','Hep 6-OMe').
contains_part(brendan,cje,capsule,'glca6etn+hep+6ome-galfnac+omepn','Hep 6-OMe').
contains_part(brendan,cje,capsule,'glca6etn+hep+omepn+6ome-galfnac+omepn','Hep 6-OMe').
contains_part(brendan,cje,capsule,'glca6etn+hep+3ome+6ome-galfnac+omepn','Hep 6-OMe').
contains_part(brendan,cje,capsule,'glca6etn+hep+omepn+3ome+6ome-galfnac+omepn','Hep 6-OMe').
contains_part(brendan,cje,capsule,'glca6etn+hep+6ome-galfnac-ribf','Hep 6-OMe').
contains_part(brendan,cje,capsule,'glca6etn+hep+omepn+6ome-galfnac-ribf','Hep 6-OMe').
contains_part(brendan,cje,capsule,'glca6etn+hep+3ome+6ome-galfnac-ribf','Hep 6-OMe').
contains_part(brendan,cje,capsule,'glca6etn+hep+omepn+3ome+6ome-galfnac-ribf','Hep 6-OMe').
contains_part(brendan,cje,capsule,'glca6etn+hep+6ome-galfnac+omepn-ribf','Hep 6-OMe').
contains_part(brendan,cje,capsule,'glca6etn+hep+omepn+6ome-galfnac+omepn-ribf','Hep 6-OMe').
```

```
contains_part(brendan,cje,capsule,'glca6etn+hep+3ome+6ome-galfnac+omepn-ribf','Hep 6-OMe').
contains_part(brendan,cje,capsule,'glca6etn+hep+omepn+3ome+6ome-galfnac+omepn-ribf','Hep 6-OMe').
contains_part(brendan,cje,capsule,'glca+hep+6ome','Hep 6-OMe').
contains_part(brendan,cje,capsule,'glca+hep+omepn+6ome','Hep 6-OMe').
contains_part(brendan,cje,capsule,'glca+hep+3ome+6ome','Hep 6-OMe').
contains_part(brendan,cje,capsule,'glca+hep+omepn+3ome+6ome','Hep 6-OMe').
contains_part(brendan,cje,capsule,'glca+hep+6ome-galfnac','Hep 6-OMe').
contains_part(brendan,cje,capsule,'glca+hep+omepn+6ome-galfnac','Hep 6-OMe').
contains_part(brendan,cje,capsule,'glca+hep+3ome+6ome-galfnac','Hep 6-OMe').
contains_part(brendan,cje,capsule,'glca+hep+omepn+3ome+6ome-galfnac','Hep 6-OMe').
contains_part(brendan,cje,capsule,'glca+hep+6ome-galfnac+omepn','Hep 6-OMe').
contains_part(brendan,cje,capsule,'glca+hep+omepn+6ome-galfnac+omepn','Hep 6-OMe').
contains_part(brendan,cje,capsule,'glca+hep+3ome+6ome-galfnac+omepn','Hep 6-OMe').
contains_part(brendan,cje,capsule,'glca+hep+omepn+3ome+6ome-galfnac+omepn','Hep 6-OMe').
contains_part(brendan,cje,capsule,'glca+hep+6ome-galfnac-ribf','Hep 6-OMe').
contains_part(brendan,cje,capsule,'glca+hep+omepn+6ome-galfnac-ribf','Hep 6-OMe').
contains_part(brendan,cje,capsule,'glca+hep+3ome+6ome-galfnac-ribf','Hep 6-OMe').
contains_part(brendan,cje,capsule,'glca+hep+omepn+3ome+6ome-galfnac-ribf','Hep 6-OMe').
contains_part(brendan,cje,capsule,'glca+hep+6ome-galfnac+omepn-ribf','Hep 6-OMe').
contains_part(brendan,cje,capsule,'glca+hep+omepn+6ome-galfnac+omepn-ribf','Hep 6-OMe').
contains_part(brendan,cje,capsule,'glca+hep+3ome+6ome-galfnac+omepn-ribf','Hep 6-OMe').
contains_part(brendan,cje,capsule,'glca+hep+omepn+3ome+6ome-galfnac+omepn-ribf','Hep 6-OMe').

%% GalfNAc

contains_part(brendan,cje,capsule,'glca6ngro-galfnac','GalfNAc').
contains_part(brendan,cje,capsule,'glca6ngro+hep-galfnac','GalfNAc').
contains_part(brendan,cje,capsule,'glca6ngro+hep+omepn-galfnac','GalfNAc').
contains_part(brendan,cje,capsule,'glca6ngro+hep+3ome-galfnac','GalfNAc').
contains_part(brendan,cje,capsule,'glca6ngro+hep+6ome-galfnac','GalfNAc').
contains_part(brendan,cje,capsule,'glca6ngro+hep+omepn+3ome-galfnac','GalfNAc').
contains_part(brendan,cje,capsule,'glca6ngro+hep+omepn+6ome-galfnac','GalfNAc').
contains_part(brendan,cje,capsule,'glca6ngro+hep+3ome+6ome-galfnac','GalfNAc').
contains_part(brendan,cje,capsule,'glca6ngro+hep+omepn+3ome+6ome-galfnac','GalfNAc').
contains_part(brendan,cje,capsule,'glca6ngro-galfnac+omepn','GalfNAc').
contains_part(brendan,cje,capsule,'glca6ngro+hep-galfnac+omepn','GalfNAc').
contains_part(brendan,cje,capsule,'glca6ngro+hep+omepn-galfnac+omepn','GalfNAc').
contains_part(brendan,cje,capsule,'glca6ngro+hep+3ome-galfnac+omepn','GalfNAc').
contains_part(brendan,cje,capsule,'glca6ngro+hep+6ome-galfnac+omepn','GalfNAc').
contains_part(brendan,cje,capsule,'glca6ngro+hep+omepn+3ome-galfnac+omepn','GalfNAc').
contains_part(brendan,cje,capsule,'glca6ngro+hep+omepn+6ome-galfnac+omepn','GalfNAc').
contains_part(brendan,cje,capsule,'glca6ngro+hep+3ome+6ome-galfnac+omepn','GalfNAc').
contains_part(brendan,cje,capsule,'glca6ngro+hep+omepn+3ome+6ome-galfnac+omepn','GalfNAc').
contains_part(brendan,cje,capsule,'glca6ngro-galfnac-ribf','GalfNAc').
contains_part(brendan,cje,capsule,'glca6ngro+hep-galfnac-ribf','GalfNAc').
contains_part(brendan,cje,capsule,'glca6ngro+hep+omepn-galfnac-ribf','GalfNAc').
contains_part(brendan,cje,capsule,'glca6ngro+hep+3ome-galfnac-ribf','GalfNAc').
contains_part(brendan,cje,capsule,'glca6ngro+hep+6ome-galfnac-ribf','GalfNAc').
contains_part(brendan,cje,capsule,'glca6ngro+hep+omepn+3ome-galfnac-ribf','GalfNAc').
contains_part(brendan,cje,capsule,'glca6ngro+hep+omepn+6ome-galfnac-ribf','GalfNAc').
contains_part(brendan,cje,capsule,'glca6ngro+hep+3ome+6ome-galfnac-ribf','GalfNAc').
contains_part(brendan,cje,capsule,'glca6ngro+hep+omepn+3ome+6ome-galfnac-ribf','GalfNAc').
contains_part(brendan,cje,capsule,'glca6ngro-galfnac+omepn-ribf','GalfNAc').
contains_part(brendan,cje,capsule,'glca6ngro+hep-galfnac+omepn-ribf','GalfNAc').
```

contains_part(brendan,cje,capsule,'glca6ngro+hep+omepn-galfnac+omepn-ribf','GalfNAc').
contains_part(brendan,cje,capsule,'glca6ngro+hep+3ome-galfnac+omepn-ribf','GalfNAc').
contains_part(brendan,cje,capsule,'glca6ngro+hep+6ome-galfnac+omepn-ribf','GalfNAc').
contains_part(brendan,cje,capsule,'glca6ngro+hep+omepn+3ome-galfnac+omepn-ribf','GalfNAc').
contains_part(brendan,cje,capsule,'glca6ngro+hep+omepn+6ome-galfnac+omepn-ribf','GalfNAc').
contains_part(brendan,cje,capsule,'glca6ngro+hep+3ome+6ome-galfnac+omepn-ribf','GalfNAc').
contains_part(brendan,cje,capsule,'glca6ngro+hep+omepn+3ome+6ome-galfnac+omepn-ribf','GalfNAc').
contains_part(brendan,cje,capsule,'glca6etn-galfnac','GalfNAc').
contains_part(brendan,cje,capsule,'glca6etn+hep-galfnac','GalfNAc').
contains_part(brendan,cje,capsule,'glca6etn+hep+omepn-galfnac','GalfNAc').
contains_part(brendan,cje,capsule,'glca6etn+hep+3ome-galfnac','GalfNAc').
contains_part(brendan,cje,capsule,'glca6etn+hep+6ome-galfnac','GalfNAc').
contains_part(brendan,cje,capsule,'glca6etn+hep+omepn+3ome-galfnac','GalfNAc').
contains_part(brendan,cje,capsule,'glca6etn+hep+omepn+6ome-galfnac','GalfNAc').
contains_part(brendan,cje,capsule,'glca6etn+hep+3ome+6ome-galfnac','GalfNAc').
contains_part(brendan,cje,capsule,'glca6etn+hep+omepn+3ome+6ome-galfnac','GalfNAc').
contains_part(brendan,cje,capsule,'glca6etn-galfnac+omepn','GalfNAc').
contains_part(brendan,cje,capsule,'glca6etn+hep-galfnac+omepn','GalfNAc').
contains_part(brendan,cje,capsule,'glca6etn+hep+omepn-galfnac+omepn','GalfNAc').
contains_part(brendan,cje,capsule,'glca6etn+hep+3ome-galfnac+omepn','GalfNAc').
contains_part(brendan,cje,capsule,'glca6etn+hep+6ome-galfnac+omepn','GalfNAc').
contains_part(brendan,cje,capsule,'glca6etn+hep+omepn+3ome-galfnac+omepn','GalfNAc').
contains_part(brendan,cje,capsule,'glca6etn+hep+omepn+6ome-galfnac+omepn','GalfNAc').
contains_part(brendan,cje,capsule,'glca6etn+hep+3ome+6ome-galfnac+omepn','GalfNAc').
contains_part(brendan,cje,capsule,'glca6etn+hep+omepn+3ome+6ome-galfnac+omepn','GalfNAc').
contains_part(brendan,cje,capsule,'glca6etn-galfnac-ribf','GalfNAc').
contains_part(brendan,cje,capsule,'glca6etn+hep-galfnac-ribf','GalfNAc').
contains_part(brendan,cje,capsule,'glca6etn+hep+omepn-galfnac-ribf','GalfNAc').
contains_part(brendan,cje,capsule,'glca6etn+hep+3ome-galfnac-ribf','GalfNAc').
contains_part(brendan,cje,capsule,'glca6etn+hep+6ome-galfnac-ribf','GalfNAc').
contains_part(brendan,cje,capsule,'glca6etn+hep+omepn+3ome-galfnac-ribf','GalfNAc').
contains_part(brendan,cje,capsule,'glca6etn+hep+omepn+6ome-galfnac-ribf','GalfNAc').
contains_part(brendan,cje,capsule,'glca6etn+hep+3ome+6ome-galfnac-ribf','GalfNAc').
contains_part(brendan,cje,capsule,'glca6etn+hep+omepn+3ome+6ome-galfnac-ribf','GalfNAc').
contains_part(brendan,cje,capsule,'glca6etn-galfnac+omepn-ribf','GalfNAc').
contains_part(brendan,cje,capsule,'glca6etn+hep-galfnac+omepn-ribf','GalfNAc').
contains_part(brendan,cje,capsule,'glca6etn+hep+omepn-galfnac+omepn-ribf','GalfNAc').
contains_part(brendan,cje,capsule,'glca6etn+hep+3ome-galfnac+omepn-ribf','GalfNAc').
contains_part(brendan,cje,capsule,'glca6etn+hep+6ome-galfnac+omepn-ribf','GalfNAc').
contains_part(brendan,cje,capsule,'glca6etn+hep+omepn+3ome-galfnac+omepn-ribf','GalfNAc').
contains_part(brendan,cje,capsule,'glca6etn+hep+omepn+6ome-galfnac+omepn-ribf','GalfNAc').
contains_part(brendan,cje,capsule,'glca6etn+hep+3ome+6ome-galfnac+omepn-ribf','GalfNAc').
contains_part(brendan,cje,capsule,'glca6etn+hep+omepn+3ome+6ome-galfnac+omepn-ribf','GalfNAc').
contains_part(brendan,cje,capsule,'glca-galfnac','GalfNAc').
contains_part(brendan,cje,capsule,'glca+hep-galfnac','GalfNAc').
contains_part(brendan,cje,capsule,'glca+hep+omepn-galfnac','GalfNAc').
contains_part(brendan,cje,capsule,'glca+hep+3ome-galfnac','GalfNAc').
contains_part(brendan,cje,capsule,'glca+hep+6ome-galfnac','GalfNAc').
contains_part(brendan,cje,capsule,'glca+hep+omepn+3ome-galfnac','GalfNAc').
contains_part(brendan,cje,capsule,'glca+hep+omepn+6ome-galfnac','GalfNAc').
contains_part(brendan,cje,capsule,'glca+hep+3ome+6ome-galfnac','GalfNAc').
contains_part(brendan,cje,capsule,'glca+hep+omepn+3ome+6ome-galfnac','GalfNAc').
contains_part(brendan,cje,capsule,'glca-galfnac+omepn','GalfNAc').
contains_part(brendan,cje,capsule,'glca+hep-galfnac+omepn','GalfNAc').

contains_part(brendan,cje,capsule,'glca+hep+omepn-galfnac+omepn','GalfNAc').
contains_part(brendan,cje,capsule,'glca+hep+3ome-galfnac+omepn','GalfNAc').
contains_part(brendan,cje,capsule,'glca+hep+6ome-galfnac+omepn','GalfNAc').
contains_part(brendan,cje,capsule,'glca+hep+omepn+3ome-galfnac+omepn','GalfNAc').
contains_part(brendan,cje,capsule,'glca+hep+omepn+6ome-galfnac+omepn','GalfNAc').
contains_part(brendan,cje,capsule,'glca+hep+3ome+6ome-galfnac+omepn','GalfNAc').
contains_part(brendan,cje,capsule,'glca+hep+omepn+3ome+6ome-galfnac+omepn','GalfNAc').
contains_part(brendan,cje,capsule,'glca-galfnac-ribf','GalfNAc').
contains_part(brendan,cje,capsule,'glca+hep-galfnac-ribf','GalfNAc').
contains_part(brendan,cje,capsule,'glca+hep+omepn-galfnac-ribf','GalfNAc').
contains_part(brendan,cje,capsule,'glca+hep+3ome-galfnac-ribf','GalfNAc').
contains_part(brendan,cje,capsule,'glca+hep+6ome-galfnac-ribf','GalfNAc').
contains_part(brendan,cje,capsule,'glca+hep+omepn+3ome-galfnac-ribf','GalfNAc').
contains_part(brendan,cje,capsule,'glca+hep+omepn+6ome-galfnac-ribf','GalfNAc').
contains_part(brendan,cje,capsule,'glca+hep+3ome+6ome-galfnac-ribf','GalfNAc').
contains_part(brendan,cje,capsule,'glca+hep+omepn+3ome+6ome-galfnac-ribf','GalfNAc').
contains_part(brendan,cje,capsule,'glca-galfnac+omepn-ribf','GalfNAc').
contains_part(brendan,cje,capsule,'glca+hep-galfnac+omepn-ribf','GalfNAc').
contains_part(brendan,cje,capsule,'glca+hep+omepn-galfnac+omepn-ribf','GalfNAc').
contains_part(brendan,cje,capsule,'glca+hep+3ome-galfnac+omepn-ribf','GalfNAc').
contains_part(brendan,cje,capsule,'glca+hep+6ome-galfnac+omepn-ribf','GalfNAc').
contains_part(brendan,cje,capsule,'glca+hep+omepn+3ome-galfnac+omepn-ribf','GalfNAc').
contains_part(brendan,cje,capsule,'glca+hep+omepn+6ome-galfnac+omepn-ribf','GalfNAc').
contains_part(brendan,cje,capsule,'glca+hep+3ome+6ome-galfnac+omepn-ribf','GalfNAc').
contains_part(brendan,cje,capsule,'glca+hep+omepn+3ome+6ome-galfnac+omepn-ribf','GalfNAc').

%% GalfNAc OMePN

contains_part(brendan,cje,capsule,'glca6ngro-galfnac+omepn','GalfNAc OMePN').
contains_part(brendan,cje,capsule,'glca6ngro+hep-galfnac+omepn','GalfNAc OMePN').
contains_part(brendan,cje,capsule,'glca6ngro+hep+omepn-galfnac+omepn','GalfNAc OMePN').
contains_part(brendan,cje,capsule,'glca6ngro+hep+3ome-galfnac+omepn','GalfNAc OMePN').
contains_part(brendan,cje,capsule,'glca6ngro+hep+6ome-galfnac+omepn','GalfNAc OMePN').
contains_part(brendan,cje,capsule,'glca6ngro+hep+omepn+3ome-galfnac+omepn','GalfNAc OMePN').
contains_part(brendan,cje,capsule,'glca6ngro+hep+omepn+6ome-galfnac+omepn','GalfNAc OMePN').
contains_part(brendan,cje,capsule,'glca6ngro+hep+3ome+6ome-galfnac+omepn','GalfNAc OMePN').
contains_part(brendan,cje,capsule,'glca6ngro+hep+omepn+3ome+6ome-galfnac+omepn','GalfNAc OMePN').
contains_part(brendan,cje,capsule,'glca6ngro-galfnac+omepn-ribf','GalfNAc OMePN').
contains_part(brendan,cje,capsule,'glca6ngro+hep-galfnac+omepn-ribf','GalfNAc OMePN').
contains_part(brendan,cje,capsule,'glca6ngro+hep+omepn-galfnac+omepn-ribf','GalfNAc OMePN').
contains_part(brendan,cje,capsule,'glca6ngro+hep+3ome-galfnac+omepn-ribf','GalfNAc OMePN').
contains_part(brendan,cje,capsule,'glca6ngro+hep+6ome-galfnac+omepn-ribf','GalfNAc OMePN').
contains_part(brendan,cje,capsule,'glca6ngro+hep+omepn+3ome-galfnac+omepn-ribf','GalfNAc OMePN').
contains_part(brendan,cje,capsule,'glca6ngro+hep+omepn+6ome-galfnac+omepn-ribf','GalfNAc OMePN').
contains_part(brendan,cje,capsule,'glca6ngro+hep+3ome+6ome-galfnac+omepn-ribf','GalfNAc OMePN').
contains_part(brendan,cje,capsule,'glca6ngro+hep+omepn+3ome+6ome-galfnac+omepn-ribf','GalfNAc OMePN').
contains_part(brendan,cje,capsule,'glca6etn-galfnac+omepn','GalfNAc OMePN').
contains_part(brendan,cje,capsule,'glca6etn+hep-galfnac+omepn','GalfNAc OMePN').
contains_part(brendan,cje,capsule,'glca6etn+hep+omepn-galfnac+omepn','GalfNAc OMePN').
contains_part(brendan,cje,capsule,'glca6etn+hep+3ome-galfnac+omepn','GalfNAc OMePN').
contains_part(brendan,cje,capsule,'glca6etn+hep+6ome-galfnac+omepn','GalfNAc OMePN').
contains_part(brendan,cje,capsule,'glca6etn+hep+omepn+3ome-galfnac+omepn','GalfNAc OMePN').
contains_part(brendan,cje,capsule,'glca6etn+hep+omepn+6ome-galfnac+omepn','GalfNAc OMePN').
contains_part(brendan,cje,capsule,'glca6etn+hep+3ome+6ome-galfnac+omepn','GalfNAc OMePN').

contains_part(brendan,cje,capsule,'glca6etn+hep+omepn+3ome+6ome-galfnac+omepn','GalfNAc OMePN').
contains_part(brendan,cje,capsule,'glca6etn-galfnac+omepn-ribf','GalfNAc OMePN').
contains_part(brendan,cje,capsule,'glca6etn+hep-galfnac+omepn-ribf','GalfNAc OMePN').
contains_part(brendan,cje,capsule,'glca6etn+hep+omepn-galfnac+omepn-ribf','GalfNAc OMePN').
contains_part(brendan,cje,capsule,'glca6etn+hep+3ome-galfnac+omepn-ribf','GalfNAc OMePN').
contains_part(brendan,cje,capsule,'glca6etn+hep+6ome-galfnac+omepn-ribf','GalfNAc OMePN').
contains_part(brendan,cje,capsule,'glca6etn+hep+omepn+3ome-galfnac+omepn-ribf','GalfNAc OMePN').
contains_part(brendan,cje,capsule,'glca6etn+hep+omepn+6ome-galfnac+omepn-ribf','GalfNAc OMePN').
contains_part(brendan,cje,capsule,'glca6etn+hep+3ome+6ome-galfnac+omepn-ribf','GalfNAc OMePN').
contains_part(brendan,cje,capsule,'glca6etn+hep+omepn+3ome+6ome-galfnac+omepn-ribf','GalfNAc OMePN').
contains_part(brendan,cje,capsule,'glca-galfnac+omepn','GalfNAc OMePN').
contains_part(brendan,cje,capsule,'glca+hep-galfnac+omepn','GalfNAc OMePN').
contains_part(brendan,cje,capsule,'glca+hep+omepn-galfnac+omepn','GalfNAc OMePN').
contains_part(brendan,cje,capsule,'glca+hep+3ome-galfnac+omepn','GalfNAc OMePN').
contains_part(brendan,cje,capsule,'glca+hep+6ome-galfnac+omepn','GalfNAc OMePN').
contains_part(brendan,cje,capsule,'glca+hep+omepn+3ome-galfnac+omepn','GalfNAc OMePN').
contains_part(brendan,cje,capsule,'glca+hep+omepn+6ome-galfnac+omepn','GalfNAc OMePN').
contains_part(brendan,cje,capsule,'glca+hep+3ome+6ome-galfnac+omepn','GalfNAc OMePN').
contains_part(brendan,cje,capsule,'glca+hep+omepn+3ome+6ome-galfnac+omepn','GalfNAc OMePN').
contains_part(brendan,cje,capsule,'glca-galfnac+omepn-ribf','GalfNAc OMePN').
contains_part(brendan,cje,capsule,'glca+hep-galfnac+omepn-ribf','GalfNAc OMePN').
contains_part(brendan,cje,capsule,'glca+hep+omepn-galfnac+omepn-ribf','GalfNAc OMePN').
contains_part(brendan,cje,capsule,'glca+hep+3ome-galfnac+omepn-ribf','GalfNAc OMePN').
contains_part(brendan,cje,capsule,'glca+hep+6ome-galfnac+omepn-ribf','GalfNAc OMePN').
contains_part(brendan,cje,capsule,'glca+hep+omepn+3ome-galfnac+omepn-ribf','GalfNAc OMePN').
contains_part(brendan,cje,capsule,'glca+hep+omepn+6ome-galfnac+omepn-ribf','GalfNAc OMePN').
contains_part(brendan,cje,capsule,'glca+hep+3ome+6ome-galfnac+omepn-ribf','GalfNAc OMePN').
contains_part(brendan,cje,capsule,'glca+hep+omepn+3ome+6ome-galfnac+omepn-ribf','GalfNAc OMePN').

%% Ribf

contains_part(brendan,cje,capsule,'glca6ngro-galfnac-ribf','Ribf').
contains_part(brendan,cje,capsule,'glca6ngro+hep-galfnac-ribf','Ribf').
contains_part(brendan,cje,capsule,'glca6ngro+hep+omepn-galfnac-ribf','Ribf').
contains_part(brendan,cje,capsule,'glca6ngro+hep+3ome-galfnac-ribf','Ribf').
contains_part(brendan,cje,capsule,'glca6ngro+hep+6ome-galfnac-ribf','Ribf').
contains_part(brendan,cje,capsule,'glca6ngro+hep+omepn+3ome-galfnac-ribf','Ribf').
contains_part(brendan,cje,capsule,'glca6ngro+hep+omepn+6ome-galfnac-ribf','Ribf').
contains_part(brendan,cje,capsule,'glca6ngro+hep+3ome+6ome-galfnac-ribf','Ribf').
contains_part(brendan,cje,capsule,'glca6ngro+hep+omepn+3ome+6ome-galfnac-ribf','Ribf').
contains_part(brendan,cje,capsule,'glca6ngro-galfnac+omepn-ribf','Ribf').
contains_part(brendan,cje,capsule,'glca6ngro+hep-galfnac+omepn-ribf','Ribf').
contains_part(brendan,cje,capsule,'glca6ngro+hep+omepn-galfnac+omepn-ribf','Ribf').
contains_part(brendan,cje,capsule,'glca6ngro+hep+3ome-galfnac+omepn-ribf','Ribf').
contains_part(brendan,cje,capsule,'glca6ngro+hep+6ome-galfnac+omepn-ribf','Ribf').
contains_part(brendan,cje,capsule,'glca6ngro+hep+omepn+3ome-galfnac+omepn-ribf','Ribf').
contains_part(brendan,cje,capsule,'glca6ngro+hep+omepn+6ome-galfnac+omepn-ribf','Ribf').
contains_part(brendan,cje,capsule,'glca6ngro+hep+3ome+6ome-galfnac+omepn-ribf','Ribf').
contains_part(brendan,cje,capsule,'glca6ngro+hep+omepn+3ome+6ome-galfnac+omepn-ribf','Ribf').
contains_part(brendan,cje,capsule,'glca6etn-galfnac-ribf','Ribf').
contains_part(brendan,cje,capsule,'glca6etn+hep-galfnac-ribf','Ribf').
contains_part(brendan,cje,capsule,'glca6etn+hep+omepn-galfnac-ribf','Ribf').
contains_part(brendan,cje,capsule,'glca6etn+hep+3ome-galfnac-ribf','Ribf').
contains_part(brendan,cje,capsule,'glca6etn+hep+6ome-galfnac-ribf','Ribf').

```
contains_part(brendan,cje,capsule,'glca6etn+hep+omepn+3ome-galfnac-ribf','Ribf').
contains_part(brendan,cje,capsule,'glca6etn+hep+omepn+6ome-galfnac-ribf','Ribf').
contains_part(brendan,cje,capsule,'glca6etn+hep+3ome+6ome-galfnac-ribf','Ribf').
contains_part(brendan,cje,capsule,'glca6etn+hep+omepn+3ome+6ome-galfnac-ribf','Ribf').
contains_part(brendan,cje,capsule,'glca6etn-galfnac+omepn-ribf','Ribf').
contains_part(brendan,cje,capsule,'glca6etn+hep-galfnac+omepn-ribf','Ribf').
contains_part(brendan,cje,capsule,'glca6etn+hep+omepn-galfnac+omepn-ribf','Ribf').
contains_part(brendan,cje,capsule,'glca6etn+hep+3ome-galfnac+omepn-ribf','Ribf').
contains_part(brendan,cje,capsule,'glca6etn+hep+6ome-galfnac+omepn-ribf','Ribf').
contains_part(brendan,cje,capsule,'glca6etn+hep+omepn+3ome-galfnac+omepn-ribf','Ribf').
contains_part(brendan,cje,capsule,'glca6etn+hep+omepn+6ome-galfnac+omepn-ribf','Ribf').
contains_part(brendan,cje,capsule,'glca6etn+hep+3ome+6ome-galfnac+omepn-ribf','Ribf').
contains_part(brendan,cje,capsule,'glca6etn+hep+omepn+3ome+6ome-galfnac+omepn-ribf','Ribf').
contains_part(brendan,cje,capsule,'glca-galfnac-ribf','Ribf').
contains_part(brendan,cje,capsule,'glca+hep-galfnac-ribf','Ribf').
contains_part(brendan,cje,capsule,'glca+hep+omepn-galfnac-ribf','Ribf').
contains_part(brendan,cje,capsule,'glca+hep+3ome-galfnac-ribf','Ribf').
contains_part(brendan,cje,capsule,'glca+hep+6ome-galfnac-ribf','Ribf').
contains_part(brendan,cje,capsule,'glca+hep+omepn+3ome-galfnac-ribf','Ribf').
contains_part(brendan,cje,capsule,'glca+hep+omepn+6ome-galfnac-ribf','Ribf').
contains_part(brendan,cje,capsule,'glca+hep+3ome+6ome-galfnac-ribf','Ribf').
contains_part(brendan,cje,capsule,'glca+hep+omepn+3ome+6ome-galfnac-ribf','Ribf').
contains_part(brendan,cje,capsule,'glca-galfnac+omepn-ribf','Ribf').
contains_part(brendan,cje,capsule,'glca+hep-galfnac+omepn-ribf','Ribf').
contains_part(brendan,cje,capsule,'glca+hep+omepn-galfnac+omepn-ribf','Ribf').
contains_part(brendan,cje,capsule,'glca+hep+3ome-galfnac+omepn-ribf','Ribf').
contains_part(brendan,cje,capsule,'glca+hep+6ome-galfnac+omepn-ribf','Ribf').
contains_part(brendan,cje,capsule,'glca+hep+omepn+3ome-galfnac+omepn-ribf','Ribf').
contains_part(brendan,cje,capsule,'glca+hep+omepn+6ome-galfnac+omepn-ribf','Ribf').
contains_part(brendan,cje,capsule,'glca+hep+3ome+6ome-galfnac+omepn-ribf','Ribf').
contains_part(brendan,cje,capsule,'glca+hep+omepn+3ome+6ome-galfnac+omepn-ribf','Ribf').


%% Adds part

%% GlcA

adds_part(brendan,cje,capsule,capsule_0,'GlcA').

%% NGro

adds_part(brendan,cje,capsule,capsule_ngro0,'NGro').
adds_part(brendan,cje,capsule,capsule_addngro1,'NGro').
adds_part(brendan,cje,capsule,capsule_addngro2,'NGro').
adds_part(brendan,cje,capsule,capsule_addngro3,'NGro').
adds_part(brendan,cje,capsule,capsule_addngro4,'NGro').
adds_part(brendan,cje,capsule,capsule_addngro5,'NGro').
adds_part(brendan,cje,capsule,capsule_addngro6,'NGro').
adds_part(brendan,cje,capsule,capsule_addngro7,'NGro').
adds_part(brendan,cje,capsule,capsule_addngro8,'NGro').
adds_part(brendan,cje,capsule,capsule_addngro9,'NGro').
adds_part(brendan,cje,capsule,capsule_addngro10,'NGro').
adds_part(brendan,cje,capsule,capsule_addngro11,'NGro').
adds_part(brendan,cje,capsule,capsule_addngro12,'NGro').
```

```
adds_part(brendan,cje,capsule,capsule_addngro13,'NGro').
adds_part(brendan,cje,capsule,capsule_addngro14,'NGro').
adds_part(brendan,cje,capsule,capsule_addngro15,'NGro').
adds_part(brendan,cje,capsule,capsule_addngro16,'NGro').
adds_part(brendan,cje,capsule,capsule_addngro17,'NGro').
adds_part(brendan,cje,capsule,capsule_addngro18,'NGro').
adds_part(brendan,cje,capsule,capsule_addngro19,'NGro').
adds_part(brendan,cje,capsule,capsule_addngro20,'NGro').
adds_part(brendan,cje,capsule,capsule_addngro21,'NGro').
adds_part(brendan,cje,capsule,capsule_addngro22,'NGro').
adds_part(brendan,cje,capsule,capsule_addngro23,'NGro').
adds_part(brendan,cje,capsule,capsule_addngro24,'NGro').
adds_part(brendan,cje,capsule,capsule_addngro25,'NGro').
adds_part(brendan,cje,capsule,capsule_addngro26,'NGro').
adds_part(brendan,cje,capsule,capsule_addngro27,'NGro').
adds_part(brendan,cje,capsule,capsule_addngro28,'NGro').
adds_part(brendan,cje,capsule,capsule_addngro29,'NGro').
adds_part(brendan,cje,capsule,capsule_addngro30,'NGro').
adds_part(brendan,cje,capsule,capsule_addngro31,'NGro').
adds_part(brendan,cje,capsule,capsule_addngro32,'NGro').
adds_part(brendan,cje,capsule,capsule_addngro33,'NGro').
adds_part(brendan,cje,capsule,capsule_addngro34,'NGro').
adds_part(brendan,cje,capsule,capsule_addngro35,'NGro').
adds_part(brendan,cje,capsule,capsule_addngro36,'NGro').
adds_part(brendan,cje,capsule,capsule_addngro37,'NGro').
adds_part(brendan,cje,capsule,capsule_addngro38,'NGro').
adds_part(brendan,cje,capsule,capsule_addngro39,'NGro').
adds_part(brendan,cje,capsule,capsule_addngro40,'NGro').
adds_part(brendan,cje,capsule,capsule_addngro41,'NGro').
adds_part(brendan,cje,capsule,capsule_addngro42,'NGro').
adds_part(brendan,cje,capsule,capsule_addngro43,'NGro').
adds_part(brendan,cje,capsule,capsule_addngro44,'NGro').

%% EtN

adds_part(brendan,cje,capsule,capsule_etn0,'EtN').
adds_part(brendan,cje,capsule,capsule_addetn1,'EtN').
adds_part(brendan,cje,capsule,capsule_addetn2,'EtN').
adds_part(brendan,cje,capsule,capsule_addetn3,'EtN').
adds_part(brendan,cje,capsule,capsule_addetn4,'EtN').
adds_part(brendan,cje,capsule,capsule_addetn5,'EtN').
adds_part(brendan,cje,capsule,capsule_addetn6,'EtN').
adds_part(brendan,cje,capsule,capsule_addetn7,'EtN').
adds_part(brendan,cje,capsule,capsule_addetn8,'EtN').
adds_part(brendan,cje,capsule,capsule_addetn9,'EtN').
adds_part(brendan,cje,capsule,capsule_addetn10,'EtN').
adds_part(brendan,cje,capsule,capsule_addetn11,'EtN').
adds_part(brendan,cje,capsule,capsule_addetn12,'EtN').
adds_part(brendan,cje,capsule,capsule_addetn13,'EtN').
adds_part(brendan,cje,capsule,capsule_addetn14,'EtN').
adds_part(brendan,cje,capsule,capsule_addetn15,'EtN').
adds_part(brendan,cje,capsule,capsule_addetn16,'EtN').
adds_part(brendan,cje,capsule,capsule_addetn17,'EtN').
adds_part(brendan,cje,capsule,capsule_addetn18,'EtN').
```

```
adds_part(brendan,cje,capsule,capsule_addetn19,'EtN').
adds_part(brendan,cje,capsule,capsule_addetn20,'EtN').
adds_part(brendan,cje,capsule,capsule_addetn21,'EtN').
adds_part(brendan,cje,capsule,capsule_addetn22,'EtN').
adds_part(brendan,cje,capsule,capsule_addetn23,'EtN').
adds_part(brendan,cje,capsule,capsule_addetn24,'EtN').
adds_part(brendan,cje,capsule,capsule_addetn25,'EtN').
adds_part(brendan,cje,capsule,capsule_addetn26,'EtN').
adds_part(brendan,cje,capsule,capsule_addetn27,'EtN').
adds_part(brendan,cje,capsule,capsule_addetn28,'EtN').
adds_part(brendan,cje,capsule,capsule_addetn29,'EtN').
adds_part(brendan,cje,capsule,capsule_addetn30,'EtN').
adds_part(brendan,cje,capsule,capsule_addetn31,'EtN').
adds_part(brendan,cje,capsule,capsule_addetn32,'EtN').
adds_part(brendan,cje,capsule,capsule_addetn33,'EtN').
adds_part(brendan,cje,capsule,capsule_addetn34,'EtN').
adds_part(brendan,cje,capsule,capsule_addetn35,'EtN').
adds_part(brendan,cje,capsule,capsule_addetn36,'EtN').
adds_part(brendan,cje,capsule,capsule_addetn37,'EtN').
adds_part(brendan,cje,capsule,capsule_addetn38,'EtN').
adds_part(brendan,cje,capsule,capsule_addetn39,'EtN').
adds_part(brendan,cje,capsule,capsule_addetn40,'EtN').
adds_part(brendan,cje,capsule,capsule_addetn41,'EtN').
adds_part(brendan,cje,capsule,capsule_addetn42,'EtN').
adds_part(brendan,cje,capsule,capsule_addetn43,'EtN').
adds_part(brendan,cje,capsule,capsule_addetn44,'EtN').

%% Hep

adds_part(brendan,cje,capsule,capsule_ngro1,'Hep').
adds_part(brendan,cje,capsule,capsule_ngro1a,'Hep').
adds_part(brendan,cje,capsule,capsule_ngro1b,'Hep').
adds_part(brendan,cje,capsule,capsule_ngro1c,'Hep').
adds_part(brendan,cje,capsule,capsule_etn1,'Hep').
adds_part(brendan,cje,capsule,capsule_etn1a,'Hep').
adds_part(brendan,cje,capsule,capsule_etn1b,'Hep').
adds_part(brendan,cje,capsule,capsule_etn1c,'Hep').
adds_part(brendan,cje,capsule,capsule_1,'Hep').
adds_part(brendan,cje,capsule,capsule_1a,'Hep').
adds_part(brendan,cje,capsule,capsule_1b,'Hep').
adds_part(brendan,cje,capsule,capsule_1c,'Hep').

%% Hep OMePN

adds_part(brendan,cje,capsule,capsule_ngro4,'Hep OMePN').
adds_part(brendan,cje,capsule,capsule_ngro5,'Hep OMePN').
adds_part(brendan,cje,capsule,capsule_ngro5a,'Hep OMePN').
adds_part(brendan,cje,capsule,capsule_ngro5b,'Hep OMePN').
adds_part(brendan,cje,capsule,capsule_ngro8,'Hep OMePN').
adds_part(brendan,cje,capsule,capsule_ngro9,'Hep OMePN').
adds_part(brendan,cje,capsule,capsule_ngro9a,'Hep OMePN').
adds_part(brendan,cje,capsule,capsule_ngro9b,'Hep OMePN').
adds_part(brendan,cje,capsule,capsule_ngro12,'Hep OMePN').
adds_part(brendan,cje,capsule,capsule_ngro13,'Hep OMePN').
```

```
adds_part(brendan,cje,capsule,capsule_ngro13a,'Hep OMePN').
adds_part(brendan,cje,capsule,capsule_ngro13b,'Hep OMePN').
adds_part(brendan,cje,capsule,capsule_ngro16,'Hep OMePN').
adds_part(brendan,cje,capsule,capsule_ngro17,'Hep OMePN').
adds_part(brendan,cje,capsule,capsule_ngro17a,'Hep OMePN').
adds_part(brendan,cje,capsule,capsule_ngro17b,'Hep OMePN').
adds_part(brendan,cje,capsule,capsule_ngro20,'Hep OMePN').
adds_part(brendan,cje,capsule,capsule_ngro21,'Hep OMePN').
adds_part(brendan,cje,capsule,capsule_ngro21a,'Hep OMePN').
adds_part(brendan,cje,capsule,capsule_ngro21b,'Hep OMePN').
adds_part(brendan,cje,capsule,capsule_etn4,'Hep OMePN').
adds_part(brendan,cje,capsule,capsule_etn5,'Hep OMePN').
adds_part(brendan,cje,capsule,capsule_etn5a,'Hep OMePN').
adds_part(brendan,cje,capsule,capsule_etn5b,'Hep OMePN').
adds_part(brendan,cje,capsule,capsule_etn8,'Hep OMePN').
adds_part(brendan,cje,capsule,capsule_etn9,'Hep OMePN').
adds_part(brendan,cje,capsule,capsule_etn9a,'Hep OMePN').
adds_part(brendan,cje,capsule,capsule_etn9b,'Hep OMePN').
adds_part(brendan,cje,capsule,capsule_etn12,'Hep OMePN').
adds_part(brendan,cje,capsule,capsule_etn13,'Hep OMePN').
adds_part(brendan,cje,capsule,capsule_etn13a,'Hep OMePN').
adds_part(brendan,cje,capsule,capsule_etn13b,'Hep OMePN').
adds_part(brendan,cje,capsule,capsule_etn16,'Hep OMePN').
adds_part(brendan,cje,capsule,capsule_etn17,'Hep OMePN').
adds_part(brendan,cje,capsule,capsule_etn17a,'Hep OMePN').
adds_part(brendan,cje,capsule,capsule_etn17b,'Hep OMePN').
adds_part(brendan,cje,capsule,capsule_etn20,'Hep OMePN').
adds_part(brendan,cje,capsule,capsule_etn21,'Hep OMePN').
adds_part(brendan,cje,capsule,capsule_etn21a,'Hep OMePN').
adds_part(brendan,cje,capsule,capsule_etn21b,'Hep OMePN').
adds_part(brendan,cje,capsule,capsule_4,'Hep OMePN').
adds_part(brendan,cje,capsule,capsule_5,'Hep OMePN').
adds_part(brendan,cje,capsule,capsule_5a,'Hep OMePN').
adds_part(brendan,cje,capsule,capsule_5b,'Hep OMePN').
adds_part(brendan,cje,capsule,capsule_8,'Hep OMePN').
adds_part(brendan,cje,capsule,capsule_9,'Hep OMePN').
adds_part(brendan,cje,capsule,capsule_9a,'Hep OMePN').
adds_part(brendan,cje,capsule,capsule_9b,'Hep OMePN').
adds_part(brendan,cje,capsule,capsule_12,'Hep OMePN').
adds_part(brendan,cje,capsule,capsule_13,'Hep OMePN').
adds_part(brendan,cje,capsule,capsule_13a,'Hep OMePN').
adds_part(brendan,cje,capsule,capsule_13b,'Hep OMePN').
adds_part(brendan,cje,capsule,capsule_16,'Hep OMePN').
adds_part(brendan,cje,capsule,capsule_17,'Hep OMePN').
adds_part(brendan,cje,capsule,capsule_17a,'Hep OMePN').
adds_part(brendan,cje,capsule,capsule_17b,'Hep OMePN').
adds_part(brendan,cje,capsule,capsule_20,'Hep OMePN').
adds_part(brendan,cje,capsule,capsule_21,'Hep OMePN').
adds_part(brendan,cje,capsule,capsule_21a,'Hep OMePN').
adds_part(brendan,cje,capsule,capsule_21b,'Hep OMePN').

%% Hep 3-OMe

adds_part(brendan,cje,capsule,capsule_ngro2,'Hep 3-OMe').
```

```
adds_part(brendan,cje,capsule,capsule_ngro3,'Hep 3-OMe').
adds_part(brendan,cje,capsule,capsule_ngro3a,'Hep 3-OMe').
adds_part(brendan,cje,capsule,capsule_ngro3b,'Hep 3-OMe').
adds_part(brendan,cje,capsule,capsule_ngro6,'Hep 3-OMe').
adds_part(brendan,cje,capsule,capsule_ngro7,'Hep 3-OMe').
adds_part(brendan,cje,capsule,capsule_ngro7a,'Hep 3-OMe').
adds_part(brendan,cje,capsule,capsule_ngro7b,'Hep 3-OMe').
adds_part(brendan,cje,capsule,capsule_ngro10,'Hep 3-OMe').
adds_part(brendan,cje,capsule,capsule_ngro11,'Hep 3-OMe').
adds_part(brendan,cje,capsule,capsule_ngro11a,'Hep 3-OMe').
adds_part(brendan,cje,capsule,capsule_ngro11b,'Hep 3-OMe').
adds_part(brendan,cje,capsule,capsule_ngro14,'Hep 3-OMe').
adds_part(brendan,cje,capsule,capsule_ngro15,'Hep 3-OMe').
adds_part(brendan,cje,capsule,capsule_ngro15a,'Hep 3-OMe').
adds_part(brendan,cje,capsule,capsule_ngro15b,'Hep 3-OMe').
adds_part(brendan,cje,capsule,capsule_ngro18,'Hep 3-OMe').
adds_part(brendan,cje,capsule,capsule_ngro19,'Hep 3-OMe').
adds_part(brendan,cje,capsule,capsule_ngro19a,'Hep 3-OMe').
adds_part(brendan,cje,capsule,capsule_ngro19b,'Hep 3-OMe').
adds_part(brendan,cje,capsule,capsule_etn2,'Hep 3-OMe').
adds_part(brendan,cje,capsule,capsule_etn3,'Hep 3-OMe').
adds_part(brendan,cje,capsule,capsule_etn3a,'Hep 3-OMe').
adds_part(brendan,cje,capsule,capsule_etn3b,'Hep 3-OMe').
adds_part(brendan,cje,capsule,capsule_etn6,'Hep 3-OMe').
adds_part(brendan,cje,capsule,capsule_etn7,'Hep 3-OMe').
adds_part(brendan,cje,capsule,capsule_etn7a,'Hep 3-OMe').
adds_part(brendan,cje,capsule,capsule_etn7b,'Hep 3-OMe').
adds_part(brendan,cje,capsule,capsule_etn10,'Hep 3-OMe').
adds_part(brendan,cje,capsule,capsule_etn11,'Hep 3-OMe').
adds_part(brendan,cje,capsule,capsule_etn11a,'Hep 3-OMe').
adds_part(brendan,cje,capsule,capsule_etn11b,'Hep 3-OMe').
adds_part(brendan,cje,capsule,capsule_etn14,'Hep 3-OMe').
adds_part(brendan,cje,capsule,capsule_etn15,'Hep 3-OMe').
adds_part(brendan,cje,capsule,capsule_etn15a,'Hep 3-OMe').
adds_part(brendan,cje,capsule,capsule_etn15b,'Hep 3-OMe').
adds_part(brendan,cje,capsule,capsule_etn18,'Hep 3-OMe').
adds_part(brendan,cje,capsule,capsule_etn19,'Hep 3-OMe').
adds_part(brendan,cje,capsule,capsule_etn19a,'Hep 3-OMe').
adds_part(brendan,cje,capsule,capsule_etn19b,'Hep 3-OMe').
adds_part(brendan,cje,capsule,capsule_2,'Hep 3-OMe').
adds_part(brendan,cje,capsule,capsule_3,'Hep 3-OMe').
adds_part(brendan,cje,capsule,capsule_3a,'Hep 3-OMe').
adds_part(brendan,cje,capsule,capsule_3b,'Hep 3-OMe').
adds_part(brendan,cje,capsule,capsule_6,'Hep 3-OMe').
adds_part(brendan,cje,capsule,capsule_7,'Hep 3-OMe').
adds_part(brendan,cje,capsule,capsule_7a,'Hep 3-OMe').
adds_part(brendan,cje,capsule,capsule_7b,'Hep 3-OMe').
adds_part(brendan,cje,capsule,capsule_10,'Hep 3-OMe').
adds_part(brendan,cje,capsule,capsule_11,'Hep 3-OMe').
adds_part(brendan,cje,capsule,capsule_11a,'Hep 3-OMe').
adds_part(brendan,cje,capsule,capsule_11b,'Hep 3-OMe').
adds_part(brendan,cje,capsule,capsule_14,'Hep 3-OMe').
adds_part(brendan,cje,capsule,capsule_15,'Hep 3-OMe').
adds_part(brendan,cje,capsule,capsule_15a,'Hep 3-OMe').
```

```
adds_part(brendan,cje,capsule,capsule_15b,'Hep 3-OMe').
adds_part(brendan,cje,capsule,capsule_18,'Hep 3-OMe').
adds_part(brendan,cje,capsule,capsule_19,'Hep 3-OMe').
adds_part(brendan,cje,capsule,capsule_19a,'Hep 3-OMe').
adds_part(brendan,cje,capsule,capsule_19b,'Hep 3-OMe').

%% Hep 6-Ome

adds_part(brendan,cje,capsule,capsule_ngro5c,'Hep 6-OMe').
adds_part(brendan,cje,capsule,capsule_ngro5d,'Hep 6-OMe').
adds_part(brendan,cje,capsule,capsule_ngro5e,'Hep 6-OMe').
adds_part(brendan,cje,capsule,capsule_ngro5f,'Hep 6-OMe').
adds_part(brendan,cje,capsule,capsule_ngro9c,'Hep 6-OMe').
adds_part(brendan,cje,capsule,capsule_ngro9d,'Hep 6-OMe').
adds_part(brendan,cje,capsule,capsule_ngro9e,'Hep 6-OMe').
adds_part(brendan,cje,capsule,capsule_ngro9f,'Hep 6-OMe').
adds_part(brendan,cje,capsule,capsule_ngro13c,'Hep 6-OMe').
adds_part(brendan,cje,capsule,capsule_ngro13d,'Hep 6-OMe').
adds_part(brendan,cje,capsule,capsule_ngro13e,'Hep 6-OMe').
adds_part(brendan,cje,capsule,capsule_ngro13f,'Hep 6-OMe').
adds_part(brendan,cje,capsule,capsule_ngro17c,'Hep 6-OMe').
adds_part(brendan,cje,capsule,capsule_ngro17d,'Hep 6-OMe').
adds_part(brendan,cje,capsule,capsule_ngro17e,'Hep 6-OMe').
adds_part(brendan,cje,capsule,capsule_ngro17f,'Hep 6-OMe').
adds_part(brendan,cje,capsule,capsule_ngro21c,'Hep 6-OMe').
adds_part(brendan,cje,capsule,capsule_ngro21d,'Hep 6-OMe').
adds_part(brendan,cje,capsule,capsule_ngro21e,'Hep 6-OMe').
adds_part(brendan,cje,capsule,capsule_ngro21f,'Hep 6-OMe').
adds_part(brendan,cje,capsule,capsule_etn5c,'Hep 6-OMe').
adds_part(brendan,cje,capsule,capsule_etn5d,'Hep 6-OMe').
adds_part(brendan,cje,capsule,capsule_etn5e,'Hep 6-OMe').
adds_part(brendan,cje,capsule,capsule_etn5f,'Hep 6-OMe').
adds_part(brendan,cje,capsule,capsule_etn9c,'Hep 6-OMe').
adds_part(brendan,cje,capsule,capsule_etn9d,'Hep 6-OMe').
adds_part(brendan,cje,capsule,capsule_etn9e,'Hep 6-OMe').
adds_part(brendan,cje,capsule,capsule_etn9f,'Hep 6-OMe').
adds_part(brendan,cje,capsule,capsule_etn13c,'Hep 6-OMe').
adds_part(brendan,cje,capsule,capsule_etn13d,'Hep 6-OMe').
adds_part(brendan,cje,capsule,capsule_etn13e,'Hep 6-OMe').
adds_part(brendan,cje,capsule,capsule_etn13f,'Hep 6-OMe').
adds_part(brendan,cje,capsule,capsule_etn17c,'Hep 6-OMe').
adds_part(brendan,cje,capsule,capsule_etn17d,'Hep 6-OMe').
adds_part(brendan,cje,capsule,capsule_etn17e,'Hep 6-OMe').
adds_part(brendan,cje,capsule,capsule_etn17f,'Hep 6-OMe').
adds_part(brendan,cje,capsule,capsule_etn21c,'Hep 6-OMe').
adds_part(brendan,cje,capsule,capsule_etn21d,'Hep 6-OMe').
adds_part(brendan,cje,capsule,capsule_etn21e,'Hep 6-OMe').
adds_part(brendan,cje,capsule,capsule_etn21f,'Hep 6-OMe').
adds_part(brendan,cje,capsule,capsule_5c,'Hep 6-OMe').
adds_part(brendan,cje,capsule,capsule_5d,'Hep 6-OMe').
adds_part(brendan,cje,capsule,capsule_5e,'Hep 6-OMe').
adds_part(brendan,cje,capsule,capsule_5f,'Hep 6-OMe').
adds_part(brendan,cje,capsule,capsule_9c,'Hep 6-OMe').
adds_part(brendan,cje,capsule,capsule_9d,'Hep 6-OMe').
```

```
adds_part(brendan,cje,capsule,capsule_9e,'Hep 6-OMe').
adds_part(brendan,cje,capsule,capsule_9f,'Hep 6-OMe').
adds_part(brendan,cje,capsule,capsule_13c,'Hep 6-OMe').
adds_part(brendan,cje,capsule,capsule_13d,'Hep 6-OMe').
adds_part(brendan,cje,capsule,capsule_13e,'Hep 6-OMe').
adds_part(brendan,cje,capsule,capsule_13f,'Hep 6-OMe').
adds_part(brendan,cje,capsule,capsule_17c,'Hep 6-OMe').
adds_part(brendan,cje,capsule,capsule_17d,'Hep 6-OMe').
adds_part(brendan,cje,capsule,capsule_17e,'Hep 6-OMe').
adds_part(brendan,cje,capsule,capsule_17f,'Hep 6-OMe').
adds_part(brendan,cje,capsule,capsule_21c,'Hep 6-OMe').
adds_part(brendan,cje,capsule,capsule_21d,'Hep 6-OMe').
adds_part(brendan,cje,capsule,capsule_21e,'Hep 6-OMe').
adds_part(brendan,cje,capsule,capsule_21f,'Hep 6-OMe').

%% GalfNAc

adds_part(brendan,cje,capsule,capsule_ngro22,'GalfNAc').
adds_part(brendan,cje,capsule,capsule_ngro23,'GalfNAc').
adds_part(brendan,cje,capsule,capsule_ngro24,'GalfNAc').
adds_part(brendan,cje,capsule,capsule_ngro24a,'GalfNAc').
adds_part(brendan,cje,capsule,capsule_ngro25,'GalfNAc').
adds_part(brendan,cje,capsule,capsule_ngro26,'GalfNAc').
adds_part(brendan,cje,capsule,capsule_ngro26a,'GalfNAc').
adds_part(brendan,cje,capsule,capsule_ngro26b,'GalfNAc').
adds_part(brendan,cje,capsule,capsule_ngro26c,'GalfNAc').
adds_part(brendan,cje,capsule,capsule_etn22,'GalfNAc').
adds_part(brendan,cje,capsule,capsule_etn23,'GalfNAc').
adds_part(brendan,cje,capsule,capsule_etn24,'GalfNAc').
adds_part(brendan,cje,capsule,capsule_etn24a,'GalfNAc').
adds_part(brendan,cje,capsule,capsule_etn25,'GalfNAc').
adds_part(brendan,cje,capsule,capsule_etn26,'GalfNAc').
adds_part(brendan,cje,capsule,capsule_etn26a,'GalfNAc').
adds_part(brendan,cje,capsule,capsule_etn26b,'GalfNAc').
adds_part(brendan,cje,capsule,capsule_etn26c,'GalfNAc').
adds_part(brendan,cje,capsule,capsule_22,'GalfNAc').
adds_part(brendan,cje,capsule,capsule_23,'GalfNAc').
adds_part(brendan,cje,capsule,capsule_24,'GalfNAc').
adds_part(brendan,cje,capsule,capsule_24a,'GalfNAc').
adds_part(brendan,cje,capsule,capsule_25,'GalfNAc').
adds_part(brendan,cje,capsule,capsule_26,'GalfNAc').
adds_part(brendan,cje,capsule,capsule_26a,'GalfNAc').
adds_part(brendan,cje,capsule,capsule_26b,'GalfNAc').
adds_part(brendan,cje,capsule,capsule_26c,'GalfNAc').

%% GalfNAc OMePN

adds_part(brendan,cje,capsule,capsule_ngro27,'GalfNAc OMePN').
adds_part(brendan,cje,capsule,capsule_ngro28,'GalfNAc OMePN').
adds_part(brendan,cje,capsule,capsule_ngro29,'GalfNAc OMePN').
adds_part(brendan,cje,capsule,capsule_ngro29a,'GalfNAc OMePN').
adds_part(brendan,cje,capsule,capsule_ngro30,'GalfNAc OMePN').
adds_part(brendan,cje,capsule,capsule_ngro31,'GalfNAc OMePN').
adds_part(brendan,cje,capsule,capsule_ngro31a,'GalfNAc OMePN').
```

```
adds_part(brendan,cje,capsule,capsule_ngro31b,'GalfNAc OMePN').
adds_part(brendan,cje,capsule,capsule_ngro31c,'GalfNAc OMePN').
adds_part(brendan,cje,capsule,capsule_ngro32,'GalfNAc OMePN').
adds_part(brendan,cje,capsule,capsule_ngro33,'GalfNAc OMePN').
adds_part(brendan,cje,capsule,capsule_ngro34,'GalfNAc OMePN').
adds_part(brendan,cje,capsule,capsule_ngro34a,'GalfNAc OMePN').
adds_part(brendan,cje,capsule,capsule_ngro35,'GalfNAc OMePN').
adds_part(brendan,cje,capsule,capsule_ngro36,'GalfNAc OMePN').
adds_part(brendan,cje,capsule,capsule_ngro36a,'GalfNAc OMePN').
adds_part(brendan,cje,capsule,capsule_ngro36b,'GalfNAc OMePN').
adds_part(brendan,cje,capsule,capsule_ngro36c,'GalfNAc OMePN').
adds_part(brendan,cje,capsule,capsule_etn27,'GalfNAc OMePN').
adds_part(brendan,cje,capsule,capsule_etn28,'GalfNAc OMePN').
adds_part(brendan,cje,capsule,capsule_etn29,'GalfNAc OMePN').
adds_part(brendan,cje,capsule,capsule_etn29a,'GalfNAc OMePN').
adds_part(brendan,cje,capsule,capsule_etn30,'GalfNAc OMePN').
adds_part(brendan,cje,capsule,capsule_etn31,'GalfNAc OMePN').
adds_part(brendan,cje,capsule,capsule_etn31a,'GalfNAc OMePN').
adds_part(brendan,cje,capsule,capsule_etn31b,'GalfNAc OMePN').
adds_part(brendan,cje,capsule,capsule_etn31c,'GalfNAc OMePN').
adds_part(brendan,cje,capsule,capsule_etn32,'GalfNAc OMePN').
adds_part(brendan,cje,capsule,capsule_etn33,'GalfNAc OMePN').
adds_part(brendan,cje,capsule,capsule_etn34,'GalfNAc OMePN').
adds_part(brendan,cje,capsule,capsule_etn34a,'GalfNAc OMePN').
adds_part(brendan,cje,capsule,capsule_etn35,'GalfNAc OMePN').
adds_part(brendan,cje,capsule,capsule_etn36,'GalfNAc OMePN').
adds_part(brendan,cje,capsule,capsule_etn36a,'GalfNAc OMePN').
adds_part(brendan,cje,capsule,capsule_etn36b,'GalfNAc OMePN').
adds_part(brendan,cje,capsule,capsule_etn36c,'GalfNAc OMePN').
adds_part(brendan,cje,capsule,capsule_27,'GalfNAc OMePN').
adds_part(brendan,cje,capsule,capsule_28,'GalfNAc OMePN').
adds_part(brendan,cje,capsule,capsule_29,'GalfNAc OMePN').
adds_part(brendan,cje,capsule,capsule_29a,'GalfNAc OMePN').
adds_part(brendan,cje,capsule,capsule_30,'GalfNAc OMePN').
adds_part(brendan,cje,capsule,capsule_31,'GalfNAc OMePN').
adds_part(brendan,cje,capsule,capsule_31a,'GalfNAc OMePN').
adds_part(brendan,cje,capsule,capsule_31b,'GalfNAc OMePN').
adds_part(brendan,cje,capsule,capsule_31c,'GalfNAc OMePN').
adds_part(brendan,cje,capsule,capsule_32,'GalfNAc OMePN').
adds_part(brendan,cje,capsule,capsule_33,'GalfNAc OMePN').
adds_part(brendan,cje,capsule,capsule_34,'GalfNAc OMePN').
adds_part(brendan,cje,capsule,capsule_34a,'GalfNAc OMePN').
adds_part(brendan,cje,capsule,capsule_35,'GalfNAc OMePN').
adds_part(brendan,cje,capsule,capsule_36,'GalfNAc OMePN').
adds_part(brendan,cje,capsule,capsule_36a,'GalfNAc OMePN').
adds_part(brendan,cje,capsule,capsule_36b,'GalfNAc OMePN').
adds_part(brendan,cje,capsule,capsule_36c,'GalfNAc OMePN').

%% Ribf

adds_part(brendan,cje,capsule,capsule_ngro37,'Ribf').
adds_part(brendan,cje,capsule,capsule_ngro38,'Ribf').
adds_part(brendan,cje,capsule,capsule_ngro39,'Ribf').
adds_part(brendan,cje,capsule,capsule_ngro39a,'Ribf').
```

```
adds_part(brendan,cje,capsule,capsule_ngro40,'Ribf').
adds_part(brendan,cje,capsule,capsule_ngro41,'Ribf').
adds_part(brendan,cje,capsule,capsule_ngro41a,'Ribf').
adds_part(brendan,cje,capsule,capsule_ngro41b,'Ribf').
adds_part(brendan,cje,capsule,capsule_ngro41c,'Ribf').
adds_part(brendan,cje,capsule,capsule_ngro42,'Ribf').
adds_part(brendan,cje,capsule,capsule_ngro43,'Ribf').
adds_part(brendan,cje,capsule,capsule_ngro44,'Ribf').
adds_part(brendan,cje,capsule,capsule_ngro44a,'Ribf').
adds_part(brendan,cje,capsule,capsule_ngro45,'Ribf').
adds_part(brendan,cje,capsule,capsule_ngro46,'Ribf').
adds_part(brendan,cje,capsule,capsule_ngro46a,'Ribf').
adds_part(brendan,cje,capsule,capsule_ngro46b,'Ribf').
adds_part(brendan,cje,capsule,capsule_ngro46c,'Ribf').
adds_part(brendan,cje,capsule,capsule_etn37,'Ribf').
adds_part(brendan,cje,capsule,capsule_etn38,'Ribf').
adds_part(brendan,cje,capsule,capsule_etn39,'Ribf').
adds_part(brendan,cje,capsule,capsule_etn39a,'Ribf').
adds_part(brendan,cje,capsule,capsule_etn40,'Ribf').
adds_part(brendan,cje,capsule,capsule_etn41,'Ribf').
adds_part(brendan,cje,capsule,capsule_etn41a,'Ribf').
adds_part(brendan,cje,capsule,capsule_etn41b,'Ribf').
adds_part(brendan,cje,capsule,capsule_etn41c,'Ribf').
adds_part(brendan,cje,capsule,capsule_etn42,'Ribf').
adds_part(brendan,cje,capsule,capsule_etn43,'Ribf').
adds_part(brendan,cje,capsule,capsule_etn44,'Ribf').
adds_part(brendan,cje,capsule,capsule_etn44a,'Ribf').
adds_part(brendan,cje,capsule,capsule_etn45,'Ribf').
adds_part(brendan,cje,capsule,capsule_etn46,'Ribf').
adds_part(brendan,cje,capsule,capsule_etn46a,'Ribf').
adds_part(brendan,cje,capsule,capsule_etn46b,'Ribf').
adds_part(brendan,cje,capsule,capsule_etn46c,'Ribf').
adds_part(brendan,cje,capsule,capsule_37,'Ribf').
adds_part(brendan,cje,capsule,capsule_38,'Ribf').
adds_part(brendan,cje,capsule,capsule_39,'Ribf').
adds_part(brendan,cje,capsule,capsule_39a,'Ribf').
adds_part(brendan,cje,capsule,capsule_40,'Ribf').
adds_part(brendan,cje,capsule,capsule_41,'Ribf').
adds_part(brendan,cje,capsule,capsule_41a,'Ribf').
adds_part(brendan,cje,capsule,capsule_41b,'Ribf').
adds_part(brendan,cje,capsule,capsule_41c,'Ribf').
adds_part(brendan,cje,capsule,capsule_42,'Ribf').
adds_part(brendan,cje,capsule,capsule_43,'Ribf').
adds_part(brendan,cje,capsule,capsule_44,'Ribf').
adds_part(brendan,cje,capsule,capsule_44a,'Ribf').
adds_part(brendan,cje,capsule,capsule_45,'Ribf').
adds_part(brendan,cje,capsule,capsule_46,'Ribf').
adds_part(brendan,cje,capsule,capsule_46a,'Ribf').
adds_part(brendan,cje,capsule,capsule_46b,'Ribf').
adds_part(brendan,cje,capsule,capsule_46c,'Ribf').

%%%%%%%%%%%%%%%%%%%%%%%%%%%%%%%%%%%%%%%%%%%%%%%%%%%%%%%%%%%%%%%%%%%%%%%%%%%%%%%%
```

```
neighbour_reaction(capsule_0,capsule_pre_pre_glca).
neighbour_reaction(capsule_pre_pre_glca,capsule_0).
neighbour_reaction(capsule_pre_glca,capsule_pre_pre_glca).
neighbour_reaction(capsule_pre_pre_glca,capsule_pre_glca).

neighbour_reaction(capsule_0,capsule_ngro1c).
neighbour_reaction(capsule_ngro1c,capsule_0).

neighbour_reaction(capsule_pre_pre_galfnac,capsule_22).
neighbour_reaction(capsule_22,capsule_pre_pre_galfnac).
neighbour_reaction(capsule_pre_pre_galfnac,capsule_pre_galfnac).
neighbour_reaction(capsule_pre_galfnac,capsule_pre_pre_galfnac).

neighbour_reaction(capsule_ngro36a,capsule_22).
neighbour_reaction(capsule_22,capsule_ngro36a).

neighbour_reaction(capsule_pre_pre_ribf,capsule_37).
neighbour_reaction(capsule_37,capsule_pre_pre_ribf).

neighbour_reaction(capsule_22,capsule_37).
neighbour_reaction(capsule_37,capsule_22).

neighbour_reaction(capsule_ngro36a,capsule_37).
neighbour_reaction(capsule_37,capsule_ngro36a).

neighbour_reaction(capsule_pre_glca,capsule_pre_hep).
neighbour_reaction(capsule_pre_hep,capsule_pre_glca).
neighbour_reaction(capsule_pre_glca,capsule_1).
neighbour_reaction(capsule_1,capsule_pre_glca).
neighbour_reaction(capsule_0,capsule_1).
neighbour_reaction(capsule_1,capsule_0).

neighbour_reaction(capsule_pre_glca,capsule_ngro21c).
neighbour_reaction(capsule_pre_glca,capsule_ngro21a).
neighbour_reaction(capsule_ngro21c,capsule_pre_glca).
neighbour_reaction(capsule_ngro21a,capsule_pre_glca).

neighbour_reaction(capsule_pre_pre_glca,capsule_pre_hep).
neighbour_reaction(capsule_pre_pre_glca,capsule_ngro21c).
neighbour_reaction(capsule_pre_pre_glca,capsule_ngro21a).
neighbour_reaction(capsule_pre_hep,capsule_pre_pre_glca).
neighbour_reaction(capsule_ngro21c,capsule_pre_pre_glca).
neighbour_reaction(capsule_ngro21a,capsule_pre_pre_glca).

neighbour_reaction(capsule_pre_hep,capsule_ngro21c).
neighbour_reaction(capsule_pre_hep,capsule_ngro21a).
neighbour_reaction(capsule_ngro21c,capsule_pre_hep).
neighbour_reaction(capsule_ngro21a,capsule_pre_hep).

neighbour_reaction(capsule_ngro21c,capsule_ngro21a).
neighbour_reaction(capsule_ngro21a,capsule_ngro21c).

neighbour_reaction(capsule_pre_pre_galfnac,capsule_ngro36a).
neighbour_reaction(capsule_pre_pre_galfnac,capsule_pre_pre_omepn).
```

```
neighbour_reaction(capsule_ngro36a,capsule_pre_pre_galfnac).
neighbour_reaction(capsule_pre_pre_omepn,capsule_pre_pre_galfnac).

neighbour_reaction(capsule_ngro21a,capsule_pre_pre_omepn).
neighbour_reaction(capsule_pre_pre_omepn,capsule_ngro21a).
neighbour_reaction(capsule_ngro36a,capsule_pre_pre_omepn).
neighbour_reaction(capsule_pre_pre_omepn,capsule_ngro36a).

neighbour_reaction(capsule_pre_pre_glca,capsule_pre_hep).
neighbour_reaction(capsule_pre_hep,capsule_pre_pre_glca).

neighbour_reaction(capsule_ngro1c,capsule_ngro1c).
neighbour_reaction(capsule_ngro1c,capsule_ngro21a).

%%%%%%%%%%%%%%%%%%%%%%%%%%%%%%%%%%%%%%%%%%%%%%%%%%%%%%%%%%%%%%%%%%%%%%%%
%%%%%%%%%%%%%%%%%%%%%%
% additional reaction information related to pre-cursors
%

reaction(brendan,cje,capsule_pre_pre_omepn,'pre-pre-omepn','pre-omepn',1).
chain(capsule_pre_pre_omepn).


reaction(brendan,cje,capsule_pre_pre_ribf,'pre-pre-ribf','pre-ribf',1).
chain(capsule_pre_pre_ribf).


reaction(brendan,cje,capsule_pre_pre_glca,'pre-pre-glca','pre-glca',1).

reaction(brendan,cje,capsule_pre_pre_galfnac,'pre-pre-galfnac','pre-galfnac',1).

%reaction(brendan,cje,capsule_pre_pre_omepn,'pre-pre-omepn','pre-omepn',1).

reaction(brendan,cje,capsule_pre_pre_hep,'pre-pre-hep','pre-hep',1).
chain(capsule_pre_pre_hep).

reaction(brendan,cje,capsule_pre_hep,'glca6ngro','glca6ngro+hep',1).
reaction(brendan,cje,capsule_pre_hep,'pre-hep','glca6ngro+hep',1).
```

```
%       FILE GLYCAN_STRUCTURE_PL
%       The Campy CPS glycan structures. This includes the definition of struct_next
%       which states that one glycan structure is related to another by the addition
%       of a single chemical group

struct_next('pre-glca','glca').
struct_next('pre-galfnac','galfnac').
struct_next('pre-ribf','ribf').

struct_next('glca','glca-galfnac').

struct_next('glca-galfnac',
        'glca-galfnac-ribf').

struct_next('galfnac-ribf',
        'glca-galfnac-ribf').

struct_next('glca-ribf',
        'glca-galfnac-ribf').

struct_next('glca-galfnac-ribf',
        'glca6ngro-galfnac-ribf').


struct_next('glca6ngro-galfnac-ribf',
        'glca6ngro-galfnac+omepn-ribf').


struct_next('glca6ngro-galfnac+omepn-ribf',
        'glca6ngro+hep-galfnac+omepn-ribf').

struct_next('glca6ngro+hep-galfnac+omepn-ribf',
        'glca6ngro+hep+6ome-galfnac+omepn-ribf').

struct_next('glca6ngro+hep+6ome-galfnac-ribf',
        'glca6ngro+hep+6ome-galfnac+omepn-ribf').

struct_next('glca6ngro+hep+6ome-galfnac-ribf',
        'glca6ngro+hep+omepn+6ome-galfnac-ribf').

struct_next('glca6ngro+hep+omepn+6ome-galfnac-ribf',
        'glca6ngro+hep+omepn+6ome-galfnac+omepn-ribf').

struct_next('glca6ngro+hep+6ome-galfnac+omepn-ribf',
        'glca6ngro+hep+omepn+6ome-galfnac+omepn-ribf').



struct_observable('glca6ngro+hep+6ome-galfnac-ribf',
            'glca6ngro+hep+6ome-galfnac-ribf').
struct_observable('glca6ngro+hep+6ome-galfnac+omepn-ribf',
            'glca6ngro+hep+6ome-galfnac+omepn-ribf').
struct_observable('glca6ngro+hep+omepn+6ome-galfnac-ribf',
```

```
                'glca6ngro+hep+omepn+6ome-galfnac-ribf').
struct_observable('glca6ngro-galfnac+omepn-ribf',
                'glca6ngro-galfnac+omepn-ribf').
struct_observable('glca6ngro+hep-galfnac+omepn-ribf',
                'glca6ngro+hep-galfnac+omepn-ribf').
struct_observable('glca6ngro-galfnac+omepn-ribf',
                'glca6ngro-galfnac+omepn-ribf').

struct_observable('glca-galfnac','acapsular').
struct_observable('galfnac-ribf','acapsular').
struct_observable('glca-ribf','acapsular').
struct_observable('glca','acapsular').
struct_observable('pre-glca','acapsular').

struct_observable('pre_pre-glca','pre_pre-glca').
struct_observable('pre_pre-galfnac','pre_pre-galfnac').
struct_observable('pre_pre-hep','pre_pre-hep').

struct_observable('glca-galfnac','glca-galfnac').
struct_observable('galfnac-ribf','galfnac-ribf').
struct_observable('glca-ribf','glca-ribf').
```

```
%	FILE MUTANTS_PL
%	The gene knock-out experiments for Campy CPS. The data is mainly compiled from
%	previously published data (McNally, 2007). The data is represented by instances
%	of the 'knockout_observable' predicate which  states when a specific gene is
%	knocked out this result causes a particular glycan structure to be the largest
%	synthesised

knockout_observable(cj1416c,'glca6ngro+hep+6ome-galfnac-ribf').
knockout_observable(cj1417c,'glca6ngro+hep+6ome-galfnac-ribf').
knockout_observable(cj1418c,'glca6ngro+hep+6ome-galfnac-ribf').
knockout_observable(cj1421c,'glca6ngro+hep+6ome-galfnac+omepn-ribf').
knockout_observable(cj1422c,'glca6ngro+hep+omepn+6ome-galfnac-ribf').
knockout_observable(cj1423c,'glca6ngro-galfnac+omepn-ribf').
knockout_observable(cj1424c,'glca6ngro-galfnac+omepn-ribf').
knockout_observable(cj1425c,'glca6ngro-galfnac+omepn-ribf').
knockout_observable(cj1426c,'glca6ngro+hep-galfnac+omepn-ribf').
knockout_observable(cj1427c,'glca6ngro-galfnac+omepn-ribf').
knockout_observable(cj1428c,'glca6ngro-galfnac+omepn-ribf').
knockout_observable(cj1430c,'glca6ngro-galfnac+omepn-ribf').
knockout_observable(cj1431c,'glca6ngro-galfnac+omepn-ribf').
knockout_observable(cj1432c,'acapsular').
knockout_observable(cj1434c,'acapsular').
knockout_observable(cj1435c,'acapsular').
knockout_observable(cj1437c,'acapsular').
knockout_observable(cj1438c,'acapsular').
knockout_observable(cj1439c,'acapsular').
knockout_observable(cj1440c,'acapsular').
knockout_observable(cj1441c,'acapsular').
knockout_observable(cj1442c,'acapsular').
knockout_observable(cj1448c,'acapsular').
```

```
%	FILE STRAINS_PL
%	The cross-strain genomic data for the CPS loci originate from an in-progress study
%	in which 270 C. jejuni isolates were analysed by comparative phylogenomics (whole
%	genome comparisons of bacteria using DNA microarrays, combined with Bayesian-based
%	algorithms, to model the phylogeny), using a previously published method (Champion,
%	2005).The data includes predicates 'absent' and 'occurs'  which state that a particular
%	gene is absent or present from a particular strain.

occurs(strainClinicalG1,cj1413c).
occurs(strainClinicalG3,cj1413c).
occurs(strainChickenicken11974,cj1413c).
occurs(strainChickenicken13249,cj1413c).
occurs(strainChickenicken13411,cj1413c).
occurs(strainClinical44811,cj1413c).
occurs(strainClinical48612,cj1413c).
occurs(strainClinical58473,cj1413c).
occurs(strainChicken11848,cj1413c).
occurs(strainChicken12567,cj1413c).
occurs(strainChicken11919,cj1413c).
occurs(strainChicken11973,cj1413c).
occurs(strainChicken13713,cj1413c).
occurs(strainBeach1791,cj1413c).
occurs(strainClinical43205,cj1413c).
occurs(strainClinical33106,cj1413c).
occurs(strainClinicall38556,cj1413c).
occurs(strainClinicall39182,cj1413c).
occurs(strainClinical44933,cj1413c).
occurs(strainClinical45631,cj1413c).
occurs(strainClinical55320,cj1413c).
occurs(strainClinical55703,cj1413c).
occurs(strainClinical30280,cj1413c).
occurs(strainClinical30328,cj1413c).
occurs(strainClinical41651,cj1413c).
occurs(strainClinical32799,cj1413c).
occurs(strainClinical43983,cj1413c).
occurs(strainClinical40671,cj1413c).
occurs(strainClinical44958,cj1413c).
occurs(strainClinical52331,cj1413c).
occurs(strainClinical56281,cj1413c).
occurs(strainClinical56282,cj1413c).
occurs(strainClinical56832,cj1413c).
occurs(strainOvine12241,cj1413c).
occurs(strainOvine12481,cj1413c).
occurs(strainBovinevineine13305,cj1413c).
occurs(strainChickenicken12912,cj1413c).
occurs(strainChickenicken11818,cj1413c).
occurs(strainChicken12196,cj1413c).
occurs(strainChickenicken12450,cj1413c).
occurs(strainChickenicken12487,cj1413c).
occurs(strainChickenicken13082,cj1413c).
occurs(strainChickenicken13040,cj1413c).
occurs(strainChickenicken11856,cj1413c).
occurs(strainClinical36069,cj1413c).
```

occurs(strainChicken40209,cj1413c).
occurs(strainBeach1793,cj1413c).
occurs(strainClinical81116,cj1413c).
occurs(strainClinical36439,cj1413c).
occurs(strainClinical56519,cj1413c).
occurs(strainClinical32787,cj1413c).
occurs(strainClinicall31467,cj1413c).
occurs(strainClinical44119,cj1413c).
occurs(strainClinical34007,cj1413c).
occurs(strainClinical38762,cj1413c).
occurs(strainClinicalinical15168,cj1413c).
occurs(strainClinical18836,cj1413c).
occurs(strainClinicalM1,cj1413c).
occurs(strainClinical36860,cj1413c).
occurs(strainClinical40917,cj1413c).
occurs(strainClinical38857,cj1413c).
occurs(strainCjejuniRM1221,cj1413c).
occurs(strainChicken47693,cj1413c).
occurs(strainClinical63326,cj1413c).
occurs(strainClinical64555,cj1413c).
occurs(strainClinical59364,cj1413c).
occurs(strainClinical59424,cj1413c).
occurs(strainClinical33084,cj1413c).
occurs(strainClinical31481,cj1413c).
occurs(strainClinical39828,cj1413c).
occurs(strainBeach1771,cj1413c).
occurs(strainClinical53250,cj1413c).
occurs(strainClinical45557,cj1413c).
occurs(strainClinical47939,cj1413c).
occurs(strainClinicalG1,cj1414c).
occurs(strainChickenicken11974,cj1414c).
occurs(strainChickenicken13249,cj1414c).
occurs(strainChickenicken13411,cj1414c).
occurs(strainClinical44811,cj1414c).
occurs(strainClinical48612,cj1414c).
occurs(strainClinical58473,cj1414c).
occurs(strainChicken11848,cj1414c).
occurs(strainChicken12567,cj1414c).
occurs(strainChicken11919,cj1414c).
occurs(strainChicken11973,cj1414c).
occurs(strainChicken13713,cj1414c).
occurs(strainBeach1791,cj1414c).
occurs(strainClinical43205,cj1414c).
occurs(strainClinical33106,cj1414c).
occurs(strainClinicall38556,cj1414c).
occurs(strainClinicall39182,cj1414c).
occurs(strainClinical44933,cj1414c).
occurs(strainClinical45631,cj1414c).
occurs(strainClinical55320,cj1414c).
occurs(strainClinical55703,cj1414c).
occurs(strainClinical30280,cj1414c).
occurs(strainClinical30328,cj1414c).
occurs(strainClinical41651,cj1414c).
occurs(strainClinical40671,cj1414c).

occurs(strainClinical44958,cj1414c).
occurs(strainClinical52331,cj1414c).
occurs(strainClinical56281,cj1414c).
occurs(strainClinical56282,cj1414c).
occurs(strainClinical56832,cj1414c).
occurs(strainOvine12241,cj1414c).
occurs(strainOvine12481,cj1414c).
occurs(strainChickenicken12912,cj1414c).
occurs(strainChicken12196,cj1414c).
occurs(strainChickenicken12450,cj1414c).
occurs(strainChickenicken12487,cj1414c).
occurs(strainChickenicken13082,cj1414c).
occurs(strainChickenicken13040,cj1414c).
occurs(strainChickenicken11856,cj1414c).
occurs(strainClinical36069,cj1414c).
occurs(strainChicken40209,cj1414c).
occurs(strainBeach1793,cj1414c).
occurs(strainClinical56519,cj1414c).
occurs(strainClinical44119,cj1414c).
occurs(strainClinical34007,cj1414c).
occurs(strainClinicalinical15168,cj1414c).
occurs(strainClinical18836,cj1414c).
occurs(strainClinical38857,cj1414c).
occurs(strainCjejuniRM1221,cj1414c).
occurs(strainChicken47693,cj1414c).
occurs(strainClinical63326,cj1414c).
occurs(strainClinical64555,cj1414c).
occurs(strainClinical59364,cj1414c).
occurs(strainClinical59424,cj1414c).
occurs(strainClinical33084,cj1414c).
occurs(strainClinical31481,cj1414c).
occurs(strainClinical39828,cj1414c).
occurs(strainBeach1771,cj1414c).
occurs(strainClinical53250,cj1414c).
occurs(strainClinical45557,cj1414c).
occurs(strainClinical47939,cj1414c).
occurs(strainClinicalG1,cj1415c).
occurs(strainClinicalG3,cj1415c).
occurs(strainChickenicken11974,cj1415c).
occurs(strainChickenicken13249,cj1415c).
occurs(strainChickenicken13411,cj1415c).
occurs(strainClinical44811,cj1415c).
occurs(strainClinical48612,cj1415c).
occurs(strainClinical58473,cj1415c).
occurs(strainChicken11848,cj1415c).
occurs(strainChicken12567,cj1415c).
occurs(strainChicken11919,cj1415c).
occurs(strainChicken11973,cj1415c).
occurs(strainChicken13713,cj1415c).
occurs(strainBeach1791,cj1415c).
occurs(strainClinical43205,cj1415c).
occurs(strainClinical33106,cj1415c).
occurs(strainClinical55703,cj1415c).
occurs(strainClinical30280,cj1415c).

occurs(strainClinical30328,cj1415c).
occurs(strainClinical41651,cj1415c).
occurs(strainClinical32799,cj1415c).
occurs(strainClinical43983,cj1415c).
occurs(strainClinical40671,cj1415c).
occurs(strainClinical44958,cj1415c).
occurs(strainClinical52331,cj1415c).
occurs(strainClinical56281,cj1415c).
occurs(strainClinical56282,cj1415c).
occurs(strainClinical56832,cj1415c).
occurs(strainOvine12241,cj1415c).
occurs(strainOvine12481,cj1415c).
occurs(strainBovinevineine13305,cj1415c).
occurs(strainChickenicken12912,cj1415c).
occurs(strainChickenicken11818,cj1415c).
occurs(strainChicken12196,cj1415c).
occurs(strainChickenicken12450,cj1415c).
occurs(strainChickenicken12487,cj1415c).
occurs(strainChickenicken13082,cj1415c).
occurs(strainChickenicken13040,cj1415c).
occurs(strainChickenicken11856,cj1415c).
occurs(strainChicken40209,cj1415c).
occurs(strainBeach1793,cj1415c).
occurs(strainClinical32787,cj1415c).
occurs(strainClinicall31467,cj1415c).
occurs(strainClinical38762,cj1415c).
occurs(strainClinicalinical15168,cj1415c).
occurs(strainClinical18836,cj1415c).
occurs(strainClinical38857,cj1415c).
occurs(strainCjejuniRM1221,cj1415c).
occurs(strainClinical33084,cj1415c).
occurs(strainClinical31481,cj1415c).
occurs(strainClinical39828,cj1415c).
occurs(strainClinical45557,cj1415c).
occurs(strainClinicalG1,cj1416c).
occurs(strainClinicalG3,cj1416c).
occurs(strainChickenicken11974,cj1416c).
occurs(strainChickenicken13249,cj1416c).
occurs(strainChickenicken13411,cj1416c).
occurs(strainClinical44811,cj1416c).
occurs(strainClinical48612,cj1416c).
occurs(strainClinical58473,cj1416c).
occurs(strainChicken11848,cj1416c).
occurs(strainChicken12567,cj1416c).
occurs(strainChicken11919,cj1416c).
occurs(strainChicken11973,cj1416c).
occurs(strainChicken13713,cj1416c).
occurs(strainBeach1791,cj1416c).
occurs(strainClinical43205,cj1416c).
occurs(strainClinical33106,cj1416c).
occurs(strainClinical55320,cj1416c).
occurs(strainClinical55703,cj1416c).
occurs(strainClinical30280,cj1416c).
occurs(strainClinical30328,cj1416c).

occurs(strainClinical41651,cj1416c).
occurs(strainClinical32799,cj1416c).
occurs(strainClinical43983,cj1416c).
occurs(strainClinical40671,cj1416c).
occurs(strainClinical44958,cj1416c).
occurs(strainClinical52331,cj1416c).
occurs(strainClinical56281,cj1416c).
occurs(strainClinical56282,cj1416c).
occurs(strainClinical56832,cj1416c).
occurs(strainOvine12241,cj1416c).
occurs(strainOvine12481,cj1416c).
occurs(strainBovinevineine13305,cj1416c).
occurs(strainChickenicken12912,cj1416c).
occurs(strainChickenicken11818,cj1416c).
occurs(strainChicken12196,cj1416c).
occurs(strainChickenicken12450,cj1416c).
occurs(strainChickenicken12487,cj1416c).
occurs(strainChickenicken13082,cj1416c).
occurs(strainChickenicken13040,cj1416c).
occurs(strainChickenicken11856,cj1416c).
occurs(strainChicken40209,cj1416c).
occurs(strainBeach1793,cj1416c).
occurs(strainClinical81116,cj1416c).
occurs(strainClinical56519,cj1416c).
occurs(strainClinical32787,cj1416c).
occurs(strainClinicall31467,cj1416c).
occurs(strainClinical38762,cj1416c).
occurs(strainClinicalinical15168,cj1416c).
occurs(strainClinical18836,cj1416c).
occurs(strainClinicalM1,cj1416c).
occurs(strainClinical36860,cj1416c).
occurs(strainClinical40917,cj1416c).
occurs(strainClinical38857,cj1416c).
occurs(strainChicken47693,cj1416c).
occurs(strainClinical33084,cj1416c).
occurs(strainClinical31481,cj1416c).
occurs(strainClinical39828,cj1416c).
occurs(strainClinical53250,cj1416c).
occurs(strainClinical45557,cj1416c).
occurs(strainClinicalG1,cj1417c).
occurs(strainClinicalG3,cj1417c).
occurs(strainChickenicken11974,cj1417c).
occurs(strainChickenicken13249,cj1417c).
occurs(strainChickenicken13411,cj1417c).
occurs(strainClinical44811,cj1417c).
occurs(strainClinical48612,cj1417c).
occurs(strainClinical58473,cj1417c).
occurs(strainChicken11848,cj1417c).
occurs(strainChicken12567,cj1417c).
occurs(strainChicken11919,cj1417c).
occurs(strainChicken11973,cj1417c).
occurs(strainChicken13713,cj1417c).
occurs(strainBeach1791,cj1417c).
occurs(strainClinical43205,cj1417c).

occurs(strainClinical33106,cj1417c).
occurs(strainClinical55703,cj1417c).
occurs(strainClinical30280,cj1417c).
occurs(strainClinical30328,cj1417c).
occurs(strainClinical41651,cj1417c).
occurs(strainClinical32799,cj1417c).
occurs(strainClinical43983,cj1417c).
occurs(strainClinical40671,cj1417c).
occurs(strainClinical44958,cj1417c).
occurs(strainClinical52331,cj1417c).
occurs(strainClinical56281,cj1417c).
occurs(strainClinical56282,cj1417c).
occurs(strainClinical56832,cj1417c).
occurs(strainOvine12241,cj1417c).
occurs(strainOvine12481,cj1417c).
occurs(strainBovinevineine13305,cj1417c).
occurs(strainChickenicken12912,cj1417c).
occurs(strainChickenicken11818,cj1417c).
occurs(strainChicken12196,cj1417c).
occurs(strainChickenicken12450,cj1417c).
occurs(strainChickenicken12487,cj1417c).
occurs(strainChickenicken13082,cj1417c).
occurs(strainChickenicken13040,cj1417c).
occurs(strainChickenicken11856,cj1417c).
occurs(strainChicken40209,cj1417c).
occurs(strainBeach1793,cj1417c).
occurs(strainClinical81116,cj1417c).
occurs(strainClinical56519,cj1417c).
occurs(strainClinical32787,cj1417c).
occurs(strainClinicall31467,cj1417c).
occurs(strainClinical38762,cj1417c).
occurs(strainClinicalinical15168,cj1417c).
occurs(strainClinical18836,cj1417c).
occurs(strainClinicalM1,cj1417c).
occurs(strainClinical36860,cj1417c).
occurs(strainClinical40917,cj1417c).
occurs(strainClinical38857,cj1417c).
occurs(strainChicken47693,cj1417c).
occurs(strainClinical33084,cj1417c).
occurs(strainClinical31481,cj1417c).
occurs(strainClinical39828,cj1417c).
occurs(strainClinical53250,cj1417c).
occurs(strainClinical45557,cj1417c).
occurs(strainClinicalG1,cj1418c).
occurs(strainClinicalG3,cj1418c).
occurs(strainChickenicken11974,cj1418c).
occurs(strainChickenicken13249,cj1418c).
occurs(strainChickenicken13411,cj1418c).
occurs(strainClinical44811,cj1418c).
occurs(strainClinical48612,cj1418c).
occurs(strainClinical58473,cj1418c).
occurs(strainChicken11848,cj1418c).
occurs(strainChicken12567,cj1418c).
occurs(strainChicken11919,cj1418c).

occurs(strainChicken11973,cj1418c).
occurs(strainChicken13713,cj1418c).
occurs(strainBeach1791,cj1418c).
occurs(strainClinical43205,cj1418c).
occurs(strainClinical33106,cj1418c).
occurs(strainClinical55703,cj1418c).
occurs(strainClinical30280,cj1418c).
occurs(strainClinical30328,cj1418c).
occurs(strainClinical41651,cj1418c).
occurs(strainClinical32799,cj1418c).
occurs(strainClinical43983,cj1418c).
occurs(strainClinical40671,cj1418c).
occurs(strainClinical44958,cj1418c).
occurs(strainClinical52331,cj1418c).
occurs(strainClinical56281,cj1418c).
occurs(strainClinical56282,cj1418c).
occurs(strainClinical56832,cj1418c).
occurs(strainOvine12241,cj1418c).
occurs(strainOvine12481,cj1418c).
occurs(strainBovinevineine13305,cj1418c).
occurs(strainChickenicken12912,cj1418c).
occurs(strainChickenicken11818,cj1418c).
occurs(strainChicken12196,cj1418c).
occurs(strainChickenicken12450,cj1418c).
occurs(strainChickenicken12487,cj1418c).
occurs(strainChickenicken13082,cj1418c).
occurs(strainChickenicken13040,cj1418c).
occurs(strainChickenicken11856,cj1418c).
occurs(strainChicken40209,cj1418c).
occurs(strainBeach1793,cj1418c).
occurs(strainClinical81116,cj1418c).
occurs(strainClinical56519,cj1418c).
occurs(strainClinical32787,cj1418c).
occurs(strainClinicall31467,cj1418c).
occurs(strainClinical38762,cj1418c).
occurs(strainClinicalinical15168,cj1418c).
occurs(strainClinical18836,cj1418c).
occurs(strainClinicalM1,cj1418c).
occurs(strainClinical36860,cj1418c).
occurs(strainClinical40917,cj1418c).
occurs(strainClinical38857,cj1418c).
occurs(strainChicken47693,cj1418c).
occurs(strainClinical33084,cj1418c).
occurs(strainClinical31481,cj1418c).
occurs(strainClinical39828,cj1418c).
occurs(strainClinical53250,cj1418c).
occurs(strainClinical45557,cj1418c).
occurs(strainClinicalG1,cj1419c).
occurs(strainClinicalG3,cj1419c).
occurs(strainChickenicken11974,cj1419c).
occurs(strainChickenicken13249,cj1419c).
occurs(strainChickenicken13411,cj1419c).
occurs(strainClinical44811,cj1419c).
occurs(strainClinical48612,cj1419c).

```
occurs(strainClinical58473,cj1419c).
occurs(strainChicken11848,cj1419c).
occurs(strainChicken12567,cj1419c).
occurs(strainChicken11919,cj1419c).
occurs(strainChicken11973,cj1419c).
occurs(strainChicken13713,cj1419c).
occurs(strainBeach1791,cj1419c).
occurs(strainClinical43205,cj1419c).
occurs(strainClinical33106,cj1419c).
occurs(strainClinical55703,cj1419c).
occurs(strainClinical30280,cj1419c).
occurs(strainClinical30328,cj1419c).
occurs(strainClinical41651,cj1419c).
occurs(strainClinical32799,cj1419c).
occurs(strainClinical43983,cj1419c).
occurs(strainClinical40671,cj1419c).
occurs(strainClinical44958,cj1419c).
occurs(strainClinical52331,cj1419c).
occurs(strainClinical56281,cj1419c).
occurs(strainClinical56282,cj1419c).
occurs(strainClinical56832,cj1419c).
occurs(strainOvine12241,cj1419c).
occurs(strainOvine12481,cj1419c).
occurs(strainBovinevineine13305,cj1419c).
occurs(strainChickenicken12912,cj1419c).
occurs(strainChickenicken11818,cj1419c).
occurs(strainChicken12196,cj1419c).
occurs(strainChickenicken12450,cj1419c).
occurs(strainChickenicken12487,cj1419c).
occurs(strainChickenicken13082,cj1419c).
occurs(strainChickenicken13040,cj1419c).
occurs(strainChickenicken11856,cj1419c).
occurs(strainChicken40209,cj1419c).
occurs(strainBeach1793,cj1419c).
occurs(strainClinical81116,cj1419c).
occurs(strainClinical56519,cj1419c).
occurs(strainClinical32787,cj1419c).
occurs(strainClinicall31467,cj1419c).
occurs(strainClinical38762,cj1419c).
occurs(strainClinicalinical15168,cj1419c).
occurs(strainClinical18836,cj1419c).
occurs(strainClinicalM1,cj1419c).
occurs(strainClinical36860,cj1419c).
occurs(strainClinical40917,cj1419c).
occurs(strainClinical38857,cj1419c).
occurs(strainChicken47693,cj1419c).
occurs(strainClinical33084,cj1419c).
occurs(strainClinical31481,cj1419c).
occurs(strainClinical39828,cj1419c).
occurs(strainClinical53250,cj1419c).
occurs(strainClinical45557,cj1419c).
occurs(strainChickenicken13411,cj1421c).
occurs(strainClinical44811,cj1421c).
occurs(strainClinical48612,cj1421c).
```

occurs(strainChicken11848,cj1421c).
occurs(strainChicken12567,cj1421c).
occurs(strainChicken11919,cj1421c).
occurs(strainChicken11973,cj1421c).
occurs(strainChicken13713,cj1421c).
occurs(strainBeach1791,cj1421c).
occurs(strainClinical55703,cj1421c).
occurs(strainClinical30280,cj1421c).
occurs(strainClinical30328,cj1421c).
occurs(strainChickenicken12450,cj1421c).
occurs(strainChickenicken12487,cj1421c).
occurs(strainChickenicken13082,cj1421c).
occurs(strainCjejuniRM1221,cj1421c).
occurs(strainChickenicken13411,cj1422c).
occurs(strainClinical44811,cj1422c).
occurs(strainClinical48612,cj1422c).
occurs(strainChicken11848,cj1422c).
occurs(strainChicken12567,cj1422c).
occurs(strainChicken11919,cj1422c).
occurs(strainChicken11973,cj1422c).
occurs(strainChicken13713,cj1422c).
occurs(strainBeach1791,cj1422c).
occurs(strainClinical55320,cj1422c).
occurs(strainClinical55703,cj1422c).
occurs(strainClinical30280,cj1422c).
occurs(strainClinical30328,cj1422c).
occurs(strainChickenicken12450,cj1422c).
occurs(strainChickenicken12487,cj1422c).
occurs(strainChickenicken13082,cj1422c).
occurs(strainChickenicken13411,cj1423c).
occurs(strainClinical44811,cj1423c).
occurs(strainClinical48612,cj1423c).
occurs(strainChicken11848,cj1423c).
occurs(strainChicken12567,cj1423c).
occurs(strainChicken11919,cj1423c).
occurs(strainChicken11973,cj1423c).
occurs(strainChicken13713,cj1423c).
occurs(strainBeach1791,cj1423c).
occurs(strainClinical43205,cj1423c).
occurs(strainClinical55320,cj1423c).
occurs(strainClinical55703,cj1423c).
occurs(strainClinical30280,cj1423c).
occurs(strainClinical30328,cj1423c).
occurs(strainClinical41651,cj1423c).
occurs(strainClinical44958,cj1423c).
occurs(strainClinical56281,cj1423c).
occurs(strainClinical56282,cj1423c).
occurs(strainChickenicken12450,cj1423c).
occurs(strainChickenicken12487,cj1423c).
occurs(strainChickenicken13082,cj1423c).
occurs(strainBeach1793,cj1423c).
occurs(strainClinical33084,cj1423c).
occurs(strainClinical53250,cj1423c).
occurs(strainChickenicken13411,cj1424c).

occurs(strainClinical44811,cj1424c).
occurs(strainClinical48612,cj1424c).
occurs(strainChicken11848,cj1424c).
occurs(strainChicken12567,cj1424c).
occurs(strainChicken11919,cj1424c).
occurs(strainChicken11973,cj1424c).
occurs(strainChicken13713,cj1424c).
occurs(strainBeach1791,cj1424c).
occurs(strainClinical43205,cj1424c).
occurs(strainClinicall38556,cj1424c).
occurs(strainClinicall39182,cj1424c).
occurs(strainClinical45631,cj1424c).
occurs(strainClinical55320,cj1424c).
occurs(strainClinical55703,cj1424c).
occurs(strainClinical30280,cj1424c).
occurs(strainClinical30328,cj1424c).
occurs(strainClinical41651,cj1424c).
occurs(strainClinical43983,cj1424c).
occurs(strainClinical40671,cj1424c).
occurs(strainClinical56282,cj1424c).
occurs(strainOvine12241,cj1424c).
occurs(strainOvine12481,cj1424c).
occurs(strainBovinevineine13305,cj1424c).
occurs(strainChickenicken12450,cj1424c).
occurs(strainChickenicken12487,cj1424c).
occurs(strainChickenicken13082,cj1424c).
occurs(strainChickenicken11856,cj1424c).
occurs(strainClinical36069,cj1424c).
occurs(strainClinical32787,cj1424c).
occurs(strainClinicall31467,cj1424c).
occurs(strainClinical44119,cj1424c).
occurs(strainClinical34007,cj1424c).
occurs(strainClinical38762,cj1424c).
occurs(strainClinical38857,cj1424c).
occurs(strainChicken47693,cj1424c).
occurs(strainClinical63326,cj1424c).
occurs(strainClinical64555,cj1424c).
occurs(strainClinical59364,cj1424c).
occurs(strainClinical59424,cj1424c).
occurs(strainClinical33084,cj1424c).
occurs(strainClinical31481,cj1424c).
occurs(strainBeach1771,cj1424c).
occurs(strainClinical53250,cj1424c).
occurs(strainChickenicken13411,cj1425c).
occurs(strainClinical44811,cj1425c).
occurs(strainClinical48612,cj1425c).
occurs(strainClinical58473,cj1425c).
occurs(strainChicken11848,cj1425c).
occurs(strainChicken12567,cj1425c).
occurs(strainChicken11919,cj1425c).
occurs(strainChicken11973,cj1425c).
occurs(strainChicken13713,cj1425c).
occurs(strainBeach1791,cj1425c).
occurs(strainClinical43205,cj1425c).

occurs(strainClinical33106,cj1425c).
occurs(strainClinical44933,cj1425c).
occurs(strainClinical55320,cj1425c).
occurs(strainClinical55703,cj1425c).
occurs(strainClinical30280,cj1425c).
occurs(strainClinical30328,cj1425c).
occurs(strainClinical41651,cj1425c).
occurs(strainClinical43983,cj1425c).
occurs(strainClinical40671,cj1425c).
occurs(strainClinical44958,cj1425c).
occurs(strainClinical56281,cj1425c).
occurs(strainClinical56282,cj1425c).
occurs(strainClinical56832,cj1425c).
occurs(strainOvine12241,cj1425c).
occurs(strainChicken12196,cj1425c).
occurs(strainChickenicken12450,cj1425c).
occurs(strainChickenicken12487,cj1425c).
occurs(strainChickenicken13082,cj1425c).
occurs(strainChickenicken13040,cj1425c).
occurs(strainChickenicken11856,cj1425c).
occurs(strainBeach1793,cj1425c).
occurs(strainClinical56519,cj1425c).
occurs(strainClinicall31467,cj1425c).
occurs(strainClinical38857,cj1425c).
occurs(strainChicken47693,cj1425c).
occurs(strainClinical64555,cj1425c).
occurs(strainClinical31481,cj1425c).
occurs(strainBeach1771,cj1425c).
occurs(strainClinical53250,cj1425c).
occurs(strainChickenicken13411,cj1426c).
occurs(strainClinical44811,cj1426c).
occurs(strainClinical48612,cj1426c).
occurs(strainChicken11848,cj1426c).
occurs(strainChicken12567,cj1426c).
occurs(strainChicken11919,cj1426c).
occurs(strainChicken11973,cj1426c).
occurs(strainChicken13713,cj1426c).
occurs(strainBeach1791,cj1426c).
occurs(strainClinical55320,cj1426c).
occurs(strainClinical55703,cj1426c).
occurs(strainClinical30280,cj1426c).
occurs(strainClinical30328,cj1426c).
occurs(strainChickenicken12450,cj1426c).
occurs(strainChickenicken12487,cj1426c).
occurs(strainChickenicken13082,cj1426c).
occurs(strainCjejuniRM1221,cj1426c).
occurs(strainChickenicken13411,cj1427c).
occurs(strainClinical44811,cj1427c).
occurs(strainClinical48612,cj1427c).
occurs(strainChicken11848,cj1427c).
occurs(strainChicken12567,cj1427c).
occurs(strainChicken11919,cj1427c).
occurs(strainChicken11973,cj1427c).
occurs(strainChicken13713,cj1427c).

occurs(strainBeach1791,cj1427c).
occurs(strainClinical43205,cj1427c).
occurs(strainClinical33106,cj1427c).
occurs(strainClinical55703,cj1427c).
occurs(strainClinical30280,cj1427c).
occurs(strainClinical30328,cj1427c).
occurs(strainClinical43983,cj1427c).
occurs(strainClinical40671,cj1427c).
occurs(strainOvine12241,cj1427c).
occurs(strainOvine12481,cj1427c).
occurs(strainBovinevineine13305,cj1427c).
occurs(strainChickenicken12450,cj1427c).
occurs(strainChickenicken12487,cj1427c).
occurs(strainChickenicken13082,cj1427c).
occurs(strainClinical33084,cj1427c).
occurs(strainChickenicken13411,cj1428c).
occurs(strainClinical44811,cj1428c).
occurs(strainClinical48612,cj1428c).
occurs(strainChicken11848,cj1428c).
occurs(strainChicken12567,cj1428c).
occurs(strainChicken11919,cj1428c).
occurs(strainChicken11973,cj1428c).
occurs(strainChicken13713,cj1428c).
occurs(strainBeach1791,cj1428c).
occurs(strainClinical55703,cj1428c).
occurs(strainClinical30280,cj1428c).
occurs(strainClinical30328,cj1428c).
occurs(strainChickenicken12450,cj1428c).
occurs(strainChickenicken12487,cj1428c).
occurs(strainChickenicken13082,cj1428c).
occurs(strainBeach1793,cj1428c).
occurs(strainChickenicken13411,cj1429c).
occurs(strainClinical44811,cj1429c).
occurs(strainClinical48612,cj1429c).
occurs(strainChicken11848,cj1429c).
occurs(strainChicken12567,cj1429c).
occurs(strainChicken11919,cj1429c).
occurs(strainChicken11973,cj1429c).
occurs(strainChicken13713,cj1429c).
occurs(strainBeach1791,cj1429c).
occurs(strainClinical55703,cj1429c).
occurs(strainClinical30280,cj1429c).
occurs(strainClinical30328,cj1429c).
occurs(strainChickenicken12450,cj1429c).
occurs(strainChickenicken12487,cj1429c).
occurs(strainChickenicken13082,cj1429c).
occurs(strainBeach1793,cj1429c).
occurs(strainClinical38857,cj1429c).
occurs(strainChickenicken13411,cj1430c).
occurs(strainClinical44811,cj1430c).
occurs(strainClinical48612,cj1430c).
occurs(strainChicken11848,cj1430c).
occurs(strainChicken12567,cj1430c).
occurs(strainChicken11919,cj1430c).

occurs(strainChicken11973,cj1430c).
occurs(strainChicken13713,cj1430c).
occurs(strainBeach1791,cj1430c).
occurs(strainClinical55320,cj1430c).
occurs(strainClinical55703,cj1430c).
occurs(strainClinical30280,cj1430c).
occurs(strainClinical30328,cj1430c).
occurs(strainChickenicken12450,cj1430c).
occurs(strainChickenicken12487,cj1430c).
occurs(strainChickenicken13082,cj1430c).
occurs(strainBeach1793,cj1430c).
occurs(strainChickenicken13411,cj1431c).
occurs(strainClinical44811,cj1431c).
occurs(strainClinical48612,cj1431c).
occurs(strainChicken11848,cj1431c).
occurs(strainChicken12567,cj1431c).
occurs(strainChicken11919,cj1431c).
occurs(strainChicken11973,cj1431c).
occurs(strainChicken13713,cj1431c).
occurs(strainBeach1791,cj1431c).
occurs(strainClinical55703,cj1431c).
occurs(strainClinical30280,cj1431c).
occurs(strainClinical30328,cj1431c).
occurs(strainChickenicken12450,cj1431c).
occurs(strainChickenicken12487,cj1431c).
occurs(strainChickenicken13082,cj1431c).
occurs(strainChicken40209,cj1431c).
occurs(strainChickenicken13411,cj1432c).
occurs(strainClinical44811,cj1432c).
occurs(strainClinical48612,cj1432c).
occurs(strainChicken11848,cj1432c).
occurs(strainChicken12567,cj1432c).
occurs(strainChicken11919,cj1432c).
occurs(strainChicken11973,cj1432c).
occurs(strainChicken13713,cj1432c).
occurs(strainBeach1791,cj1432c).
occurs(strainClinical55320,cj1432c).
occurs(strainClinical55703,cj1432c).
occurs(strainClinical30280,cj1432c).
occurs(strainClinical30328,cj1432c).
occurs(strainChickenicken12450,cj1432c).
occurs(strainChickenicken12487,cj1432c).
occurs(strainChickenicken13082,cj1432c).
occurs(strainChickenicken13411,cj1433c).
occurs(strainClinical44811,cj1433c).
occurs(strainClinical48612,cj1433c).
occurs(strainChicken11848,cj1433c).
occurs(strainChicken12567,cj1433c).
occurs(strainChicken11919,cj1433c).
occurs(strainChicken11973,cj1433c).
occurs(strainChicken13713,cj1433c).
occurs(strainBeach1791,cj1433c).
occurs(strainClinical55320,cj1433c).
occurs(strainClinical55703,cj1433c).

occurs(strainClinical30280,cj1433c).
occurs(strainClinical30328,cj1433c).
occurs(strainClinical43983,cj1433c).
occurs(strainChickenicken12450,cj1433c).
occurs(strainChickenicken12487,cj1433c).
occurs(strainChickenicken13082,cj1433c).
occurs(strainChickenicken13411,cj1434c).
occurs(strainClinical44811,cj1434c).
occurs(strainClinical48612,cj1434c).
occurs(strainChicken11848,cj1434c).
occurs(strainChicken11919,cj1434c).
occurs(strainChicken11973,cj1434c).
occurs(strainChicken13713,cj1434c).
occurs(strainBeach1791,cj1434c).
occurs(strainClinical55703,cj1434c).
occurs(strainClinical30280,cj1434c).
occurs(strainClinical30328,cj1434c).
occurs(strainChickenicken12450,cj1434c).
occurs(strainChickenicken12487,cj1434c).
occurs(strainChickenicken13082,cj1434c).
occurs(strainChickenicken13411,cj1435c).
occurs(strainClinical44811,cj1435c).
occurs(strainClinical48612,cj1435c).
occurs(strainChicken11848,cj1435c).
occurs(strainChicken12567,cj1435c).
occurs(strainChicken11919,cj1435c).
occurs(strainChicken11973,cj1435c).
occurs(strainChicken13713,cj1435c).
occurs(strainBeach1791,cj1435c).
occurs(strainClinical55703,cj1435c).
occurs(strainClinical30280,cj1435c).
occurs(strainClinical30328,cj1435c).
occurs(strainClinical43983,cj1435c).
occurs(strainChickenicken12450,cj1435c).
occurs(strainChickenicken12487,cj1435c).
occurs(strainChickenicken13082,cj1435c).
occurs(strainClinical36439,cj1435c).
occurs(strainChickenicken13411,cj1436c).
occurs(strainClinical44811,cj1436c).
occurs(strainClinical48612,cj1436c).
occurs(strainChicken11848,cj1436c).
occurs(strainChicken12567,cj1436c).
occurs(strainChicken11919,cj1436c).
occurs(strainChicken11973,cj1436c).
occurs(strainChicken13713,cj1436c).
occurs(strainBeach1791,cj1436c).
occurs(strainClinical55320,cj1436c).
occurs(strainClinical55703,cj1436c).
occurs(strainClinical30280,cj1436c).
occurs(strainClinical30328,cj1436c).
occurs(strainChickenicken12450,cj1436c).
occurs(strainChickenicken12487,cj1436c).
occurs(strainChickenicken13082,cj1436c).
occurs(strainChickenicken13411,cj1437c).

occurs(strainClinical44811,cj1437c).
occurs(strainClinical48612,cj1437c).
occurs(strainChicken11848,cj1437c).
occurs(strainChicken12567,cj1437c).
occurs(strainChicken11919,cj1437c).
occurs(strainChicken11973,cj1437c).
occurs(strainChicken13713,cj1437c).
occurs(strainBeach1791,cj1437c).
occurs(strainClinical55703,cj1437c).
occurs(strainClinical30280,cj1437c).
occurs(strainClinical30328,cj1437c).
occurs(strainChickenicken12450,cj1437c).
occurs(strainChickenicken12487,cj1437c).
occurs(strainChickenicken13082,cj1437c).
occurs(strainChickenicken13411,cj1438c).
occurs(strainClinical44811,cj1438c).
occurs(strainClinical48612,cj1438c).
occurs(strainChicken11848,cj1438c).
occurs(strainChicken12567,cj1438c).
occurs(strainChicken11919,cj1438c).
occurs(strainChicken11973,cj1438c).
occurs(strainChicken13713,cj1438c).
occurs(strainBeach1791,cj1438c).
occurs(strainClinical55703,cj1438c).
occurs(strainClinical30280,cj1438c).
occurs(strainClinical30328,cj1438c).
occurs(strainChickenicken12450,cj1438c).
occurs(strainChickenicken12487,cj1438c).
occurs(strainChickenicken13082,cj1438c).
occurs(strainChickenicken13411,cj1439c).
occurs(strainClinical44811,cj1439c).
occurs(strainClinical48612,cj1439c).
occurs(strainChicken11848,cj1439c).
occurs(strainChicken12567,cj1439c).
occurs(strainChicken11919,cj1439c).
occurs(strainChicken11973,cj1439c).
occurs(strainChicken13713,cj1439c).
occurs(strainBeach1791,cj1439c).
occurs(strainClinical55703,cj1439c).
occurs(strainClinical30280,cj1439c).
occurs(strainClinical30328,cj1439c).
occurs(strainClinical43983,cj1439c).
occurs(strainChickenicken12450,cj1439c).
occurs(strainChickenicken12487,cj1439c).
occurs(strainChickenicken13082,cj1439c).
occurs(strainClinical36439,cj1439c).
occurs(strainChickenicken13411,cj1440c).
occurs(strainClinical44811,cj1440c).
occurs(strainClinical48612,cj1440c).
occurs(strainChicken11848,cj1440c).
occurs(strainChicken12567,cj1440c).
occurs(strainChicken11919,cj1440c).
occurs(strainChicken11973,cj1440c).
occurs(strainChicken13713,cj1440c).

```
occurs(strainBeach1791,cj1440c).
occurs(strainClinical55320,cj1440c).
occurs(strainClinical55703,cj1440c).
occurs(strainClinical30280,cj1440c).
occurs(strainClinical30328,cj1440c).
occurs(strainClinical56281,cj1440c).
occurs(strainChickenicken12450,cj1440c).
occurs(strainChickenicken12487,cj1440c).
occurs(strainChickenicken13082,cj1440c).
occurs(strainChickenicken13411,cj1441c).
occurs(strainClinical44811,cj1441c).
occurs(strainClinical48612,cj1441c).
occurs(strainChicken11848,cj1441c).
occurs(strainChicken12567,cj1441c).
occurs(strainChicken11919,cj1441c).
occurs(strainChicken11973,cj1441c).
occurs(strainChicken13713,cj1441c).
occurs(strainBeach1791,cj1441c).
occurs(strainClinical55320,cj1441c).
occurs(strainClinical55703,cj1441c).
occurs(strainClinical30280,cj1441c).
occurs(strainClinical30328,cj1441c).
occurs(strainClinical56281,cj1441c).
occurs(strainChickenicken12450,cj1441c).
occurs(strainChickenicken12487,cj1441c).
occurs(strainChickenicken13082,cj1441c).
occurs(strainChickenicken13411,cj1442c).
occurs(strainClinical44811,cj1442c).
occurs(strainClinical48612,cj1442c).
occurs(strainClinical58473,cj1442c).
occurs(strainChicken11848,cj1442c).
occurs(strainChicken12567,cj1442c).
occurs(strainChicken11919,cj1442c).
occurs(strainChicken11973,cj1442c).
occurs(strainChicken13713,cj1442c).
occurs(strainBeach1791,cj1442c).
occurs(strainClinical43205,cj1442c).
occurs(strainClinical55703,cj1442c).
occurs(strainClinical30280,cj1442c).
occurs(strainClinical30328,cj1442c).
occurs(strainClinical41651,cj1442c).
occurs(strainClinical43983,cj1442c).
occurs(strainClinical40671,cj1442c).
occurs(strainClinical44958,cj1442c).
occurs(strainClinical52331,cj1442c).
occurs(strainClinical56281,cj1442c).
occurs(strainClinical56282,cj1442c).
occurs(strainOvine12241,cj1442c).
occurs(strainOvine12481,cj1442c).
occurs(strainBovinevineine13305,cj1442c).
occurs(strainChickenicken11818,cj1442c).
occurs(strainChicken12196,cj1442c).
occurs(strainChickenicken12450,cj1442c).
occurs(strainChickenicken12487,cj1442c).
```

occurs(strainChickenicken13082,cj1442c).
occurs(strainChickenicken13040,cj1442c).
occurs(strainChickenicken11856,cj1442c).
occurs(strainClinical56519,cj1442c).
occurs(strainChicken47693,cj1442c).
occurs(strainBeach1771,cj1442c).
occurs(strainClinical53250,cj1442c).
occurs(strainClinicalG1,cj1443c).
occurs(strainClinicalG3,cj1443c).
occurs(strainChickenicken11974,cj1443c).
occurs(strainChickenicken13249,cj1443c).
occurs(strainChickenicken13411,cj1443c).
occurs(strainClinical44811,cj1443c).
occurs(strainClinical48612,cj1443c).
occurs(strainClinical58473,cj1443c).
occurs(strainChicken11848,cj1443c).
occurs(strainChicken12567,cj1443c).
occurs(strainChicken11919,cj1443c).
occurs(strainChicken11973,cj1443c).
occurs(strainChicken13713,cj1443c).
occurs(strainBeach1791,cj1443c).
occurs(strainClinical43205,cj1443c).
occurs(strainClinical33106,cj1443c).
occurs(strainClinicall38556,cj1443c).
occurs(strainClinicall39182,cj1443c).
occurs(strainClinical44933,cj1443c).
occurs(strainClinical45631,cj1443c).
occurs(strainClinical55320,cj1443c).
occurs(strainClinical55703,cj1443c).
occurs(strainClinical30280,cj1443c).
occurs(strainClinical30328,cj1443c).
occurs(strainClinical41651,cj1443c).
occurs(strainClinical32799,cj1443c).
occurs(strainClinical43983,cj1443c).
occurs(strainClinical40671,cj1443c).
occurs(strainClinical44958,cj1443c).
occurs(strainClinical52331,cj1443c).
occurs(strainClinical56281,cj1443c).
occurs(strainClinical56282,cj1443c).
occurs(strainClinical56832,cj1443c).
occurs(strainOvine12241,cj1443c).
occurs(strainOvine12481,cj1443c).
occurs(strainBovinevineine13305,cj1443c).
occurs(strainChicken12196,cj1443c).
occurs(strainChickenicken12450,cj1443c).
occurs(strainChickenicken12487,cj1443c).
occurs(strainChickenicken13082,cj1443c).
occurs(strainChickenicken13040,cj1443c).
occurs(strainChickenicken11856,cj1443c).
occurs(strainClinical36069,cj1443c).
occurs(strainChicken40209,cj1443c).
occurs(strainBeach1793,cj1443c).
occurs(strainClinical81116,cj1443c).
occurs(strainClinical56519,cj1443c).

occurs(strainClinical44119,cj1443c).
occurs(strainClinical34007,cj1443c).
occurs(strainClinicalinical15168,cj1443c).
occurs(strainClinical18836,cj1443c).
occurs(strainClinicalM1,cj1443c).
occurs(strainClinical36860,cj1443c).
occurs(strainClinical40917,cj1443c).
occurs(strainClinical38857,cj1443c).
occurs(strainChicken47693,cj1443c).
occurs(strainClinical63326,cj1443c).
occurs(strainClinical64555,cj1443c).
occurs(strainClinical59364,cj1443c).
occurs(strainClinical59424,cj1443c).
occurs(strainClinical33084,cj1443c).
occurs(strainClinical31481,cj1443c).
occurs(strainClinical39828,cj1443c).
occurs(strainBeach1771,cj1443c).
occurs(strainClinical53250,cj1443c).
occurs(strainClinical45557,cj1443c).
occurs(strainClinical47939,cj1443c).
occurs(strainClinicalG1,cj1444c).
occurs(strainClinicalG3,cj1444c).
occurs(strainChickenicken11974,cj1444c).
occurs(strainChickenicken13249,cj1444c).
occurs(strainChickenicken13411,cj1444c).
occurs(strainClinical44811,cj1444c).
occurs(strainClinical48612,cj1444c).
occurs(strainClinical58473,cj1444c).
occurs(strainChicken11848,cj1444c).
occurs(strainChicken12567,cj1444c).
occurs(strainChicken11919,cj1444c).
occurs(strainChicken11973,cj1444c).
occurs(strainChicken13713,cj1444c).
occurs(strainBeach1791,cj1444c).
occurs(strainClinical43205,cj1444c).
occurs(strainClinical33106,cj1444c).
occurs(strainClinicall38556,cj1444c).
occurs(strainClinicall39182,cj1444c).
occurs(strainClinical44933,cj1444c).
occurs(strainClinical45631,cj1444c).
occurs(strainClinical55320,cj1444c).
occurs(strainClinical55703,cj1444c).
occurs(strainClinical30280,cj1444c).
occurs(strainClinical30328,cj1444c).
occurs(strainClinical41651,cj1444c).
occurs(strainClinical32799,cj1444c).
occurs(strainClinical43983,cj1444c).
occurs(strainClinical40671,cj1444c).
occurs(strainClinical44958,cj1444c).
occurs(strainClinical52331,cj1444c).
occurs(strainClinical56281,cj1444c).
occurs(strainClinical56282,cj1444c).
occurs(strainClinical56832,cj1444c).
occurs(strainOvine12241,cj1444c).

occurs(strainOvine12481,cj1444c).
occurs(strainBovinevineine13305,cj1444c).
occurs(strainChickenicken12912,cj1444c).
occurs(strainChickenicken11818,cj1444c).
occurs(strainChicken12196,cj1444c).
occurs(strainChickenicken12450,cj1444c).
occurs(strainChickenicken12487,cj1444c).
occurs(strainChickenicken13082,cj1444c).
occurs(strainChickenicken13040,cj1444c).
occurs(strainChickenicken11856,cj1444c).
occurs(strainClinical36069,cj1444c).
occurs(strainChicken40209,cj1444c).
occurs(strainBeach1793,cj1444c).
occurs(strainClinical81116,cj1444c).
occurs(strainClinical36439,cj1444c).
occurs(strainClinical56519,cj1444c).
occurs(strainClinical32787,cj1444c).
occurs(strainClinicall31467,cj1444c).
occurs(strainClinical44119,cj1444c).
occurs(strainClinical34007,cj1444c).
occurs(strainClinical38762,cj1444c).
occurs(strainClinicalinical15168,cj1444c).
occurs(strainClinical18836,cj1444c).
occurs(strainClinicalM1,cj1444c).
occurs(strainClinical36860,cj1444c).
occurs(strainClinical40917,cj1444c).
occurs(strainClinical38857,cj1444c).
occurs(strainCjejuniRM1221,cj1444c).
occurs(strainChicken47693,cj1444c).
occurs(strainClinical63326,cj1444c).
occurs(strainClinical64555,cj1444c).
occurs(strainClinical59364,cj1444c).
occurs(strainClinical59424,cj1444c).
occurs(strainClinical33084,cj1444c).
occurs(strainClinical31481,cj1444c).
occurs(strainClinical39828,cj1444c).
occurs(strainBeach1771,cj1444c).
occurs(strainClinical53250,cj1444c).
occurs(strainClinical45557,cj1444c).
occurs(strainClinical47939,cj1444c).
occurs(strainClinicalG1,cj1445c).
occurs(strainChickenicken11974,cj1445c).
occurs(strainChickenicken13249,cj1445c).
occurs(strainChickenicken13411,cj1445c).
occurs(strainClinical44811,cj1445c).
occurs(strainClinical48612,cj1445c).
occurs(strainClinical58473,cj1445c).
occurs(strainChicken11848,cj1445c).
occurs(strainChicken12567,cj1445c).
occurs(strainChicken11919,cj1445c).
occurs(strainChicken11973,cj1445c).
occurs(strainChicken13713,cj1445c).
occurs(strainBeach1791,cj1445c).
occurs(strainClinical43205,cj1445c).

occurs(strainClinical33106,cj1445c).
occurs(strainClinicall38556,cj1445c).
occurs(strainClinicall39182,cj1445c).
occurs(strainClinical44933,cj1445c).
occurs(strainClinical45631,cj1445c).
occurs(strainClinical55320,cj1445c).
occurs(strainClinical55703,cj1445c).
occurs(strainClinical30280,cj1445c).
occurs(strainClinical30328,cj1445c).
occurs(strainClinical41651,cj1445c).
occurs(strainClinical43983,cj1445c).
occurs(strainClinical40671,cj1445c).
occurs(strainClinical44958,cj1445c).
occurs(strainClinical52331,cj1445c).
occurs(strainClinical56281,cj1445c).
occurs(strainClinical56282,cj1445c).
occurs(strainClinical56832,cj1445c).
occurs(strainOvine12241,cj1445c).
occurs(strainOvine12481,cj1445c).
occurs(strainBovinevineine13305,cj1445c).
occurs(strainChickenicken12912,cj1445c).
occurs(strainChicken12196,cj1445c).
occurs(strainChickenicken12450,cj1445c).
occurs(strainChickenicken12487,cj1445c).
occurs(strainChickenicken13082,cj1445c).
occurs(strainChickenicken13040,cj1445c).
occurs(strainChickenicken11856,cj1445c).
occurs(strainClinical36069,cj1445c).
occurs(strainChicken40209,cj1445c).
occurs(strainBeach1793,cj1445c).
occurs(strainClinical81116,cj1445c).
occurs(strainClinical36439,cj1445c).
occurs(strainClinical56519,cj1445c).
occurs(strainClinical32787,cj1445c).
occurs(strainClinicall31467,cj1445c).
occurs(strainClinical44119,cj1445c).
occurs(strainClinical34007,cj1445c).
occurs(strainClinical38762,cj1445c).
occurs(strainClinicalinical15168,cj1445c).
occurs(strainClinical18836,cj1445c).
occurs(strainClinicalM1,cj1445c).
occurs(strainClinical36860,cj1445c).
occurs(strainClinical40917,cj1445c).
occurs(strainClinical38857,cj1445c).
occurs(strainCjejuniRM1221,cj1445c).
occurs(strainChicken47693,cj1445c).
occurs(strainClinical63326,cj1445c).
occurs(strainClinical64555,cj1445c).
occurs(strainClinical59364,cj1445c).
occurs(strainClinical59424,cj1445c).
occurs(strainClinical33084,cj1445c).
occurs(strainClinical31481,cj1445c).
occurs(strainBeach1771,cj1445c).
occurs(strainClinical53250,cj1445c).

```
occurs(strainClinical45557,cj1445c).
occurs(strainClinical47939,cj1445c).
occurs(strainChickenicken13411,cj1447c).
occurs(strainClinical44811,cj1447c).
occurs(strainClinical48612,cj1447c).
occurs(strainClinical58473,cj1447c).
occurs(strainChicken11848,cj1447c).
occurs(strainChicken12567,cj1447c).
occurs(strainChicken11919,cj1447c).
occurs(strainChicken11973,cj1447c).
occurs(strainChicken13713,cj1447c).
occurs(strainBeach1791,cj1447c).
occurs(strainClinical43205,cj1447c).
occurs(strainClinical55703,cj1447c).
occurs(strainClinical30280,cj1447c).
occurs(strainClinical30328,cj1447c).
occurs(strainChickenicken12912,cj1447c).
occurs(strainChickenicken12450,cj1447c).
occurs(strainChickenicken12487,cj1447c).
occurs(strainChickenicken13082,cj1447c).
occurs(strainChicken40209,cj1447c).
occurs(strainClinical81116,cj1447c).
occurs(strainCjejuniRM1221,cj1447c).
occurs(strainChickenicken13411,cj1448c).
occurs(strainClinical44811,cj1448c).
occurs(strainClinical48612,cj1448c).
occurs(strainClinical58473,cj1448c).
occurs(strainChicken11848,cj1448c).
occurs(strainChicken12567,cj1448c).
occurs(strainChicken11919,cj1448c).
occurs(strainChicken11973,cj1448c).
occurs(strainChicken13713,cj1448c).
occurs(strainBeach1791,cj1448c).
occurs(strainClinical43205,cj1448c).
occurs(strainClinical55320,cj1448c).
occurs(strainClinical55703,cj1448c).
occurs(strainClinical30280,cj1448c).
occurs(strainClinical30328,cj1448c).
occurs(strainChickenicken12912,cj1448c).
occurs(strainChickenicken12450,cj1448c).
occurs(strainChickenicken12487,cj1448c).
occurs(strainChickenicken13082,cj1448c).
occurs(strainChicken40209,cj1448c).
occurs(strainBeach1793,cj1448c).
occurs(strainClinical81116,cj1448c).
occurs(strainClinical36860,cj1448c).
occurs(strainClinical53250,cj1448c).
occurs0(strainClinicalG1,cj1413c).
occurs0(strainClinicalG3,cj1413c).
occurs0(strainChickenicken11974,cj1413c).
occurs0(strainChickenicken13249,cj1413c).
occurs0(strainChickenicken13411,cj1413c).
occurs0(strainClinical44811,cj1413c).
occurs0(strainClinical48612,cj1413c).
```

occurs0(strainClinical58473,cj1413c).
occurs0(strainChicken11848,cj1413c).
occurs0(strainChicken12567,cj1413c).
occurs0(strainChicken11919,cj1413c).
occurs0(strainChicken11973,cj1413c).
occurs0(strainChicken13713,cj1413c).
occurs0(strainBeach1791,cj1413c).
occurs0(strainClinical43205,cj1413c).
occurs0(strainClinical33106,cj1413c).
occurs0(strainClinicall38556,cj1413c).
occurs0(strainClinicall39182,cj1413c).
occurs0(strainClinical44933,cj1413c).
occurs0(strainClinical45631,cj1413c).
occurs0(strainClinical55320,cj1413c).
occurs0(strainClinical55703,cj1413c).
occurs0(strainClinical30280,cj1413c).
occurs0(strainClinical30328,cj1413c).
occurs0(strainClinical41651,cj1413c).
occurs0(strainClinical32799,cj1413c).
occurs0(strainClinical43983,cj1413c).
occurs0(strainClinical40671,cj1413c).
occurs0(strainClinical44958,cj1413c).
occurs0(strainClinical52331,cj1413c).
occurs0(strainClinical56281,cj1413c).
occurs0(strainClinical56282,cj1413c).
occurs0(strainClinical56832,cj1413c).
occurs0(strainOvine12241,cj1413c).
occurs0(strainOvine12481,cj1413c).
occurs0(strainBovinevineine13305,cj1413c).
occurs0(strainChickenicken12912,cj1413c).
occurs0(strainChickenicken11818,cj1413c).
occurs0(strainChicken12196,cj1413c).
occurs0(strainChickenicken12450,cj1413c).
occurs0(strainChickenicken12487,cj1413c).
occurs0(strainChickenicken13082,cj1413c).
occurs0(strainChickenicken13040,cj1413c).
occurs0(strainChickenicken11856,cj1413c).
occurs0(strainClinical36069,cj1413c).
occurs0(strainChicken40209,cj1413c).
occurs0(strainBeach1793,cj1413c).
occurs0(strainClinical81116,cj1413c).
occurs0(strainClinical36439,cj1413c).
occurs0(strainClinical56519,cj1413c).
occurs0(strainClinical32787,cj1413c).
occurs0(strainClinicall31467,cj1413c).
occurs0(strainClinical44119,cj1413c).
occurs0(strainClinical34007,cj1413c).
occurs0(strainClinical38762,cj1413c).
occurs0(strainClinicalinical15168,cj1413c).
occurs0(strainClinical18836,cj1413c).
occurs0(strainClinicalM1,cj1413c).
occurs0(strainClinical36860,cj1413c).
occurs0(strainClinical40917,cj1413c).
occurs0(strainClinical38857,cj1413c).

occurs0(strainCjejuniRM1221,cj1413c).
occurs0(strainChicken47693,cj1413c).
occurs0(strainClinical63326,cj1413c).
occurs0(strainClinical64555,cj1413c).
occurs0(strainClinical59364,cj1413c).
occurs0(strainClinical59424,cj1413c).
occurs0(strainClinical33084,cj1413c).
occurs0(strainClinical31481,cj1413c).
occurs0(strainClinical39828,cj1413c).
occurs0(strainBeach1771,cj1413c).
occurs0(strainClinical53250,cj1413c).
occurs0(strainClinical45557,cj1413c).
occurs0(strainClinical47939,cj1413c).
occurs0(strainClinicalG1,cj1414c).
occurs0(strainChickenicken11974,cj1414c).
occurs0(strainChickenicken13249,cj1414c).
occurs0(strainChickenicken13411,cj1414c).
occurs0(strainClinical44811,cj1414c).
occurs0(strainClinical48612,cj1414c).
occurs0(strainClinical58473,cj1414c).
occurs0(strainChicken11848,cj1414c).
occurs0(strainChicken12567,cj1414c).
occurs0(strainChicken11919,cj1414c).
occurs0(strainChicken11973,cj1414c).
occurs0(strainChicken13713,cj1414c).
occurs0(strainBeach1791,cj1414c).
occurs0(strainClinical43205,cj1414c).
occurs0(strainClinical33106,cj1414c).
occurs0(strainClinicall38556,cj1414c).
occurs0(strainClinicall39182,cj1414c).
occurs0(strainClinical44933,cj1414c).
occurs0(strainClinical45631,cj1414c).
occurs0(strainClinical55320,cj1414c).
occurs0(strainClinical55703,cj1414c).
occurs0(strainClinical30280,cj1414c).
occurs0(strainClinical30328,cj1414c).
occurs0(strainClinical41651,cj1414c).
occurs0(strainClinical40671,cj1414c).
occurs0(strainClinical44958,cj1414c).
occurs0(strainClinical52331,cj1414c).
occurs0(strainClinical56281,cj1414c).
occurs0(strainClinical56282,cj1414c).
occurs0(strainClinical56832,cj1414c).
occurs0(strainOvine12241,cj1414c).
occurs0(strainOvine12481,cj1414c).
occurs0(strainChickenicken12912,cj1414c).
occurs0(strainChicken12196,cj1414c).
occurs0(strainChickenicken12450,cj1414c).
occurs0(strainChickenicken12487,cj1414c).
occurs0(strainChickenicken13082,cj1414c).
occurs0(strainChickenicken13040,cj1414c).
occurs0(strainChickenicken11856,cj1414c).
occurs0(strainClinical36069,cj1414c).
occurs0(strainChicken40209,cj1414c).

occurs0(strainBeach1793,cj1414c).
occurs0(strainClinical56519,cj1414c).
occurs0(strainClinical44119,cj1414c).
occurs0(strainClinical34007,cj1414c).
occurs0(strainClinicalinical15168,cj1414c).
occurs0(strainClinical18836,cj1414c).
occurs0(strainClinical38857,cj1414c).
occurs0(strainCjejuniRM1221,cj1414c).
occurs0(strainChicken47693,cj1414c).
occurs0(strainClinical63326,cj1414c).
occurs0(strainClinical64555,cj1414c).
occurs0(strainClinical59364,cj1414c).
occurs0(strainClinical59424,cj1414c).
occurs0(strainClinical33084,cj1414c).
occurs0(strainClinical31481,cj1414c).
occurs0(strainClinical39828,cj1414c).
occurs0(strainBeach1771,cj1414c).
occurs0(strainClinical53250,cj1414c).
occurs0(strainClinical45557,cj1414c).
occurs0(strainClinical47939,cj1414c).
occurs0(strainClinicalG1,cj1415c).
occurs0(strainClinicalG3,cj1415c).
occurs0(strainChickenicken11974,cj1415c).
occurs0(strainChickenicken13249,cj1415c).
occurs0(strainChickenicken13411,cj1415c).
occurs0(strainClinical44811,cj1415c).
occurs0(strainClinical48612,cj1415c).
occurs0(strainClinical58473,cj1415c).
occurs0(strainChicken11848,cj1415c).
occurs0(strainChicken12567,cj1415c).
occurs0(strainChicken11919,cj1415c).
occurs0(strainChicken11973,cj1415c).
occurs0(strainChicken13713,cj1415c).
occurs0(strainBeach1791,cj1415c).
occurs0(strainClinical43205,cj1415c).
occurs0(strainClinical33106,cj1415c).
occurs0(strainClinical55703,cj1415c).
occurs0(strainClinical30280,cj1415c).
occurs0(strainClinical30328,cj1415c).
occurs0(strainClinical41651,cj1415c).
occurs0(strainClinical32799,cj1415c).
occurs0(strainClinical43983,cj1415c).
occurs0(strainClinical40671,cj1415c).
occurs0(strainClinical44958,cj1415c).
occurs0(strainClinical52331,cj1415c).
occurs0(strainClinical56281,cj1415c).
occurs0(strainClinical56282,cj1415c).
occurs0(strainClinical56832,cj1415c).
occurs0(strainOvine12241,cj1415c).
occurs0(strainOvine12481,cj1415c).
occurs0(strainBovinevineine13305,cj1415c).
occurs0(strainChickenicken12912,cj1415c).
occurs0(strainChickenicken11818,cj1415c).
occurs0(strainChicken12196,cj1415c).

occurs0(strainChickenicken12450,cj1415c).
occurs0(strainChickenicken12487,cj1415c).
occurs0(strainChickenicken13082,cj1415c).
occurs0(strainChickenicken13040,cj1415c).
occurs0(strainChickenicken11856,cj1415c).
occurs0(strainChicken40209,cj1415c).
occurs0(strainBeach1793,cj1415c).
occurs0(strainClinical32787,cj1415c).
occurs0(strainClinicall31467,cj1415c).
occurs0(strainClinical38762,cj1415c).
occurs0(strainClinicalinical15168,cj1415c).
occurs0(strainClinical18836,cj1415c).
occurs0(strainClinical38857,cj1415c).
occurs0(strainCjejuniRM1221,cj1415c).
occurs0(strainClinical33084,cj1415c).
occurs0(strainClinical31481,cj1415c).
occurs0(strainClinical39828,cj1415c).
occurs0(strainClinical45557,cj1415c).
occurs0(strainClinicalG1,cj1416c).
occurs0(strainClinicalG3,cj1416c).
occurs0(strainChickenicken11974,cj1416c).
occurs0(strainChickenicken13249,cj1416c).
occurs0(strainChickenicken13411,cj1416c).
occurs0(strainClinical44811,cj1416c).
occurs0(strainClinical48612,cj1416c).
occurs0(strainClinical58473,cj1416c).
occurs0(strainChicken11848,cj1416c).
occurs0(strainChicken12567,cj1416c).
occurs0(strainChicken11919,cj1416c).
occurs0(strainChicken11973,cj1416c).
occurs0(strainChicken13713,cj1416c).
occurs0(strainBeach1791,cj1416c).
occurs0(strainClinical43205,cj1416c).
occurs0(strainClinical33106,cj1416c).
occurs0(strainClinical55320,cj1416c).
occurs0(strainClinical55703,cj1416c).
occurs0(strainClinical30280,cj1416c).
occurs0(strainClinical30328,cj1416c).
occurs0(strainClinical41651,cj1416c).
occurs0(strainClinical32799,cj1416c).
occurs0(strainClinical43983,cj1416c).
occurs0(strainClinical40671,cj1416c).
occurs0(strainClinical44958,cj1416c).
occurs0(strainClinical52331,cj1416c).
occurs0(strainClinical56281,cj1416c).
occurs0(strainClinical56282,cj1416c).
occurs0(strainClinical56832,cj1416c).
occurs0(strainOvine12241,cj1416c).
occurs0(strainOvine12481,cj1416c).
occurs0(strainBovinevineine13305,cj1416c).
occurs0(strainChickenicken12912,cj1416c).
occurs0(strainChickenicken11818,cj1416c).
occurs0(strainChicken12196,cj1416c).
occurs0(strainChickenicken12450,cj1416c).

```
occurs0(strainChickenicken12487,cj1416c).
occurs0(strainChickenicken13082,cj1416c).
occurs0(strainChickenicken13040,cj1416c).
occurs0(strainChickenicken11856,cj1416c).
occurs0(strainChicken40209,cj1416c).
occurs0(strainBeach1793,cj1416c).
occurs0(strainClinical81116,cj1416c).
occurs0(strainClinical56519,cj1416c).
occurs0(strainClinical32787,cj1416c).
occurs0(strainClinicall31467,cj1416c).
occurs0(strainClinical38762,cj1416c).
occurs0(strainClinicalinical15168,cj1416c).
occurs0(strainClinical18836,cj1416c).
occurs0(strainClinicalM1,cj1416c).
occurs0(strainClinical36860,cj1416c).
occurs0(strainClinical40917,cj1416c).
occurs0(strainClinical38857,cj1416c).
occurs0(strainChicken47693,cj1416c).
occurs0(strainClinical33084,cj1416c).
occurs0(strainClinical31481,cj1416c).
occurs0(strainClinical39828,cj1416c).
occurs0(strainClinical53250,cj1416c).
occurs0(strainClinical45557,cj1416c).
occurs0(strainClinicalG1,cj1417c).
occurs0(strainClinicalG3,cj1417c).
occurs0(strainChickenicken11974,cj1417c).
occurs0(strainChickenicken13249,cj1417c).
occurs0(strainChickenicken13411,cj1417c).
occurs0(strainClinical44811,cj1417c).
occurs0(strainClinical48612,cj1417c).
occurs0(strainClinical58473,cj1417c).
occurs0(strainChicken11848,cj1417c).
occurs0(strainChicken12567,cj1417c).
occurs0(strainChicken11919,cj1417c).
occurs0(strainChicken11973,cj1417c).
occurs0(strainChicken13713,cj1417c).
occurs0(strainBeach1791,cj1417c).
occurs0(strainClinical43205,cj1417c).
occurs0(strainClinical33106,cj1417c).
occurs0(strainClinical55703,cj1417c).
occurs0(strainClinical30280,cj1417c).
occurs0(strainClinical30328,cj1417c).
occurs0(strainClinical41651,cj1417c).
occurs0(strainClinical32799,cj1417c).
occurs0(strainClinical43983,cj1417c).
occurs0(strainClinical40671,cj1417c).
occurs0(strainClinical44958,cj1417c).
occurs0(strainClinical52331,cj1417c).
occurs0(strainClinical56281,cj1417c).
occurs0(strainClinical56282,cj1417c).
occurs0(strainClinical56832,cj1417c).
occurs0(strainOvine12241,cj1417c).
occurs0(strainOvine12481,cj1417c).
occurs0(strainBovinevineine13305,cj1417c).
```

occurs0(strainChickenicken12912,cj1417c).
occurs0(strainChickenicken11818,cj1417c).
occurs0(strainChicken12196,cj1417c).
occurs0(strainChickenicken12450,cj1417c).
occurs0(strainChickenicken12487,cj1417c).
occurs0(strainChickenicken13082,cj1417c).
occurs0(strainChickenicken13040,cj1417c).
occurs0(strainChickenicken11856,cj1417c).
occurs0(strainChicken40209,cj1417c).
occurs0(strainBeach1793,cj1417c).
occurs0(strainClinical81116,cj1417c).
occurs0(strainClinical56519,cj1417c).
occurs0(strainClinical32787,cj1417c).
occurs0(strainClinicall31467,cj1417c).
occurs0(strainClinical38762,cj1417c).
occurs0(strainClinicalinical15168,cj1417c).
occurs0(strainClinical18836,cj1417c).
occurs0(strainClinicalM1,cj1417c).
occurs0(strainClinical36860,cj1417c).
occurs0(strainClinical40917,cj1417c).
occurs0(strainClinical38857,cj1417c).
occurs0(strainChicken47693,cj1417c).
occurs0(strainClinical33084,cj1417c).
occurs0(strainClinical31481,cj1417c).
occurs0(strainClinical39828,cj1417c).
occurs0(strainClinical53250,cj1417c).
occurs0(strainClinical45557,cj1417c).
occurs0(strainClinicalG1,cj1418c).
occurs0(strainClinicalG3,cj1418c).
occurs0(strainChickenicken11974,cj1418c).
occurs0(strainChickenicken13249,cj1418c).
occurs0(strainChickenicken13411,cj1418c).
occurs0(strainClinical44811,cj1418c).
occurs0(strainClinical48612,cj1418c).
occurs0(strainClinical58473,cj1418c).
occurs0(strainChicken11848,cj1418c).
occurs0(strainChicken12567,cj1418c).
occurs0(strainChicken11919,cj1418c).
occurs0(strainChicken11973,cj1418c).
occurs0(strainChicken13713,cj1418c).
occurs0(strainBeach1791,cj1418c).
occurs0(strainClinical43205,cj1418c).
occurs0(strainClinical33106,cj1418c).
occurs0(strainClinical55703,cj1418c).
occurs0(strainClinical30280,cj1418c).
occurs0(strainClinical30328,cj1418c).
occurs0(strainClinical41651,cj1418c).
occurs0(strainClinical32799,cj1418c).
occurs0(strainClinical43983,cj1418c).
occurs0(strainClinical40671,cj1418c).
occurs0(strainClinical44958,cj1418c).
occurs0(strainClinical52331,cj1418c).
occurs0(strainClinical56281,cj1418c).
occurs0(strainClinical56282,cj1418c).

occurs0(strainClinical56832,cj1418c).
occurs0(strainOvine12241,cj1418c).
occurs0(strainOvine12481,cj1418c).
occurs0(strainBovinevineine13305,cj1418c).
occurs0(strainChickenicken12912,cj1418c).
occurs0(strainChickenicken11818,cj1418c).
occurs0(strainChicken12196,cj1418c).
occurs0(strainChickenicken12450,cj1418c).
occurs0(strainChickenicken12487,cj1418c).
occurs0(strainChickenicken13082,cj1418c).
occurs0(strainChickenicken13040,cj1418c).
occurs0(strainChickenicken11856,cj1418c).
occurs0(strainChicken40209,cj1418c).
occurs0(strainBeach1793,cj1418c).
occurs0(strainClinical81116,cj1418c).
occurs0(strainClinical56519,cj1418c).
occurs0(strainClinical32787,cj1418c).
occurs0(strainClinicall31467,cj1418c).
occurs0(strainClinical38762,cj1418c).
occurs0(strainClinicalinical15168,cj1418c).
occurs0(strainClinical18836,cj1418c).
occurs0(strainClinicalM1,cj1418c).
occurs0(strainClinical36860,cj1418c).
occurs0(strainClinical40917,cj1418c).
occurs0(strainClinical38857,cj1418c).
occurs0(strainChicken47693,cj1418c).
occurs0(strainClinical33084,cj1418c).
occurs0(strainClinical31481,cj1418c).
occurs0(strainClinical39828,cj1418c).
occurs0(strainClinical53250,cj1418c).
occurs0(strainClinical45557,cj1418c).
occurs0(strainClinicalG1,cj1419c).
occurs0(strainClinicalG3,cj1419c).
occurs0(strainChickenicken11974,cj1419c).
occurs0(strainChickenicken13249,cj1419c).
occurs0(strainChickenicken13411,cj1419c).
occurs0(strainClinical44811,cj1419c).
occurs0(strainClinical48612,cj1419c).
occurs0(strainClinical58473,cj1419c).
occurs0(strainChicken11848,cj1419c).
occurs0(strainChicken12567,cj1419c).
occurs0(strainChicken11919,cj1419c).
occurs0(strainChicken11973,cj1419c).
occurs0(strainChicken13713,cj1419c).
occurs0(strainBeach1791,cj1419c).
occurs0(strainClinical43205,cj1419c).
occurs0(strainClinical33106,cj1419c).
occurs0(strainClinical55703,cj1419c).
occurs0(strainClinical30280,cj1419c).
occurs0(strainClinical30328,cj1419c).
occurs0(strainClinical41651,cj1419c).
occurs0(strainClinical32799,cj1419c).
occurs0(strainClinical43983,cj1419c).
occurs0(strainClinical40671,cj1419c).

occurs0(strainClinical44958,cj1419c).
occurs0(strainClinical52331,cj1419c).
occurs0(strainClinical56281,cj1419c).
occurs0(strainClinical56282,cj1419c).
occurs0(strainClinical56832,cj1419c).
occurs0(strainOvine12241,cj1419c).
occurs0(strainOvine12481,cj1419c).
occurs0(strainBovinevineine13305,cj1419c).
occurs0(strainChickenicken12912,cj1419c).
occurs0(strainChickenicken11818,cj1419c).
occurs0(strainChicken12196,cj1419c).
occurs0(strainChickenicken12450,cj1419c).
occurs0(strainChickenicken12487,cj1419c).
occurs0(strainChickenicken13082,cj1419c).
occurs0(strainChickenicken13040,cj1419c).
occurs0(strainChickenicken11856,cj1419c).
occurs0(strainChicken40209,cj1419c).
occurs0(strainBeach1793,cj1419c).
occurs0(strainClinical81116,cj1419c).
occurs0(strainClinical56519,cj1419c).
occurs0(strainClinical32787,cj1419c).
occurs0(strainClinicall31467,cj1419c).
occurs0(strainClinical38762,cj1419c).
occurs0(strainClinicalinical15168,cj1419c).
occurs0(strainClinical18836,cj1419c).
occurs0(strainClinicalM1,cj1419c).
occurs0(strainClinical36860,cj1419c).
occurs0(strainClinical40917,cj1419c).
occurs0(strainClinical38857,cj1419c).
occurs0(strainChicken47693,cj1419c).
occurs0(strainClinical33084,cj1419c).
occurs0(strainClinical31481,cj1419c).
occurs0(strainClinical39828,cj1419c).
occurs0(strainClinical53250,cj1419c).
occurs0(strainClinical45557,cj1419c).
occurs0(strainChickenicken13411,cj1421c).
occurs0(strainClinical44811,cj1421c).
occurs0(strainClinical48612,cj1421c).
occurs0(strainChicken11848,cj1421c).
occurs0(strainChicken12567,cj1421c).
occurs0(strainChicken11919,cj1421c).
occurs0(strainChicken11973,cj1421c).
occurs0(strainChicken13713,cj1421c).
occurs0(strainBeach1791,cj1421c).
occurs0(strainClinical55703,cj1421c).
occurs0(strainClinical30280,cj1421c).
occurs0(strainClinical30328,cj1421c).
occurs0(strainChickenicken12450,cj1421c).
occurs0(strainChickenicken12487,cj1421c).
occurs0(strainChickenicken13082,cj1421c).
occurs0(strainCjejuniRM1221,cj1421c).
occurs0(strainChickenicken13411,cj1422c).
occurs0(strainClinical44811,cj1422c).
occurs0(strainClinical48612,cj1422c).

occurs0(strainChicken11848,cj1422c).
occurs0(strainChicken12567,cj1422c).
occurs0(strainChicken11919,cj1422c).
occurs0(strainChicken11973,cj1422c).
occurs0(strainChicken13713,cj1422c).
occurs0(strainBeach1791,cj1422c).
occurs0(strainClinical55320,cj1422c).
occurs0(strainClinical55703,cj1422c).
occurs0(strainClinical30280,cj1422c).
occurs0(strainClinical30328,cj1422c).
occurs0(strainChickenicken12450,cj1422c).
occurs0(strainChickenicken12487,cj1422c).
occurs0(strainChickenicken13082,cj1422c).
occurs0(strainChickenicken13411,cj1423c).
occurs0(strainClinical44811,cj1423c).
occurs0(strainClinical48612,cj1423c).
occurs0(strainChicken11848,cj1423c).
occurs0(strainChicken12567,cj1423c).
occurs0(strainChicken11919,cj1423c).
occurs0(strainChicken11973,cj1423c).
occurs0(strainChicken13713,cj1423c).
occurs0(strainBeach1791,cj1423c).
occurs0(strainClinical43205,cj1423c).
occurs0(strainClinical55320,cj1423c).
occurs0(strainClinical55703,cj1423c).
occurs0(strainClinical30280,cj1423c).
occurs0(strainClinical30328,cj1423c).
occurs0(strainClinical41651,cj1423c).
occurs0(strainClinical44958,cj1423c).
occurs0(strainClinical56281,cj1423c).
occurs0(strainClinical56282,cj1423c).
occurs0(strainChickenicken12450,cj1423c).
occurs0(strainChickenicken12487,cj1423c).
occurs0(strainChickenicken13082,cj1423c).
occurs0(strainBeach1793,cj1423c).
occurs0(strainClinical33084,cj1423c).
occurs0(strainClinical53250,cj1423c).
occurs0(strainChickenicken13411,cj1424c).
occurs0(strainClinical44811,cj1424c).
occurs0(strainClinical48612,cj1424c).
occurs0(strainChicken11848,cj1424c).
occurs0(strainChicken12567,cj1424c).
occurs0(strainChicken11919,cj1424c).
occurs0(strainChicken11973,cj1424c).
occurs0(strainChicken13713,cj1424c).
occurs0(strainBeach1791,cj1424c).
occurs0(strainClinical43205,cj1424c).
occurs0(strainClinicall38556,cj1424c).
occurs0(strainClinicall39182,cj1424c).
occurs0(strainClinical45631,cj1424c).
occurs0(strainClinical55320,cj1424c).
occurs0(strainClinical55703,cj1424c).
occurs0(strainClinical30280,cj1424c).
occurs0(strainClinical30328,cj1424c).

occurs0(strainClinical41651,cj1424c).
occurs0(strainClinical43983,cj1424c).
occurs0(strainClinical40671,cj1424c).
occurs0(strainClinical56282,cj1424c).
occurs0(strainOvine12241,cj1424c).
occurs0(strainOvine12481,cj1424c).
occurs0(strainBovinevineine13305,cj1424c).
occurs0(strainChickenicken12450,cj1424c).
occurs0(strainChickenicken12487,cj1424c).
occurs0(strainChickenicken13082,cj1424c).
occurs0(strainChickenicken11856,cj1424c).
occurs0(strainClinical36069,cj1424c).
occurs0(strainClinical32787,cj1424c).
occurs0(strainClinicall31467,cj1424c).
occurs0(strainClinical44119,cj1424c).
occurs0(strainClinical34007,cj1424c).
occurs0(strainClinical38762,cj1424c).
occurs0(strainClinical38857,cj1424c).
occurs0(strainChicken47693,cj1424c).
occurs0(strainClinical63326,cj1424c).
occurs0(strainClinical64555,cj1424c).
occurs0(strainClinical59364,cj1424c).
occurs0(strainClinical59424,cj1424c).
occurs0(strainClinical33084,cj1424c).
occurs0(strainClinical31481,cj1424c).
occurs0(strainBeach1771,cj1424c).
occurs0(strainClinical53250,cj1424c).
occurs0(strainChickenicken13411,cj1425c).
occurs0(strainClinical44811,cj1425c).
occurs0(strainClinical48612,cj1425c).
occurs0(strainClinical58473,cj1425c).
occurs0(strainChicken11848,cj1425c).
occurs0(strainChicken12567,cj1425c).
occurs0(strainChicken11919,cj1425c).
occurs0(strainChicken11973,cj1425c).
occurs0(strainChicken13713,cj1425c).
occurs0(strainBeach1791,cj1425c).
occurs0(strainClinical43205,cj1425c).
occurs0(strainClinical33106,cj1425c).
occurs0(strainClinical44933,cj1425c).
occurs0(strainClinical55320,cj1425c).
occurs0(strainClinical55703,cj1425c).
occurs0(strainClinical30280,cj1425c).
occurs0(strainClinical30328,cj1425c).
occurs0(strainClinical41651,cj1425c).
occurs0(strainClinical43983,cj1425c).
occurs0(strainClinical40671,cj1425c).
occurs0(strainClinical44958,cj1425c).
occurs0(strainClinical56281,cj1425c).
occurs0(strainClinical56282,cj1425c).
occurs0(strainClinical56832,cj1425c).
occurs0(strainOvine12241,cj1425c).
occurs0(strainChicken12196,cj1425c).
occurs0(strainChickenicken12450,cj1425c).

occurs0(strainChickenicken12487,cj1425c).
occurs0(strainChickenicken13082,cj1425c).
occurs0(strainChickenicken13040,cj1425c).
occurs0(strainChickenicken11856,cj1425c).
occurs0(strainBeach1793,cj1425c).
occurs0(strainClinical56519,cj1425c).
occurs0(strainClinicall31467,cj1425c).
occurs0(strainClinical38857,cj1425c).
occurs0(strainChicken47693,cj1425c).
occurs0(strainClinical64555,cj1425c).
occurs0(strainClinical31481,cj1425c).
occurs0(strainBeach1771,cj1425c).
occurs0(strainClinical53250,cj1425c).
occurs0(strainChickenicken13411,cj1426c).
occurs0(strainClinical44811,cj1426c).
occurs0(strainClinical48612,cj1426c).
occurs0(strainChicken11848,cj1426c).
occurs0(strainChicken12567,cj1426c).
occurs0(strainChicken11919,cj1426c).
occurs0(strainChicken11973,cj1426c).
occurs0(strainChicken13713,cj1426c).
occurs0(strainBeach1791,cj1426c).
occurs0(strainClinical55320,cj1426c).
occurs0(strainClinical55703,cj1426c).
occurs0(strainClinical30280,cj1426c).
occurs0(strainClinical30328,cj1426c).
occurs0(strainChickenicken12450,cj1426c).
occurs0(strainChickenicken12487,cj1426c).
occurs0(strainChickenicken13082,cj1426c).
occurs0(strainCjejuniRM1221,cj1426c).
occurs0(strainChickenicken13411,cj1427c).
occurs0(strainClinical44811,cj1427c).
occurs0(strainClinical48612,cj1427c).
occurs0(strainChicken11848,cj1427c).
occurs0(strainChicken12567,cj1427c).
occurs0(strainChicken11919,cj1427c).
occurs0(strainChicken11973,cj1427c).
occurs0(strainChicken13713,cj1427c).
occurs0(strainBeach1791,cj1427c).
occurs0(strainClinical43205,cj1427c).
occurs0(strainClinical33106,cj1427c).
occurs0(strainClinical55703,cj1427c).
occurs0(strainClinical30280,cj1427c).
occurs0(strainClinical30328,cj1427c).
occurs0(strainClinical43983,cj1427c).
occurs0(strainClinical40671,cj1427c).
occurs0(strainOvine12241,cj1427c).
occurs0(strainOvine12481,cj1427c).
occurs0(strainBovinevineine13305,cj1427c).
occurs0(strainChickenicken12450,cj1427c).
occurs0(strainChickenicken12487,cj1427c).
occurs0(strainChickenicken13082,cj1427c).
occurs0(strainClinical33084,cj1427c).
occurs0(strainChickenicken13411,cj1428c).

occurs0(strainClinical44811,cj1428c).
occurs0(strainClinical48612,cj1428c).
occurs0(strainChicken11848,cj1428c).
occurs0(strainChicken12567,cj1428c).
occurs0(strainChicken11919,cj1428c).
occurs0(strainChicken11973,cj1428c).
occurs0(strainChicken13713,cj1428c).
occurs0(strainBeach1791,cj1428c).
occurs0(strainClinical55703,cj1428c).
occurs0(strainClinical30280,cj1428c).
occurs0(strainClinical30328,cj1428c).
occurs0(strainChickenicken12450,cj1428c).
occurs0(strainChickenicken12487,cj1428c).
occurs0(strainChickenicken13082,cj1428c).
occurs0(strainBeach1793,cj1428c).
occurs0(strainChickenicken13411,cj1429c).
occurs0(strainClinical44811,cj1429c).
occurs0(strainClinical48612,cj1429c).
occurs0(strainChicken11848,cj1429c).
occurs0(strainChicken12567,cj1429c).
occurs0(strainChicken11919,cj1429c).
occurs0(strainChicken11973,cj1429c).
occurs0(strainChicken13713,cj1429c).
occurs0(strainBeach1791,cj1429c).
occurs0(strainClinical55703,cj1429c).
occurs0(strainClinical30280,cj1429c).
occurs0(strainClinical30328,cj1429c).
occurs0(strainChickenicken12450,cj1429c).
occurs0(strainChickenicken12487,cj1429c).
occurs0(strainChickenicken13082,cj1429c).
occurs0(strainBeach1793,cj1429c).
occurs0(strainClinical38857,cj1429c).
occurs0(strainChickenicken13411,cj1430c).
occurs0(strainClinical44811,cj1430c).
occurs0(strainClinical48612,cj1430c).
occurs0(strainChicken11848,cj1430c).
occurs0(strainChicken12567,cj1430c).
occurs0(strainChicken11919,cj1430c).
occurs0(strainChicken11973,cj1430c).
occurs0(strainChicken13713,cj1430c).
occurs0(strainBeach1791,cj1430c).
occurs0(strainClinical55320,cj1430c).
occurs0(strainClinical55703,cj1430c).
occurs0(strainClinical30280,cj1430c).
occurs0(strainClinical30328,cj1430c).
occurs0(strainChickenicken12450,cj1430c).
occurs0(strainChickenicken12487,cj1430c).
occurs0(strainChickenicken13082,cj1430c).
occurs0(strainBeach1793,cj1430c).
occurs0(strainChickenicken13411,cj1431c).
occurs0(strainClinical44811,cj1431c).
occurs0(strainClinical48612,cj1431c).
occurs0(strainChicken11848,cj1431c).
occurs0(strainChicken12567,cj1431c).

occurs0(strainChicken11919,cj1431c).
occurs0(strainChicken11973,cj1431c).
occurs0(strainChicken13713,cj1431c).
occurs0(strainBeach1791,cj1431c).
occurs0(strainClinical55703,cj1431c).
occurs0(strainClinical30280,cj1431c).
occurs0(strainClinical30328,cj1431c).
occurs0(strainChickenicken12450,cj1431c).
occurs0(strainChickenicken12487,cj1431c).
occurs0(strainChickenicken13082,cj1431c).
occurs0(strainChicken40209,cj1431c).
occurs0(strainChickenicken13411,cj1432c).
occurs0(strainClinical44811,cj1432c).
occurs0(strainClinical48612,cj1432c).
occurs0(strainChicken11848,cj1432c).
occurs0(strainChicken12567,cj1432c).
occurs0(strainChicken11919,cj1432c).
occurs0(strainChicken11973,cj1432c).
occurs0(strainChicken13713,cj1432c).
occurs0(strainBeach1791,cj1432c).
occurs0(strainClinical55320,cj1432c).
occurs0(strainClinical55703,cj1432c).
occurs0(strainClinical30280,cj1432c).
occurs0(strainClinical30328,cj1432c).
occurs0(strainChickenicken12450,cj1432c).
occurs0(strainChickenicken12487,cj1432c).
occurs0(strainChickenicken13082,cj1432c).
occurs0(strainChickenicken13411,cj1433c).
occurs0(strainClinical44811,cj1433c).
occurs0(strainClinical48612,cj1433c).
occurs0(strainChicken11848,cj1433c).
occurs0(strainChicken12567,cj1433c).
occurs0(strainChicken11919,cj1433c).
occurs0(strainChicken11973,cj1433c).
occurs0(strainChicken13713,cj1433c).
occurs0(strainBeach1791,cj1433c).
occurs0(strainClinical55320,cj1433c).
occurs0(strainClinical55703,cj1433c).
occurs0(strainClinical30280,cj1433c).
occurs0(strainClinical30328,cj1433c).
occurs0(strainClinical43983,cj1433c).
occurs0(strainChickenicken12450,cj1433c).
occurs0(strainChickenicken12487,cj1433c).
occurs0(strainChickenicken13082,cj1433c).
occurs0(strainChickenicken13411,cj1434c).
occurs0(strainClinical44811,cj1434c).
occurs0(strainClinical48612,cj1434c).
occurs0(strainChicken11848,cj1434c).
occurs0(strainChicken11919,cj1434c).
occurs0(strainChicken11973,cj1434c).
occurs0(strainChicken13713,cj1434c).
occurs0(strainBeach1791,cj1434c).
occurs0(strainClinical55703,cj1434c).
occurs0(strainClinical30280,cj1434c).

occurs0(strainClinical30328,cj1434c).
occurs0(strainChickenicken12450,cj1434c).
occurs0(strainChickenicken12487,cj1434c).
occurs0(strainChickenicken13082,cj1434c).
occurs0(strainChickenicken13411,cj1435c).
occurs0(strainClinical44811,cj1435c).
occurs0(strainClinical48612,cj1435c).
occurs0(strainChicken11848,cj1435c).
occurs0(strainChicken12567,cj1435c).
occurs0(strainChicken11919,cj1435c).
occurs0(strainChicken11973,cj1435c).
occurs0(strainChicken13713,cj1435c).
occurs0(strainBeach1791,cj1435c).
occurs0(strainClinical55703,cj1435c).
occurs0(strainClinical30280,cj1435c).
occurs0(strainClinical30328,cj1435c).
occurs0(strainClinical43983,cj1435c).
occurs0(strainChickenicken12450,cj1435c).
occurs0(strainChickenicken12487,cj1435c).
occurs0(strainChickenicken13082,cj1435c).
occurs0(strainClinical36439,cj1435c).
occurs0(strainChickenicken13411,cj1436c).
occurs0(strainClinical44811,cj1436c).
occurs0(strainClinical48612,cj1436c).
occurs0(strainChicken11848,cj1436c).
occurs0(strainChicken12567,cj1436c).
occurs0(strainChicken11919,cj1436c).
occurs0(strainChicken11973,cj1436c).
occurs0(strainChicken13713,cj1436c).
occurs0(strainBeach1791,cj1436c).
occurs0(strainClinical55320,cj1436c).
occurs0(strainClinical55703,cj1436c).
occurs0(strainClinical30280,cj1436c).
occurs0(strainClinical30328,cj1436c).
occurs0(strainChickenicken12450,cj1436c).
occurs0(strainChickenicken12487,cj1436c).
occurs0(strainChickenicken13082,cj1436c).
occurs0(strainChickenicken13411,cj1437c).
occurs0(strainClinical44811,cj1437c).
occurs0(strainClinical48612,cj1437c).
occurs0(strainChicken11848,cj1437c).
occurs0(strainChicken12567,cj1437c).
occurs0(strainChicken11919,cj1437c).
occurs0(strainChicken11973,cj1437c).
occurs0(strainChicken13713,cj1437c).
occurs0(strainBeach1791,cj1437c).
occurs0(strainClinical55703,cj1437c).
occurs0(strainClinical30280,cj1437c).
occurs0(strainClinical30328,cj1437c).
occurs0(strainChickenicken12450,cj1437c).
occurs0(strainChickenicken12487,cj1437c).
occurs0(strainChickenicken13082,cj1437c).
occurs0(strainChickenicken13411,cj1438c).
occurs0(strainClinical44811,cj1438c).

occurs0(strainClinical48612,cj1438c).
occurs0(strainChicken11848,cj1438c).
occurs0(strainChicken12567,cj1438c).
occurs0(strainChicken11919,cj1438c).
occurs0(strainChicken11973,cj1438c).
occurs0(strainChicken13713,cj1438c).
occurs0(strainBeach1791,cj1438c).
occurs0(strainClinical55703,cj1438c).
occurs0(strainClinical30280,cj1438c).
occurs0(strainClinical30328,cj1438c).
occurs0(strainChickenicken12450,cj1438c).
occurs0(strainChickenicken12487,cj1438c).
occurs0(strainChickenicken13082,cj1438c).
occurs0(strainChickenicken13411,cj1439c).
occurs0(strainClinical44811,cj1439c).
occurs0(strainClinical48612,cj1439c).
occurs0(strainChicken11848,cj1439c).
occurs0(strainChicken12567,cj1439c).
occurs0(strainChicken11919,cj1439c).
occurs0(strainChicken11973,cj1439c).
occurs0(strainChicken13713,cj1439c).
occurs0(strainBeach1791,cj1439c).
occurs0(strainClinical55703,cj1439c).
occurs0(strainClinical30280,cj1439c).
occurs0(strainClinical30328,cj1439c).
occurs0(strainClinical43983,cj1439c).
occurs0(strainChickenicken12450,cj1439c).
occurs0(strainChickenicken12487,cj1439c).
occurs0(strainChickenicken13082,cj1439c).
occurs0(strainClinical36439,cj1439c).
occurs0(strainChickenicken13411,cj1440c).
occurs0(strainClinical44811,cj1440c).
occurs0(strainClinical48612,cj1440c).
occurs0(strainChicken11848,cj1440c).
occurs0(strainChicken12567,cj1440c).
occurs0(strainChicken11919,cj1440c).
occurs0(strainChicken11973,cj1440c).
occurs0(strainChicken13713,cj1440c).
occurs0(strainBeach1791,cj1440c).
occurs0(strainClinical55320,cj1440c).
occurs0(strainClinical55703,cj1440c).
occurs0(strainClinical30280,cj1440c).
occurs0(strainClinical30328,cj1440c).
occurs0(strainClinical56281,cj1440c).
occurs0(strainChickenicken12450,cj1440c).
occurs0(strainChickenicken12487,cj1440c).
occurs0(strainChickenicken13082,cj1440c).
occurs0(strainChickenicken13411,cj1441c).
occurs0(strainClinical44811,cj1441c).
occurs0(strainClinical48612,cj1441c).
occurs0(strainChicken11848,cj1441c).
occurs0(strainChicken12567,cj1441c).
occurs0(strainChicken11919,cj1441c).
occurs0(strainChicken11973,cj1441c).

occurs0(strainChicken13713,cj1441c).
occurs0(strainBeach1791,cj1441c).
occurs0(strainClinical55320,cj1441c).
occurs0(strainClinical55703,cj1441c).
occurs0(strainClinical30280,cj1441c).
occurs0(strainClinical30328,cj1441c).
occurs0(strainClinical56281,cj1441c).
occurs0(strainChickenicken12450,cj1441c).
occurs0(strainChickenicken12487,cj1441c).
occurs0(strainChickenicken13082,cj1441c).
occurs0(strainChickenicken13411,cj1442c).
occurs0(strainClinical44811,cj1442c).
occurs0(strainClinical48612,cj1442c).
occurs0(strainClinical58473,cj1442c).
occurs0(strainChicken11848,cj1442c).
occurs0(strainChicken12567,cj1442c).
occurs0(strainChicken11919,cj1442c).
occurs0(strainChicken11973,cj1442c).
occurs0(strainChicken13713,cj1442c).
occurs0(strainBeach1791,cj1442c).
occurs0(strainClinical43205,cj1442c).
occurs0(strainClinical55703,cj1442c).
occurs0(strainClinical30280,cj1442c).
occurs0(strainClinical30328,cj1442c).
occurs0(strainClinical41651,cj1442c).
occurs0(strainClinical43983,cj1442c).
occurs0(strainClinical40671,cj1442c).
occurs0(strainClinical44958,cj1442c).
occurs0(strainClinical52331,cj1442c).
occurs0(strainClinical56281,cj1442c).
occurs0(strainClinical56282,cj1442c).
occurs0(strainOvine12241,cj1442c).
occurs0(strainOvine12481,cj1442c).
occurs0(strainBovinevineine13305,cj1442c).
occurs0(strainChickenicken11818,cj1442c).
occurs0(strainChicken12196,cj1442c).
occurs0(strainChickenicken12450,cj1442c).
occurs0(strainChickenicken12487,cj1442c).
occurs0(strainChickenicken13082,cj1442c).
occurs0(strainChickenicken13040,cj1442c).
occurs0(strainChickenicken11856,cj1442c).
occurs0(strainClinical56519,cj1442c).
occurs0(strainChicken47693,cj1442c).
occurs0(strainBeach1771,cj1442c).
occurs0(strainClinical53250,cj1442c).
occurs0(strainClinicalG1,cj1443c).
occurs0(strainClinicalG3,cj1443c).
occurs0(strainChickenicken11974,cj1443c).
occurs0(strainChickenicken13249,cj1443c).
occurs0(strainChickenicken13411,cj1443c).
occurs0(strainClinical44811,cj1443c).
occurs0(strainClinical48612,cj1443c).
occurs0(strainClinical58473,cj1443c).
occurs0(strainChicken11848,cj1443c).

occurs0(strainChicken12567,cj1443c).
occurs0(strainChicken11919,cj1443c).
occurs0(strainChicken11973,cj1443c).
occurs0(strainChicken13713,cj1443c).
occurs0(strainBeach1791,cj1443c).
occurs0(strainClinical43205,cj1443c).
occurs0(strainClinical33106,cj1443c).
occurs0(strainClinicall38556,cj1443c).
occurs0(strainClinicall39182,cj1443c).
occurs0(strainClinical44933,cj1443c).
occurs0(strainClinical45631,cj1443c).
occurs0(strainClinical55320,cj1443c).
occurs0(strainClinical55703,cj1443c).
occurs0(strainClinical30280,cj1443c).
occurs0(strainClinical30328,cj1443c).
occurs0(strainClinical41651,cj1443c).
occurs0(strainClinical32799,cj1443c).
occurs0(strainClinical43983,cj1443c).
occurs0(strainClinical40671,cj1443c).
occurs0(strainClinical44958,cj1443c).
occurs0(strainClinical52331,cj1443c).
occurs0(strainClinical56281,cj1443c).
occurs0(strainClinical56282,cj1443c).
occurs0(strainClinical56832,cj1443c).
occurs0(strainOvine12241,cj1443c).
occurs0(strainOvine12481,cj1443c).
occurs0(strainBovinevineine13305,cj1443c).
occurs0(strainChicken12196,cj1443c).
occurs0(strainChickenicken12450,cj1443c).
occurs0(strainChickenicken12487,cj1443c).
occurs0(strainChickenicken13082,cj1443c).
occurs0(strainChickenicken13040,cj1443c).
occurs0(strainChickenicken11856,cj1443c).
occurs0(strainClinical36069,cj1443c).
occurs0(strainChicken40209,cj1443c).
occurs0(strainBeach1793,cj1443c).
occurs0(strainClinical81116,cj1443c).
occurs0(strainClinical56519,cj1443c).
occurs0(strainClinical44119,cj1443c).
occurs0(strainClinical34007,cj1443c).
occurs0(strainClinicalinical15168,cj1443c).
occurs0(strainClinical18836,cj1443c).
occurs0(strainClinicalM1,cj1443c).
occurs0(strainClinical36860,cj1443c).
occurs0(strainClinical40917,cj1443c).
occurs0(strainClinical38857,cj1443c).
occurs0(strainChicken47693,cj1443c).
occurs0(strainClinical63326,cj1443c).
occurs0(strainClinical64555,cj1443c).
occurs0(strainClinical59364,cj1443c).
occurs0(strainClinical59424,cj1443c).
occurs0(strainClinical33084,cj1443c).
occurs0(strainClinical31481,cj1443c).
occurs0(strainClinical39828,cj1443c).

occurs0(strainBeach1771,cj1443c).
occurs0(strainClinical53250,cj1443c).
occurs0(strainClinical45557,cj1443c).
occurs0(strainClinical47939,cj1443c).
occurs0(strainClinicalG1,cj1444c).
occurs0(strainClinicalG3,cj1444c).
occurs0(strainChickenicken11974,cj1444c).
occurs0(strainChickenicken13249,cj1444c).
occurs0(strainChickenicken13411,cj1444c).
occurs0(strainClinical44811,cj1444c).
occurs0(strainClinical48612,cj1444c).
occurs0(strainClinical58473,cj1444c).
occurs0(strainChicken11848,cj1444c).
occurs0(strainChicken12567,cj1444c).
occurs0(strainChicken11919,cj1444c).
occurs0(strainChicken11973,cj1444c).
occurs0(strainChicken13713,cj1444c).
occurs0(strainBeach1791,cj1444c).
occurs0(strainClinical43205,cj1444c).
occurs0(strainClinical33106,cj1444c).
occurs0(strainClinicall38556,cj1444c).
occurs0(strainClinicall39182,cj1444c).
occurs0(strainClinical44933,cj1444c).
occurs0(strainClinical45631,cj1444c).
occurs0(strainClinical55320,cj1444c).
occurs0(strainClinical55703,cj1444c).
occurs0(strainClinical30280,cj1444c).
occurs0(strainClinical30328,cj1444c).
occurs0(strainClinical41651,cj1444c).
occurs0(strainClinical32799,cj1444c).
occurs0(strainClinical43983,cj1444c).
occurs0(strainClinical40671,cj1444c).
occurs0(strainClinical44958,cj1444c).
occurs0(strainClinical52331,cj1444c).
occurs0(strainClinical56281,cj1444c).
occurs0(strainClinical56282,cj1444c).
occurs0(strainClinical56832,cj1444c).
occurs0(strainOvine12241,cj1444c).
occurs0(strainOvine12481,cj1444c).
occurs0(strainBovinevineine13305,cj1444c).
occurs0(strainChickenicken12912,cj1444c).
occurs0(strainChickenicken11818,cj1444c).
occurs0(strainChicken12196,cj1444c).
occurs0(strainChickenicken12450,cj1444c).
occurs0(strainChickenicken12487,cj1444c).
occurs0(strainChickenicken13082,cj1444c).
occurs0(strainChickenicken13040,cj1444c).
occurs0(strainChickenicken11856,cj1444c).
occurs0(strainClinical36069,cj1444c).
occurs0(strainChicken40209,cj1444c).
occurs0(strainBeach1793,cj1444c).
occurs0(strainClinical81116,cj1444c).
occurs0(strainClinical36439,cj1444c).
occurs0(strainClinical56519,cj1444c).

occurs0(strainClinical32787,cj1444c).
occurs0(strainClinicall31467,cj1444c).
occurs0(strainClinical44119,cj1444c).
occurs0(strainClinical34007,cj1444c).
occurs0(strainClinical38762,cj1444c).
occurs0(strainClinicalinical15168,cj1444c).
occurs0(strainClinical18836,cj1444c).
occurs0(strainClinicalM1,cj1444c).
occurs0(strainClinical36860,cj1444c).
occurs0(strainClinical40917,cj1444c).
occurs0(strainClinical38857,cj1444c).
occurs0(strainCjejuniRM1221,cj1444c).
occurs0(strainChicken47693,cj1444c).
occurs0(strainClinical63326,cj1444c).
occurs0(strainClinical64555,cj1444c).
occurs0(strainClinical59364,cj1444c).
occurs0(strainClinical59424,cj1444c).
occurs0(strainClinical33084,cj1444c).
occurs0(strainClinical31481,cj1444c).
occurs0(strainClinical39828,cj1444c).
occurs0(strainBeach1771,cj1444c).
occurs0(strainClinical53250,cj1444c).
occurs0(strainClinical45557,cj1444c).
occurs0(strainClinical47939,cj1444c).
occurs0(strainClinicalG1,cj1445c).
occurs0(strainChickenicken11974,cj1445c).
occurs0(strainChickenicken13249,cj1445c).
occurs0(strainChickenicken13411,cj1445c).
occurs0(strainClinical44811,cj1445c).
occurs0(strainClinical48612,cj1445c).
occurs0(strainClinical58473,cj1445c).
occurs0(strainChicken11848,cj1445c).
occurs0(strainChicken12567,cj1445c).
occurs0(strainChicken11919,cj1445c).
occurs0(strainChicken11973,cj1445c).
occurs0(strainChicken13713,cj1445c).
occurs0(strainBeach1791,cj1445c).
occurs0(strainClinical43205,cj1445c).
occurs0(strainClinical33106,cj1445c).
occurs0(strainClinicall38556,cj1445c).
occurs0(strainClinicall39182,cj1445c).
occurs0(strainClinical44933,cj1445c).
occurs0(strainClinical45631,cj1445c).
occurs0(strainClinical55320,cj1445c).
occurs0(strainClinical55703,cj1445c).
occurs0(strainClinical30280,cj1445c).
occurs0(strainClinical30328,cj1445c).
occurs0(strainClinical41651,cj1445c).
occurs0(strainClinical43983,cj1445c).
occurs0(strainClinical40671,cj1445c).
occurs0(strainClinical44958,cj1445c).
occurs0(strainClinical52331,cj1445c).
occurs0(strainClinical56281,cj1445c).
occurs0(strainClinical56282,cj1445c).

occurs0(strainClinical56832,cj1445c).
occurs0(strainOvine12241,cj1445c).
occurs0(strainOvine12481,cj1445c).
occurs0(strainBovinevineine13305,cj1445c).
occurs0(strainChickenicken12912,cj1445c).
occurs0(strainChicken12196,cj1445c).
occurs0(strainChickenicken12450,cj1445c).
occurs0(strainChickenicken12487,cj1445c).
occurs0(strainChickenicken13082,cj1445c).
occurs0(strainChickenicken13040,cj1445c).
occurs0(strainChickenicken11856,cj1445c).
occurs0(strainClinical36069,cj1445c).
occurs0(strainChicken40209,cj1445c).
occurs0(strainBeach1793,cj1445c).
occurs0(strainClinical81116,cj1445c).
occurs0(strainClinical36439,cj1445c).
occurs0(strainClinical56519,cj1445c).
occurs0(strainClinical32787,cj1445c).
occurs0(strainClinicall31467,cj1445c).
occurs0(strainClinical44119,cj1445c).
occurs0(strainClinical34007,cj1445c).
occurs0(strainClinical38762,cj1445c).
occurs0(strainClinicalinical15168,cj1445c).
occurs0(strainClinical18836,cj1445c).
occurs0(strainClinicalM1,cj1445c).
occurs0(strainClinical36860,cj1445c).
occurs0(strainClinical40917,cj1445c).
occurs0(strainClinical38857,cj1445c).
occurs0(strainCjejuniRM1221,cj1445c).
occurs0(strainChicken47693,cj1445c).
occurs0(strainClinical63326,cj1445c).
occurs0(strainClinical64555,cj1445c).
occurs0(strainClinical59364,cj1445c).
occurs0(strainClinical59424,cj1445c).
occurs0(strainClinical33084,cj1445c).
occurs0(strainClinical31481,cj1445c).
occurs0(strainBeach1771,cj1445c).
occurs0(strainClinical53250,cj1445c).
occurs0(strainClinical45557,cj1445c).
occurs0(strainClinical47939,cj1445c).
occurs0(strainChickenicken13411,cj1447c).
occurs0(strainClinical44811,cj1447c).
occurs0(strainClinical48612,cj1447c).
occurs0(strainClinical58473,cj1447c).
occurs0(strainChicken11848,cj1447c).
occurs0(strainChicken12567,cj1447c).
occurs0(strainChicken11919,cj1447c).
occurs0(strainChicken11973,cj1447c).
occurs0(strainChicken13713,cj1447c).
occurs0(strainBeach1791,cj1447c).
occurs0(strainClinical43205,cj1447c).
occurs0(strainClinical55703,cj1447c).
occurs0(strainClinical30280,cj1447c).
occurs0(strainClinical30328,cj1447c).

occurs0(strainChickenicken12912,cj1447c).
occurs0(strainChickenicken12450,cj1447c).
occurs0(strainChickenicken12487,cj1447c).
occurs0(strainChickenicken13082,cj1447c).
occurs0(strainChicken40209,cj1447c).
occurs0(strainClinical81116,cj1447c).
occurs0(strainCjejuniRM1221,cj1447c).
occurs0(strainChickenicken13411,cj1448c).
occurs0(strainClinical44811,cj1448c).
occurs0(strainClinical48612,cj1448c).
occurs0(strainClinical58473,cj1448c).
occurs0(strainChicken11848,cj1448c).
occurs0(strainChicken12567,cj1448c).
occurs0(strainChicken11919,cj1448c).
occurs0(strainChicken11973,cj1448c).
occurs0(strainChicken13713,cj1448c).
occurs0(strainBeach1791,cj1448c).
occurs0(strainClinical43205,cj1448c).
occurs0(strainClinical55320,cj1448c).
occurs0(strainClinical55703,cj1448c).
occurs0(strainClinical30280,cj1448c).
occurs0(strainClinical30328,cj1448c).
occurs0(strainChickenicken12912,cj1448c).
occurs0(strainChickenicken12450,cj1448c).
occurs0(strainChickenicken12487,cj1448c).
occurs0(strainChickenicken13082,cj1448c).
occurs0(strainChicken40209,cj1448c).
occurs0(strainBeach1793,cj1448c).
occurs0(strainClinical81116,cj1448c).
occurs0(strainClinical36860,cj1448c).
occurs0(strainClinical53250,cj1448c).
absent(strainClinicalG3,cj1414c).
absent(strainClinical32799,cj1414c).
absent(strainClinical43983,cj1414c).
absent(strainBovinevineine13305,cj1414c).
absent(strainChickenicken11818,cj1414c).
absent(strainClinical81116,cj1414c).
absent(strainClinical36439,cj1414c).
absent(strainClinical32787,cj1414c).
absent(strainClinicall31467,cj1414c).
absent(strainClinical38762,cj1414c).
absent(strainClinicalM1,cj1414c).
absent(strainClinical36860,cj1414c).
absent(strainClinical40917,cj1414c).
absent(strainClinicall38556,cj1415c).
absent(strainClinicall39182,cj1415c).
absent(strainClinical44933,cj1415c).
absent(strainClinical45631,cj1415c).
absent(strainClinical55320,cj1415c).
absent(strainClinical36069,cj1415c).
absent(strainClinical81116,cj1415c).
absent(strainClinical36439,cj1415c).
absent(strainClinical56519,cj1415c).
absent(strainClinical44119,cj1415c).

absent(strainClinical34007,cj1415c).
absent(strainClinicalM1,cj1415c).
absent(strainClinical36860,cj1415c).
absent(strainClinical40917,cj1415c).
absent(strainChicken47693,cj1415c).
absent(strainClinical63326,cj1415c).
absent(strainClinical64555,cj1415c).
absent(strainClinical59364,cj1415c).
absent(strainClinical59424,cj1415c).
absent(strainBeach1771,cj1415c).
absent(strainClinical53250,cj1415c).
absent(strainClinical47939,cj1415c).
absent(strainClinicall38556,cj1416c).
absent(strainClinicall39182,cj1416c).
absent(strainClinical44933,cj1416c).
absent(strainClinical45631,cj1416c).
absent(strainClinical36069,cj1416c).
absent(strainClinical36439,cj1416c).
absent(strainClinical44119,cj1416c).
absent(strainClinical34007,cj1416c).
absent(strainCjejuniRM1221,cj1416c).
absent(strainClinical63326,cj1416c).
absent(strainClinical64555,cj1416c).
absent(strainClinical59364,cj1416c).
absent(strainClinical59424,cj1416c).
absent(strainBeach1771,cj1416c).
absent(strainClinical47939,cj1416c).
absent(strainClinicall38556,cj1417c).
absent(strainClinicall39182,cj1417c).
absent(strainClinical44933,cj1417c).
absent(strainClinical45631,cj1417c).
absent(strainClinical55320,cj1417c).
absent(strainClinical36069,cj1417c).
absent(strainClinical36439,cj1417c).
absent(strainClinical44119,cj1417c).
absent(strainClinical34007,cj1417c).
absent(strainCjejuniRM1221,cj1417c).
absent(strainClinical63326,cj1417c).
absent(strainClinical64555,cj1417c).
absent(strainClinical59364,cj1417c).
absent(strainClinical59424,cj1417c).
absent(strainBeach1771,cj1417c).
absent(strainClinical47939,cj1417c).
absent(strainClinicall38556,cj1418c).
absent(strainClinicall39182,cj1418c).
absent(strainClinical44933,cj1418c).
absent(strainClinical45631,cj1418c).
absent(strainClinical55320,cj1418c).
absent(strainClinical36069,cj1418c).
absent(strainClinical36439,cj1418c).
absent(strainClinical44119,cj1418c).
absent(strainClinical34007,cj1418c).
absent(strainCjejuniRM1221,cj1418c).
absent(strainClinical63326,cj1418c).

absent(strainClinical64555,cj1418c).
absent(strainClinical59364,cj1418c).
absent(strainClinical59424,cj1418c).
absent(strainBeach1771,cj1418c).
absent(strainClinical47939,cj1418c).
absent(strainClinicall38556,cj1419c).
absent(strainClinicall39182,cj1419c).
absent(strainClinical44933,cj1419c).
absent(strainClinical45631,cj1419c).
absent(strainClinical55320,cj1419c).
absent(strainClinical36069,cj1419c).
absent(strainClinical36439,cj1419c).
absent(strainClinical44119,cj1419c).
absent(strainClinical34007,cj1419c).
absent(strainCjejuniRM1221,cj1419c).
absent(strainClinical63326,cj1419c).
absent(strainClinical64555,cj1419c).
absent(strainClinical59364,cj1419c).
absent(strainClinical59424,cj1419c).
absent(strainBeach1771,cj1419c).
absent(strainClinical47939,cj1419c).
absent(strainClinicalG1,cj1421c).
absent(strainClinicalG3,cj1421c).
absent(strainChickenicken11974,cj1421c).
absent(strainChickenicken13249,cj1421c).
absent(strainClinical58473,cj1421c).
absent(strainClinical43205,cj1421c).
absent(strainClinical33106,cj1421c).
absent(strainClinicall38556,cj1421c).
absent(strainClinicall39182,cj1421c).
absent(strainClinical44933,cj1421c).
absent(strainClinical45631,cj1421c).
absent(strainClinical55320,cj1421c).
absent(strainClinical41651,cj1421c).
absent(strainClinical32799,cj1421c).
absent(strainClinical43983,cj1421c).
absent(strainClinical40671,cj1421c).
absent(strainClinical44958,cj1421c).
absent(strainClinical52331,cj1421c).
absent(strainClinical56281,cj1421c).
absent(strainClinical56282,cj1421c).
absent(strainClinical56832,cj1421c).
absent(strainOvine12241,cj1421c).
absent(strainOvine12481,cj1421c).
absent(strainBovinevineine13305,cj1421c).
absent(strainChickenicken12912,cj1421c).
absent(strainChickenicken11818,cj1421c).
absent(strainChicken12196,cj1421c).
absent(strainChickenicken13040,cj1421c).
absent(strainChickenicken11856,cj1421c).
absent(strainClinical36069,cj1421c).
absent(strainChicken40209,cj1421c).
absent(strainBeach1793,cj1421c).
absent(strainClinical81116,cj1421c).

absent(strainClinical36439,cj1421c).
absent(strainClinical56519,cj1421c).
absent(strainClinical32787,cj1421c).
absent(strainClinicall31467,cj1421c).
absent(strainClinical44119,cj1421c).
absent(strainClinical34007,cj1421c).
absent(strainClinical38762,cj1421c).
absent(strainClinicalinical15168,cj1421c).
absent(strainClinical18836,cj1421c).
absent(strainClinicalM1,cj1421c).
absent(strainClinical36860,cj1421c).
absent(strainClinical40917,cj1421c).
absent(strainClinical38857,cj1421c).
absent(strainChicken47693,cj1421c).
absent(strainClinical63326,cj1421c).
absent(strainClinical64555,cj1421c).
absent(strainClinical59364,cj1421c).
absent(strainClinical59424,cj1421c).
absent(strainClinical33084,cj1421c).
absent(strainClinical31481,cj1421c).
absent(strainClinical39828,cj1421c).
absent(strainBeach1771,cj1421c).
absent(strainClinical53250,cj1421c).
absent(strainClinical45557,cj1421c).
absent(strainClinical47939,cj1421c).
absent(strainClinicalG1,cj1422c).
absent(strainClinicalG3,cj1422c).
absent(strainChickenicken11974,cj1422c).
absent(strainChickenicken13249,cj1422c).
absent(strainClinical58473,cj1422c).
absent(strainClinical43205,cj1422c).
absent(strainClinical33106,cj1422c).
absent(strainClinicall38556,cj1422c).
absent(strainClinicall39182,cj1422c).
absent(strainClinical44933,cj1422c).
absent(strainClinical45631,cj1422c).
absent(strainClinical41651,cj1422c).
absent(strainClinical32799,cj1422c).
absent(strainClinical43983,cj1422c).
absent(strainClinical40671,cj1422c).
absent(strainClinical44958,cj1422c).
absent(strainClinical52331,cj1422c).
absent(strainClinical56281,cj1422c).
absent(strainClinical56282,cj1422c).
absent(strainClinical56832,cj1422c).
absent(strainOvine12241,cj1422c).
absent(strainOvine12481,cj1422c).
absent(strainBovinevineine13305,cj1422c).
absent(strainChickenicken12912,cj1422c).
absent(strainChickenicken11818,cj1422c).
absent(strainChicken12196,cj1422c).
absent(strainChickenicken13040,cj1422c).
absent(strainChickenicken11856,cj1422c).
absent(strainClinical36069,cj1422c).

absent(strainChicken40209,cj1422c).
absent(strainBeach1793,cj1422c).
absent(strainClinical81116,cj1422c).
absent(strainClinical36439,cj1422c).
absent(strainClinical56519,cj1422c).
absent(strainClinical32787,cj1422c).
absent(strainClinicall31467,cj1422c).
absent(strainClinical44119,cj1422c).
absent(strainClinical34007,cj1422c).
absent(strainClinical38762,cj1422c).
absent(strainClinicalinical15168,cj1422c).
absent(strainClinical18836,cj1422c).
absent(strainClinicalM1,cj1422c).
absent(strainClinical36860,cj1422c).
absent(strainClinical40917,cj1422c).
absent(strainClinical38857,cj1422c).
absent(strainCjejuniRM1221,cj1422c).
absent(strainChicken47693,cj1422c).
absent(strainClinical63326,cj1422c).
absent(strainClinical64555,cj1422c).
absent(strainClinical59364,cj1422c).
absent(strainClinical59424,cj1422c).
absent(strainClinical33084,cj1422c).
absent(strainClinical31481,cj1422c).
absent(strainBeach1771,cj1422c).
absent(strainClinical53250,cj1422c).
absent(strainClinical45557,cj1422c).
absent(strainClinical47939,cj1422c).
absent(strainClinicalG1,cj1423c).
absent(strainClinicalG3,cj1423c).
absent(strainChickenicken11974,cj1423c).
absent(strainChickenicken13249,cj1423c).
absent(strainClinical58473,cj1423c).
absent(strainClinical33106,cj1423c).
absent(strainClinicall38556,cj1423c).
absent(strainClinicall39182,cj1423c).
absent(strainClinical44933,cj1423c).
absent(strainClinical45631,cj1423c).
absent(strainClinical32799,cj1423c).
absent(strainClinical43983,cj1423c).
absent(strainClinical40671,cj1423c).
absent(strainClinical52331,cj1423c).
absent(strainClinical56832,cj1423c).
absent(strainOvine12241,cj1423c).
absent(strainOvine12481,cj1423c).
absent(strainBovinevineine13305,cj1423c).
absent(strainChickenicken12912,cj1423c).
absent(strainChickenicken11818,cj1423c).
absent(strainChicken12196,cj1423c).
absent(strainChickenicken13040,cj1423c).
absent(strainChickenicken11856,cj1423c).
absent(strainClinical36069,cj1423c).
absent(strainChicken40209,cj1423c).
absent(strainClinical81116,cj1423c).

absent(strainClinical36439,cj1423c).
absent(strainClinical56519,cj1423c).
absent(strainClinical32787,cj1423c).
absent(strainClinicall31467,cj1423c).
absent(strainClinical44119,cj1423c).
absent(strainClinical34007,cj1423c).
absent(strainClinical38762,cj1423c).
absent(strainClinicalinical15168,cj1423c).
absent(strainClinical18836,cj1423c).
absent(strainClinicalM1,cj1423c).
absent(strainClinical36860,cj1423c).
absent(strainClinical40917,cj1423c).
absent(strainClinical38857,cj1423c).
absent(strainCjejuniRM1221,cj1423c).
absent(strainChicken47693,cj1423c).
absent(strainClinical63326,cj1423c).
absent(strainClinical64555,cj1423c).
absent(strainClinical59364,cj1423c).
absent(strainClinical59424,cj1423c).
absent(strainClinical31481,cj1423c).
absent(strainClinical39828,cj1423c).
absent(strainBeach1771,cj1423c).
absent(strainClinical45557,cj1423c).
absent(strainClinical47939,cj1423c).
absent(strainClinicalG1,cj1424c).
absent(strainClinicalG3,cj1424c).
absent(strainChickenicken11974,cj1424c).
absent(strainChickenicken13249,cj1424c).
absent(strainClinical58473,cj1424c).
absent(strainClinical33106,cj1424c).
absent(strainClinical44933,cj1424c).
absent(strainClinical32799,cj1424c).
absent(strainClinical44958,cj1424c).
absent(strainClinical52331,cj1424c).
absent(strainClinical56281,cj1424c).
absent(strainClinical56832,cj1424c).
absent(strainChickenicken12912,cj1424c).
absent(strainChickenicken11818,cj1424c).
absent(strainChicken12196,cj1424c).
absent(strainChickenicken13040,cj1424c).
absent(strainChicken40209,cj1424c).
absent(strainBeach1793,cj1424c).
absent(strainClinical36439,cj1424c).
absent(strainClinical56519,cj1424c).
absent(strainClinicalinical15168,cj1424c).
absent(strainClinical18836,cj1424c).
absent(strainClinicalM1,cj1424c).
absent(strainClinical36860,cj1424c).
absent(strainClinical40917,cj1424c).
absent(strainCjejuniRM1221,cj1424c).
absent(strainClinical39828,cj1424c).
absent(strainClinical45557,cj1424c).
absent(strainClinical47939,cj1424c).
absent(strainClinicalG1,cj1425c).

absent(strainClinicalG3,cj1425c).
absent(strainChickenicken11974,cj1425c).
absent(strainChickenicken13249,cj1425c).
absent(strainClinicall38556,cj1425c).
absent(strainClinicall39182,cj1425c).
absent(strainClinical45631,cj1425c).
absent(strainClinical32799,cj1425c).
absent(strainClinical52331,cj1425c).
absent(strainOvine12481,cj1425c).
absent(strainBovinevineine13305,cj1425c).
absent(strainChickenicken12912,cj1425c).
absent(strainChickenicken11818,cj1425c).
absent(strainClinical36069,cj1425c).
absent(strainChicken40209,cj1425c).
absent(strainClinical36439,cj1425c).
absent(strainClinical32787,cj1425c).
absent(strainClinical44119,cj1425c).
absent(strainClinical34007,cj1425c).
absent(strainClinical38762,cj1425c).
absent(strainClinicalinical15168,cj1425c).
absent(strainClinical18836,cj1425c).
absent(strainClinicalM1,cj1425c).
absent(strainClinical36860,cj1425c).
absent(strainClinical40917,cj1425c).
absent(strainCjejuniRM1221,cj1425c).
absent(strainClinical63326,cj1425c).
absent(strainClinical59364,cj1425c).
absent(strainClinical59424,cj1425c).
absent(strainClinical33084,cj1425c).
absent(strainClinical39828,cj1425c).
absent(strainClinical45557,cj1425c).
absent(strainClinical47939,cj1425c).
absent(strainClinicalG1,cj1426c).
absent(strainClinicalG3,cj1426c).
absent(strainChickenicken11974,cj1426c).
absent(strainChickenicken13249,cj1426c).
absent(strainClinical58473,cj1426c).
absent(strainClinical43205,cj1426c).
absent(strainClinical33106,cj1426c).
absent(strainClinicall38556,cj1426c).
absent(strainClinicall39182,cj1426c).
absent(strainClinical44933,cj1426c).
absent(strainClinical45631,cj1426c).
absent(strainClinical41651,cj1426c).
absent(strainClinical32799,cj1426c).
absent(strainClinical43983,cj1426c).
absent(strainClinical40671,cj1426c).
absent(strainClinical44958,cj1426c).
absent(strainClinical52331,cj1426c).
absent(strainClinical56281,cj1426c).
absent(strainClinical56282,cj1426c).
absent(strainClinical56832,cj1426c).
absent(strainOvine12241,cj1426c).
absent(strainOvine12481,cj1426c).

absent(strainBovinevineine13305,cj1426c).
absent(strainChickenicken12912,cj1426c).
absent(strainChickenicken11818,cj1426c).
absent(strainChicken12196,cj1426c).
absent(strainChickenicken13040,cj1426c).
absent(strainChickenicken11856,cj1426c).
absent(strainClinical36069,cj1426c).
absent(strainChicken40209,cj1426c).
absent(strainBeach1793,cj1426c).
absent(strainClinical81116,cj1426c).
absent(strainClinical36439,cj1426c).
absent(strainClinical56519,cj1426c).
absent(strainClinical32787,cj1426c).
absent(strainClinicall31467,cj1426c).
absent(strainClinical44119,cj1426c).
absent(strainClinical34007,cj1426c).
absent(strainClinical38762,cj1426c).
absent(strainClinicalinical15168,cj1426c).
absent(strainClinical18836,cj1426c).
absent(strainClinicalM1,cj1426c).
absent(strainClinical36860,cj1426c).
absent(strainClinical40917,cj1426c).
absent(strainClinical38857,cj1426c).
absent(strainChicken47693,cj1426c).
absent(strainClinical63326,cj1426c).
absent(strainClinical64555,cj1426c).
absent(strainClinical59364,cj1426c).
absent(strainClinical59424,cj1426c).
absent(strainClinical33084,cj1426c).
absent(strainClinical31481,cj1426c).
absent(strainClinical39828,cj1426c).
absent(strainBeach1771,cj1426c).
absent(strainClinical53250,cj1426c).
absent(strainClinical45557,cj1426c).
absent(strainClinical47939,cj1426c).
absent(strainClinicalG1,cj1427c).
absent(strainClinicalG3,cj1427c).
absent(strainChickenicken11974,cj1427c).
absent(strainChickenicken13249,cj1427c).
absent(strainClinical58473,cj1427c).
absent(strainClinicall38556,cj1427c).
absent(strainClinicall39182,cj1427c).
absent(strainClinical44933,cj1427c).
absent(strainClinical45631,cj1427c).
absent(strainClinical55320,cj1427c).
absent(strainClinical41651,cj1427c).
absent(strainClinical32799,cj1427c).
absent(strainClinical44958,cj1427c).
absent(strainClinical52331,cj1427c).
absent(strainClinical56282,cj1427c).
absent(strainClinical56832,cj1427c).
absent(strainChickenicken12912,cj1427c).
absent(strainChickenicken11818,cj1427c).
absent(strainChicken12196,cj1427c).

absent(strainChickenicken13040,cj1427c).
absent(strainChickenicken11856,cj1427c).
absent(strainClinical36069,cj1427c).
absent(strainChicken40209,cj1427c).
absent(strainBeach1793,cj1427c).
absent(strainClinical81116,cj1427c).
absent(strainClinical36439,cj1427c).
absent(strainClinical56519,cj1427c).
absent(strainClinical32787,cj1427c).
absent(strainClinicall31467,cj1427c).
absent(strainClinical44119,cj1427c).
absent(strainClinical34007,cj1427c).
absent(strainClinical38762,cj1427c).
absent(strainClinicalinical15168,cj1427c).
absent(strainClinical18836,cj1427c).
absent(strainClinicalM1,cj1427c).
absent(strainClinical36860,cj1427c).
absent(strainClinical40917,cj1427c).
absent(strainClinical38857,cj1427c).
absent(strainCjejuniRM1221,cj1427c).
absent(strainChicken47693,cj1427c).
absent(strainClinical63326,cj1427c).
absent(strainClinical64555,cj1427c).
absent(strainClinical59364,cj1427c).
absent(strainClinical59424,cj1427c).
absent(strainClinical31481,cj1427c).
absent(strainClinical39828,cj1427c).
absent(strainBeach1771,cj1427c).
absent(strainClinical53250,cj1427c).
absent(strainClinical45557,cj1427c).
absent(strainClinical47939,cj1427c).
absent(strainClinicalG1,cj1428c).
absent(strainClinicalG3,cj1428c).
absent(strainChickenicken11974,cj1428c).
absent(strainChickenicken13249,cj1428c).
absent(strainClinical58473,cj1428c).
absent(strainClinical43205,cj1428c).
absent(strainClinical33106,cj1428c).
absent(strainClinicall38556,cj1428c).
absent(strainClinicall39182,cj1428c).
absent(strainClinical44933,cj1428c).
absent(strainClinical45631,cj1428c).
absent(strainClinical41651,cj1428c).
absent(strainClinical32799,cj1428c).
absent(strainClinical43983,cj1428c).
absent(strainClinical40671,cj1428c).
absent(strainClinical44958,cj1428c).
absent(strainClinical52331,cj1428c).
absent(strainClinical56281,cj1428c).
absent(strainClinical56282,cj1428c).
absent(strainClinical56832,cj1428c).
absent(strainOvine12241,cj1428c).
absent(strainOvine12481,cj1428c).
absent(strainBovinevineine13305,cj1428c).

absent(strainChickenicken12912,cj1428c).
absent(strainChickenicken11818,cj1428c).
absent(strainChicken12196,cj1428c).
absent(strainChickenicken13040,cj1428c).
absent(strainChickenicken11856,cj1428c).
absent(strainClinical36069,cj1428c).
absent(strainChicken40209,cj1428c).
absent(strainClinical36439,cj1428c).
absent(strainClinical56519,cj1428c).
absent(strainClinical32787,cj1428c).
absent(strainClinicall31467,cj1428c).
absent(strainClinical44119,cj1428c).
absent(strainClinical34007,cj1428c).
absent(strainClinical38762,cj1428c).
absent(strainClinicalinical15168,cj1428c).
absent(strainClinical18836,cj1428c).
absent(strainClinicalM1,cj1428c).
absent(strainClinical36860,cj1428c).
absent(strainClinical38857,cj1428c).
absent(strainCjejuniRM1221,cj1428c).
absent(strainChicken47693,cj1428c).
absent(strainClinical63326,cj1428c).
absent(strainClinical64555,cj1428c).
absent(strainClinical59364,cj1428c).
absent(strainClinical59424,cj1428c).
absent(strainClinical33084,cj1428c).
absent(strainClinical31481,cj1428c).
absent(strainClinical39828,cj1428c).
absent(strainBeach1771,cj1428c).
absent(strainClinical53250,cj1428c).
absent(strainClinical45557,cj1428c).
absent(strainClinical47939,cj1428c).
absent(strainClinicalG1,cj1429c).
absent(strainClinicalG3,cj1429c).
absent(strainChickenicken11974,cj1429c).
absent(strainChickenicken13249,cj1429c).
absent(strainClinical58473,cj1429c).
absent(strainClinical43205,cj1429c).
absent(strainClinical33106,cj1429c).
absent(strainClinicall38556,cj1429c).
absent(strainClinicall39182,cj1429c).
absent(strainClinical44933,cj1429c).
absent(strainClinical45631,cj1429c).
absent(strainClinical55320,cj1429c).
absent(strainClinical41651,cj1429c).
absent(strainClinical32799,cj1429c).
absent(strainClinical43983,cj1429c).
absent(strainClinical40671,cj1429c).
absent(strainClinical44958,cj1429c).
absent(strainClinical52331,cj1429c).
absent(strainClinical56281,cj1429c).
absent(strainClinical56282,cj1429c).
absent(strainClinical56832,cj1429c).
absent(strainOvine12241,cj1429c).

absent(strainOvine12481,cj1429c).
absent(strainBovinevineine13305,cj1429c).
absent(strainChickenicken12912,cj1429c).
absent(strainChickenicken11818,cj1429c).
absent(strainChicken12196,cj1429c).
absent(strainChickenicken13040,cj1429c).
absent(strainChickenicken11856,cj1429c).
absent(strainClinical36069,cj1429c).
absent(strainChicken40209,cj1429c).
absent(strainClinical81116,cj1429c).
absent(strainClinical36439,cj1429c).
absent(strainClinical56519,cj1429c).
absent(strainClinical32787,cj1429c).
absent(strainClinicall31467,cj1429c).
absent(strainClinical44119,cj1429c).
absent(strainClinical34007,cj1429c).
absent(strainClinical38762,cj1429c).
absent(strainClinicalinical15168,cj1429c).
absent(strainClinical18836,cj1429c).
absent(strainClinicalM1,cj1429c).
absent(strainClinical36860,cj1429c).
absent(strainClinical40917,cj1429c).
absent(strainCjejuniRM1221,cj1429c).
absent(strainChicken47693,cj1429c).
absent(strainClinical63326,cj1429c).
absent(strainClinical64555,cj1429c).
absent(strainClinical59364,cj1429c).
absent(strainClinical59424,cj1429c).
absent(strainClinical33084,cj1429c).
absent(strainClinical31481,cj1429c).
absent(strainClinical39828,cj1429c).
absent(strainBeach1771,cj1429c).
absent(strainClinical53250,cj1429c).
absent(strainClinical45557,cj1429c).
absent(strainClinical47939,cj1429c).
absent(strainClinicalG1,cj1430c).
absent(strainClinicalG3,cj1430c).
absent(strainChickenicken11974,cj1430c).
absent(strainChickenicken13249,cj1430c).
absent(strainClinical58473,cj1430c).
absent(strainClinical43205,cj1430c).
absent(strainClinical33106,cj1430c).
absent(strainClinicall38556,cj1430c).
absent(strainClinicall39182,cj1430c).
absent(strainClinical44933,cj1430c).
absent(strainClinical45631,cj1430c).
absent(strainClinical41651,cj1430c).
absent(strainClinical32799,cj1430c).
absent(strainClinical43983,cj1430c).
absent(strainClinical40671,cj1430c).
absent(strainClinical44958,cj1430c).
absent(strainClinical52331,cj1430c).
absent(strainClinical56281,cj1430c).
absent(strainClinical56282,cj1430c).

absent(strainClinical56832,cj1430c).
absent(strainOvine12241,cj1430c).
absent(strainOvine12481,cj1430c).
absent(strainBovinevineine13305,cj1430c).
absent(strainChickenicken12912,cj1430c).
absent(strainChickenicken11818,cj1430c).
absent(strainChicken12196,cj1430c).
absent(strainChickenicken13040,cj1430c).
absent(strainChickenicken11856,cj1430c).
absent(strainClinical36069,cj1430c).
absent(strainChicken40209,cj1430c).
absent(strainClinical81116,cj1430c).
absent(strainClinical36439,cj1430c).
absent(strainClinical56519,cj1430c).
absent(strainClinical32787,cj1430c).
absent(strainClinicall31467,cj1430c).
absent(strainClinical44119,cj1430c).
absent(strainClinical34007,cj1430c).
absent(strainClinical38762,cj1430c).
absent(strainClinicalinical15168,cj1430c).
absent(strainClinical18836,cj1430c).
absent(strainClinicalM1,cj1430c).
absent(strainClinical36860,cj1430c).
absent(strainClinical40917,cj1430c).
absent(strainClinical38857,cj1430c).
absent(strainCjejuniRM1221,cj1430c).
absent(strainChicken47693,cj1430c).
absent(strainClinical63326,cj1430c).
absent(strainClinical64555,cj1430c).
absent(strainClinical59364,cj1430c).
absent(strainClinical59424,cj1430c).
absent(strainClinical33084,cj1430c).
absent(strainClinical31481,cj1430c).
absent(strainClinical39828,cj1430c).
absent(strainBeach1771,cj1430c).
absent(strainClinical53250,cj1430c).
absent(strainClinical45557,cj1430c).
absent(strainClinical47939,cj1430c).
absent(strainClinicalG1,cj1431c).
absent(strainClinicalG3,cj1431c).
absent(strainChickenicken11974,cj1431c).
absent(strainChickenicken13249,cj1431c).
absent(strainClinical58473,cj1431c).
absent(strainClinical43205,cj1431c).
absent(strainClinical33106,cj1431c).
absent(strainClinicall38556,cj1431c).
absent(strainClinicall39182,cj1431c).
absent(strainClinical44933,cj1431c).
absent(strainClinical45631,cj1431c).
absent(strainClinical55320,cj1431c).
absent(strainClinical41651,cj1431c).
absent(strainClinical32799,cj1431c).
absent(strainClinical43983,cj1431c).
absent(strainClinical40671,cj1431c).

absent(strainClinical44958,cj1431c).
absent(strainClinical52331,cj1431c).
absent(strainClinical56281,cj1431c).
absent(strainClinical56282,cj1431c).
absent(strainClinical56832,cj1431c).
absent(strainOvine12241,cj1431c).
absent(strainOvine12481,cj1431c).
absent(strainBovinevineine13305,cj1431c).
absent(strainChickenicken12912,cj1431c).
absent(strainChickenicken11818,cj1431c).
absent(strainChicken12196,cj1431c).
absent(strainChickenicken13040,cj1431c).
absent(strainChickenicken11856,cj1431c).
absent(strainClinical36069,cj1431c).
absent(strainBeach1793,cj1431c).
absent(strainClinical81116,cj1431c).
absent(strainClinical36439,cj1431c).
absent(strainClinical56519,cj1431c).
absent(strainClinical32787,cj1431c).
absent(strainClinicall31467,cj1431c).
absent(strainClinical44119,cj1431c).
absent(strainClinical34007,cj1431c).
absent(strainClinical38762,cj1431c).
absent(strainClinicalinical15168,cj1431c).
absent(strainClinical18836,cj1431c).
absent(strainClinicalM1,cj1431c).
absent(strainClinical36860,cj1431c).
absent(strainClinical40917,cj1431c).
absent(strainClinical38857,cj1431c).
absent(strainCjejuniRM1221,cj1431c).
absent(strainChicken47693,cj1431c).
absent(strainClinical63326,cj1431c).
absent(strainClinical64555,cj1431c).
absent(strainClinical59364,cj1431c).
absent(strainClinical59424,cj1431c).
absent(strainClinical33084,cj1431c).
absent(strainClinical31481,cj1431c).
absent(strainClinical39828,cj1431c).
absent(strainBeach1771,cj1431c).
absent(strainClinical53250,cj1431c).
absent(strainClinical45557,cj1431c).
absent(strainClinical47939,cj1431c).
absent(strainClinicalG1,cj1432c).
absent(strainClinicalG3,cj1432c).
absent(strainChickenicken11974,cj1432c).
absent(strainChickenicken13249,cj1432c).
absent(strainClinical58473,cj1432c).
absent(strainClinical43205,cj1432c).
absent(strainClinical33106,cj1432c).
absent(strainClinicall38556,cj1432c).
absent(strainClinicall39182,cj1432c).
absent(strainClinical44933,cj1432c).
absent(strainClinical45631,cj1432c).
absent(strainClinical41651,cj1432c).

absent(strainClinical32799,cj1432c).
absent(strainClinical43983,cj1432c).
absent(strainClinical44958,cj1432c).
absent(strainClinical52331,cj1432c).
absent(strainClinical56281,cj1432c).
absent(strainClinical56282,cj1432c).
absent(strainClinical56832,cj1432c).
absent(strainOvine12241,cj1432c).
absent(strainOvine12481,cj1432c).
absent(strainBovinevineine13305,cj1432c).
absent(strainChickenicken12912,cj1432c).
absent(strainChickenicken11818,cj1432c).
absent(strainChicken12196,cj1432c).
absent(strainChickenicken13040,cj1432c).
absent(strainChickenicken11856,cj1432c).
absent(strainClinical36069,cj1432c).
absent(strainChicken40209,cj1432c).
absent(strainClinical36439,cj1432c).
absent(strainClinical56519,cj1432c).
absent(strainClinical32787,cj1432c).
absent(strainClinicall31467,cj1432c).
absent(strainClinical44119,cj1432c).
absent(strainClinical34007,cj1432c).
absent(strainClinical38762,cj1432c).
absent(strainClinicalinical15168,cj1432c).
absent(strainClinical18836,cj1432c).
absent(strainClinicalM1,cj1432c).
absent(strainClinical36860,cj1432c).
absent(strainClinical40917,cj1432c).
absent(strainClinical38857,cj1432c).
absent(strainCjejuniRM1221,cj1432c).
absent(strainChicken47693,cj1432c).
absent(strainClinical63326,cj1432c).
absent(strainClinical64555,cj1432c).
absent(strainClinical59364,cj1432c).
absent(strainClinical59424,cj1432c).
absent(strainClinical33084,cj1432c).
absent(strainClinical31481,cj1432c).
absent(strainClinical39828,cj1432c).
absent(strainBeach1771,cj1432c).
absent(strainClinical53250,cj1432c).
absent(strainClinical45557,cj1432c).
absent(strainClinical47939,cj1432c).
absent(strainClinicalG1,cj1433c).
absent(strainClinicalG3,cj1433c).
absent(strainChickenicken11974,cj1433c).
absent(strainChickenicken13249,cj1433c).
absent(strainClinical58473,cj1433c).
absent(strainClinical43205,cj1433c).
absent(strainClinical33106,cj1433c).
absent(strainClinicall38556,cj1433c).
absent(strainClinicall39182,cj1433c).
absent(strainClinical44933,cj1433c).
absent(strainClinical45631,cj1433c).

absent(strainClinical41651,cj1433c).
absent(strainClinical32799,cj1433c).
absent(strainClinical44958,cj1433c).
absent(strainClinical52331,cj1433c).
absent(strainClinical56281,cj1433c).
absent(strainClinical56282,cj1433c).
absent(strainClinical56832,cj1433c).
absent(strainOvine12241,cj1433c).
absent(strainOvine12481,cj1433c).
absent(strainBovinevineine13305,cj1433c).
absent(strainChickenicken12912,cj1433c).
absent(strainChickenicken11818,cj1433c).
absent(strainChicken12196,cj1433c).
absent(strainChickenicken13040,cj1433c).
absent(strainChickenicken11856,cj1433c).
absent(strainClinical36069,cj1433c).
absent(strainChicken40209,cj1433c).
absent(strainBeach1793,cj1433c).
absent(strainClinical81116,cj1433c).
absent(strainClinical36439,cj1433c).
absent(strainClinical56519,cj1433c).
absent(strainClinical32787,cj1433c).
absent(strainClinicall31467,cj1433c).
absent(strainClinical44119,cj1433c).
absent(strainClinical34007,cj1433c).
absent(strainClinical38762,cj1433c).
absent(strainClinicalinical15168,cj1433c).
absent(strainClinical18836,cj1433c).
absent(strainClinicalM1,cj1433c).
absent(strainClinical36860,cj1433c).
absent(strainClinical40917,cj1433c).
absent(strainClinical38857,cj1433c).
absent(strainCjejuniRM1221,cj1433c).
absent(strainChicken47693,cj1433c).
absent(strainClinical63326,cj1433c).
absent(strainClinical64555,cj1433c).
absent(strainClinical59364,cj1433c).
absent(strainClinical59424,cj1433c).
absent(strainClinical33084,cj1433c).
absent(strainClinical31481,cj1433c).
absent(strainClinical39828,cj1433c).
absent(strainBeach1771,cj1433c).
absent(strainClinical53250,cj1433c).
absent(strainClinical45557,cj1433c).
absent(strainClinical47939,cj1433c).
absent(strainClinicalG1,cj1434c).
absent(strainClinicalG3,cj1434c).
absent(strainChickenicken13249,cj1434c).
absent(strainClinical58473,cj1434c).
absent(strainClinical43205,cj1434c).
absent(strainClinical33106,cj1434c).
absent(strainClinicall38556,cj1434c).
absent(strainClinicall39182,cj1434c).
absent(strainClinical44933,cj1434c).

absent(strainClinical45631,cj1434c).
absent(strainClinical55320,cj1434c).
absent(strainClinical41651,cj1434c).
absent(strainClinical32799,cj1434c).
absent(strainClinical43983,cj1434c).
absent(strainClinical40671,cj1434c).
absent(strainClinical44958,cj1434c).
absent(strainClinical52331,cj1434c).
absent(strainClinical56281,cj1434c).
absent(strainClinical56282,cj1434c).
absent(strainClinical56832,cj1434c).
absent(strainOvine12241,cj1434c).
absent(strainOvine12481,cj1434c).
absent(strainBovinevineine13305,cj1434c).
absent(strainChickenicken12912,cj1434c).
absent(strainChickenicken11818,cj1434c).
absent(strainChicken12196,cj1434c).
absent(strainChickenicken13040,cj1434c).
absent(strainChickenicken11856,cj1434c).
absent(strainClinical36069,cj1434c).
absent(strainChicken40209,cj1434c).
absent(strainBeach1793,cj1434c).
absent(strainClinical81116,cj1434c).
absent(strainClinical36439,cj1434c).
absent(strainClinical56519,cj1434c).
absent(strainClinical32787,cj1434c).
absent(strainClinicall31467,cj1434c).
absent(strainClinical44119,cj1434c).
absent(strainClinical34007,cj1434c).
absent(strainClinical38762,cj1434c).
absent(strainClinicalinical15168,cj1434c).
absent(strainClinical18836,cj1434c).
absent(strainClinicalM1,cj1434c).
absent(strainClinical36860,cj1434c).
absent(strainClinical40917,cj1434c).
absent(strainClinical38857,cj1434c).
absent(strainCjejuniRM1221,cj1434c).
absent(strainChicken47693,cj1434c).
absent(strainClinical63326,cj1434c).
absent(strainClinical64555,cj1434c).
absent(strainClinical59364,cj1434c).
absent(strainClinical59424,cj1434c).
absent(strainClinical33084,cj1434c).
absent(strainClinical31481,cj1434c).
absent(strainClinical39828,cj1434c).
absent(strainBeach1771,cj1434c).
absent(strainClinical53250,cj1434c).
absent(strainClinical45557,cj1434c).
absent(strainClinical47939,cj1434c).
absent(strainClinicalG1,cj1435c).
absent(strainClinicalG3,cj1435c).
absent(strainChickenicken11974,cj1435c).
absent(strainChickenicken13249,cj1435c).
absent(strainClinical58473,cj1435c).

absent(strainClinical43205,cj1435c).
absent(strainClinical33106,cj1435c).
absent(strainClinicall38556,cj1435c).
absent(strainClinicall39182,cj1435c).
absent(strainClinical45631,cj1435c).
absent(strainClinical55320,cj1435c).
absent(strainClinical41651,cj1435c).
absent(strainClinical32799,cj1435c).
absent(strainClinical40671,cj1435c).
absent(strainClinical44958,cj1435c).
absent(strainClinical52331,cj1435c).
absent(strainClinical56281,cj1435c).
absent(strainClinical56282,cj1435c).
absent(strainClinical56832,cj1435c).
absent(strainOvine12241,cj1435c).
absent(strainOvine12481,cj1435c).
absent(strainBovinevineine13305,cj1435c).
absent(strainChickenicken12912,cj1435c).
absent(strainChickenicken11818,cj1435c).
absent(strainChicken12196,cj1435c).
absent(strainChickenicken13040,cj1435c).
absent(strainChickenicken11856,cj1435c).
absent(strainClinical36069,cj1435c).
absent(strainChicken40209,cj1435c).
absent(strainBeach1793,cj1435c).
absent(strainClinical81116,cj1435c).
absent(strainClinical56519,cj1435c).
absent(strainClinical32787,cj1435c).
absent(strainClinicall31467,cj1435c).
absent(strainClinical44119,cj1435c).
absent(strainClinical34007,cj1435c).
absent(strainClinical38762,cj1435c).
absent(strainClinical18836,cj1435c).
absent(strainClinicalM1,cj1435c).
absent(strainClinical36860,cj1435c).
absent(strainClinical40917,cj1435c).
absent(strainClinical38857,cj1435c).
absent(strainCjejuniRM1221,cj1435c).
absent(strainChicken47693,cj1435c).
absent(strainClinical63326,cj1435c).
absent(strainClinical64555,cj1435c).
absent(strainClinical59364,cj1435c).
absent(strainClinical59424,cj1435c).
absent(strainClinical33084,cj1435c).
absent(strainClinical31481,cj1435c).
absent(strainClinical39828,cj1435c).
absent(strainBeach1771,cj1435c).
absent(strainClinical53250,cj1435c).
absent(strainClinical45557,cj1435c).
absent(strainClinical47939,cj1435c).
absent(strainClinicalG1,cj1436c).
absent(strainClinicalG3,cj1436c).
absent(strainChickenicken11974,cj1436c).
absent(strainChickenicken13249,cj1436c).

absent(strainClinical58473,cj1436c).
absent(strainClinical43205,cj1436c).
absent(strainClinical33106,cj1436c).
absent(strainClinicall38556,cj1436c).
absent(strainClinicall39182,cj1436c).
absent(strainClinical44933,cj1436c).
absent(strainClinical45631,cj1436c).
absent(strainClinical41651,cj1436c).
absent(strainClinical32799,cj1436c).
absent(strainClinical43983,cj1436c).
absent(strainClinical40671,cj1436c).
absent(strainClinical44958,cj1436c).
absent(strainClinical52331,cj1436c).
absent(strainClinical56281,cj1436c).
absent(strainClinical56282,cj1436c).
absent(strainClinical56832,cj1436c).
absent(strainOvine12241,cj1436c).
absent(strainOvine12481,cj1436c).
absent(strainBovinevineine13305,cj1436c).
absent(strainChickenicken12912,cj1436c).
absent(strainChickenicken11818,cj1436c).
absent(strainChicken12196,cj1436c).
absent(strainChickenicken13040,cj1436c).
absent(strainChickenicken11856,cj1436c).
absent(strainClinical36069,cj1436c).
absent(strainChicken40209,cj1436c).
absent(strainClinical81116,cj1436c).
absent(strainClinical36439,cj1436c).
absent(strainClinical56519,cj1436c).
absent(strainClinical32787,cj1436c).
absent(strainClinicall31467,cj1436c).
absent(strainClinical44119,cj1436c).
absent(strainClinical34007,cj1436c).
absent(strainClinical38762,cj1436c).
absent(strainClinicalinical15168,cj1436c).
absent(strainClinical18836,cj1436c).
absent(strainClinicalM1,cj1436c).
absent(strainClinical36860,cj1436c).
absent(strainClinical40917,cj1436c).
absent(strainCjejuniRM1221,cj1436c).
absent(strainChicken47693,cj1436c).
absent(strainClinical63326,cj1436c).
absent(strainClinical64555,cj1436c).
absent(strainClinical59364,cj1436c).
absent(strainClinical59424,cj1436c).
absent(strainClinical33084,cj1436c).
absent(strainClinical31481,cj1436c).
absent(strainClinical39828,cj1436c).
absent(strainBeach1771,cj1436c).
absent(strainClinical53250,cj1436c).
absent(strainClinical45557,cj1436c).
absent(strainClinical47939,cj1436c).
absent(strainClinicalG1,cj1437c).
absent(strainClinicalG3,cj1437c).

absent(strainChickenicken11974,cj1437c).
absent(strainChickenicken13249,cj1437c).
absent(strainClinical58473,cj1437c).
absent(strainClinical43205,cj1437c).
absent(strainClinical33106,cj1437c).
absent(strainClinicall38556,cj1437c).
absent(strainClinicall39182,cj1437c).
absent(strainClinical44933,cj1437c).
absent(strainClinical45631,cj1437c).
absent(strainClinical55320,cj1437c).
absent(strainClinical41651,cj1437c).
absent(strainClinical32799,cj1437c).
absent(strainClinical43983,cj1437c).
absent(strainClinical40671,cj1437c).
absent(strainClinical44958,cj1437c).
absent(strainClinical52331,cj1437c).
absent(strainClinical56281,cj1437c).
absent(strainClinical56282,cj1437c).
absent(strainClinical56832,cj1437c).
absent(strainOvine12241,cj1437c).
absent(strainOvine12481,cj1437c).
absent(strainBovinevineine13305,cj1437c).
absent(strainChickenicken12912,cj1437c).
absent(strainChickenicken11818,cj1437c).
absent(strainChicken12196,cj1437c).
absent(strainChickenicken13040,cj1437c).
absent(strainChickenicken11856,cj1437c).
absent(strainClinical36069,cj1437c).
absent(strainChicken40209,cj1437c).
absent(strainBeach1793,cj1437c).
absent(strainClinical81116,cj1437c).
absent(strainClinical36439,cj1437c).
absent(strainClinical56519,cj1437c).
absent(strainClinical32787,cj1437c).
absent(strainClinicall31467,cj1437c).
absent(strainClinical44119,cj1437c).
absent(strainClinical34007,cj1437c).
absent(strainClinical38762,cj1437c).
absent(strainClinicalinical15168,cj1437c).
absent(strainClinical18836,cj1437c).
absent(strainClinicalM1,cj1437c).
absent(strainClinical36860,cj1437c).
absent(strainClinical40917,cj1437c).
absent(strainClinical38857,cj1437c).
absent(strainCjejuniRM1221,cj1437c).
absent(strainChicken47693,cj1437c).
absent(strainClinical63326,cj1437c).
absent(strainClinical64555,cj1437c).
absent(strainClinical59364,cj1437c).
absent(strainClinical59424,cj1437c).
absent(strainClinical33084,cj1437c).
absent(strainClinical31481,cj1437c).
absent(strainClinical39828,cj1437c).
absent(strainBeach1771,cj1437c).

absent(strainClinical53250,cj1437c).
absent(strainClinical45557,cj1437c).
absent(strainClinical47939,cj1437c).
absent(strainClinicalG1,cj1438c).
absent(strainClinicalG3,cj1438c).
absent(strainChickenicken11974,cj1438c).
absent(strainChickenicken13249,cj1438c).
absent(strainClinical58473,cj1438c).
absent(strainClinical43205,cj1438c).
absent(strainClinical33106,cj1438c).
absent(strainClinicall38556,cj1438c).
absent(strainClinicall39182,cj1438c).
absent(strainClinical44933,cj1438c).
absent(strainClinical45631,cj1438c).
absent(strainClinical55320,cj1438c).
absent(strainClinical41651,cj1438c).
absent(strainClinical32799,cj1438c).
absent(strainClinical43983,cj1438c).
absent(strainClinical40671,cj1438c).
absent(strainClinical44958,cj1438c).
absent(strainClinical52331,cj1438c).
absent(strainClinical56281,cj1438c).
absent(strainClinical56282,cj1438c).
absent(strainClinical56832,cj1438c).
absent(strainOvine12241,cj1438c).
absent(strainOvine12481,cj1438c).
absent(strainBovinevineine13305,cj1438c).
absent(strainChickenicken12912,cj1438c).
absent(strainChickenicken11818,cj1438c).
absent(strainChicken12196,cj1438c).
absent(strainChickenicken13040,cj1438c).
absent(strainChickenicken11856,cj1438c).
absent(strainClinical36069,cj1438c).
absent(strainChicken40209,cj1438c).
absent(strainBeach1793,cj1438c).
absent(strainClinical81116,cj1438c).
absent(strainClinical36439,cj1438c).
absent(strainClinical56519,cj1438c).
absent(strainClinical32787,cj1438c).
absent(strainClinicall31467,cj1438c).
absent(strainClinical44119,cj1438c).
absent(strainClinical34007,cj1438c).
absent(strainClinical38762,cj1438c).
absent(strainClinicalinical15168,cj1438c).
absent(strainClinical18836,cj1438c).
absent(strainClinicalM1,cj1438c).
absent(strainClinical36860,cj1438c).
absent(strainClinical40917,cj1438c).
absent(strainClinical38857,cj1438c).
absent(strainCjejuniRM1221,cj1438c).
absent(strainChicken47693,cj1438c).
absent(strainClinical63326,cj1438c).
absent(strainClinical64555,cj1438c).
absent(strainClinical59364,cj1438c).

absent(strainClinical59424,cj1438c).
absent(strainClinical33084,cj1438c).
absent(strainClinical31481,cj1438c).
absent(strainClinical39828,cj1438c).
absent(strainBeach1771,cj1438c).
absent(strainClinical53250,cj1438c).
absent(strainClinical45557,cj1438c).
absent(strainClinical47939,cj1438c).
absent(strainClinicalG3,cj1439c).
absent(strainChickenicken11974,cj1439c).
absent(strainChickenicken13249,cj1439c).
absent(strainClinical58473,cj1439c).
absent(strainClinical43205,cj1439c).
absent(strainClinical33106,cj1439c).
absent(strainClinicall38556,cj1439c).
absent(strainClinicall39182,cj1439c).
absent(strainClinical44933,cj1439c).
absent(strainClinical45631,cj1439c).
absent(strainClinical55320,cj1439c).
absent(strainClinical41651,cj1439c).
absent(strainClinical32799,cj1439c).
absent(strainClinical40671,cj1439c).
absent(strainClinical52331,cj1439c).
absent(strainClinical56281,cj1439c).
absent(strainClinical56282,cj1439c).
absent(strainClinical56832,cj1439c).
absent(strainOvine12241,cj1439c).
absent(strainOvine12481,cj1439c).
absent(strainBovinevineine13305,cj1439c).
absent(strainChickenicken12912,cj1439c).
absent(strainChickenicken11818,cj1439c).
absent(strainChicken12196,cj1439c).
absent(strainChickenicken13040,cj1439c).
absent(strainChickenicken11856,cj1439c).
absent(strainClinical36069,cj1439c).
absent(strainChicken40209,cj1439c).
absent(strainBeach1793,cj1439c).
absent(strainClinical81116,cj1439c).
absent(strainClinical56519,cj1439c).
absent(strainClinical32787,cj1439c).
absent(strainClinicall31467,cj1439c).
absent(strainClinical44119,cj1439c).
absent(strainClinical34007,cj1439c).
absent(strainClinical38762,cj1439c).
absent(strainClinicalinical15168,cj1439c).
absent(strainClinical18836,cj1439c).
absent(strainClinicalM1,cj1439c).
absent(strainClinical36860,cj1439c).
absent(strainClinical40917,cj1439c).
absent(strainClinical38857,cj1439c).
absent(strainCjejuniRM1221,cj1439c).
absent(strainChicken47693,cj1439c).
absent(strainClinical63326,cj1439c).
absent(strainClinical64555,cj1439c).

absent(strainClinical59364,cj1439c).
absent(strainClinical59424,cj1439c).
absent(strainClinical33084,cj1439c).
absent(strainClinical31481,cj1439c).
absent(strainClinical39828,cj1439c).
absent(strainBeach1771,cj1439c).
absent(strainClinical53250,cj1439c).
absent(strainClinical45557,cj1439c).
absent(strainClinical47939,cj1439c).
absent(strainClinicalG1,cj1440c).
absent(strainClinicalG3,cj1440c).
absent(strainChickenicken11974,cj1440c).
absent(strainChickenicken13249,cj1440c).
absent(strainClinical58473,cj1440c).
absent(strainClinical43205,cj1440c).
absent(strainClinical33106,cj1440c).
absent(strainClinicall38556,cj1440c).
absent(strainClinicall39182,cj1440c).
absent(strainClinical44933,cj1440c).
absent(strainClinical45631,cj1440c).
absent(strainClinical41651,cj1440c).
absent(strainClinical32799,cj1440c).
absent(strainClinical43983,cj1440c).
absent(strainClinical40671,cj1440c).
absent(strainClinical44958,cj1440c).
absent(strainClinical52331,cj1440c).
absent(strainClinical56282,cj1440c).
absent(strainClinical56832,cj1440c).
absent(strainOvine12241,cj1440c).
absent(strainOvine12481,cj1440c).
absent(strainBovinevineine13305,cj1440c).
absent(strainChickenicken12912,cj1440c).
absent(strainChickenicken11818,cj1440c).
absent(strainChicken12196,cj1440c).
absent(strainChickenicken13040,cj1440c).
absent(strainChickenicken11856,cj1440c).
absent(strainClinical36069,cj1440c).
absent(strainChicken40209,cj1440c).
absent(strainBeach1793,cj1440c).
absent(strainClinical81116,cj1440c).
absent(strainClinical36439,cj1440c).
absent(strainClinical56519,cj1440c).
absent(strainClinical32787,cj1440c).
absent(strainClinicall31467,cj1440c).
absent(strainClinical44119,cj1440c).
absent(strainClinical34007,cj1440c).
absent(strainClinical38762,cj1440c).
absent(strainClinicalinical15168,cj1440c).
absent(strainClinical18836,cj1440c).
absent(strainClinicalM1,cj1440c).
absent(strainClinical36860,cj1440c).
absent(strainClinical40917,cj1440c).
absent(strainClinical38857,cj1440c).
absent(strainCjejuniRM1221,cj1440c).

absent(strainChicken47693,cj1440c).
absent(strainClinical63326,cj1440c).
absent(strainClinical59364,cj1440c).
absent(strainClinical59424,cj1440c).
absent(strainClinical33084,cj1440c).
absent(strainClinical31481,cj1440c).
absent(strainClinical39828,cj1440c).
absent(strainBeach1771,cj1440c).
absent(strainClinical53250,cj1440c).
absent(strainClinical45557,cj1440c).
absent(strainClinical47939,cj1440c).
absent(strainClinicalG1,cj1441c).
absent(strainClinicalG3,cj1441c).
absent(strainChickenicken11974,cj1441c).
absent(strainChickenicken13249,cj1441c).
absent(strainClinical58473,cj1441c).
absent(strainClinical43205,cj1441c).
absent(strainClinical33106,cj1441c).
absent(strainClinicall38556,cj1441c).
absent(strainClinicall39182,cj1441c).
absent(strainClinical44933,cj1441c).
absent(strainClinical45631,cj1441c).
absent(strainClinical41651,cj1441c).
absent(strainClinical32799,cj1441c).
absent(strainClinical43983,cj1441c).
absent(strainClinical40671,cj1441c).
absent(strainClinical44958,cj1441c).
absent(strainClinical52331,cj1441c).
absent(strainClinical56282,cj1441c).
absent(strainClinical56832,cj1441c).
absent(strainOvine12241,cj1441c).
absent(strainOvine12481,cj1441c).
absent(strainBovinevineine13305,cj1441c).
absent(strainChickenicken12912,cj1441c).
absent(strainChickenicken11818,cj1441c).
absent(strainChicken12196,cj1441c).
absent(strainChickenicken13040,cj1441c).
absent(strainChickenicken11856,cj1441c).
absent(strainClinical36069,cj1441c).
absent(strainChicken40209,cj1441c).
absent(strainBeach1793,cj1441c).
absent(strainClinical81116,cj1441c).
absent(strainClinical36439,cj1441c).
absent(strainClinical56519,cj1441c).
absent(strainClinical32787,cj1441c).
absent(strainClinicall31467,cj1441c).
absent(strainClinical44119,cj1441c).
absent(strainClinical34007,cj1441c).
absent(strainClinical38762,cj1441c).
absent(strainClinicalinical15168,cj1441c).
absent(strainClinical18836,cj1441c).
absent(strainClinicalM1,cj1441c).
absent(strainClinical36860,cj1441c).
absent(strainClinical40917,cj1441c).

absent(strainClinical38857,cj1441c).
absent(strainCjejuniRM1221,cj1441c).
absent(strainChicken47693,cj1441c).
absent(strainClinical63326,cj1441c).
absent(strainClinical64555,cj1441c).
absent(strainClinical59364,cj1441c).
absent(strainClinical59424,cj1441c).
absent(strainClinical33084,cj1441c).
absent(strainClinical31481,cj1441c).
absent(strainClinical39828,cj1441c).
absent(strainBeach1771,cj1441c).
absent(strainClinical53250,cj1441c).
absent(strainClinical45557,cj1441c).
absent(strainClinical47939,cj1441c).
absent(strainClinicalG1,cj1442c).
absent(strainClinicalG3,cj1442c).
absent(strainChickenicken11974,cj1442c).
absent(strainChickenicken13249,cj1442c).
absent(strainClinical33106,cj1442c).
absent(strainClinicall38556,cj1442c).
absent(strainClinicall39182,cj1442c).
absent(strainClinical44933,cj1442c).
absent(strainClinical45631,cj1442c).
absent(strainClinical55320,cj1442c).
absent(strainClinical32799,cj1442c).
absent(strainClinical56832,cj1442c).
absent(strainChickenicken12912,cj1442c).
absent(strainClinical36069,cj1442c).
absent(strainChicken40209,cj1442c).
absent(strainBeach1793,cj1442c).
absent(strainClinical81116,cj1442c).
absent(strainClinical36439,cj1442c).
absent(strainClinical32787,cj1442c).
absent(strainClinicall31467,cj1442c).
absent(strainClinical44119,cj1442c).
absent(strainClinical34007,cj1442c).
absent(strainClinical38762,cj1442c).
absent(strainClinicalinical15168,cj1442c).
absent(strainClinical18836,cj1442c).
absent(strainClinicalM1,cj1442c).
absent(strainClinical36860,cj1442c).
absent(strainClinical40917,cj1442c).
absent(strainClinical38857,cj1442c).
absent(strainCjejuniRM1221,cj1442c).
absent(strainClinical63326,cj1442c).
absent(strainClinical64555,cj1442c).
absent(strainClinical59364,cj1442c).
absent(strainClinical59424,cj1442c).
absent(strainClinical33084,cj1442c).
absent(strainClinical31481,cj1442c).
absent(strainClinical39828,cj1442c).
absent(strainClinical45557,cj1442c).
absent(strainClinical47939,cj1442c).
absent(strainChickenicken12912,cj1443c).

absent(strainChickenicken11818,cj1443c).
absent(strainClinical36439,cj1443c).
absent(strainClinical32787,cj1443c).
absent(strainClinicall31467,cj1443c).
absent(strainClinical38762,cj1443c).
absent(strainCjejuniRM1221,cj1443c).
absent(strainClinicalG3,cj1445c).
absent(strainClinical32799,cj1445c).
absent(strainChickenicken11818,cj1445c).
absent(strainClinical39828,cj1445c).
absent(strainClinicalG1,cj1447c).
absent(strainClinicalG3,cj1447c).
absent(strainChickenicken11974,cj1447c).
absent(strainChickenicken13249,cj1447c).
absent(strainClinical33106,cj1447c).
absent(strainClinicall38556,cj1447c).
absent(strainClinicall39182,cj1447c).
absent(strainClinical44933,cj1447c).
absent(strainClinical45631,cj1447c).
absent(strainClinical55320,cj1447c).
absent(strainClinical41651,cj1447c).
absent(strainClinical32799,cj1447c).
absent(strainClinical43983,cj1447c).
absent(strainClinical40671,cj1447c).
absent(strainClinical44958,cj1447c).
absent(strainClinical52331,cj1447c).
absent(strainClinical56281,cj1447c).
absent(strainClinical56282,cj1447c).
absent(strainClinical56832,cj1447c).
absent(strainOvine12241,cj1447c).
absent(strainOvine12481,cj1447c).
absent(strainBovinevineine13305,cj1447c).
absent(strainChickenicken11818,cj1447c).
absent(strainChicken12196,cj1447c).
absent(strainChickenicken13040,cj1447c).
absent(strainChickenicken11856,cj1447c).
absent(strainClinical36069,cj1447c).
absent(strainBeach1793,cj1447c).
absent(strainClinical36439,cj1447c).
absent(strainClinical56519,cj1447c).
absent(strainClinical32787,cj1447c).
absent(strainClinicall31467,cj1447c).
absent(strainClinical44119,cj1447c).
absent(strainClinical34007,cj1447c).
absent(strainClinical38762,cj1447c).
absent(strainClinicalinical15168,cj1447c).
absent(strainClinical18836,cj1447c).
absent(strainClinicalM1,cj1447c).
absent(strainClinical36860,cj1447c).
absent(strainClinical40917,cj1447c).
absent(strainClinical38857,cj1447c).
absent(strainChicken47693,cj1447c).
absent(strainClinical63326,cj1447c).
absent(strainClinical64555,cj1447c).

absent(strainClinical59364,cj1447c).
absent(strainClinical59424,cj1447c).
absent(strainClinical33084,cj1447c).
absent(strainClinical31481,cj1447c).
absent(strainClinical39828,cj1447c).
absent(strainBeach1771,cj1447c).
absent(strainClinical53250,cj1447c).
absent(strainClinical45557,cj1447c).
absent(strainClinical47939,cj1447c).
absent(strainClinicalG1,cj1448c).
absent(strainClinicalG3,cj1448c).
absent(strainChickenicken11974,cj1448c).
absent(strainChickenicken13249,cj1448c).
absent(strainClinical33106,cj1448c).
absent(strainClinicall38556,cj1448c).
absent(strainClinicall39182,cj1448c).
absent(strainClinical44933,cj1448c).
absent(strainClinical45631,cj1448c).
absent(strainClinical41651,cj1448c).
absent(strainClinical32799,cj1448c).
absent(strainClinical43983,cj1448c).
absent(strainClinical40671,cj1448c).
absent(strainClinical44958,cj1448c).
absent(strainClinical52331,cj1448c).
absent(strainClinical56281,cj1448c).
absent(strainClinical56282,cj1448c).
absent(strainClinical56832,cj1448c).
absent(strainOvine12241,cj1448c).
absent(strainOvine12481,cj1448c).
absent(strainBovinevineine13305,cj1448c).
absent(strainChickenicken11818,cj1448c).
absent(strainChicken12196,cj1448c).
absent(strainChickenicken13040,cj1448c).
absent(strainChickenicken11856,cj1448c).
absent(strainClinical36069,cj1448c).
absent(strainClinical36439,cj1448c).
absent(strainClinical56519,cj1448c).
absent(strainClinical32787,cj1448c).
absent(strainClinicall31467,cj1448c).
absent(strainClinical44119,cj1448c).
absent(strainClinical34007,cj1448c).
absent(strainClinical38762,cj1448c).
absent(strainClinicalinical15168,cj1448c).
absent(strainClinical18836,cj1448c).
absent(strainClinicalM1,cj1448c).
absent(strainClinical40917,cj1448c).
absent(strainClinical38857,cj1448c).
absent(strainCjejuniRM1221,cj1448c).
absent(strainChicken47693,cj1448c).
absent(strainClinical63326,cj1448c).
absent(strainClinical64555,cj1448c).
absent(strainClinical59364,cj1448c).
absent(strainClinical59424,cj1448c).
absent(strainClinical33084,cj1448c).

absent(strainClinical31481,cj1448c).
absent(strainClinical39828,cj1448c).
absent(strainBeach1771,cj1448c).
absent(strainClinical45557,cj1448c).
absent(strainClinical47939,cj1448c).
absent0(strainClinicalG3,cj1414c).
absent0(strainClinical32799,cj1414c).
absent0(strainClinical43983,cj1414c).
absent0(strainBovinevineine13305,cj1414c).
absent0(strainChickenicken11818,cj1414c).
absent0(strainClinical81116,cj1414c).
absent0(strainClinical36439,cj1414c).
absent0(strainClinical32787,cj1414c).
absent0(strainClinicall31467,cj1414c).
absent0(strainClinical38762,cj1414c).
absent0(strainClinicalM1,cj1414c).
absent0(strainClinical36860,cj1414c).
absent0(strainClinical40917,cj1414c).
absent0(strainClinicall38556,cj1415c).
absent0(strainClinicall39182,cj1415c).
absent0(strainClinical44933,cj1415c).
absent0(strainClinical45631,cj1415c).
absent0(strainClinical55320,cj1415c).
absent0(strainClinical36069,cj1415c).
absent0(strainClinical81116,cj1415c).
absent0(strainClinical36439,cj1415c).
absent0(strainClinical56519,cj1415c).
absent0(strainClinical44119,cj1415c).
absent0(strainClinical34007,cj1415c).
absent0(strainClinicalM1,cj1415c).
absent0(strainClinical36860,cj1415c).
absent0(strainClinical40917,cj1415c).
absent0(strainChicken47693,cj1415c).
absent0(strainClinical63326,cj1415c).
absent0(strainClinical64555,cj1415c).
absent0(strainClinical59364,cj1415c).
absent0(strainClinical59424,cj1415c).
absent0(strainBeach1771,cj1415c).
absent0(strainClinical53250,cj1415c).
absent0(strainClinical47939,cj1415c).
absent0(strainClinicall38556,cj1416c).
absent0(strainClinicall39182,cj1416c).
absent0(strainClinical44933,cj1416c).
absent0(strainClinical45631,cj1416c).
absent0(strainClinical36069,cj1416c).
absent0(strainClinical36439,cj1416c).
absent0(strainClinical44119,cj1416c).
absent0(strainClinical34007,cj1416c).
absent0(strainCjejuniRM1221,cj1416c).
absent0(strainClinical63326,cj1416c).
absent0(strainClinical64555,cj1416c).
absent0(strainClinical59364,cj1416c).
absent0(strainClinical59424,cj1416c).
absent0(strainBeach1771,cj1416c).

absent0(strainClinical47939,cj1416c).
absent0(strainClinicall38556,cj1417c).
absent0(strainClinicall39182,cj1417c).
absent0(strainClinical44933,cj1417c).
absent0(strainClinical45631,cj1417c).
absent0(strainClinical55320,cj1417c).
absent0(strainClinical36069,cj1417c).
absent0(strainClinical36439,cj1417c).
absent0(strainClinical44119,cj1417c).
absent0(strainClinical34007,cj1417c).
absent0(strainCjejuniRM1221,cj1417c).
absent0(strainClinical63326,cj1417c).
absent0(strainClinical64555,cj1417c).
absent0(strainClinical59364,cj1417c).
absent0(strainClinical59424,cj1417c).
absent0(strainBeach1771,cj1417c).
absent0(strainClinical47939,cj1417c).
absent0(strainClinicall38556,cj1418c).
absent0(strainClinicall39182,cj1418c).
absent0(strainClinical44933,cj1418c).
absent0(strainClinical45631,cj1418c).
absent0(strainClinical55320,cj1418c).
absent0(strainClinical36069,cj1418c).
absent0(strainClinical36439,cj1418c).
absent0(strainClinical44119,cj1418c).
absent0(strainClinical34007,cj1418c).
absent0(strainCjejuniRM1221,cj1418c).
absent0(strainClinical63326,cj1418c).
absent0(strainClinical64555,cj1418c).
absent0(strainClinical59364,cj1418c).
absent0(strainClinical59424,cj1418c).
absent0(strainBeach1771,cj1418c).
absent0(strainClinical47939,cj1418c).
absent0(strainClinicall38556,cj1419c).
absent0(strainClinicall39182,cj1419c).
absent0(strainClinical44933,cj1419c).
absent0(strainClinical45631,cj1419c).
absent0(strainClinical55320,cj1419c).
absent0(strainClinical36069,cj1419c).
absent0(strainClinical36439,cj1419c).
absent0(strainClinical44119,cj1419c).
absent0(strainClinical34007,cj1419c).
absent0(strainCjejuniRM1221,cj1419c).
absent0(strainClinical63326,cj1419c).
absent0(strainClinical64555,cj1419c).
absent0(strainClinical59364,cj1419c).
absent0(strainClinical59424,cj1419c).
absent0(strainBeach1771,cj1419c).
absent0(strainClinical47939,cj1419c).
absent0(strainClinicalG1,cj1421c).
absent0(strainClinicalG3,cj1421c).
absent0(strainChickenicken11974,cj1421c).
absent0(strainChickenicken13249,cj1421c).
absent0(strainClinical58473,cj1421c).

absent0(strainClinical43205,cj1421c).
absent0(strainClinical33106,cj1421c).
absent0(strainClinicall38556,cj1421c).
absent0(strainClinicall39182,cj1421c).
absent0(strainClinical44933,cj1421c).
absent0(strainClinical45631,cj1421c).
absent0(strainClinical55320,cj1421c).
absent0(strainClinical41651,cj1421c).
absent0(strainClinical32799,cj1421c).
absent0(strainClinical43983,cj1421c).
absent0(strainClinical40671,cj1421c).
absent0(strainClinical44958,cj1421c).
absent0(strainClinical52331,cj1421c).
absent0(strainClinical56281,cj1421c).
absent0(strainClinical56282,cj1421c).
absent0(strainClinical56832,cj1421c).
absent0(strainOvine12241,cj1421c).
absent0(strainOvine12481,cj1421c).
absent0(strainBovinevineine13305,cj1421c).
absent0(strainChickenicken12912,cj1421c).
absent0(strainChickenicken11818,cj1421c).
absent0(strainChicken12196,cj1421c).
absent0(strainChickenicken13040,cj1421c).
absent0(strainChickenicken11856,cj1421c).
absent0(strainClinical36069,cj1421c).
absent0(strainChicken40209,cj1421c).
absent0(strainBeach1793,cj1421c).
absent0(strainClinical81116,cj1421c).
absent0(strainClinical36439,cj1421c).
absent0(strainClinical56519,cj1421c).
absent0(strainClinical32787,cj1421c).
absent0(strainClinicall31467,cj1421c).
absent0(strainClinical44119,cj1421c).
absent0(strainClinical34007,cj1421c).
absent0(strainClinical38762,cj1421c).
absent0(strainClinicalinical15168,cj1421c).
absent0(strainClinical18836,cj1421c).
absent0(strainClinicalM1,cj1421c).
absent0(strainClinical36860,cj1421c).
absent0(strainClinical40917,cj1421c).
absent0(strainClinical38857,cj1421c).
absent0(strainChicken47693,cj1421c).
absent0(strainClinical63326,cj1421c).
absent0(strainClinical64555,cj1421c).
absent0(strainClinical59364,cj1421c).
absent0(strainClinical59424,cj1421c).
absent0(strainClinical33084,cj1421c).
absent0(strainClinical31481,cj1421c).
absent0(strainClinical39828,cj1421c).
absent0(strainBeach1771,cj1421c).
absent0(strainClinical53250,cj1421c).
absent0(strainClinical45557,cj1421c).
absent0(strainClinical47939,cj1421c).
absent0(strainClinicalG1,cj1422c).

absent0(strainClinicalG3,cj1422c).
absent0(strainChickenicken11974,cj1422c).
absent0(strainChickenicken13249,cj1422c).
absent0(strainClinical58473,cj1422c).
absent0(strainClinical43205,cj1422c).
absent0(strainClinical33106,cj1422c).
absent0(strainClinicall38556,cj1422c).
absent0(strainClinicall39182,cj1422c).
absent0(strainClinical44933,cj1422c).
absent0(strainClinical45631,cj1422c).
absent0(strainClinical41651,cj1422c).
absent0(strainClinical32799,cj1422c).
absent0(strainClinical43983,cj1422c).
absent0(strainClinical40671,cj1422c).
absent0(strainClinical44958,cj1422c).
absent0(strainClinical52331,cj1422c).
absent0(strainClinical56281,cj1422c).
absent0(strainClinical56282,cj1422c).
absent0(strainClinical56832,cj1422c).
absent0(strainOvine12241,cj1422c).
absent0(strainOvine12481,cj1422c).
absent0(strainBovinevineine13305,cj1422c).
absent0(strainChickenicken12912,cj1422c).
absent0(strainChickenicken11818,cj1422c).
absent0(strainChicken12196,cj1422c).
absent0(strainChickenicken13040,cj1422c).
absent0(strainChickenicken11856,cj1422c).
absent0(strainClinical36069,cj1422c).
absent0(strainChicken40209,cj1422c).
absent0(strainBeach1793,cj1422c).
absent0(strainClinical81116,cj1422c).
absent0(strainClinical36439,cj1422c).
absent0(strainClinical56519,cj1422c).
absent0(strainClinical32787,cj1422c).
absent0(strainClinicall31467,cj1422c).
absent0(strainClinical44119,cj1422c).
absent0(strainClinical34007,cj1422c).
absent0(strainClinical38762,cj1422c).
absent0(strainClinicalinical15168,cj1422c).
absent0(strainClinical18836,cj1422c).
absent0(strainClinicalM1,cj1422c).
absent0(strainClinical36860,cj1422c).
absent0(strainClinical40917,cj1422c).
absent0(strainClinical38857,cj1422c).
absent0(strainCjejuniRM1221,cj1422c).
absent0(strainChicken47693,cj1422c).
absent0(strainClinical63326,cj1422c).
absent0(strainClinical64555,cj1422c).
absent0(strainClinical59364,cj1422c).
absent0(strainClinical59424,cj1422c).
absent0(strainClinical33084,cj1422c).
absent0(strainClinical31481,cj1422c).
absent0(strainBeach1771,cj1422c).
absent0(strainClinical53250,cj1422c).

absent0(strainClinical45557,cj1422c).
absent0(strainClinical47939,cj1422c).
absent0(strainClinicalG1,cj1423c).
absent0(strainClinicalG3,cj1423c).
absent0(strainChickenicken11974,cj1423c).
absent0(strainChickenicken13249,cj1423c).
absent0(strainClinical58473,cj1423c).
absent0(strainClinical33106,cj1423c).
absent0(strainClinicall38556,cj1423c).
absent0(strainClinicall39182,cj1423c).
absent0(strainClinical44933,cj1423c).
absent0(strainClinical45631,cj1423c).
absent0(strainClinical32799,cj1423c).
absent0(strainClinical43983,cj1423c).
absent0(strainClinical40671,cj1423c).
absent0(strainClinical52331,cj1423c).
absent0(strainClinical56832,cj1423c).
absent0(strainOvine12241,cj1423c).
absent0(strainOvine12481,cj1423c).
absent0(strainBovinevineine13305,cj1423c).
absent0(strainChickenicken12912,cj1423c).
absent0(strainChickenicken11818,cj1423c).
absent0(strainChicken12196,cj1423c).
absent0(strainChickenicken13040,cj1423c).
absent0(strainChickenicken11856,cj1423c).
absent0(strainClinical36069,cj1423c).
absent0(strainChicken40209,cj1423c).
absent0(strainClinical81116,cj1423c).
absent0(strainClinical36439,cj1423c).
absent0(strainClinical56519,cj1423c).
absent0(strainClinical32787,cj1423c).
absent0(strainClinicall31467,cj1423c).
absent0(strainClinical44119,cj1423c).
absent0(strainClinical34007,cj1423c).
absent0(strainClinical38762,cj1423c).
absent0(strainClinicalinical15168,cj1423c).
absent0(strainClinical18836,cj1423c).
absent0(strainClinicalM1,cj1423c).
absent0(strainClinical36860,cj1423c).
absent0(strainClinical40917,cj1423c).
absent0(strainClinical38857,cj1423c).
absent0(strainCjejuniRM1221,cj1423c).
absent0(strainChicken47693,cj1423c).
absent0(strainClinical63326,cj1423c).
absent0(strainClinical64555,cj1423c).
absent0(strainClinical59364,cj1423c).
absent0(strainClinical59424,cj1423c).
absent0(strainClinical31481,cj1423c).
absent0(strainClinical39828,cj1423c).
absent0(strainBeach1771,cj1423c).
absent0(strainClinical45557,cj1423c).
absent0(strainClinical47939,cj1423c).
absent0(strainClinicalG1,cj1424c).
absent0(strainClinicalG3,cj1424c).

absent0(strainChickenicken11974,cj1424c).
absent0(strainChickenicken13249,cj1424c).
absent0(strainClinical58473,cj1424c).
absent0(strainClinical33106,cj1424c).
absent0(strainClinical44933,cj1424c).
absent0(strainClinical32799,cj1424c).
absent0(strainClinical44958,cj1424c).
absent0(strainClinical52331,cj1424c).
absent0(strainClinical56281,cj1424c).
absent0(strainClinical56832,cj1424c).
absent0(strainChickenicken12912,cj1424c).
absent0(strainChickenicken11818,cj1424c).
absent0(strainChicken12196,cj1424c).
absent0(strainChickenicken13040,cj1424c).
absent0(strainChicken40209,cj1424c).
absent0(strainBeach1793,cj1424c).
absent0(strainClinical36439,cj1424c).
absent0(strainClinical56519,cj1424c).
absent0(strainClinicalinical15168,cj1424c).
absent0(strainClinical18836,cj1424c).
absent0(strainClinicalM1,cj1424c).
absent0(strainClinical36860,cj1424c).
absent0(strainClinical40917,cj1424c).
absent0(strainCjejuniRM1221,cj1424c).
absent0(strainClinical39828,cj1424c).
absent0(strainClinical45557,cj1424c).
absent0(strainClinical47939,cj1424c).
absent0(strainClinicalG1,cj1425c).
absent0(strainClinicalG3,cj1425c).
absent0(strainChickenicken11974,cj1425c).
absent0(strainChickenicken13249,cj1425c).
absent0(strainClinicall38556,cj1425c).
absent0(strainClinicall39182,cj1425c).
absent0(strainClinical45631,cj1425c).
absent0(strainClinical32799,cj1425c).
absent0(strainClinical52331,cj1425c).
absent0(strainOvine12481,cj1425c).
absent0(strainBovinevineine13305,cj1425c).
absent0(strainChickenicken12912,cj1425c).
absent0(strainChickenicken11818,cj1425c).
absent0(strainClinical36069,cj1425c).
absent0(strainChicken40209,cj1425c).
absent0(strainClinical36439,cj1425c).
absent0(strainClinical32787,cj1425c).
absent0(strainClinical44119,cj1425c).
absent0(strainClinical34007,cj1425c).
absent0(strainClinical38762,cj1425c).
absent0(strainClinicalinical15168,cj1425c).
absent0(strainClinical18836,cj1425c).
absent0(strainClinicalM1,cj1425c).
absent0(strainClinical36860,cj1425c).
absent0(strainClinical40917,cj1425c).
absent0(strainCjejuniRM1221,cj1425c).
absent0(strainClinical63326,cj1425c).

absent0(strainClinical59364,cj1425c).
absent0(strainClinical59424,cj1425c).
absent0(strainClinical33084,cj1425c).
absent0(strainClinical39828,cj1425c).
absent0(strainClinical45557,cj1425c).
absent0(strainClinical47939,cj1425c).
absent0(strainClinicalG1,cj1426c).
absent0(strainClinicalG3,cj1426c).
absent0(strainChickenicken11974,cj1426c).
absent0(strainChickenicken13249,cj1426c).
absent0(strainClinical58473,cj1426c).
absent0(strainClinical43205,cj1426c).
absent0(strainClinical33106,cj1426c).
absent0(strainClinicall38556,cj1426c).
absent0(strainClinicall39182,cj1426c).
absent0(strainClinical44933,cj1426c).
absent0(strainClinical45631,cj1426c).
absent0(strainClinical41651,cj1426c).
absent0(strainClinical32799,cj1426c).
absent0(strainClinical43983,cj1426c).
absent0(strainClinical40671,cj1426c).
absent0(strainClinical44958,cj1426c).
absent0(strainClinical52331,cj1426c).
absent0(strainClinical56281,cj1426c).
absent0(strainClinical56282,cj1426c).
absent0(strainClinical56832,cj1426c).
absent0(strainOvine12241,cj1426c).
absent0(strainOvine12481,cj1426c).
absent0(strainBovinevineine13305,cj1426c).
absent0(strainChickenicken12912,cj1426c).
absent0(strainChickenicken11818,cj1426c).
absent0(strainChicken12196,cj1426c).
absent0(strainChickenicken13040,cj1426c).
absent0(strainChickenicken11856,cj1426c).
absent0(strainClinical36069,cj1426c).
absent0(strainChicken40209,cj1426c).
absent0(strainBeach1793,cj1426c).
absent0(strainClinical81116,cj1426c).
absent0(strainClinical36439,cj1426c).
absent0(strainClinical56519,cj1426c).
absent0(strainClinical32787,cj1426c).
absent0(strainClinicall31467,cj1426c).
absent0(strainClinical44119,cj1426c).
absent0(strainClinical34007,cj1426c).
absent0(strainClinical38762,cj1426c).
absent0(strainClinicalinical15168,cj1426c).
absent0(strainClinical18836,cj1426c).
absent0(strainClinicalM1,cj1426c).
absent0(strainClinical36860,cj1426c).
absent0(strainClinical40917,cj1426c).
absent0(strainClinical38857,cj1426c).
absent0(strainChicken47693,cj1426c).
absent0(strainClinical63326,cj1426c).
absent0(strainClinical64555,cj1426c).

absent0(strainClinical59364,cj1426c).
absent0(strainClinical59424,cj1426c).
absent0(strainClinical33084,cj1426c).
absent0(strainClinical31481,cj1426c).
absent0(strainClinical39828,cj1426c).
absent0(strainBeach1771,cj1426c).
absent0(strainClinical53250,cj1426c).
absent0(strainClinical45557,cj1426c).
absent0(strainClinical47939,cj1426c).
absent0(strainClinicalG1,cj1427c).
absent0(strainClinicalG3,cj1427c).
absent0(strainChickenicken11974,cj1427c).
absent0(strainChickenicken13249,cj1427c).
absent0(strainClinical58473,cj1427c).
absent0(strainClinicall38556,cj1427c).
absent0(strainClinicall39182,cj1427c).
absent0(strainClinical44933,cj1427c).
absent0(strainClinical45631,cj1427c).
absent0(strainClinical55320,cj1427c).
absent0(strainClinical41651,cj1427c).
absent0(strainClinical32799,cj1427c).
absent0(strainClinical44958,cj1427c).
absent0(strainClinical52331,cj1427c).
absent0(strainClinical56282,cj1427c).
absent0(strainClinical56832,cj1427c).
absent0(strainChickenicken12912,cj1427c).
absent0(strainChickenicken11818,cj1427c).
absent0(strainChicken12196,cj1427c).
absent0(strainChickenicken13040,cj1427c).
absent0(strainChickenicken11856,cj1427c).
absent0(strainClinical36069,cj1427c).
absent0(strainChicken40209,cj1427c).
absent0(strainBeach1793,cj1427c).
absent0(strainClinical81116,cj1427c).
absent0(strainClinical36439,cj1427c).
absent0(strainClinical56519,cj1427c).
absent0(strainClinical32787,cj1427c).
absent0(strainClinicall31467,cj1427c).
absent0(strainClinical44119,cj1427c).
absent0(strainClinical34007,cj1427c).
absent0(strainClinical38762,cj1427c).
absent0(strainClinicalinical15168,cj1427c).
absent0(strainClinical18836,cj1427c).
absent0(strainClinicalM1,cj1427c).
absent0(strainClinical36860,cj1427c).
absent0(strainClinical40917,cj1427c).
absent0(strainClinical38857,cj1427c).
absent0(strainCjejuniRM1221,cj1427c).
absent0(strainChicken47693,cj1427c).
absent0(strainClinical63326,cj1427c).
absent0(strainClinical64555,cj1427c).
absent0(strainClinical59364,cj1427c).
absent0(strainClinical59424,cj1427c).
absent0(strainClinical31481,cj1427c).

absent0(strainClinical39828,cj1427c).
absent0(strainBeach1771,cj1427c).
absent0(strainClinical53250,cj1427c).
absent0(strainClinical45557,cj1427c).
absent0(strainClinical47939,cj1427c).
absent0(strainClinicalG1,cj1428c).
absent0(strainClinicalG3,cj1428c).
absent0(strainChickenicken11974,cj1428c).
absent0(strainChickenicken13249,cj1428c).
absent0(strainClinical58473,cj1428c).
absent0(strainClinical43205,cj1428c).
absent0(strainClinical33106,cj1428c).
absent0(strainClinicall38556,cj1428c).
absent0(strainClinicall39182,cj1428c).
absent0(strainClinical44933,cj1428c).
absent0(strainClinical45631,cj1428c).
absent0(strainClinical41651,cj1428c).
absent0(strainClinical32799,cj1428c).
absent0(strainClinical43983,cj1428c).
absent0(strainClinical40671,cj1428c).
absent0(strainClinical44958,cj1428c).
absent0(strainClinical52331,cj1428c).
absent0(strainClinical56281,cj1428c).
absent0(strainClinical56282,cj1428c).
absent0(strainClinical56832,cj1428c).
absent0(strainOvine12241,cj1428c).
absent0(strainOvine12481,cj1428c).
absent0(strainBovinevineine13305,cj1428c).
absent0(strainChickenicken12912,cj1428c).
absent0(strainChickenicken11818,cj1428c).
absent0(strainChicken12196,cj1428c).
absent0(strainChickenicken13040,cj1428c).
absent0(strainChickenicken11856,cj1428c).
absent0(strainClinical36069,cj1428c).
absent0(strainChicken40209,cj1428c).
absent0(strainClinical36439,cj1428c).
absent0(strainClinical56519,cj1428c).
absent0(strainClinical32787,cj1428c).
absent0(strainClinicall31467,cj1428c).
absent0(strainClinical44119,cj1428c).
absent0(strainClinical34007,cj1428c).
absent0(strainClinical38762,cj1428c).
absent0(strainClinicalinical15168,cj1428c).
absent0(strainClinical18836,cj1428c).
absent0(strainClinicalM1,cj1428c).
absent0(strainClinical36860,cj1428c).
absent0(strainClinical38857,cj1428c).
absent0(strainCjejuniRM1221,cj1428c).
absent0(strainChicken47693,cj1428c).
absent0(strainClinical63326,cj1428c).
absent0(strainClinical64555,cj1428c).
absent0(strainClinical59364,cj1428c).
absent0(strainClinical59424,cj1428c).
absent0(strainClinical33084,cj1428c).

absent0(strainClinical31481,cj1428c).
absent0(strainClinical39828,cj1428c).
absent0(strainBeach1771,cj1428c).
absent0(strainClinical53250,cj1428c).
absent0(strainClinical45557,cj1428c).
absent0(strainClinical47939,cj1428c).
absent0(strainClinicalG1,cj1429c).
absent0(strainClinicalG3,cj1429c).
absent0(strainChickenicken11974,cj1429c).
absent0(strainChickenicken13249,cj1429c).
absent0(strainClinical58473,cj1429c).
absent0(strainClinical43205,cj1429c).
absent0(strainClinical33106,cj1429c).
absent0(strainClinicall38556,cj1429c).
absent0(strainClinicall39182,cj1429c).
absent0(strainClinical44933,cj1429c).
absent0(strainClinical45631,cj1429c).
absent0(strainClinical55320,cj1429c).
absent0(strainClinical41651,cj1429c).
absent0(strainClinical32799,cj1429c).
absent0(strainClinical43983,cj1429c).
absent0(strainClinical40671,cj1429c).
absent0(strainClinical44958,cj1429c).
absent0(strainClinical52331,cj1429c).
absent0(strainClinical56281,cj1429c).
absent0(strainClinical56282,cj1429c).
absent0(strainClinical56832,cj1429c).
absent0(strainOvine12241,cj1429c).
absent0(strainOvine12481,cj1429c).
absent0(strainBovinevineine13305,cj1429c).
absent0(strainChickenicken12912,cj1429c).
absent0(strainChickenicken11818,cj1429c).
absent0(strainChicken12196,cj1429c).
absent0(strainChickenicken13040,cj1429c).
absent0(strainChickenicken11856,cj1429c).
absent0(strainClinical36069,cj1429c).
absent0(strainChicken40209,cj1429c).
absent0(strainClinical81116,cj1429c).
absent0(strainClinical36439,cj1429c).
absent0(strainClinical56519,cj1429c).
absent0(strainClinical32787,cj1429c).
absent0(strainClinicall31467,cj1429c).
absent0(strainClinical44119,cj1429c).
absent0(strainClinical34007,cj1429c).
absent0(strainClinical38762,cj1429c).
absent0(strainClinicalinical15168,cj1429c).
absent0(strainClinical18836,cj1429c).
absent0(strainClinicalM1,cj1429c).
absent0(strainClinical36860,cj1429c).
absent0(strainClinical40917,cj1429c).
absent0(strainCjejuniRM1221,cj1429c).
absent0(strainChicken47693,cj1429c).
absent0(strainClinical63326,cj1429c).
absent0(strainClinical64555,cj1429c).

absent0(strainClinical59364,cj1429c).
absent0(strainClinical59424,cj1429c).
absent0(strainClinical33084,cj1429c).
absent0(strainClinical31481,cj1429c).
absent0(strainClinical39828,cj1429c).
absent0(strainBeach1771,cj1429c).
absent0(strainClinical53250,cj1429c).
absent0(strainClinical45557,cj1429c).
absent0(strainClinical47939,cj1429c).
absent0(strainClinicalG1,cj1430c).
absent0(strainClinicalG3,cj1430c).
absent0(strainChickenicken11974,cj1430c).
absent0(strainChickenicken13249,cj1430c).
absent0(strainClinical58473,cj1430c).
absent0(strainClinical43205,cj1430c).
absent0(strainClinical33106,cj1430c).
absent0(strainClinicall38556,cj1430c).
absent0(strainClinicall39182,cj1430c).
absent0(strainClinical44933,cj1430c).
absent0(strainClinical45631,cj1430c).
absent0(strainClinical41651,cj1430c).
absent0(strainClinical32799,cj1430c).
absent0(strainClinical43983,cj1430c).
absent0(strainClinical40671,cj1430c).
absent0(strainClinical44958,cj1430c).
absent0(strainClinical52331,cj1430c).
absent0(strainClinical56281,cj1430c).
absent0(strainClinical56282,cj1430c).
absent0(strainClinical56832,cj1430c).
absent0(strainOvine12241,cj1430c).
absent0(strainOvine12481,cj1430c).
absent0(strainBovinevineine13305,cj1430c).
absent0(strainChickenicken12912,cj1430c).
absent0(strainChickenicken11818,cj1430c).
absent0(strainChicken12196,cj1430c).
absent0(strainChickenicken13040,cj1430c).
absent0(strainChickenicken11856,cj1430c).
absent0(strainClinical36069,cj1430c).
absent0(strainChicken40209,cj1430c).
absent0(strainClinical81116,cj1430c).
absent0(strainClinical36439,cj1430c).
absent0(strainClinical56519,cj1430c).
absent0(strainClinical32787,cj1430c).
absent0(strainClinicall31467,cj1430c).
absent0(strainClinical44119,cj1430c).
absent0(strainClinical34007,cj1430c).
absent0(strainClinical38762,cj1430c).
absent0(strainClinicalinical15168,cj1430c).
absent0(strainClinical18836,cj1430c).
absent0(strainClinicalM1,cj1430c).
absent0(strainClinical36860,cj1430c).
absent0(strainClinical40917,cj1430c).
absent0(strainClinical38857,cj1430c).
absent0(strainCjejuniRM1221,cj1430c).

absent0(strainChicken47693,cj1430c).
absent0(strainClinical63326,cj1430c).
absent0(strainClinical64555,cj1430c).
absent0(strainClinical59364,cj1430c).
absent0(strainClinical59424,cj1430c).
absent0(strainClinical33084,cj1430c).
absent0(strainClinical31481,cj1430c).
absent0(strainClinical39828,cj1430c).
absent0(strainBeach1771,cj1430c).
absent0(strainClinical53250,cj1430c).
absent0(strainClinical45557,cj1430c).
absent0(strainClinical47939,cj1430c).
absent0(strainClinicalG1,cj1431c).
absent0(strainClinicalG3,cj1431c).
absent0(strainChickenicken11974,cj1431c).
absent0(strainChickenicken13249,cj1431c).
absent0(strainClinical58473,cj1431c).
absent0(strainClinical43205,cj1431c).
absent0(strainClinical33106,cj1431c).
absent0(strainClinicall38556,cj1431c).
absent0(strainClinicall39182,cj1431c).
absent0(strainClinical44933,cj1431c).
absent0(strainClinical45631,cj1431c).
absent0(strainClinical55320,cj1431c).
absent0(strainClinical41651,cj1431c).
absent0(strainClinical32799,cj1431c).
absent0(strainClinical43983,cj1431c).
absent0(strainClinical40671,cj1431c).
absent0(strainClinical44958,cj1431c).
absent0(strainClinical52331,cj1431c).
absent0(strainClinical56281,cj1431c).
absent0(strainClinical56282,cj1431c).
absent0(strainClinical56832,cj1431c).
absent0(strainOvine12241,cj1431c).
absent0(strainOvine12481,cj1431c).
absent0(strainBovinevineine13305,cj1431c).
absent0(strainChickenicken12912,cj1431c).
absent0(strainChickenicken11818,cj1431c).
absent0(strainChicken12196,cj1431c).
absent0(strainChickenicken13040,cj1431c).
absent0(strainChickenicken11856,cj1431c).
absent0(strainClinical36069,cj1431c).
absent0(strainBeach1793,cj1431c).
absent0(strainClinical81116,cj1431c).
absent0(strainClinical36439,cj1431c).
absent0(strainClinical56519,cj1431c).
absent0(strainClinical32787,cj1431c).
absent0(strainClinicall31467,cj1431c).
absent0(strainClinical44119,cj1431c).
absent0(strainClinical34007,cj1431c).
absent0(strainClinical38762,cj1431c).
absent0(strainClinicalinical15168,cj1431c).
absent0(strainClinical18836,cj1431c).
absent0(strainClinicalM1,cj1431c).

absent0(strainClinical36860,cj1431c).
absent0(strainClinical40917,cj1431c).
absent0(strainClinical38857,cj1431c).
absent0(strainCjejuniRM1221,cj1431c).
absent0(strainChicken47693,cj1431c).
absent0(strainClinical63326,cj1431c).
absent0(strainClinical64555,cj1431c).
absent0(strainClinical59364,cj1431c).
absent0(strainClinical59424,cj1431c).
absent0(strainClinical33084,cj1431c).
absent0(strainClinical31481,cj1431c).
absent0(strainClinical39828,cj1431c).
absent0(strainBeach1771,cj1431c).
absent0(strainClinical53250,cj1431c).
absent0(strainClinical45557,cj1431c).
absent0(strainClinical47939,cj1431c).
absent0(strainClinicalG1,cj1432c).
absent0(strainClinicalG3,cj1432c).
absent0(strainChickenicken11974,cj1432c).
absent0(strainChickenicken13249,cj1432c).
absent0(strainClinical58473,cj1432c).
absent0(strainClinical43205,cj1432c).
absent0(strainClinical33106,cj1432c).
absent0(strainClinicall38556,cj1432c).
absent0(strainClinicall39182,cj1432c).
absent0(strainClinical44933,cj1432c).
absent0(strainClinical45631,cj1432c).
absent0(strainClinical41651,cj1432c).
absent0(strainClinical32799,cj1432c).
absent0(strainClinical43983,cj1432c).
absent0(strainClinical44958,cj1432c).
absent0(strainClinical52331,cj1432c).
absent0(strainClinical56281,cj1432c).
absent0(strainClinical56282,cj1432c).
absent0(strainClinical56832,cj1432c).
absent0(strainOvine12241,cj1432c).
absent0(strainOvine12481,cj1432c).
absent0(strainBovinevineine13305,cj1432c).
absent0(strainChickenicken12912,cj1432c).
absent0(strainChickenicken11818,cj1432c).
absent0(strainChicken12196,cj1432c).
absent0(strainChickenicken13040,cj1432c).
absent0(strainChickenicken11856,cj1432c).
absent0(strainClinical36069,cj1432c).
absent0(strainChicken40209,cj1432c).
absent0(strainClinical36439,cj1432c).
absent0(strainClinical56519,cj1432c).
absent0(strainClinical32787,cj1432c).
absent0(strainClinicall31467,cj1432c).
absent0(strainClinical44119,cj1432c).
absent0(strainClinical34007,cj1432c).
absent0(strainClinical38762,cj1432c).
absent0(strainClinicalinical15168,cj1432c).
absent0(strainClinical18836,cj1432c).

absent0(strainClinicalM1,cj1432c).
absent0(strainClinical36860,cj1432c).
absent0(strainClinical40917,cj1432c).
absent0(strainClinical38857,cj1432c).
absent0(strainCjejuniRM1221,cj1432c).
absent0(strainChicken47693,cj1432c).
absent0(strainClinical63326,cj1432c).
absent0(strainClinical64555,cj1432c).
absent0(strainClinical59364,cj1432c).
absent0(strainClinical59424,cj1432c).
absent0(strainClinical33084,cj1432c).
absent0(strainClinical31481,cj1432c).
absent0(strainClinical39828,cj1432c).
absent0(strainBeach1771,cj1432c).
absent0(strainClinical53250,cj1432c).
absent0(strainClinical45557,cj1432c).
absent0(strainClinical47939,cj1432c).
absent0(strainClinicalG1,cj1433c).
absent0(strainClinicalG3,cj1433c).
absent0(strainChickenicken11974,cj1433c).
absent0(strainChickenicken13249,cj1433c).
absent0(strainClinical58473,cj1433c).
absent0(strainClinical43205,cj1433c).
absent0(strainClinical33106,cj1433c).
absent0(strainClinicall38556,cj1433c).
absent0(strainClinicall39182,cj1433c).
absent0(strainClinical44933,cj1433c).
absent0(strainClinical45631,cj1433c).
absent0(strainClinical41651,cj1433c).
absent0(strainClinical32799,cj1433c).
absent0(strainClinical44958,cj1433c).
absent0(strainClinical52331,cj1433c).
absent0(strainClinical56281,cj1433c).
absent0(strainClinical56282,cj1433c).
absent0(strainClinical56832,cj1433c).
absent0(strainOvine12241,cj1433c).
absent0(strainOvine12481,cj1433c).
absent0(strainBovinevineine13305,cj1433c).
absent0(strainChickenicken12912,cj1433c).
absent0(strainChickenicken11818,cj1433c).
absent0(strainChicken12196,cj1433c).
absent0(strainChickenicken13040,cj1433c).
absent0(strainChickenicken11856,cj1433c).
absent0(strainClinical36069,cj1433c).
absent0(strainChicken40209,cj1433c).
absent0(strainBeach1793,cj1433c).
absent0(strainClinical81116,cj1433c).
absent0(strainClinical36439,cj1433c).
absent0(strainClinical56519,cj1433c).
absent0(strainClinical32787,cj1433c).
absent0(strainClinicall31467,cj1433c).
absent0(strainClinical44119,cj1433c).
absent0(strainClinical34007,cj1433c).
absent0(strainClinical38762,cj1433c).

absent0(strainClinicalinical15168,cj1433c).
absent0(strainClinical18836,cj1433c).
absent0(strainClinicalM1,cj1433c).
absent0(strainClinical36860,cj1433c).
absent0(strainClinical40917,cj1433c).
absent0(strainClinical38857,cj1433c).
absent0(strainCjejuniRM1221,cj1433c).
absent0(strainChicken47693,cj1433c).
absent0(strainClinical63326,cj1433c).
absent0(strainClinical64555,cj1433c).
absent0(strainClinical59364,cj1433c).
absent0(strainClinical59424,cj1433c).
absent0(strainClinical33084,cj1433c).
absent0(strainClinical31481,cj1433c).
absent0(strainClinical39828,cj1433c).
absent0(strainBeach1771,cj1433c).
absent0(strainClinical53250,cj1433c).
absent0(strainClinical45557,cj1433c).
absent0(strainClinical47939,cj1433c).
absent0(strainClinicalG1,cj1434c).
absent0(strainClinicalG3,cj1434c).
absent0(strainChickenicken13249,cj1434c).
absent0(strainClinical58473,cj1434c).
absent0(strainClinical43205,cj1434c).
absent0(strainClinical33106,cj1434c).
absent0(strainClinicall38556,cj1434c).
absent0(strainClinicall39182,cj1434c).
absent0(strainClinical44933,cj1434c).
absent0(strainClinical45631,cj1434c).
absent0(strainClinical55320,cj1434c).
absent0(strainClinical41651,cj1434c).
absent0(strainClinical32799,cj1434c).
absent0(strainClinical43983,cj1434c).
absent0(strainClinical40671,cj1434c).
absent0(strainClinical44958,cj1434c).
absent0(strainClinical52331,cj1434c).
absent0(strainClinical56281,cj1434c).
absent0(strainClinical56282,cj1434c).
absent0(strainClinical56832,cj1434c).
absent0(strainOvine12241,cj1434c).
absent0(strainOvine12481,cj1434c).
absent0(strainBovinevineine13305,cj1434c).
absent0(strainChickenicken12912,cj1434c).
absent0(strainChickenicken11818,cj1434c).
absent0(strainChicken12196,cj1434c).
absent0(strainChickenicken13040,cj1434c).
absent0(strainChickenicken11856,cj1434c).
absent0(strainClinical36069,cj1434c).
absent0(strainChicken40209,cj1434c).
absent0(strainBeach1793,cj1434c).
absent0(strainClinical81116,cj1434c).
absent0(strainClinical36439,cj1434c).
absent0(strainClinical56519,cj1434c).
absent0(strainClinical32787,cj1434c).

absent0(strainClinicall31467,cj1434c).
absent0(strainClinical44119,cj1434c).
absent0(strainClinical34007,cj1434c).
absent0(strainClinical38762,cj1434c).
absent0(strainClinicalinical15168,cj1434c).
absent0(strainClinical18836,cj1434c).
absent0(strainClinicalM1,cj1434c).
absent0(strainClinical36860,cj1434c).
absent0(strainClinical40917,cj1434c).
absent0(strainClinical38857,cj1434c).
absent0(strainCjejuniRM1221,cj1434c).
absent0(strainChicken47693,cj1434c).
absent0(strainClinical63326,cj1434c).
absent0(strainClinical64555,cj1434c).
absent0(strainClinical59364,cj1434c).
absent0(strainClinical59424,cj1434c).
absent0(strainClinical33084,cj1434c).
absent0(strainClinical31481,cj1434c).
absent0(strainClinical39828,cj1434c).
absent0(strainBeach1771,cj1434c).
absent0(strainClinical53250,cj1434c).
absent0(strainClinical45557,cj1434c).
absent0(strainClinical47939,cj1434c).
absent0(strainClinicalG1,cj1435c).
absent0(strainClinicalG3,cj1435c).
absent0(strainChickenicken11974,cj1435c).
absent0(strainChickenicken13249,cj1435c).
absent0(strainClinical58473,cj1435c).
absent0(strainClinical43205,cj1435c).
absent0(strainClinical33106,cj1435c).
absent0(strainClinicall38556,cj1435c).
absent0(strainClinicall39182,cj1435c).
absent0(strainClinical45631,cj1435c).
absent0(strainClinical55320,cj1435c).
absent0(strainClinical41651,cj1435c).
absent0(strainClinical32799,cj1435c).
absent0(strainClinical40671,cj1435c).
absent0(strainClinical44958,cj1435c).
absent0(strainClinical52331,cj1435c).
absent0(strainClinical56281,cj1435c).
absent0(strainClinical56282,cj1435c).
absent0(strainClinical56832,cj1435c).
absent0(strainOvine12241,cj1435c).
absent0(strainOvine12481,cj1435c).
absent0(strainBovinevineine13305,cj1435c).
absent0(strainChickenicken12912,cj1435c).
absent0(strainChickenicken11818,cj1435c).
absent0(strainChicken12196,cj1435c).
absent0(strainChickenicken13040,cj1435c).
absent0(strainChickenicken11856,cj1435c).
absent0(strainClinical36069,cj1435c).
absent0(strainChicken40209,cj1435c).
absent0(strainBeach1793,cj1435c).
absent0(strainClinical81116,cj1435c).

absent0(strainClinical56519,cj1435c).
absent0(strainClinical32787,cj1435c).
absent0(strainClinicall31467,cj1435c).
absent0(strainClinical44119,cj1435c).
absent0(strainClinical34007,cj1435c).
absent0(strainClinical38762,cj1435c).
absent0(strainClinical18836,cj1435c).
absent0(strainClinicalM1,cj1435c).
absent0(strainClinical36860,cj1435c).
absent0(strainClinical40917,cj1435c).
absent0(strainClinical38857,cj1435c).
absent0(strainCjejuniRM1221,cj1435c).
absent0(strainChicken47693,cj1435c).
absent0(strainClinical63326,cj1435c).
absent0(strainClinical64555,cj1435c).
absent0(strainClinical59364,cj1435c).
absent0(strainClinical59424,cj1435c).
absent0(strainClinical33084,cj1435c).
absent0(strainClinical31481,cj1435c).
absent0(strainClinical39828,cj1435c).
absent0(strainBeach1771,cj1435c).
absent0(strainClinical53250,cj1435c).
absent0(strainClinical45557,cj1435c).
absent0(strainClinical47939,cj1435c).
absent0(strainClinicalG1,cj1436c).
absent0(strainClinicalG3,cj1436c).
absent0(strainChickenicken11974,cj1436c).
absent0(strainChickenicken13249,cj1436c).
absent0(strainClinical58473,cj1436c).
absent0(strainClinical43205,cj1436c).
absent0(strainClinical33106,cj1436c).
absent0(strainClinicall38556,cj1436c).
absent0(strainClinicall39182,cj1436c).
absent0(strainClinical44933,cj1436c).
absent0(strainClinical45631,cj1436c).
absent0(strainClinical41651,cj1436c).
absent0(strainClinical32799,cj1436c).
absent0(strainClinical43983,cj1436c).
absent0(strainClinical40671,cj1436c).
absent0(strainClinical44958,cj1436c).
absent0(strainClinical52331,cj1436c).
absent0(strainClinical56281,cj1436c).
absent0(strainClinical56282,cj1436c).
absent0(strainClinical56832,cj1436c).
absent0(strainOvine12241,cj1436c).
absent0(strainOvine12481,cj1436c).
absent0(strainBovinevineine13305,cj1436c).
absent0(strainChickenicken12912,cj1436c).
absent0(strainChickenicken11818,cj1436c).
absent0(strainChicken12196,cj1436c).
absent0(strainChickenicken13040,cj1436c).
absent0(strainChickenicken11856,cj1436c).
absent0(strainClinical36069,cj1436c).
absent0(strainChicken40209,cj1436c).

absent0(strainClinical81116,cj1436c).
absent0(strainClinical36439,cj1436c).
absent0(strainClinical56519,cj1436c).
absent0(strainClinical32787,cj1436c).
absent0(strainClinicall31467,cj1436c).
absent0(strainClinical44119,cj1436c).
absent0(strainClinical34007,cj1436c).
absent0(strainClinical38762,cj1436c).
absent0(strainClinicalinical15168,cj1436c).
absent0(strainClinical18836,cj1436c).
absent0(strainClinicalM1,cj1436c).
absent0(strainClinical36860,cj1436c).
absent0(strainClinical40917,cj1436c).
absent0(strainCjejuniRM1221,cj1436c).
absent0(strainChicken47693,cj1436c).
absent0(strainClinical63326,cj1436c).
absent0(strainClinical64555,cj1436c).
absent0(strainClinical59364,cj1436c).
absent0(strainClinical59424,cj1436c).
absent0(strainClinical33084,cj1436c).
absent0(strainClinical31481,cj1436c).
absent0(strainClinical39828,cj1436c).
absent0(strainBeach1771,cj1436c).
absent0(strainClinical53250,cj1436c).
absent0(strainClinical45557,cj1436c).
absent0(strainClinical47939,cj1436c).
absent0(strainClinicalG1,cj1437c).
absent0(strainClinicalG3,cj1437c).
absent0(strainChickenicken11974,cj1437c).
absent0(strainChickenicken13249,cj1437c).
absent0(strainClinical58473,cj1437c).
absent0(strainClinical43205,cj1437c).
absent0(strainClinical33106,cj1437c).
absent0(strainClinicall38556,cj1437c).
absent0(strainClinicall39182,cj1437c).
absent0(strainClinical44933,cj1437c).
absent0(strainClinical45631,cj1437c).
absent0(strainClinical55320,cj1437c).
absent0(strainClinical41651,cj1437c).
absent0(strainClinical32799,cj1437c).
absent0(strainClinical43983,cj1437c).
absent0(strainClinical40671,cj1437c).
absent0(strainClinical44958,cj1437c).
absent0(strainClinical52331,cj1437c).
absent0(strainClinical56281,cj1437c).
absent0(strainClinical56282,cj1437c).
absent0(strainClinical56832,cj1437c).
absent0(strainOvine12241,cj1437c).
absent0(strainOvine12481,cj1437c).
absent0(strainBovinevineine13305,cj1437c).
absent0(strainChickenicken12912,cj1437c).
absent0(strainChickenicken11818,cj1437c).
absent0(strainChicken12196,cj1437c).
absent0(strainChickenicken13040,cj1437c).

absent0(strainChickenicken11856,cj1437c).
absent0(strainClinical36069,cj1437c).
absent0(strainChicken40209,cj1437c).
absent0(strainBeach1793,cj1437c).
absent0(strainClinical81116,cj1437c).
absent0(strainClinical36439,cj1437c).
absent0(strainClinical56519,cj1437c).
absent0(strainClinical32787,cj1437c).
absent0(strainClinicall31467,cj1437c).
absent0(strainClinical44119,cj1437c).
absent0(strainClinical34007,cj1437c).
absent0(strainClinical38762,cj1437c).
absent0(strainClinicalinical15168,cj1437c).
absent0(strainClinical18836,cj1437c).
absent0(strainClinicalM1,cj1437c).
absent0(strainClinical36860,cj1437c).
absent0(strainClinical40917,cj1437c).
absent0(strainClinical38857,cj1437c).
absent0(strainCjejuniRM1221,cj1437c).
absent0(strainChicken47693,cj1437c).
absent0(strainClinical63326,cj1437c).
absent0(strainClinical64555,cj1437c).
absent0(strainClinical59364,cj1437c).
absent0(strainClinical59424,cj1437c).
absent0(strainClinical33084,cj1437c).
absent0(strainClinical31481,cj1437c).
absent0(strainClinical39828,cj1437c).
absent0(strainBeach1771,cj1437c).
absent0(strainClinical53250,cj1437c).
absent0(strainClinical45557,cj1437c).
absent0(strainClinical47939,cj1437c).
absent0(strainClinicalG1,cj1438c).
absent0(strainClinicalG3,cj1438c).
absent0(strainChickenicken11974,cj1438c).
absent0(strainChickenicken13249,cj1438c).
absent0(strainClinical58473,cj1438c).
absent0(strainClinical43205,cj1438c).
absent0(strainClinical33106,cj1438c).
absent0(strainClinicall38556,cj1438c).
absent0(strainClinicall39182,cj1438c).
absent0(strainClinical44933,cj1438c).
absent0(strainClinical45631,cj1438c).
absent0(strainClinical55320,cj1438c).
absent0(strainClinical41651,cj1438c).
absent0(strainClinical32799,cj1438c).
absent0(strainClinical43983,cj1438c).
absent0(strainClinical40671,cj1438c).
absent0(strainClinical44958,cj1438c).
absent0(strainClinical52331,cj1438c).
absent0(strainClinical56281,cj1438c).
absent0(strainClinical56282,cj1438c).
absent0(strainClinical56832,cj1438c).
absent0(strainOvine12241,cj1438c).
absent0(strainOvine12481,cj1438c).

absent0(strainBovinevineine13305,cj1438c).
absent0(strainChickenicken12912,cj1438c).
absent0(strainChickenicken11818,cj1438c).
absent0(strainChicken12196,cj1438c).
absent0(strainChickenicken13040,cj1438c).
absent0(strainChickenicken11856,cj1438c).
absent0(strainClinical36069,cj1438c).
absent0(strainChicken40209,cj1438c).
absent0(strainBeach1793,cj1438c).
absent0(strainClinical81116,cj1438c).
absent0(strainClinical36439,cj1438c).
absent0(strainClinical56519,cj1438c).
absent0(strainClinical32787,cj1438c).
absent0(strainClinicall31467,cj1438c).
absent0(strainClinical44119,cj1438c).
absent0(strainClinical34007,cj1438c).
absent0(strainClinical38762,cj1438c).
absent0(strainClinicalinical15168,cj1438c).
absent0(strainClinical18836,cj1438c).
absent0(strainClinicalM1,cj1438c).
absent0(strainClinical36860,cj1438c).
absent0(strainClinical40917,cj1438c).
absent0(strainClinical38857,cj1438c).
absent0(strainCjejuniRM1221,cj1438c).
absent0(strainChicken47693,cj1438c).
absent0(strainClinical63326,cj1438c).
absent0(strainClinical64555,cj1438c).
absent0(strainClinical59364,cj1438c).
absent0(strainClinical59424,cj1438c).
absent0(strainClinical33084,cj1438c).
absent0(strainClinical31481,cj1438c).
absent0(strainClinical39828,cj1438c).
absent0(strainBeach1771,cj1438c).
absent0(strainClinical53250,cj1438c).
absent0(strainClinical45557,cj1438c).
absent0(strainClinical47939,cj1438c).
absent0(strainClinicalG3,cj1439c).
absent0(strainChickenicken11974,cj1439c).
absent0(strainChickenicken13249,cj1439c).
absent0(strainClinical58473,cj1439c).
absent0(strainClinical43205,cj1439c).
absent0(strainClinical33106,cj1439c).
absent0(strainClinicall38556,cj1439c).
absent0(strainClinicall39182,cj1439c).
absent0(strainClinical44933,cj1439c).
absent0(strainClinical45631,cj1439c).
absent0(strainClinical55320,cj1439c).
absent0(strainClinical41651,cj1439c).
absent0(strainClinical32799,cj1439c).
absent0(strainClinical40671,cj1439c).
absent0(strainClinical52331,cj1439c).
absent0(strainClinical56281,cj1439c).
absent0(strainClinical56282,cj1439c).
absent0(strainClinical56832,cj1439c).

absent0(strainOvine12241,cj1439c).
absent0(strainOvine12481,cj1439c).
absent0(strainBovinevineine13305,cj1439c).
absent0(strainChickenicken12912,cj1439c).
absent0(strainChickenicken11818,cj1439c).
absent0(strainChicken12196,cj1439c).
absent0(strainChickenicken13040,cj1439c).
absent0(strainChickenicken11856,cj1439c).
absent0(strainClinical36069,cj1439c).
absent0(strainChicken40209,cj1439c).
absent0(strainBeach1793,cj1439c).
absent0(strainClinical81116,cj1439c).
absent0(strainClinical56519,cj1439c).
absent0(strainClinical32787,cj1439c).
absent0(strainClinicall31467,cj1439c).
absent0(strainClinical44119,cj1439c).
absent0(strainClinical34007,cj1439c).
absent0(strainClinical38762,cj1439c).
absent0(strainClinicalinical15168,cj1439c).
absent0(strainClinical18836,cj1439c).
absent0(strainClinicalM1,cj1439c).
absent0(strainClinical36860,cj1439c).
absent0(strainClinical40917,cj1439c).
absent0(strainClinical38857,cj1439c).
absent0(strainCjejuniRM1221,cj1439c).
absent0(strainChicken47693,cj1439c).
absent0(strainClinical63326,cj1439c).
absent0(strainClinical64555,cj1439c).
absent0(strainClinical59364,cj1439c).
absent0(strainClinical59424,cj1439c).
absent0(strainClinical33084,cj1439c).
absent0(strainClinical31481,cj1439c).
absent0(strainClinical39828,cj1439c).
absent0(strainBeach1771,cj1439c).
absent0(strainClinical53250,cj1439c).
absent0(strainClinical45557,cj1439c).
absent0(strainClinical47939,cj1439c).
absent0(strainClinicalG1,cj1440c).
absent0(strainClinicalG3,cj1440c).
absent0(strainChickenicken11974,cj1440c).
absent0(strainChickenicken13249,cj1440c).
absent0(strainClinical58473,cj1440c).
absent0(strainClinical43205,cj1440c).
absent0(strainClinical33106,cj1440c).
absent0(strainClinicall38556,cj1440c).
absent0(strainClinicall39182,cj1440c).
absent0(strainClinical44933,cj1440c).
absent0(strainClinical45631,cj1440c).
absent0(strainClinical41651,cj1440c).
absent0(strainClinical32799,cj1440c).
absent0(strainClinical43983,cj1440c).
absent0(strainClinical40671,cj1440c).
absent0(strainClinical44958,cj1440c).
absent0(strainClinical52331,cj1440c).

absent0(strainClinical56282,cj1440c).
absent0(strainClinical56832,cj1440c).
absent0(strainOvine12241,cj1440c).
absent0(strainOvine12481,cj1440c).
absent0(strainBovinevineine13305,cj1440c).
absent0(strainChickenicken12912,cj1440c).
absent0(strainChickenicken11818,cj1440c).
absent0(strainChicken12196,cj1440c).
absent0(strainChickenicken13040,cj1440c).
absent0(strainChickenicken11856,cj1440c).
absent0(strainClinical36069,cj1440c).
absent0(strainChicken40209,cj1440c).
absent0(strainBeach1793,cj1440c).
absent0(strainClinical81116,cj1440c).
absent0(strainClinical36439,cj1440c).
absent0(strainClinical56519,cj1440c).
absent0(strainClinical32787,cj1440c).
absent0(strainClinicall31467,cj1440c).
absent0(strainClinical44119,cj1440c).
absent0(strainClinical34007,cj1440c).
absent0(strainClinical38762,cj1440c).
absent0(strainClinicalinical15168,cj1440c).
absent0(strainClinical18836,cj1440c).
absent0(strainClinicalM1,cj1440c).
absent0(strainClinical36860,cj1440c).
absent0(strainClinical40917,cj1440c).
absent0(strainClinical38857,cj1440c).
absent0(strainCjejuniRM1221,cj1440c).
absent0(strainChicken47693,cj1440c).
absent0(strainClinical63326,cj1440c).
absent0(strainClinical59364,cj1440c).
absent0(strainClinical59424,cj1440c).
absent0(strainClinical33084,cj1440c).
absent0(strainClinical31481,cj1440c).
absent0(strainClinical39828,cj1440c).
absent0(strainBeach1771,cj1440c).
absent0(strainClinical53250,cj1440c).
absent0(strainClinical45557,cj1440c).
absent0(strainClinical47939,cj1440c).
absent0(strainClinicalG1,cj1441c).
absent0(strainClinicalG3,cj1441c).
absent0(strainChickenicken11974,cj1441c).
absent0(strainChickenicken13249,cj1441c).
absent0(strainClinical58473,cj1441c).
absent0(strainClinical43205,cj1441c).
absent0(strainClinical33106,cj1441c).
absent0(strainClinicall38556,cj1441c).
absent0(strainClinicall39182,cj1441c).
absent0(strainClinical44933,cj1441c).
absent0(strainClinical45631,cj1441c).
absent0(strainClinical41651,cj1441c).
absent0(strainClinical32799,cj1441c).
absent0(strainClinical43983,cj1441c).
absent0(strainClinical40671,cj1441c).

absent0(strainClinical44958,cj1441c).
absent0(strainClinical52331,cj1441c).
absent0(strainClinical56282,cj1441c).
absent0(strainClinical56832,cj1441c).
absent0(strainOvine12241,cj1441c).
absent0(strainOvine12481,cj1441c).
absent0(strainBovinevineine13305,cj1441c).
absent0(strainChickenicken12912,cj1441c).
absent0(strainChickenicken11818,cj1441c).
absent0(strainChicken12196,cj1441c).
absent0(strainChickenicken13040,cj1441c).
absent0(strainChickenicken11856,cj1441c).
absent0(strainClinical36069,cj1441c).
absent0(strainChicken40209,cj1441c).
absent0(strainBeach1793,cj1441c).
absent0(strainClinical81116,cj1441c).
absent0(strainClinical36439,cj1441c).
absent0(strainClinical56519,cj1441c).
absent0(strainClinical32787,cj1441c).
absent0(strainClinicall31467,cj1441c).
absent0(strainClinical44119,cj1441c).
absent0(strainClinical34007,cj1441c).
absent0(strainClinical38762,cj1441c).
absent0(strainClinicalinical15168,cj1441c).
absent0(strainClinical18836,cj1441c).
absent0(strainClinicalM1,cj1441c).
absent0(strainClinical36860,cj1441c).
absent0(strainClinical40917,cj1441c).
absent0(strainClinical38857,cj1441c).
absent0(strainCjejuniRM1221,cj1441c).
absent0(strainChicken47693,cj1441c).
absent0(strainClinical63326,cj1441c).
absent0(strainClinical64555,cj1441c).
absent0(strainClinical59364,cj1441c).
absent0(strainClinical59424,cj1441c).
absent0(strainClinical33084,cj1441c).
absent0(strainClinical31481,cj1441c).
absent0(strainClinical39828,cj1441c).
absent0(strainBeach1771,cj1441c).
absent0(strainClinical53250,cj1441c).
absent0(strainClinical45557,cj1441c).
absent0(strainClinical47939,cj1441c).
absent0(strainClinicalG1,cj1442c).
absent0(strainClinicalG3,cj1442c).
absent0(strainChickenicken11974,cj1442c).
absent0(strainChickenicken13249,cj1442c).
absent0(strainClinical33106,cj1442c).
absent0(strainClinicall38556,cj1442c).
absent0(strainClinicall39182,cj1442c).
absent0(strainClinical44933,cj1442c).
absent0(strainClinical45631,cj1442c).
absent0(strainClinical55320,cj1442c).
absent0(strainClinical32799,cj1442c).
absent0(strainClinical56832,cj1442c).

absent0(strainChickenicken12912,cj1442c).
absent0(strainClinical36069,cj1442c).
absent0(strainChicken40209,cj1442c).
absent0(strainBeach1793,cj1442c).
absent0(strainClinical81116,cj1442c).
absent0(strainClinical36439,cj1442c).
absent0(strainClinical32787,cj1442c).
absent0(strainClinicall31467,cj1442c).
absent0(strainClinical44119,cj1442c).
absent0(strainClinical34007,cj1442c).
absent0(strainClinical38762,cj1442c).
absent0(strainClinicalinical15168,cj1442c).
absent0(strainClinical18836,cj1442c).
absent0(strainClinicalM1,cj1442c).
absent0(strainClinical36860,cj1442c).
absent0(strainClinical40917,cj1442c).
absent0(strainClinical38857,cj1442c).
absent0(strainCjejuniRM1221,cj1442c).
absent0(strainClinical63326,cj1442c).
absent0(strainClinical64555,cj1442c).
absent0(strainClinical59364,cj1442c).
absent0(strainClinical59424,cj1442c).
absent0(strainClinical33084,cj1442c).
absent0(strainClinical31481,cj1442c).
absent0(strainClinical39828,cj1442c).
absent0(strainClinical45557,cj1442c).
absent0(strainClinical47939,cj1442c).
absent0(strainChickenicken12912,cj1443c).
absent0(strainChickenicken11818,cj1443c).
absent0(strainClinical36439,cj1443c).
absent0(strainClinical32787,cj1443c).
absent0(strainClinicall31467,cj1443c).
absent0(strainClinical38762,cj1443c).
absent0(strainCjejuniRM1221,cj1443c).
absent0(strainClinicalG3,cj1445c).
absent0(strainClinical32799,cj1445c).
absent0(strainChickenicken11818,cj1445c).
absent0(strainClinical39828,cj1445c).
absent0(strainClinicalG1,cj1447c).
absent0(strainClinicalG3,cj1447c).
absent0(strainChickenicken11974,cj1447c).
absent0(strainChickenicken13249,cj1447c).
absent0(strainClinical33106,cj1447c).
absent0(strainClinicall38556,cj1447c).
absent0(strainClinicall39182,cj1447c).
absent0(strainClinical44933,cj1447c).
absent0(strainClinical45631,cj1447c).
absent0(strainClinical55320,cj1447c).
absent0(strainClinical41651,cj1447c).
absent0(strainClinical32799,cj1447c).
absent0(strainClinical43983,cj1447c).
absent0(strainClinical40671,cj1447c).
absent0(strainClinical44958,cj1447c).
absent0(strainClinical52331,cj1447c).

absent0(strainClinical56281,cj1447c).
absent0(strainClinical56282,cj1447c).
absent0(strainClinical56832,cj1447c).
absent0(strainOvine12241,cj1447c).
absent0(strainOvine12481,cj1447c).
absent0(strainBovinevineine13305,cj1447c).
absent0(strainChickenicken11818,cj1447c).
absent0(strainChicken12196,cj1447c).
absent0(strainChickenicken13040,cj1447c).
absent0(strainChickenicken11856,cj1447c).
absent0(strainClinical36069,cj1447c).
absent0(strainBeach1793,cj1447c).
absent0(strainClinical36439,cj1447c).
absent0(strainClinical56519,cj1447c).
absent0(strainClinical32787,cj1447c).
absent0(strainClinicall31467,cj1447c).
absent0(strainClinical44119,cj1447c).
absent0(strainClinical34007,cj1447c).
absent0(strainClinical38762,cj1447c).
absent0(strainClinicalinical15168,cj1447c).
absent0(strainClinical18836,cj1447c).
absent0(strainClinicalM1,cj1447c).
absent0(strainClinical36860,cj1447c).
absent0(strainClinical40917,cj1447c).
absent0(strainClinical38857,cj1447c).
absent0(strainChicken47693,cj1447c).
absent0(strainClinical63326,cj1447c).
absent0(strainClinical64555,cj1447c).
absent0(strainClinical59364,cj1447c).
absent0(strainClinical59424,cj1447c).
absent0(strainClinical33084,cj1447c).
absent0(strainClinical31481,cj1447c).
absent0(strainClinical39828,cj1447c).
absent0(strainBeach1771,cj1447c).
absent0(strainClinical53250,cj1447c).
absent0(strainClinical45557,cj1447c).
absent0(strainClinical47939,cj1447c).
absent0(strainClinicalG1,cj1448c).
absent0(strainClinicalG3,cj1448c).
absent0(strainChickenicken11974,cj1448c).
absent0(strainChickenicken13249,cj1448c).
absent0(strainClinical33106,cj1448c).
absent0(strainClinicall38556,cj1448c).
absent0(strainClinicall39182,cj1448c).
absent0(strainClinical44933,cj1448c).
absent0(strainClinical45631,cj1448c).
absent0(strainClinical41651,cj1448c).
absent0(strainClinical32799,cj1448c).
absent0(strainClinical43983,cj1448c).
absent0(strainClinical40671,cj1448c).
absent0(strainClinical44958,cj1448c).
absent0(strainClinical52331,cj1448c).
absent0(strainClinical56281,cj1448c).
absent0(strainClinical56282,cj1448c).

absent0(strainClinical56832,cj1448c).
absent0(strainOvine12241,cj1448c).
absent0(strainOvine12481,cj1448c).
absent0(strainBovinevineine13305,cj1448c).
absent0(strainChickenicken11818,cj1448c).
absent0(strainChicken12196,cj1448c).
absent0(strainChickenicken13040,cj1448c).
absent0(strainChickenicken11856,cj1448c).
absent0(strainClinical36069,cj1448c).
absent0(strainClinical36439,cj1448c).
absent0(strainClinical56519,cj1448c).
absent0(strainClinical32787,cj1448c).
absent0(strainClinicall31467,cj1448c).
absent0(strainClinical44119,cj1448c).
absent0(strainClinical34007,cj1448c).
absent0(strainClinical38762,cj1448c).
absent0(strainClinicalinical15168,cj1448c).
absent0(strainClinical18836,cj1448c).
absent0(strainClinicalM1,cj1448c).
absent0(strainClinical40917,cj1448c).
absent0(strainClinical38857,cj1448c).
absent0(strainCjejuniRM1221,cj1448c).
absent0(strainChicken47693,cj1448c).
absent0(strainClinical63326,cj1448c).
absent0(strainClinical64555,cj1448c).
absent0(strainClinical59364,cj1448c).
absent0(strainClinical59424,cj1448c).
absent0(strainClinical33084,cj1448c).
absent0(strainClinical31481,cj1448c).
absent0(strainClinical39828,cj1448c).
absent0(strainBeach1771,cj1448c).
absent0(strainClinical45557,cj1448c).
absent0(strainClinical47939,cj1448c).

```
%	FILE LEARN_H_PL
%	Example learning file for the reaction ?H in the paper. This is an input file
%	for Progol 5.0 which loads other files in this directory into Progol and does the
%	learning.


:-set(r,10)?
:-set(h,10000)?

:-['background.pl']?
:-['codes.pl']?
:-['common.pl']?
:-['CPS_pathway.pl']?
:-['glycan_structure.pl']?
:-['mutants.pl']?
:-['strains.pl']?

%%%%%%%%%%%%%%%%%%%%%%%%%%%%%%%%%%%%%%%%%%%%%%%%%%%%%%%%%%%
%%%%%

unassigned_reaction(capsule_pre_pre_ribf).  % reaction A
unassigned_reaction(capsule_37).  % reaction B
unassigned_reaction(capsule_0). % reaction C
unassigned_reaction(capsule_22).  % reaction D
unassigned_reaction(capsule_pre_pre_omepn). % reaction H

unassigned_gene(cj1416c).
%unassigned_gene(cj1417c).
%unassigned_gene(cj1418c).

%unassigned_gene(cj1432c).
%unassigned_gene(cj1434c).
%unassigned_gene(cj1438c).
%unassigned_gene(cj1440c).
%unassigned_gene(cj1442c).
```

FILE   LEARN_H_OUT

```
CProgol Version 5.0

[:- set(r,10)? - Time taken 0.00s]
[:- set(h,10000)? - Time taken 0.00s]
[:- modeh(1,codes(#any,#any))? - Time taken 0.00s]
[:- observable(knockout_observable/2)? - Time taken 0.00s]
[Testing for contradictions]
[No contradictions found]
[:- [background.pl]? - Time taken 0.00s]
[Testing for contradictions]
[No contradictions found]
[:- [codes.pl]? - Time taken 0.00s]
[Testing for contradictions]
[No contradictions found]
[:- [common.pl]? - Time taken 0.01s]
[Testing for contradictions]
[No contradictions found]
[:- [CPS_pathway.pl]? - Time taken 0.02s]
[Testing for contradictions]
[No contradictions found]
[:- [glycan_structure.pl]? - Time taken 0.00s]
[Testing for contradictions]
[No contradictions found]
[:- [mutants.pl]? - Time taken 0.00s]
[Testing for contradictions]
[No contradictions found]
[:- [strains.pl]? - Time taken 0.04s]
[Testing for contradictions]
[No contradictions found]
[Generalising knockout_observable/2]
[Generalising knockout_observable(cj1416c,'glca6ngro+hep+6ome-galfnac-ribf').]
[Generalising <codes(cj1416c,capsule_pre_pre_omepn).>]
[Most specific clause is]

codes(cj1416c,capsule_pre_pre_omepn).

[C:1211,138,0,0 codes(cj1416c,capsule_pre_pre_omepn).]
[1 explored search nodes]
f=1211,p=138,n=0,h=0
[Result of search is]

codes(cj1416c,capsule_pre_pre_omepn).

[Result of search is]

codes(cj1416c,capsule_pre_pre_omepn).

[1 redundant clauses retracted from knockout_observable/2]
[130 redundant clauses retracted from occurs/2]
[Generalising knockout_observable(cj1417c,'glca6ngro+hep+6ome-galfnac-ribf').]
[Generalising knockout_observable(cj1418c,'glca6ngro+hep+6ome-galfnac-ribf').]
[Generalising knockout_observable(cj1421c,'glca6ngro+hep+6ome-galfnac+omepn-ribf').]
```

[Generalising knockout_observable(cj1422c,'glca6ngro+hep+omepn+6ome-galfnac-ribf').]
[Generalising knockout_observable(cj1423c,'glca6ngro-galfnac+omepn-ribf').]
[Generalising knockout_observable(cj1424c,'glca6ngro-galfnac+omepn-ribf').]
[Generalising knockout_observable(cj1425c,'glca6ngro-galfnac+omepn-ribf').]
[Generalising knockout_observable(cj1426c,'glca6ngro+hep-galfnac+omepn-ribf').]
[Generalising knockout_observable(cj1427c,'glca6ngro-galfnac+omepn-ribf').]
[Generalising knockout_observable(cj1428c,'glca6ngro-galfnac+omepn-ribf').]
[Generalising knockout_observable(cj1430c,'glca6ngro-galfnac+omepn-ribf').]
[Generalising knockout_observable(cj1431c,'glca6ngro-galfnac+omepn-ribf').]
[Generalising knockout_observable(cj1432c,acapsular).]
[Generalising knockout_observable(cj1434c,acapsular).]
[Generalising knockout_observable(cj1435c,acapsular).]
[Generalising knockout_observable(cj1437c,acapsular).]
[Generalising knockout_observable(cj1438c,acapsular).]
[Generalising knockout_observable(cj1439c,acapsular).]
[Generalising knockout_observable(cj1440c,acapsular).]
[Generalising knockout_observable(cj1441c,acapsular).]
[Generalising knockout_observable(cj1442c,acapsular).]
[Generalising knockout_observable(cj1448c,acapsular).]
knockout_observable(Gene,Observable) :- unassigned_gene(Gene),
    struct_observable(Prs_Struct,Observable), struct_next(Prs_Struct,
    Abs_Struct), path3(R,Prs_Struct,Abs_Struct), unassigned_reaction(R),
    codes(Gene,R).
knockout_observable(cj1417c,'glca6ngro+hep+6ome-galfnac-ribf').
knockout_observable(cj1418c,'glca6ngro+hep+6ome-galfnac-ribf').
knockout_observable(cj1421c,'glca6ngro+hep+6ome-galfnac+omepn-ribf').
knockout_observable(cj1422c,'glca6ngro+hep+omepn+6ome-galfnac-ribf').
knockout_observable(cj1423c,'glca6ngro-galfnac+omepn-ribf').
knockout_observable(cj1424c,'glca6ngro-galfnac+omepn-ribf').
knockout_observable(cj1425c,'glca6ngro-galfnac+omepn-ribf').
knockout_observable(cj1426c,'glca6ngro+hep-galfnac+omepn-ribf').
knockout_observable(cj1427c,'glca6ngro-galfnac+omepn-ribf').
knockout_observable(cj1428c,'glca6ngro-galfnac+omepn-ribf').
knockout_observable(cj1430c,'glca6ngro-galfnac+omepn-ribf').
knockout_observable(cj1431c,'glca6ngro-galfnac+omepn-ribf').
knockout_observable(cj1432c,acapsular).
knockout_observable(cj1434c,acapsular).
knockout_observable(cj1435c,acapsular).
knockout_observable(cj1437c,acapsular).
knockout_observable(cj1438c,acapsular).
knockout_observable(cj1439c,acapsular).
knockout_observable(cj1440c,acapsular).
knockout_observable(cj1441c,acapsular).
knockout_observable(cj1442c,acapsular).
knockout_observable(cj1448c,acapsular).

[Total number of clauses = 23]

[Time taken 0.050s]

[The following definitions changed]

codes(cj1421c,capsule_ngro36a).

```
codes(cj1422c,capsule_ngro21a).
codes(cj1426c,capsule_ngro21c).
codes(cj1430c,capsule_hep5).
codes(cj1431c,capsule_ngro1c).
codes(cj1439c,capsule_pre_pre_galfnac).
codes(cj1441c,capsule_pre_pre_glca).
codes(cj1416c,capsule_pre_pre_omepn).

knockout_observable(Gene,Observable) :- unassigned_gene(Gene),
        struct_observable(Prs_Struct,Observable), struct_next(Prs_Struct,
        Abs_Struct), path3(R,Prs_Struct,Abs_Struct), unassigned_reaction(R),
        codes(Gene,R).
knockout_observable(cj1417c,'glca6ngro+hep+6ome-galfnac-ribf').
knockout_observable(cj1418c,'glca6ngro+hep+6ome-galfnac-ribf').
knockout_observable(cj1421c,'glca6ngro+hep+6ome-galfnac+omepn-ribf').
knockout_observable(cj1422c,'glca6ngro+hep+omepn+6ome-galfnac-ribf').
knockout_observable(cj1423c,'glca6ngro-galfnac+omepn-ribf').
knockout_observable(cj1424c,'glca6ngro-galfnac+omepn-ribf').
knockout_observable(cj1425c,'glca6ngro-galfnac+omepn-ribf').
knockout_observable(cj1426c,'glca6ngro+hep-galfnac+omepn-ribf').
knockout_observable(cj1427c,'glca6ngro-galfnac+omepn-ribf').
knockout_observable(cj1428c,'glca6ngro-galfnac+omepn-ribf').
knockout_observable(cj1430c,'glca6ngro-galfnac+omepn-ribf').
knockout_observable(cj1431c,'glca6ngro-galfnac+omepn-ribf').
knockout_observable(cj1432c,acapsular).
knockout_observable(cj1434c,acapsular).
knockout_observable(cj1435c,acapsular).
knockout_observable(cj1437c,acapsular).
knockout_observable(cj1438c,acapsular).
knockout_observable(cj1439c,acapsular).
knockout_observable(cj1440c,acapsular).
knockout_observable(cj1441c,acapsular).
knockout_observable(cj1442c,acapsular).
knockout_observable(cj1448c,acapsular).

occurs(Strain1,Gene) :- codes(Gene,R1), neighbour_reaction(R1,
        R2), codes(G2,R2), occurs0(Strain2,G2), have_same_serotype(Strain1,
        Strain2), !.
occurs(strainClinicalG1,cj1413c).
occurs(strainClinicalG3,cj1413c).
occurs(strainChickenicken11974,cj1413c).
occurs(strainChickenicken13249,cj1413c).
occurs(strainChickenicken13411,cj1413c).
occurs(strainClinical44811,cj1413c).
occurs(strainClinical48612,cj1413c).
occurs(strainClinical58473,cj1413c).
occurs(strainChicken11848,cj1413c).
occurs(strainChicken12567,cj1413c).
occurs(strainChicken11919,cj1413c).
occurs(strainChicken11973,cj1413c).
occurs(strainChicken13713,cj1413c).
occurs(strainBeach1791,cj1413c).
occurs(strainClinical43205,cj1413c).
occurs(strainClinical33106,cj1413c).
```

occurs(strainClinicall38556,cj1413c).
occurs(strainClinicall39182,cj1413c).
occurs(strainClinical44933,cj1413c).
occurs(strainClinical45631,cj1413c).
occurs(strainClinical55320,cj1413c).
occurs(strainClinical55703,cj1413c).
occurs(strainClinical30280,cj1413c).
occurs(strainClinical30328,cj1413c).
occurs(strainClinical41651,cj1413c).
occurs(strainClinical32799,cj1413c).
occurs(strainClinical43983,cj1413c).
occurs(strainClinical40671,cj1413c).
occurs(strainClinical44958,cj1413c).
occurs(strainClinical52331,cj1413c).
occurs(strainClinical56281,cj1413c).
occurs(strainClinical56282,cj1413c).
occurs(strainClinical56832,cj1413c).
occurs(strainOvine12241,cj1413c).
occurs(strainOvine12481,cj1413c).
occurs(strainBovinevineine13305,cj1413c).
occurs(strainChickenicken12912,cj1413c).
occurs(strainChickenicken11818,cj1413c).
occurs(strainChicken12196,cj1413c).
occurs(strainChickenicken12450,cj1413c).
occurs(strainChickenicken12487,cj1413c).
occurs(strainChickenicken13082,cj1413c).
occurs(strainChickenicken13040,cj1413c).
occurs(strainChickenicken11856,cj1413c).
occurs(strainClinical36069,cj1413c).
occurs(strainChicken40209,cj1413c).
occurs(strainBeach1793,cj1413c).
occurs(strainClinical81116,cj1413c).
occurs(strainClinical36439,cj1413c).
occurs(strainClinical56519,cj1413c).
occurs(strainClinical32787,cj1413c).
occurs(strainClinicall31467,cj1413c).
occurs(strainClinical44119,cj1413c).
occurs(strainClinical34007,cj1413c).
occurs(strainClinical38762,cj1413c).
occurs(strainClinicalinical15168,cj1413c).
occurs(strainClinical18836,cj1413c).
occurs(strainClinicalM1,cj1413c).
occurs(strainClinical36860,cj1413c).
occurs(strainClinical40917,cj1413c).
occurs(strainClinical38857,cj1413c).
occurs(strainCjejuniRM1221,cj1413c).
occurs(strainChicken47693,cj1413c).
occurs(strainClinical63326,cj1413c).
occurs(strainClinical64555,cj1413c).
occurs(strainClinical59364,cj1413c).
occurs(strainClinical59424,cj1413c).
occurs(strainClinical33084,cj1413c).
occurs(strainClinical31481,cj1413c).
occurs(strainClinical39828,cj1413c).

```
occurs(strainBeach1771,cj1413c).
occurs(strainClinical53250,cj1413c).
occurs(strainClinical45557,cj1413c).
occurs(strainClinical47939,cj1413c).
occurs(strainClinicalG1,cj1414c).
occurs(strainChickenicken11974,cj1414c).
occurs(strainChickenicken13249,cj1414c).
occurs(strainChickenicken13411,cj1414c).
occurs(strainClinical44811,cj1414c).
occurs(strainClinical48612,cj1414c).
occurs(strainClinical58473,cj1414c).
occurs(strainChicken11848,cj1414c).
occurs(strainChicken12567,cj1414c).
occurs(strainChicken11919,cj1414c).
occurs(strainChicken11973,cj1414c).
occurs(strainChicken13713,cj1414c).
occurs(strainBeach1791,cj1414c).
occurs(strainClinical43205,cj1414c).
occurs(strainClinical33106,cj1414c).
occurs(strainClinicall38556,cj1414c).
occurs(strainClinicall39182,cj1414c).
occurs(strainClinical44933,cj1414c).
occurs(strainClinical45631,cj1414c).
occurs(strainClinical55320,cj1414c).
occurs(strainClinical55703,cj1414c).
occurs(strainClinical30280,cj1414c).
occurs(strainClinical30328,cj1414c).
occurs(strainClinical41651,cj1414c).
occurs(strainClinical40671,cj1414c).
occurs(strainClinical44958,cj1414c).
occurs(strainClinical52331,cj1414c).
occurs(strainClinical56281,cj1414c).
occurs(strainClinical56282,cj1414c).
occurs(strainClinical56832,cj1414c).
occurs(strainOvine12241,cj1414c).
occurs(strainOvine12481,cj1414c).
occurs(strainChickenicken12912,cj1414c).
occurs(strainChicken12196,cj1414c).
occurs(strainChickenicken12450,cj1414c).
occurs(strainChickenicken12487,cj1414c).
occurs(strainChickenicken13082,cj1414c).
occurs(strainChickenicken13040,cj1414c).
occurs(strainChickenicken11856,cj1414c).
occurs(strainClinical36069,cj1414c).
occurs(strainChicken40209,cj1414c).
occurs(strainBeach1793,cj1414c).
occurs(strainClinical56519,cj1414c).
occurs(strainClinical44119,cj1414c).
occurs(strainClinical34007,cj1414c).
occurs(strainClinicalinical15168,cj1414c).
occurs(strainClinical18836,cj1414c).
occurs(strainClinical38857,cj1414c).
occurs(strainCjejuniRM1221,cj1414c).
occurs(strainChicken47693,cj1414c).
```

occurs(strainClinical63326,cj1414c).
occurs(strainClinical64555,cj1414c).
occurs(strainClinical59364,cj1414c).
occurs(strainClinical59424,cj1414c).
occurs(strainClinical33084,cj1414c).
occurs(strainClinical31481,cj1414c).
occurs(strainClinical39828,cj1414c).
occurs(strainBeach1771,cj1414c).
occurs(strainClinical53250,cj1414c).
occurs(strainClinical45557,cj1414c).
occurs(strainClinical47939,cj1414c).
occurs(strainClinicalG1,cj1415c).
occurs(strainClinicalG3,cj1415c).
occurs(strainChickenicken11974,cj1415c).
occurs(strainChickenicken13249,cj1415c).
occurs(strainChickenicken13411,cj1415c).
occurs(strainClinical44811,cj1415c).
occurs(strainClinical48612,cj1415c).
occurs(strainClinical58473,cj1415c).
occurs(strainChicken11848,cj1415c).
occurs(strainChicken12567,cj1415c).
occurs(strainChicken11919,cj1415c).
occurs(strainChicken11973,cj1415c).
occurs(strainChicken13713,cj1415c).
occurs(strainBeach1791,cj1415c).
occurs(strainClinical43205,cj1415c).
occurs(strainClinical33106,cj1415c).
occurs(strainClinical55703,cj1415c).
occurs(strainClinical30280,cj1415c).
occurs(strainClinical30328,cj1415c).
occurs(strainClinical41651,cj1415c).
occurs(strainClinical32799,cj1415c).
occurs(strainClinical43983,cj1415c).
occurs(strainClinical40671,cj1415c).
occurs(strainClinical44958,cj1415c).
occurs(strainClinical52331,cj1415c).
occurs(strainClinical56281,cj1415c).
occurs(strainClinical56282,cj1415c).
occurs(strainClinical56832,cj1415c).
occurs(strainOvine12241,cj1415c).
occurs(strainOvine12481,cj1415c).
occurs(strainBovinevineine13305,cj1415c).
occurs(strainChickenicken12912,cj1415c).
occurs(strainChickenicken11818,cj1415c).
occurs(strainChicken12196,cj1415c).
occurs(strainChickenicken12450,cj1415c).
occurs(strainChickenicken12487,cj1415c).
occurs(strainChickenicken13082,cj1415c).
occurs(strainChickenicken13040,cj1415c).
occurs(strainChickenicken11856,cj1415c).
occurs(strainChicken40209,cj1415c).
occurs(strainBeach1793,cj1415c).
occurs(strainClinical32787,cj1415c).
occurs(strainClinicall31467,cj1415c).

occurs(strainClinical38762,cj1415c).
occurs(strainClinicalinical15168,cj1415c).
occurs(strainClinical18836,cj1415c).
occurs(strainClinical38857,cj1415c).
occurs(strainCjejuniRM1221,cj1415c).
occurs(strainClinical33084,cj1415c).
occurs(strainClinical31481,cj1415c).
occurs(strainClinical39828,cj1415c).
occurs(strainClinical45557,cj1415c).
occurs(strainClinicalG1,cj1416c).
occurs(strainClinicalG3,cj1416c).
occurs(strainChickenicken13411,cj1416c).
occurs(strainClinical33106,cj1416c).
occurs(strainChicken40209,cj1416c).
occurs(strainBeach1793,cj1416c).
occurs(strainClinical81116,cj1416c).
occurs(strainClinical32787,cj1416c).
occurs(strainClinicall31467,cj1416c).
occurs(strainClinical38762,cj1416c).
occurs(strainClinicalinical15168,cj1416c).
occurs(strainClinical18836,cj1416c).
occurs(strainClinicalM1,cj1416c).
occurs(strainClinical36860,cj1416c).
occurs(strainClinical40917,cj1416c).
occurs(strainClinical38857,cj1416c).
occurs(strainChicken47693,cj1416c).
occurs(strainClinical33084,cj1416c).
occurs(strainClinical31481,cj1416c).
occurs(strainClinical39828,cj1416c).
occurs(strainClinical53250,cj1416c).
occurs(strainClinical45557,cj1416c).
occurs(strainClinicalG1,cj1417c).
occurs(strainClinicalG3,cj1417c).
occurs(strainChickenicken11974,cj1417c).
occurs(strainChickenicken13249,cj1417c).
occurs(strainChickenicken13411,cj1417c).
occurs(strainClinical44811,cj1417c).
occurs(strainClinical48612,cj1417c).
occurs(strainClinical58473,cj1417c).
occurs(strainChicken11848,cj1417c).
occurs(strainChicken12567,cj1417c).
occurs(strainChicken11919,cj1417c).
occurs(strainChicken11973,cj1417c).
occurs(strainChicken13713,cj1417c).
occurs(strainBeach1791,cj1417c).
occurs(strainClinical43205,cj1417c).
occurs(strainClinical33106,cj1417c).
occurs(strainClinical55703,cj1417c).
occurs(strainClinical30280,cj1417c).
occurs(strainClinical30328,cj1417c).
occurs(strainClinical41651,cj1417c).
occurs(strainClinical32799,cj1417c).
occurs(strainClinical43983,cj1417c).
occurs(strainClinical40671,cj1417c).

occurs(strainClinical44958,cj1417c).
occurs(strainClinical52331,cj1417c).
occurs(strainClinical56281,cj1417c).
occurs(strainClinical56282,cj1417c).
occurs(strainClinical56832,cj1417c).
occurs(strainOvine12241,cj1417c).
occurs(strainOvine12481,cj1417c).
occurs(strainBovinevineine13305,cj1417c).
occurs(strainChickenicken12912,cj1417c).
occurs(strainChickenicken11818,cj1417c).
occurs(strainChicken12196,cj1417c).
occurs(strainChickenicken12450,cj1417c).
occurs(strainChickenicken12487,cj1417c).
occurs(strainChickenicken13082,cj1417c).
occurs(strainChickenicken13040,cj1417c).
occurs(strainChickenicken11856,cj1417c).
occurs(strainChicken40209,cj1417c).
occurs(strainBeach1793,cj1417c).
occurs(strainClinical81116,cj1417c).
occurs(strainClinical56519,cj1417c).
occurs(strainClinical32787,cj1417c).
occurs(strainClinicall31467,cj1417c).
occurs(strainClinical38762,cj1417c).
occurs(strainClinicalinical15168,cj1417c).
occurs(strainClinical18836,cj1417c).
occurs(strainClinicalM1,cj1417c).
occurs(strainClinical36860,cj1417c).
occurs(strainClinical40917,cj1417c).
occurs(strainClinical38857,cj1417c).
occurs(strainChicken47693,cj1417c).
occurs(strainClinical33084,cj1417c).
occurs(strainClinical31481,cj1417c).
occurs(strainClinical39828,cj1417c).
occurs(strainClinical53250,cj1417c).
occurs(strainClinical45557,cj1417c).
occurs(strainClinicalG1,cj1418c).
occurs(strainClinicalG3,cj1418c).
occurs(strainChickenicken11974,cj1418c).
occurs(strainChickenicken13249,cj1418c).
occurs(strainChickenicken13411,cj1418c).
occurs(strainClinical44811,cj1418c).
occurs(strainClinical48612,cj1418c).
occurs(strainClinical58473,cj1418c).
occurs(strainChicken11848,cj1418c).
occurs(strainChicken12567,cj1418c).
occurs(strainChicken11919,cj1418c).
occurs(strainChicken11973,cj1418c).
occurs(strainChicken13713,cj1418c).
occurs(strainBeach1791,cj1418c).
occurs(strainClinical43205,cj1418c).
occurs(strainClinical33106,cj1418c).
occurs(strainClinical55703,cj1418c).
occurs(strainClinical30280,cj1418c).
occurs(strainClinical30328,cj1418c).

occurs(strainClinical41651,cj1418c).
occurs(strainClinical32799,cj1418c).
occurs(strainClinical43983,cj1418c).
occurs(strainClinical40671,cj1418c).
occurs(strainClinical44958,cj1418c).
occurs(strainClinical52331,cj1418c).
occurs(strainClinical56281,cj1418c).
occurs(strainClinical56282,cj1418c).
occurs(strainClinical56832,cj1418c).
occurs(strainOvine12241,cj1418c).
occurs(strainOvine12481,cj1418c).
occurs(strainBovinevineine13305,cj1418c).
occurs(strainChickenicken12912,cj1418c).
occurs(strainChickenicken11818,cj1418c).
occurs(strainChicken12196,cj1418c).
occurs(strainChickenicken12450,cj1418c).
occurs(strainChickenicken12487,cj1418c).
occurs(strainChickenicken13082,cj1418c).
occurs(strainChickenicken13040,cj1418c).
occurs(strainChickenicken11856,cj1418c).
occurs(strainChicken40209,cj1418c).
occurs(strainBeach1793,cj1418c).
occurs(strainClinical81116,cj1418c).
occurs(strainClinical56519,cj1418c).
occurs(strainClinical32787,cj1418c).
occurs(strainClinicall31467,cj1418c).
occurs(strainClinical38762,cj1418c).
occurs(strainClinicalinical15168,cj1418c).
occurs(strainClinical18836,cj1418c).
occurs(strainClinicalM1,cj1418c).
occurs(strainClinical36860,cj1418c).
occurs(strainClinical40917,cj1418c).
occurs(strainClinical38857,cj1418c).
occurs(strainChicken47693,cj1418c).
occurs(strainClinical33084,cj1418c).
occurs(strainClinical31481,cj1418c).
occurs(strainClinical39828,cj1418c).
occurs(strainClinical53250,cj1418c).
occurs(strainClinical45557,cj1418c).
occurs(strainClinicalG1,cj1419c).
occurs(strainClinicalG3,cj1419c).
occurs(strainChickenicken11974,cj1419c).
occurs(strainChickenicken13249,cj1419c).
occurs(strainChickenicken13411,cj1419c).
occurs(strainClinical44811,cj1419c).
occurs(strainClinical48612,cj1419c).
occurs(strainClinical58473,cj1419c).
occurs(strainChicken11848,cj1419c).
occurs(strainChicken12567,cj1419c).
occurs(strainChicken11919,cj1419c).
occurs(strainChicken11973,cj1419c).
occurs(strainChicken13713,cj1419c).
occurs(strainBeach1791,cj1419c).
occurs(strainClinical43205,cj1419c).

occurs(strainClinical33106,cj1419c).
occurs(strainClinical55703,cj1419c).
occurs(strainClinical30280,cj1419c).
occurs(strainClinical30328,cj1419c).
occurs(strainClinical41651,cj1419c).
occurs(strainClinical32799,cj1419c).
occurs(strainClinical43983,cj1419c).
occurs(strainClinical40671,cj1419c).
occurs(strainClinical44958,cj1419c).
occurs(strainClinical52331,cj1419c).
occurs(strainClinical56281,cj1419c).
occurs(strainClinical56282,cj1419c).
occurs(strainClinical56832,cj1419c).
occurs(strainOvine12241,cj1419c).
occurs(strainOvine12481,cj1419c).
occurs(strainBovinevineine13305,cj1419c).
occurs(strainChickenicken12912,cj1419c).
occurs(strainChickenicken11818,cj1419c).
occurs(strainChicken12196,cj1419c).
occurs(strainChickenicken12450,cj1419c).
occurs(strainChickenicken12487,cj1419c).
occurs(strainChickenicken13082,cj1419c).
occurs(strainChickenicken13040,cj1419c).
occurs(strainChickenicken11856,cj1419c).
occurs(strainChicken40209,cj1419c).
occurs(strainBeach1793,cj1419c).
occurs(strainClinical81116,cj1419c).
occurs(strainClinical56519,cj1419c).
occurs(strainClinical32787,cj1419c).
occurs(strainClinicall31467,cj1419c).
occurs(strainClinical38762,cj1419c).
occurs(strainClinicalinical15168,cj1419c).
occurs(strainClinical18836,cj1419c).
occurs(strainClinicalM1,cj1419c).
occurs(strainClinical36860,cj1419c).
occurs(strainClinical40917,cj1419c).
occurs(strainClinical38857,cj1419c).
occurs(strainChicken47693,cj1419c).
occurs(strainClinical33084,cj1419c).
occurs(strainClinical31481,cj1419c).
occurs(strainClinical39828,cj1419c).
occurs(strainClinical53250,cj1419c).
occurs(strainClinical45557,cj1419c).
occurs(strainCjejuniRM1221,cj1421c).
occurs(strainChickenicken13411,cj1423c).
occurs(strainClinical44811,cj1423c).
occurs(strainClinical48612,cj1423c).
occurs(strainChicken11848,cj1423c).
occurs(strainChicken12567,cj1423c).
occurs(strainChicken11919,cj1423c).
occurs(strainChicken11973,cj1423c).
occurs(strainChicken13713,cj1423c).
occurs(strainBeach1791,cj1423c).
occurs(strainClinical43205,cj1423c).

occurs(strainClinical55320,cj1423c).
occurs(strainClinical55703,cj1423c).
occurs(strainClinical30280,cj1423c).
occurs(strainClinical30328,cj1423c).
occurs(strainClinical41651,cj1423c).
occurs(strainClinical44958,cj1423c).
occurs(strainClinical56281,cj1423c).
occurs(strainClinical56282,cj1423c).
occurs(strainChickenicken12450,cj1423c).
occurs(strainChickenicken12487,cj1423c).
occurs(strainChickenicken13082,cj1423c).
occurs(strainBeach1793,cj1423c).
occurs(strainClinical33084,cj1423c).
occurs(strainClinical53250,cj1423c).
occurs(strainChickenicken13411,cj1424c).
occurs(strainClinical44811,cj1424c).
occurs(strainClinical48612,cj1424c).
occurs(strainChicken11848,cj1424c).
occurs(strainChicken12567,cj1424c).
occurs(strainChicken11919,cj1424c).
occurs(strainChicken11973,cj1424c).
occurs(strainChicken13713,cj1424c).
occurs(strainBeach1791,cj1424c).
occurs(strainClinical43205,cj1424c).
occurs(strainClinicall38556,cj1424c).
occurs(strainClinicall39182,cj1424c).
occurs(strainClinical45631,cj1424c).
occurs(strainClinical55320,cj1424c).
occurs(strainClinical55703,cj1424c).
occurs(strainClinical30280,cj1424c).
occurs(strainClinical30328,cj1424c).
occurs(strainClinical41651,cj1424c).
occurs(strainClinical43983,cj1424c).
occurs(strainClinical40671,cj1424c).
occurs(strainClinical56282,cj1424c).
occurs(strainOvine12241,cj1424c).
occurs(strainOvine12481,cj1424c).
occurs(strainBovinevineine13305,cj1424c).
occurs(strainChickenicken12450,cj1424c).
occurs(strainChickenicken12487,cj1424c).
occurs(strainChickenicken13082,cj1424c).
occurs(strainChickenicken11856,cj1424c).
occurs(strainClinical36069,cj1424c).
occurs(strainClinical32787,cj1424c).
occurs(strainClinicall31467,cj1424c).
occurs(strainClinical44119,cj1424c).
occurs(strainClinical34007,cj1424c).
occurs(strainClinical38762,cj1424c).
occurs(strainClinical38857,cj1424c).
occurs(strainChicken47693,cj1424c).
occurs(strainClinical63326,cj1424c).
occurs(strainClinical64555,cj1424c).
occurs(strainClinical59364,cj1424c).
occurs(strainClinical59424,cj1424c).

occurs(strainClinical33084,cj1424c).
occurs(strainClinical31481,cj1424c).
occurs(strainBeach1771,cj1424c).
occurs(strainClinical53250,cj1424c).
occurs(strainChickenicken13411,cj1425c).
occurs(strainClinical44811,cj1425c).
occurs(strainClinical48612,cj1425c).
occurs(strainClinical58473,cj1425c).
occurs(strainChicken11848,cj1425c).
occurs(strainChicken12567,cj1425c).
occurs(strainChicken11919,cj1425c).
occurs(strainChicken11973,cj1425c).
occurs(strainChicken13713,cj1425c).
occurs(strainBeach1791,cj1425c).
occurs(strainClinical43205,cj1425c).
occurs(strainClinical33106,cj1425c).
occurs(strainClinical44933,cj1425c).
occurs(strainClinical55320,cj1425c).
occurs(strainClinical55703,cj1425c).
occurs(strainClinical30280,cj1425c).
occurs(strainClinical30328,cj1425c).
occurs(strainClinical41651,cj1425c).
occurs(strainClinical43983,cj1425c).
occurs(strainClinical40671,cj1425c).
occurs(strainClinical44958,cj1425c).
occurs(strainClinical56281,cj1425c).
occurs(strainClinical56282,cj1425c).
occurs(strainClinical56832,cj1425c).
occurs(strainOvine12241,cj1425c).
occurs(strainChicken12196,cj1425c).
occurs(strainChickenicken12450,cj1425c).
occurs(strainChickenicken12487,cj1425c).
occurs(strainChickenicken13082,cj1425c).
occurs(strainChickenicken13040,cj1425c).
occurs(strainChickenicken11856,cj1425c).
occurs(strainBeach1793,cj1425c).
occurs(strainClinical56519,cj1425c).
occurs(strainClinicall31467,cj1425c).
occurs(strainClinical38857,cj1425c).
occurs(strainChicken47693,cj1425c).
occurs(strainClinical64555,cj1425c).
occurs(strainClinical31481,cj1425c).
occurs(strainBeach1771,cj1425c).
occurs(strainClinical53250,cj1425c).
occurs(strainChickenicken13411,cj1426c).
occurs(strainCjejuniRM1221,cj1426c).
occurs(strainChickenicken13411,cj1427c).
occurs(strainClinical44811,cj1427c).
occurs(strainClinical48612,cj1427c).
occurs(strainChicken11848,cj1427c).
occurs(strainChicken12567,cj1427c).
occurs(strainChicken11919,cj1427c).
occurs(strainChicken11973,cj1427c).
occurs(strainChicken13713,cj1427c).

occurs(strainBeach1791,cj1427c).
occurs(strainClinical43205,cj1427c).
occurs(strainClinical33106,cj1427c).
occurs(strainClinical55703,cj1427c).
occurs(strainClinical30280,cj1427c).
occurs(strainClinical30328,cj1427c).
occurs(strainClinical43983,cj1427c).
occurs(strainClinical40671,cj1427c).
occurs(strainOvine12241,cj1427c).
occurs(strainOvine12481,cj1427c).
occurs(strainBovinevineine13305,cj1427c).
occurs(strainChickenicken12450,cj1427c).
occurs(strainChickenicken12487,cj1427c).
occurs(strainChickenicken13082,cj1427c).
occurs(strainClinical33084,cj1427c).
occurs(strainChickenicken13411,cj1428c).
occurs(strainClinical44811,cj1428c).
occurs(strainClinical48612,cj1428c).
occurs(strainChicken11848,cj1428c).
occurs(strainChicken12567,cj1428c).
occurs(strainChicken11919,cj1428c).
occurs(strainChicken11973,cj1428c).
occurs(strainChicken13713,cj1428c).
occurs(strainBeach1791,cj1428c).
occurs(strainClinical55703,cj1428c).
occurs(strainClinical30280,cj1428c).
occurs(strainClinical30328,cj1428c).
occurs(strainChickenicken12450,cj1428c).
occurs(strainChickenicken12487,cj1428c).
occurs(strainChickenicken13082,cj1428c).
occurs(strainBeach1793,cj1428c).
occurs(strainChickenicken13411,cj1429c).
occurs(strainClinical44811,cj1429c).
occurs(strainClinical48612,cj1429c).
occurs(strainChicken11848,cj1429c).
occurs(strainChicken12567,cj1429c).
occurs(strainChicken11919,cj1429c).
occurs(strainChicken11973,cj1429c).
occurs(strainChicken13713,cj1429c).
occurs(strainBeach1791,cj1429c).
occurs(strainClinical55703,cj1429c).
occurs(strainClinical30280,cj1429c).
occurs(strainClinical30328,cj1429c).
occurs(strainChickenicken12450,cj1429c).
occurs(strainChickenicken12487,cj1429c).
occurs(strainChickenicken13082,cj1429c).
occurs(strainBeach1793,cj1429c).
occurs(strainClinical38857,cj1429c).
occurs(strainChickenicken13411,cj1430c).
occurs(strainClinical44811,cj1430c).
occurs(strainClinical48612,cj1430c).
occurs(strainChicken11848,cj1430c).
occurs(strainChicken12567,cj1430c).
occurs(strainChicken11919,cj1430c).

```
occurs(strainChicken11973,cj1430c).
occurs(strainChicken13713,cj1430c).
occurs(strainBeach1791,cj1430c).
occurs(strainClinical55320,cj1430c).
occurs(strainClinical55703,cj1430c).
occurs(strainClinical30280,cj1430c).
occurs(strainClinical30328,cj1430c).
occurs(strainChickenicken12450,cj1430c).
occurs(strainChickenicken12487,cj1430c).
occurs(strainChickenicken13082,cj1430c).
occurs(strainBeach1793,cj1430c).
occurs(strainChickenicken13411,cj1431c).
occurs(strainChicken40209,cj1431c).
occurs(strainChickenicken13411,cj1432c).
occurs(strainClinical44811,cj1432c).
occurs(strainClinical48612,cj1432c).
occurs(strainChicken11848,cj1432c).
occurs(strainChicken12567,cj1432c).
occurs(strainChicken11919,cj1432c).
occurs(strainChicken11973,cj1432c).
occurs(strainChicken13713,cj1432c).
occurs(strainBeach1791,cj1432c).
occurs(strainClinical55320,cj1432c).
occurs(strainClinical55703,cj1432c).
occurs(strainClinical30280,cj1432c).
occurs(strainClinical30328,cj1432c).
occurs(strainChickenicken12450,cj1432c).
occurs(strainChickenicken12487,cj1432c).
occurs(strainChickenicken13082,cj1432c).
occurs(strainChickenicken13411,cj1433c).
occurs(strainClinical44811,cj1433c).
occurs(strainClinical48612,cj1433c).
occurs(strainChicken11848,cj1433c).
occurs(strainChicken12567,cj1433c).
occurs(strainChicken11919,cj1433c).
occurs(strainChicken11973,cj1433c).
occurs(strainChicken13713,cj1433c).
occurs(strainBeach1791,cj1433c).
occurs(strainClinical55320,cj1433c).
occurs(strainClinical55703,cj1433c).
occurs(strainClinical30280,cj1433c).
occurs(strainClinical30328,cj1433c).
occurs(strainClinical43983,cj1433c).
occurs(strainChickenicken12450,cj1433c).
occurs(strainChickenicken12487,cj1433c).
occurs(strainChickenicken13082,cj1433c).
occurs(strainChickenicken13411,cj1434c).
occurs(strainClinical44811,cj1434c).
occurs(strainClinical48612,cj1434c).
occurs(strainChicken11848,cj1434c).
occurs(strainChicken11919,cj1434c).
occurs(strainChicken11973,cj1434c).
occurs(strainChicken13713,cj1434c).
occurs(strainBeach1791,cj1434c).
```

occurs(strainClinical55703,cj1434c).
occurs(strainClinical30280,cj1434c).
occurs(strainClinical30328,cj1434c).
occurs(strainChickenicken12450,cj1434c).
occurs(strainChickenicken12487,cj1434c).
occurs(strainChickenicken13082,cj1434c).
occurs(strainChickenicken13411,cj1435c).
occurs(strainClinical44811,cj1435c).
occurs(strainClinical48612,cj1435c).
occurs(strainChicken11848,cj1435c).
occurs(strainChicken12567,cj1435c).
occurs(strainChicken11919,cj1435c).
occurs(strainChicken11973,cj1435c).
occurs(strainChicken13713,cj1435c).
occurs(strainBeach1791,cj1435c).
occurs(strainClinical55703,cj1435c).
occurs(strainClinical30280,cj1435c).
occurs(strainClinical30328,cj1435c).
occurs(strainClinical43983,cj1435c).
occurs(strainChickenicken12450,cj1435c).
occurs(strainChickenicken12487,cj1435c).
occurs(strainChickenicken13082,cj1435c).
occurs(strainClinical36439,cj1435c).
occurs(strainChickenicken13411,cj1436c).
occurs(strainClinical44811,cj1436c).
occurs(strainClinical48612,cj1436c).
occurs(strainChicken11848,cj1436c).
occurs(strainChicken12567,cj1436c).
occurs(strainChicken11919,cj1436c).
occurs(strainChicken11973,cj1436c).
occurs(strainChicken13713,cj1436c).
occurs(strainBeach1791,cj1436c).
occurs(strainClinical55320,cj1436c).
occurs(strainClinical55703,cj1436c).
occurs(strainClinical30280,cj1436c).
occurs(strainClinical30328,cj1436c).
occurs(strainChickenicken12450,cj1436c).
occurs(strainChickenicken12487,cj1436c).
occurs(strainChickenicken13082,cj1436c).
occurs(strainChickenicken13411,cj1437c).
occurs(strainClinical44811,cj1437c).
occurs(strainClinical48612,cj1437c).
occurs(strainChicken11848,cj1437c).
occurs(strainChicken12567,cj1437c).
occurs(strainChicken11919,cj1437c).
occurs(strainChicken11973,cj1437c).
occurs(strainChicken13713,cj1437c).
occurs(strainBeach1791,cj1437c).
occurs(strainClinical55703,cj1437c).
occurs(strainClinical30280,cj1437c).
occurs(strainClinical30328,cj1437c).
occurs(strainChickenicken12450,cj1437c).
occurs(strainChickenicken12487,cj1437c).
occurs(strainChickenicken13082,cj1437c).

occurs(strainChickenicken13411,cj1438c).
occurs(strainClinical44811,cj1438c).
occurs(strainClinical48612,cj1438c).
occurs(strainChicken11848,cj1438c).
occurs(strainChicken12567,cj1438c).
occurs(strainChicken11919,cj1438c).
occurs(strainChicken11973,cj1438c).
occurs(strainChicken13713,cj1438c).
occurs(strainBeach1791,cj1438c).
occurs(strainClinical55703,cj1438c).
occurs(strainClinical30280,cj1438c).
occurs(strainClinical30328,cj1438c).
occurs(strainChickenicken12450,cj1438c).
occurs(strainChickenicken12487,cj1438c).
occurs(strainChickenicken13082,cj1438c).
occurs(strainChickenicken13411,cj1440c).
occurs(strainClinical44811,cj1440c).
occurs(strainClinical48612,cj1440c).
occurs(strainChicken11848,cj1440c).
occurs(strainChicken12567,cj1440c).
occurs(strainChicken11919,cj1440c).
occurs(strainChicken11973,cj1440c).
occurs(strainChicken13713,cj1440c).
occurs(strainBeach1791,cj1440c).
occurs(strainClinical55320,cj1440c).
occurs(strainClinical55703,cj1440c).
occurs(strainClinical30280,cj1440c).
occurs(strainClinical30328,cj1440c).
occurs(strainClinical56281,cj1440c).
occurs(strainChickenicken12450,cj1440c).
occurs(strainChickenicken12487,cj1440c).
occurs(strainChickenicken13082,cj1440c).
occurs(strainChickenicken13411,cj1441c).
occurs(strainChickenicken13411,cj1442c).
occurs(strainClinical44811,cj1442c).
occurs(strainClinical48612,cj1442c).
occurs(strainClinical58473,cj1442c).
occurs(strainChicken11848,cj1442c).
occurs(strainChicken12567,cj1442c).
occurs(strainChicken11919,cj1442c).
occurs(strainChicken11973,cj1442c).
occurs(strainChicken13713,cj1442c).
occurs(strainBeach1791,cj1442c).
occurs(strainClinical43205,cj1442c).
occurs(strainClinical55703,cj1442c).
occurs(strainClinical30280,cj1442c).
occurs(strainClinical30328,cj1442c).
occurs(strainClinical41651,cj1442c).
occurs(strainClinical43983,cj1442c).
occurs(strainClinical40671,cj1442c).
occurs(strainClinical44958,cj1442c).
occurs(strainClinical52331,cj1442c).
occurs(strainClinical56281,cj1442c).
occurs(strainClinical56282,cj1442c).

occurs(strainOvine12241,cj1442c).
occurs(strainOvine12481,cj1442c).
occurs(strainBovinevineine13305,cj1442c).
occurs(strainChickenicken11818,cj1442c).
occurs(strainChicken12196,cj1442c).
occurs(strainChickenicken12450,cj1442c).
occurs(strainChickenicken12487,cj1442c).
occurs(strainChickenicken13082,cj1442c).
occurs(strainChickenicken13040,cj1442c).
occurs(strainChickenicken11856,cj1442c).
occurs(strainClinical56519,cj1442c).
occurs(strainChicken47693,cj1442c).
occurs(strainBeach1771,cj1442c).
occurs(strainClinical53250,cj1442c).
occurs(strainClinicalG1,cj1443c).
occurs(strainClinicalG3,cj1443c).
occurs(strainChickenicken11974,cj1443c).
occurs(strainChickenicken13249,cj1443c).
occurs(strainChickenicken13411,cj1443c).
occurs(strainClinical44811,cj1443c).
occurs(strainClinical48612,cj1443c).
occurs(strainClinical58473,cj1443c).
occurs(strainChicken11848,cj1443c).
occurs(strainChicken12567,cj1443c).
occurs(strainChicken11919,cj1443c).
occurs(strainChicken11973,cj1443c).
occurs(strainChicken13713,cj1443c).
occurs(strainBeach1791,cj1443c).
occurs(strainClinical43205,cj1443c).
occurs(strainClinical33106,cj1443c).
occurs(strainClinicall38556,cj1443c).
occurs(strainClinicall39182,cj1443c).
occurs(strainClinical44933,cj1443c).
occurs(strainClinical45631,cj1443c).
occurs(strainClinical55320,cj1443c).
occurs(strainClinical55703,cj1443c).
occurs(strainClinical30280,cj1443c).
occurs(strainClinical30328,cj1443c).
occurs(strainClinical41651,cj1443c).
occurs(strainClinical32799,cj1443c).
occurs(strainClinical43983,cj1443c).
occurs(strainClinical40671,cj1443c).
occurs(strainClinical44958,cj1443c).
occurs(strainClinical52331,cj1443c).
occurs(strainClinical56281,cj1443c).
occurs(strainClinical56282,cj1443c).
occurs(strainClinical56832,cj1443c).
occurs(strainOvine12241,cj1443c).
occurs(strainOvine12481,cj1443c).
occurs(strainBovinevineine13305,cj1443c).
occurs(strainChicken12196,cj1443c).
occurs(strainChickenicken12450,cj1443c).
occurs(strainChickenicken12487,cj1443c).
occurs(strainChickenicken13082,cj1443c).

occurs(strainChickenicken13040,cj1443c).
occurs(strainChickenicken11856,cj1443c).
occurs(strainClinical36069,cj1443c).
occurs(strainChicken40209,cj1443c).
occurs(strainBeach1793,cj1443c).
occurs(strainClinical81116,cj1443c).
occurs(strainClinical56519,cj1443c).
occurs(strainClinical44119,cj1443c).
occurs(strainClinical34007,cj1443c).
occurs(strainClinicalinical15168,cj1443c).
occurs(strainClinical18836,cj1443c).
occurs(strainClinicalM1,cj1443c).
occurs(strainClinical36860,cj1443c).
occurs(strainClinical40917,cj1443c).
occurs(strainClinical38857,cj1443c).
occurs(strainChicken47693,cj1443c).
occurs(strainClinical63326,cj1443c).
occurs(strainClinical64555,cj1443c).
occurs(strainClinical59364,cj1443c).
occurs(strainClinical59424,cj1443c).
occurs(strainClinical33084,cj1443c).
occurs(strainClinical31481,cj1443c).
occurs(strainClinical39828,cj1443c).
occurs(strainBeach1771,cj1443c).
occurs(strainClinical53250,cj1443c).
occurs(strainClinical45557,cj1443c).
occurs(strainClinical47939,cj1443c).
occurs(strainClinicalG1,cj1444c).
occurs(strainClinicalG3,cj1444c).
occurs(strainChickenicken11974,cj1444c).
occurs(strainChickenicken13249,cj1444c).
occurs(strainChickenicken13411,cj1444c).
occurs(strainClinical44811,cj1444c).
occurs(strainClinical48612,cj1444c).
occurs(strainClinical58473,cj1444c).
occurs(strainChicken11848,cj1444c).
occurs(strainChicken12567,cj1444c).
occurs(strainChicken11919,cj1444c).
occurs(strainChicken11973,cj1444c).
occurs(strainChicken13713,cj1444c).
occurs(strainBeach1791,cj1444c).
occurs(strainClinical43205,cj1444c).
occurs(strainClinical33106,cj1444c).
occurs(strainClinicall38556,cj1444c).
occurs(strainClinicall39182,cj1444c).
occurs(strainClinical44933,cj1444c).
occurs(strainClinical45631,cj1444c).
occurs(strainClinical55320,cj1444c).
occurs(strainClinical55703,cj1444c).
occurs(strainClinical30280,cj1444c).
occurs(strainClinical30328,cj1444c).
occurs(strainClinical41651,cj1444c).
occurs(strainClinical32799,cj1444c).
occurs(strainClinical43983,cj1444c).

```
occurs(strainClinical40671,cj1444c).
occurs(strainClinical44958,cj1444c).
occurs(strainClinical52331,cj1444c).
occurs(strainClinical56281,cj1444c).
occurs(strainClinical56282,cj1444c).
occurs(strainClinical56832,cj1444c).
occurs(strainOvine12241,cj1444c).
occurs(strainOvine12481,cj1444c).
occurs(strainBovinevineine13305,cj1444c).
occurs(strainChickenicken12912,cj1444c).
occurs(strainChickenicken11818,cj1444c).
occurs(strainChicken12196,cj1444c).
occurs(strainChickenicken12450,cj1444c).
occurs(strainChickenicken12487,cj1444c).
occurs(strainChickenicken13082,cj1444c).
occurs(strainChickenicken13040,cj1444c).
occurs(strainChickenicken11856,cj1444c).
occurs(strainClinical36069,cj1444c).
occurs(strainChicken40209,cj1444c).
occurs(strainBeach1793,cj1444c).
occurs(strainClinical81116,cj1444c).
occurs(strainClinical36439,cj1444c).
occurs(strainClinical56519,cj1444c).
occurs(strainClinical32787,cj1444c).
occurs(strainClinicall31467,cj1444c).
occurs(strainClinical44119,cj1444c).
occurs(strainClinical34007,cj1444c).
occurs(strainClinical38762,cj1444c).
occurs(strainClinicalinical15168,cj1444c).
occurs(strainClinical18836,cj1444c).
occurs(strainClinicalM1,cj1444c).
occurs(strainClinical36860,cj1444c).
occurs(strainClinical40917,cj1444c).
occurs(strainClinical38857,cj1444c).
occurs(strainCjejuniRM1221,cj1444c).
occurs(strainChicken47693,cj1444c).
occurs(strainClinical63326,cj1444c).
occurs(strainClinical64555,cj1444c).
occurs(strainClinical59364,cj1444c).
occurs(strainClinical59424,cj1444c).
occurs(strainClinical33084,cj1444c).
occurs(strainClinical31481,cj1444c).
occurs(strainClinical39828,cj1444c).
occurs(strainBeach1771,cj1444c).
occurs(strainClinical53250,cj1444c).
occurs(strainClinical45557,cj1444c).
occurs(strainClinical47939,cj1444c).
occurs(strainClinicalG1,cj1445c).
occurs(strainChickenicken11974,cj1445c).
occurs(strainChickenicken13249,cj1445c).
occurs(strainChickenicken13411,cj1445c).
occurs(strainClinical44811,cj1445c).
occurs(strainClinical48612,cj1445c).
occurs(strainClinical58473,cj1445c).
```

```
occurs(strainChicken11848,cj1445c).
occurs(strainChicken12567,cj1445c).
occurs(strainChicken11919,cj1445c).
occurs(strainChicken11973,cj1445c).
occurs(strainChicken13713,cj1445c).
occurs(strainBeach1791,cj1445c).
occurs(strainClinical43205,cj1445c).
occurs(strainClinical33106,cj1445c).
occurs(strainClinicall38556,cj1445c).
occurs(strainClinicall39182,cj1445c).
occurs(strainClinical44933,cj1445c).
occurs(strainClinical45631,cj1445c).
occurs(strainClinical55320,cj1445c).
occurs(strainClinical55703,cj1445c).
occurs(strainClinical30280,cj1445c).
occurs(strainClinical30328,cj1445c).
occurs(strainClinical41651,cj1445c).
occurs(strainClinical43983,cj1445c).
occurs(strainClinical40671,cj1445c).
occurs(strainClinical44958,cj1445c).
occurs(strainClinical52331,cj1445c).
occurs(strainClinical56281,cj1445c).
occurs(strainClinical56282,cj1445c).
occurs(strainClinical56832,cj1445c).
occurs(strainOvine12241,cj1445c).
occurs(strainOvine12481,cj1445c).
occurs(strainBovinevineine13305,cj1445c).
occurs(strainChickenicken12912,cj1445c).
occurs(strainChicken12196,cj1445c).
occurs(strainChickenicken12450,cj1445c).
occurs(strainChickenicken12487,cj1445c).
occurs(strainChickenicken13082,cj1445c).
occurs(strainChickenicken13040,cj1445c).
occurs(strainChickenicken11856,cj1445c).
occurs(strainClinical36069,cj1445c).
occurs(strainChicken40209,cj1445c).
occurs(strainBeach1793,cj1445c).
occurs(strainClinical81116,cj1445c).
occurs(strainClinical36439,cj1445c).
occurs(strainClinical56519,cj1445c).
occurs(strainClinical32787,cj1445c).
occurs(strainClinicall31467,cj1445c).
occurs(strainClinical44119,cj1445c).
occurs(strainClinical34007,cj1445c).
occurs(strainClinical38762,cj1445c).
occurs(strainClinicalinical15168,cj1445c).
occurs(strainClinical18836,cj1445c).
occurs(strainClinicalM1,cj1445c).
occurs(strainClinical36860,cj1445c).
occurs(strainClinical40917,cj1445c).
occurs(strainClinical38857,cj1445c).
occurs(strainCjejuniRM1221,cj1445c).
occurs(strainChicken47693,cj1445c).
occurs(strainClinical63326,cj1445c).
```

occurs(strainClinical64555,cj1445c).
occurs(strainClinical59364,cj1445c).
occurs(strainClinical59424,cj1445c).
occurs(strainClinical33084,cj1445c).
occurs(strainClinical31481,cj1445c).
occurs(strainBeach1771,cj1445c).
occurs(strainClinical53250,cj1445c).
occurs(strainClinical45557,cj1445c).
occurs(strainClinical47939,cj1445c).
occurs(strainChickenicken13411,cj1447c).
occurs(strainClinical44811,cj1447c).
occurs(strainClinical48612,cj1447c).
occurs(strainClinical58473,cj1447c).
occurs(strainChicken11848,cj1447c).
occurs(strainChicken12567,cj1447c).
occurs(strainChicken11919,cj1447c).
occurs(strainChicken11973,cj1447c).
occurs(strainChicken13713,cj1447c).
occurs(strainBeach1791,cj1447c).
occurs(strainClinical43205,cj1447c).
occurs(strainClinical55703,cj1447c).
occurs(strainClinical30280,cj1447c).
occurs(strainClinical30328,cj1447c).
occurs(strainChickenicken12912,cj1447c).
occurs(strainChickenicken12450,cj1447c).
occurs(strainChickenicken12487,cj1447c).
occurs(strainChickenicken13082,cj1447c).
occurs(strainChicken40209,cj1447c).
occurs(strainClinical81116,cj1447c).
occurs(strainCjejuniRM1221,cj1447c).
occurs(strainChickenicken13411,cj1448c).
occurs(strainClinical44811,cj1448c).
occurs(strainClinical48612,cj1448c).
occurs(strainClinical58473,cj1448c).
occurs(strainChicken11848,cj1448c).
occurs(strainChicken12567,cj1448c).
occurs(strainChicken11919,cj1448c).
occurs(strainChicken11973,cj1448c).
occurs(strainChicken13713,cj1448c).
occurs(strainBeach1791,cj1448c).
occurs(strainClinical43205,cj1448c).
occurs(strainClinical55320,cj1448c).
occurs(strainClinical55703,cj1448c).
occurs(strainClinical30280,cj1448c).
occurs(strainClinical30328,cj1448c).
occurs(strainChickenicken12912,cj1448c).
occurs(strainChickenicken12450,cj1448c).
occurs(strainChickenicken12487,cj1448c).
occurs(strainChickenicken13082,cj1448c).
occurs(strainChicken40209,cj1448c).
occurs(strainBeach1793,cj1448c).
occurs(strainClinical81116,cj1448c).
occurs(strainClinical36860,cj1448c).
occurs(strainClinical53250,cj1448c).

[Total time taken 0.050s]
